# Performance in eyeblink conditioning is age and sex dependent

**DOI:** 10.1371/journal.pone.0177849

**Published:** 2017-05-18

**Authors:** Karolina Löwgren, Rasmus Bååth, Anders Rasmussen, Henk-Jan Boele, Sebastiaan K. E. Koekkoek, Chris I. De Zeeuw, Germund Hesslow

**Affiliations:** 1 Department of Clinical Sciences, Lund University, Lund, Sweden; 2 Department of Philosophy, Cognitive Science, Lund University, Lund, Sweden; 3 Department of Neuroscience, Erasmus Medical Center, Rotterdam, Netherlands; 4 Netherlands Institute for Neuroscience, Royal Academy of Arts and Sciences, Amsterdam, Netherlands; 5 Department of Experimental Medical Science, Lund University, Lund, Sweden; Tokai University, JAPAN

## Abstract

A growing body of evidence suggests that the cerebellum is involved in both cognition and language. Abnormal cerebellar development may contribute to neurodevelopmental disorders such as attention deficit hyperactivity disorder (ADHD), autism, fetal alcohol syndrome, dyslexia, and specific language impairment. Performance in eyeblink conditioning, which depends on the cerebellum, can potentially be used to clarify the neural mechanisms underlying the cerebellar dysfunction in disorders like these. However, we must first understand how the performance develops in children who do not have a disorder. In this study we assessed the performance in eyeblink conditioning in 42 typically developing children between 6 and 11 years old as well as in 26 adults. Older children produced more conditioned eyeblink responses than younger children and adults produced more than children. In addition, females produced more conditioned eyeblink responses than males among both children and adults. These results highlight the importance of considering the influence of age and sex on the performance when studying eyeblink conditioning as a measure of cerebellar development.

## Introduction

In eyeblink conditioning an originally neutral conditional stimulus (CS), such as a tone, is followed by a reflex eliciting unconditional stimulus (US), such as a puff of air to the cornea. Initially, the subject will produce an unconditional blink response (UR) to the air puff, but if the tone and air puff are paired repeatedly the subject will eventually acquire a conditioned blink response (CR), which begins before the onset of the US and peaks near the expected US. The CR is adapted in time to the specific CS-US interval used. Several lines of evidence demonstrate that the cerebellum plays a critical role in the acquisition and expression of adaptively timed conditioned eyeblink responses [1–8]. This opens up for the possibility that eyeblink conditioning can be used as a measure of cerebellar function and, by extension, of cerebellar dysfunction.

An increasing number of studies indicate that the cerebellum is involved in cognition and language [9–15]. Cerebellar abnormalities have been detected in patients with dyslexia [16], specific language impairment (SLI) [17], and attention deficit hyperactivity disorder (ADHD) [18]. Autism spectrum disorders (ASD) have also been linked to cerebellar abnormalities [19–22], although this link has later been contested [23]. Consistent with the pivotal role of the cerebellum in eyeblink conditioning, children with ADHD [24,25], ASD [26–28], and dyslexia [29] have displayed atypical timing and learning patterns during eyeblink conditioning, although no such effect was found in children with SLI [30,31]. However, the results are inconsistent concerning studies of individuals with ADHD. Other conditions, such as fetal alcohol syndrome (FAS) [24,32,33], fragile X syndrome, Down’s syndrome, and schizophrenia [34,35], have also been linked to cerebellar deficits and poor performance in eyeblink conditioning. Rats exposed to alcohol as neonates suffer from loss of cerebellar neurons and show deficits in eyeblink conditioning as adults [36]. Collectively, these studies constitute strong evidence that factors that influence the cerebellum also affect performance in eyeblink conditioning.

Given that the cerebellum, like the rest of the brain, undergoes changes throughout life [37–39], it is perhaps not surprising that performance in eyeblink conditioning is influenced by age. Adults 20–50 years old, perform better than children and individuals older than 60 years [40–43]. Yet, no age effects were observed in a study comparing adults, adolescents and typically developing children older than 9 years [31]. Surprisingly, five month old infants reach similar levels of CRs as adults, although differences in CR timing remained post training [44]. The aim of this study was to examine performance in eyeblink conditioning in school aged children (6–11 years). Our relatively large sample of 46 children and 30 adults allowed us to correlate age with various CR parameters, including the temporal profile of the CR.

## Methods

### Ethics statement

The regional ethics committee in Lund, Sweden, approved this study and all associated procedures.

### Participants

A total of 76 subjects, 46 children and 30 adults, participated in the study. The children were recruited from two local elementary schools in lower middle class to higher middle class socioeconomic areas in southern Sweden. The age of the pupils ranged from 6 to 12 years old. The adults were recruited mainly from the student population at Lund University. Participants and legal guardians were informed about the purpose of the study and signed an informed consent form prior to testing. Three children and four adults were excluded from the analysis due to technical problems with the registration of eyelid movements. In addition, one child was excluded due to a non-verbal IQ score below 65 (percentile 1), tested by Raven’s colored progressive matrices [45]. Thus, the participants included in the analysis were 42 children, 6–11 years old (mean = 8.8, SD = 1.3) and 26 adults (21 students and 5 former students), 20–55 years old (mean = 29.3, SD = 8.6). Among the children 22 were female (mean age = 9.0 years, SD = 1.4, range 6.8–11.1 years) and 20 were male (mean age = 8.6 years, SD = 1.3, range 6.9–10.6 years). Among the adults 18 were female (mean age = 30.3 years, SD = 9.3, range 21.6–55.9 years) and 8 were male (mean age = 26.8 years, SD = 6.8, range 20.6–40.8 years). All participants were screened for normal hearing with a modified Hughson-Westlake method (ISO 8253–1), at 20 dB hearing level. The average IQ score on Raven’s colored progressive matrices was 105, with a standard deviation of 16 points, for the children. The right hand was the dominant hand for 91.3 percent of the participants. The participants had no eye deficit or disease and all exhibited normal motor development. None of the children received extra support in school or used any medication.

### Procedure

The test sessions took place in a calm and quiet room (Leq 60 seconds = 45 dB(A) measured with Brüel & Kjær 2225 sound level meter), away from school or university activity. The conditioning session lasted 35 minutes on average. The participants watched a movie both as distraction from the test situation and as motivation. It also helped to fixate their gaze and eye position. The children watched a cartoon while the adults watched a classic comedy movie. The movies were selected to vary as little as possible in sound intensity. The eyeblink conditioning was performed using a Shebot (Neurasmus, The Netherlands). This device consisted of a computer, air compressor, air-puff generator, sound generator, magnetic distance measurement technique controlling hardware, movie goggles with a circular air puff opening of 1.5 millimeters in diameter, magneto-sensitive sensor, noise excluding circumaural Sennheiser HD 201 headphones and a helmet that held the goggles and headphones in place. The eyelid movements were measured with the magnetic distance measurement technique [46]. The sensor was placed on the cheek straight below a magnet (~0.1 g, 5 x 3 x 1 mm) that was attached to the eyelid, close to the eyelash. The CS, a 1 kHz tone, was presented binaurally through the headphones at 68 dB SPL during 500 ms. The tone and movie sound were calibrated through the headphones and a coupler connected to an ISO-TECH SLM52N sound level meter. The tone was clearly audible to all of the participants through the soft background sound of the movie of ~41 dB(A), but not strong enough to trigger a blink reflex by itself. The US, a 15 ms air puff of 1 bar, was released through the movie goggles, placed 1–2 centimeters from the eyes, towards the left cornea. In a few cases the duration of the US was adjusted (+/- 5 ms at most), to ensure that it elicited a clear blink reflex, without being perceived as too aversive.

**Fig 1:**
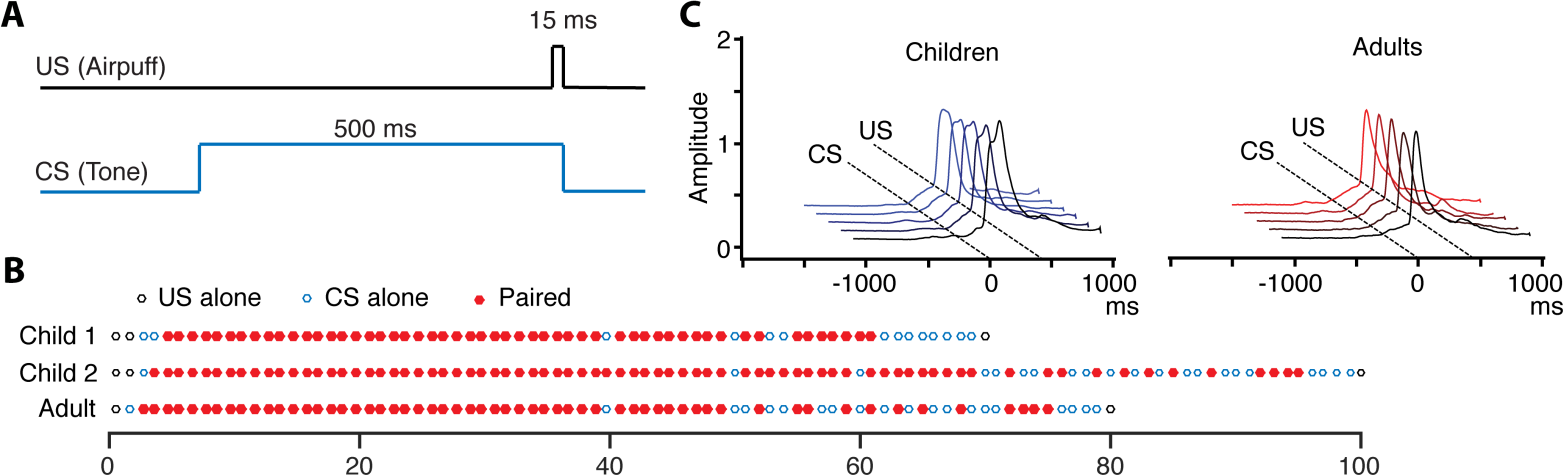
Stimulation protocols and eyeblink responses. **(**A) Onset, offset, and duration of the CS and US. The offsets of the CS and US co-terminate 500 ms after CS onset in paired trials. (B) The distribution of the US alone, CS alone, and paired (CS + US) trials in three different protocols (Child 1 from “School A”, Child 2 from “School B”). (C) Examples of CRs and URs, in paired trials.

A classical eyeblink conditioning delay paradigm, where the CS and US overlapped in time, was programmed in National Instruments LabVIEW 2011. Of the 42 children, 22 received a total of 70 trials (53 paired) while 20 received 100 trials (76 paired). The 26 adults received 80 trials (67 paired). The eyeblink conditioning protocols differed in number of trials since the children at the two schools and the adults had different amounts of time to spend on the eyeblink conditioning sessions. Yet, given that the settings were similar and the protocols, up until a certain time, were nearly identical (Fig 1B), we considered it justified to compare and combine data from these three groups. The inter-trial interval varied randomly between 15 and 25 seconds. In paired trials the CS preceded the US by 485 milliseconds and the two stimuli co-terminated in time (Fig 1A). If a spontaneous blink occurred during the 500 ms preceding the expected CS onset, the trial started over. As illustrated in Fig 1B, the majority of the trials in the three protocols were paired trials and the CS alone trials were concentrated in the last part of the conditioning session, after the first 50 trials. The participants were instructed to try to relax, concentrate on the movie and not pay attention to the stimuli or their own reactions during the experiment. They were informed that the air puffs could feel somewhat stronger in the beginning of the session. Before the conditioning session, the children were presented with 5–10 trials of paired tones and air puffs to make the test situation more familiar.

### Data analysis

The magnetic distance measurement registrations, sampled at 1 kHz, were stored in a SQL database (MySQL Server 5.1, MySQL Workbench 5.2 CE). Eyelid movements were analyzed offline trial-by-trial with a semi-automatic SQL-based visual trial identifier program made in National Instruments LabVIEW 2011. A trial was considered invalid if the registration of the response was too noisy. A CR was defined as an eyeblink with an onset between 100 and 490 ms after the CS onset on paired trials (Fig 1C), or between 100 and 700 ms after CS onset on CS alone trials. For every valid trial with a CR, the onset latency, peak latency, and peak amplitude were determined. The CR onset was defined as the earliest point in time where there was a change in the eyelid position of at least 3 SD, compared to the baseline. The CR peak was defined as the point in time the first eyelid closure appeared after the CR onset. CRs with an amplitude less than 15 percent of a participant’s mean UR amplitude were excluded. The UR was analyzed in terms of onset latency (minimum change of 3 SD compared to the baseline), peak latency (first maximum eyelid closure), and peak amplitude (~100 percent eye closure), in paired and US alone trials. Participants blinked before the US on 2% of the US alone trials.

### Statistical analysis

For the statistical analysis, the conditioning session was divided into blocks of ten trials. Since the protocols differed slightly between the groups after the fifth block (Fig 1B), the analysis was mainly focused on the first five blocks. Average measures of CRs in CS alone trials after the fifth block were also used to describe the level of conditioning post training. When referring to CS alone trials without specifying actual blocks in the result and discussion sections, block 4 and the following blocks until the end of the session were included. CR percentages were used to investigate the learning during conditioning. While analysis of the CR onset was done in both paired and CS alone trials, analysis of the peak latency was done only in CS alone trials since URs often interfered with the peak latency of the CR in paired trials. All the participants were included in the analyses regardless of level of learning. The statistical analyses were made in SPSS Statistics 23 (IBM). With repeated measures ANOVAs the CR measures in the first five blocks as within-subjects factor, and sex and age as between-subjects factors, were analyzed. Greenhouse-Geisser correction were made whenever the assumption of sphericity was violated according to Mauchly’s test of sphericity. Post-hoc pairwise comparisons were made with Bonferroni confidence interval adjustment, with the significance level 0.05. Between-subjects effects were also analyzed with standard linear regression models in different parts of the session (paired trials in block 1, blocks 2–5, and in CS alone trials post training).

## Results

### CR performance

**Fig 2:**
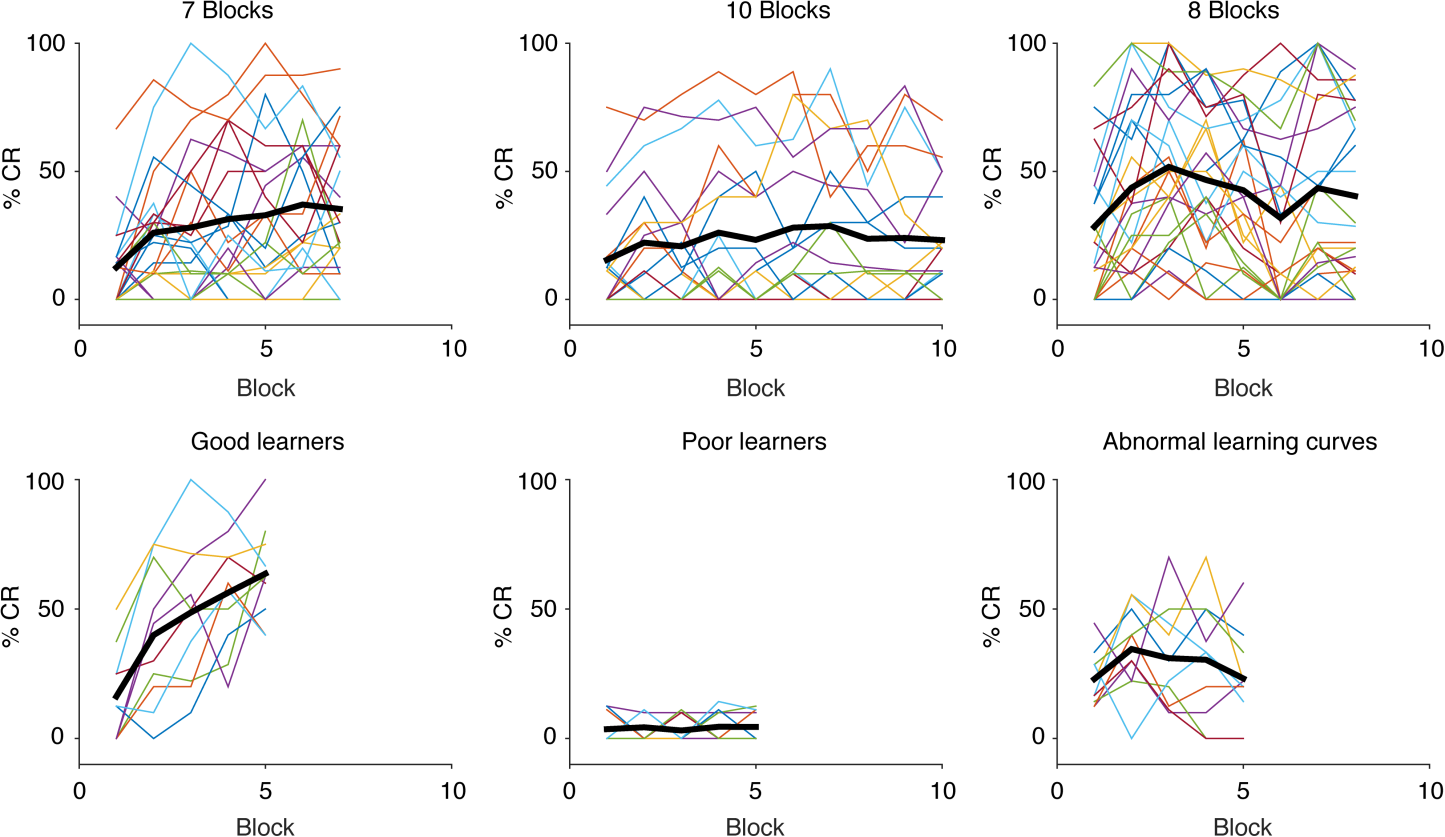
Learning curves of CR percentages over block. The three different session length protocol groups and examples of “Good learners”, “Poor learners” and “Abnormal learning curves” during the first five blocks, with averages in bold.

While the rate of learning varied substantially among participants (Fig 2), repeated measures ANOVAs with percent CRs on successive blocks as within-subjects factors showed that for both the children and the adults, the rate of CRs increases during training (Children: F(4, 152) = 6.601, p = 0.000, η^2^ = 0.148. Adults: F(2.984, 71.625) = 5.656, p = 0.002, η^2^ = 0.191). Post-hoc pairwise comparisons show that the initial increase in CRs levels off between the first and the second block for the children (block 1 vs. 2 p = 0.032, 1 vs. 3 p = 0.037, 1 vs. 4 p = 0.006, 1 vs. 5 p = 0.008), and between the first block and the third block for the adults (block 1 vs. 2 p = 0.538, 1 vs. 3 p = 0.000, 1 vs. 4 p = 0.017, 1 vs. 5 p = 0.138). There are no differences between the rest of the blocks compared to each other (p = 1.000). When comparing the first block to blocks 2–5 combined, the CRs increased with 12 percentage points among the children (t_41_ = 4.269, p = 0.000, 95% CI[1, 17]) and 15 percentage points among the adults (t_25_ = 5.400, p = 0.000, 95% CI[1, 21]) (Table 1). The CRs increase further, though only among the children, with 8 percentage points (t_41_ = 3.642, p = 0.001, 95% CI[3, 12]) from blocks 2–5 to CS alone trials during later parts of the session.

**Table 1:** Average CR percentages (SD) in paired trials and CS alone trials.

	Paired trials		CS alone		
	Block 1	Blocks 2–5	Block 1	Blocks 4–5	Blocks 4–10
**Children (n42)**	**15 (23)**	**27 (25)**	**23 (32)**	**44 (38)**	**34 (26)**
Females (n22)	19 (28)	35 (29)	23 (34)	50 (35)	42 (29)
Males (n20)	11 (16)	18 (18)	23 (30)	39 (42)	26 (19)
**Adults (n26)**	**32 (31)**	**47 (28)**	**28 (46)**	**56 (39)**	**46 (33)**
Females (n18)	40 (33)	56 (28)	35 (49)	73 (34)	52 (33)
Males (n8)	14 (18)	27 (17)	13 (35)	25 (27)	30 (30)

#### Effects of age and sex on the CR performance

**Fig 3:**
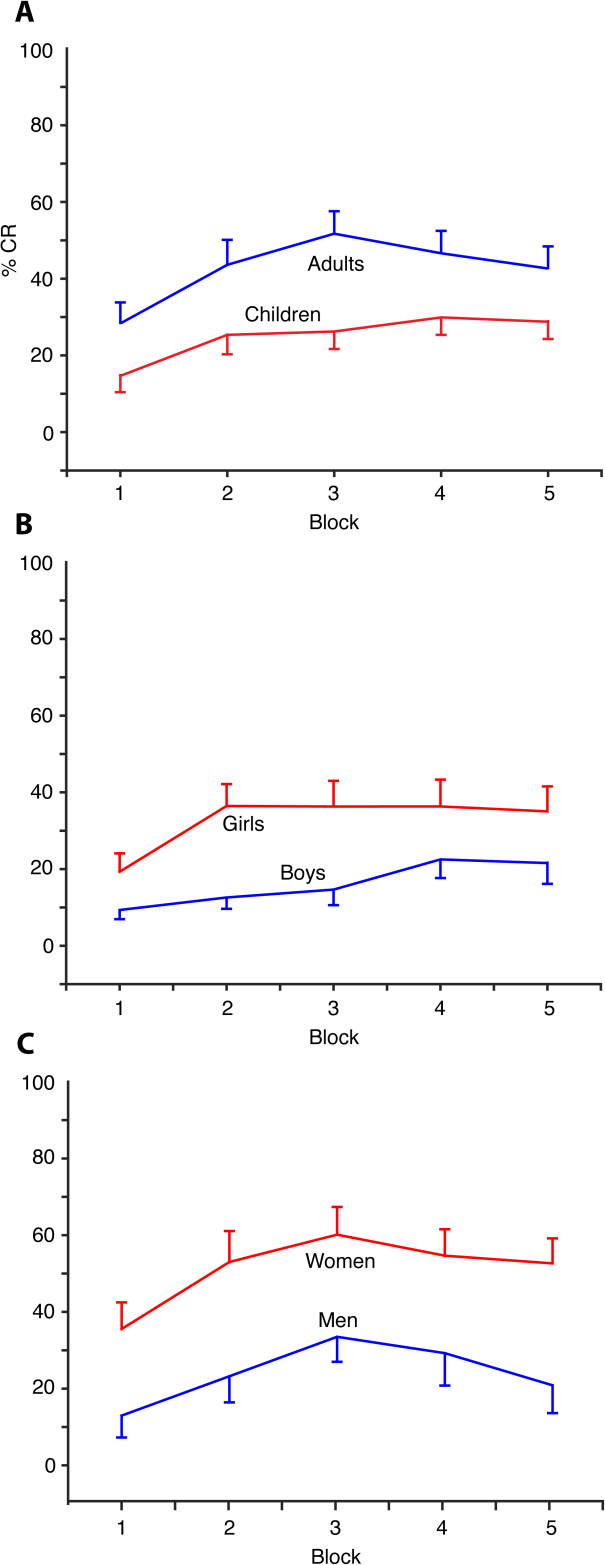
CR percentages in blocks 1–5 (mean ± SEM). The results differ between groups based on age and sex. The adults produced more CRs than the children and the females produced more CRs than the males. (A) Adults (n 26) and children (n 42). (B) Girls (n 22) and boys (n 20). (C) Women (n 18) and men (n 8).

**Fig 4:**
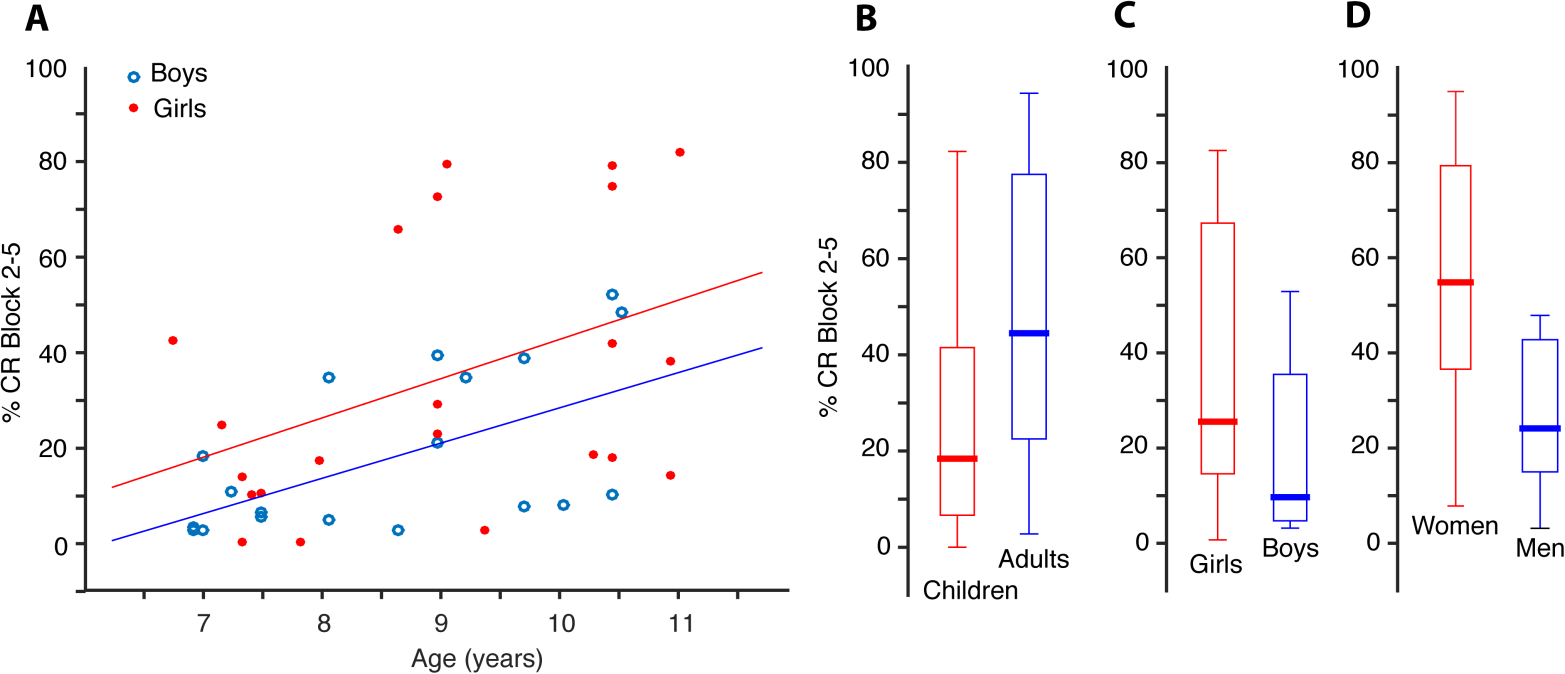
Age and sex influences rate of CRs. (A) Scatterplot illustrating average CR percentage in blocks 2–5 as a function of age of the children. (B) Boxplots showing the distribution of average CR percentage performed by the children (n 42) and adults (n 26) and (C and D) divided into groups of females and males.

As illustrated in Fig 3, the children produced on average 20 percentage points fewer CRs than the adults (t_48.52_ = -3.027, p = 0.003, 95% CI[-34, -7]). The difference is only significant in blocks 2–5. This age effect is also present when examining only the children. Pearson product-moment correlation coefficient shows positive correlations between age and percentage of CRs in the first block (r = 0.328, p = 0.034, n = 42), in blocks 2–5 (r = 0.476, p = 0.001, n = 42), and in the CS alone trials (r = 0.490, p = 0.001, n = 42) (Fig 4). No such correlation is present among the adults.

**Fig 5:**
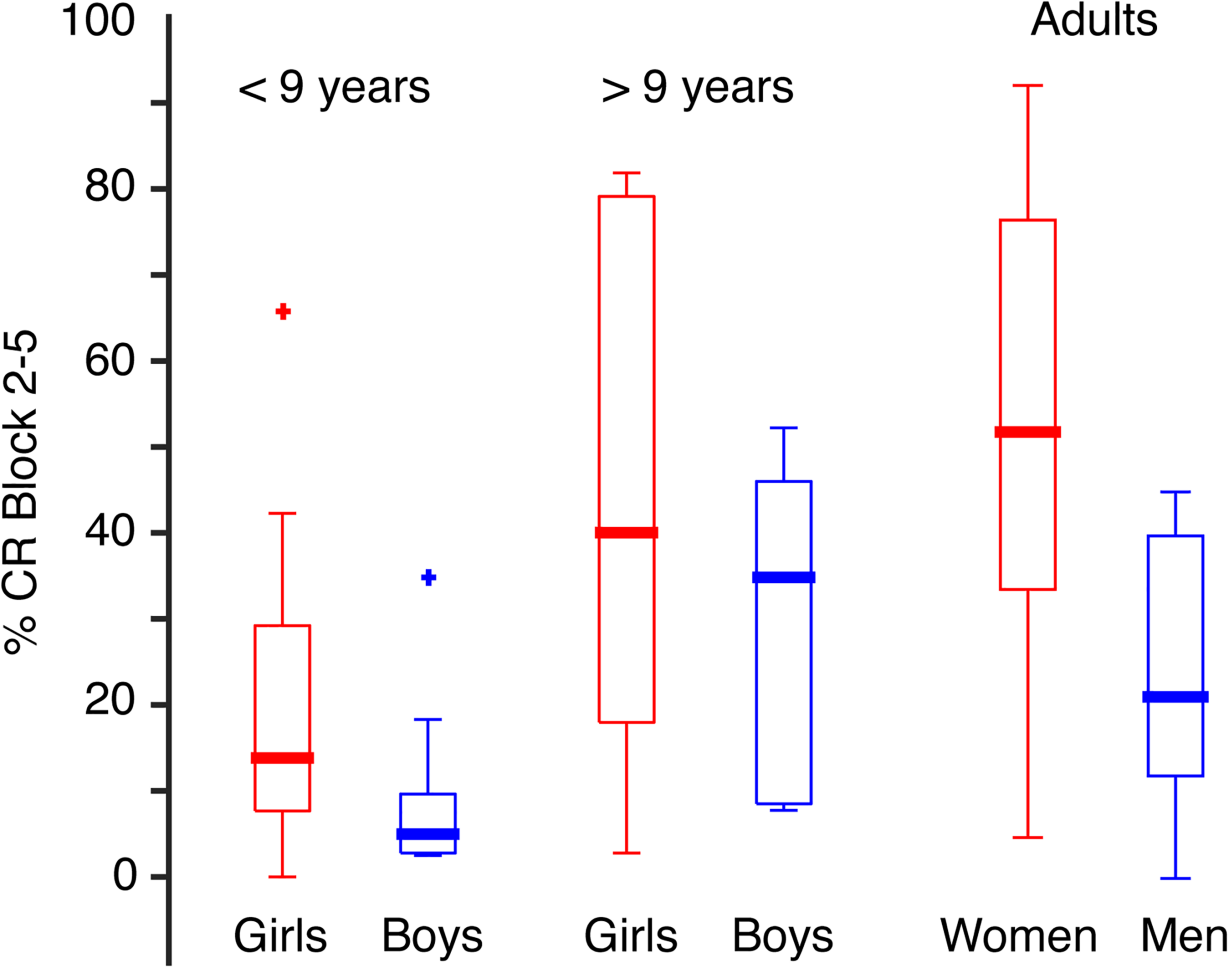
CR percentages in blocks 2–5 (mean ± SEM) in blocks 2–5 in younger girls (n 9) and boys (n 11) younger than 9 years old; older girls (n 13) and boys (n 9) 9 years old or older; women (n 18) and men (n 8). The females in each age group reached a higher average CR percentage than the males. The older children’s level of performance is near the adults’.

The females produced more CRs than the males (Fig 4). On average, the girls produced 16.6 percentage points more CRs than the boys in blocks 2–5 (t_40_ = 2.243, p = 0.031, 95% CI[1.6, 31.5]), and 15.5 percentage points more CRs than the boys in CS alone trials (t_40_ = 2.031, p = 0.049, 95% CI[7.8, 31.0]). The women produced 26 percentage points more CRs than the men in block 1 (t_2.536_ = 2.502, p = 0.020, 95% CI[4.3, 46.1]), and 29 percentage points more CRs than the men in blocks 2–5 (t_21.403_ = 3.302, p = 0.003, 95% CI[10.8, 47.5]). Girls 9 years (median age) or older reached a similar level of CRs in blocks 2–5 as the women, and boys 9 years or older performed similar to the men (Fig 5). The CR production change from block 1 to blocks 2–5 did not differ significantly between the females and males, neither among children nor among adults.

A repeated measures ANOVA, with the test session divided into the first five blocks and age split by median age (children = 9 years, adults = 28 years), demonstrates an effect of age (F(1,38) = 10.484, p = 0.003, η^2^ = 0.216), and a marginal effect of sex (F(1,38) = 3.596, p = 0.066, η^2^ = 0.086) on CR percentage among the children, without any significant interaction effect of age and sex (F(1, 38) = 0.051, p = 0.822). Similarly, there are a between-subjects effect of sex on the CR percentages among the adults (F(1, 24) = 6.730, p = 0.016, η^2^ = 0.219). The influence of the age and sex of the children is also evident in a standard linear regression model with age and sex as predictors of average CR percentages (Table 2). Together, age and sex account for 29.5% of the variance in the CR percentage in blocks 2–5 (F(2, 39) = 8.144, p = 0.001), and 29.3% of the variance in the CR percentage in CS alone trials (F(2, 39) = 8.074, p = 0.001). In block 1 there is only an age effect, which explains 10,8% (F(1, 40) = 4.836, p = 0.034) of the variance. Among the adults, there are some evidence of an effect of sex both in block 1 and in blocks 2–5, but not in CS alone trials. The regression model accounts for 14,5% (ß = -0.381, F(1, 24) = 4.075, p = 0.055) of the variance in CR percentages in block 1 and 23.8% (ß = -0.488, F(1, 24) = 7.492, p = 0.011) of the variance in CR percentages in blocks 2–5.

**Table 2:** Coefficients in standard linear regression models with centralized age in months and sex as predictors of the children’s (n = 42) CR percentages.

**Paired trials in block 1**	**Standardized coefficient (ß)**	**p-value**
Age	0.328	0.034
**Paired trials in blocks 2–5**	**Standardized coefficient (ß)**	**p-value**
Sex	-0.264	0.060
Age	0.433	0.003
**CS alone trials blocks 4–10**	**Standardized coefficient (ß)**	**p-value**
Sex	-0.223	0.096
Age	0.452	0.002

### CR timing

Among the children there was no change in the CR onset latency, or in the CR onset variability within the first five blocks (Table 3). By contrast, among the adults the CR onset latency changed during training (F(2.147, 32.203) = 4.555, p = 0.016, η^2^ = 0.233). Post-hoc pairwise comparisons with Bonferroni adjustment show that onset latency increased between block 1 and block 2 (p = 0.022), and between block 1 and block 3 (p = 0.026) but not between the later blocks. There was also an onset latency increase of 38 ms between block 1 and blocks 2–5 combined (t_17_ = 2.173, p = 0.044, 95% CI[1,74]), while there was no increase between blocks 2–5 and CS alone trials (Table 4). Statistically significant CR latency differences between children and adults are not found in any part of the session.

**Table 3:** Average CR onset latency mean (SD) and CR onset variability/mean SD (SD) in ms after CS onset in paired trials with CS-US ISI of 485 ms.

		Children			Adults		
		All	Females	Males	All	Females	Males
**Block 1**	CR onset latency	**323 (91)**	344 (94)	299 (87)	**272 (75)**	277 (74)	253 (87)
	CR onset mean SD	**99 (79)**	77 (55)	153 (96)	**77 (28)**	83 (22)	53 (41)
**Block 2**	CR onset latency	**340 (79)**	340 (74)	341 (89)	**298 (70)**	277 (67)	357 (41)
	CR onset mean SD	**83 (55)**	87 (60)	75 (45)	**68 (35)**	71 (37)	58 (32)
**Block 3**	CR onset latency	**330 (82)**	326 (75)	337 (96)	**321 (53)**	305 (48)	354 (50)
	CR onset mean SD	**67 (40)**	74 (41)	54 (36)	**65 (33)**	69 (23)	57 (50)
**Block 4**	CR onset latency	**334 (86)**	365 (38)	305 (108)	**321(59)**	313 (55)	345 (70)
	CR onset mean SD	**72 (33)**	73 (37)	72 (28)	**64 (34)**	63 (38)	72 (17)
**Block 5**	CR onset latency	**345 (63)**	356 (60)	331 (66)	**301 (59)**	299 (46)	307 (92)
	CR onset mean SD	**78 (47)**	79 (42)	76 (59)	**64 (32)**	71 (29)	40 (34)

**Table 4:** Average CR onset and peak latency mean and variability/mean SD (SD) in ms after CS onset in paired and CS alone trials.

	Onset paired trials (blocks 2–5)		Onset CS alone trials		Peak CS alone trials	
	Latency	Mean SD	Latency	Mean SD	Latency	Mean SD
**Children**	**331 (69)**	**89 (35)**	**333 (70)**	**78 (34)**	**545 (128)**	**129 (69)**
Females	340 (51)	89 (25)	327 (60)	79 (36)	500 (93)	125 (74)
Males	321 (84)	88 (45)	339 (82)	76 (33)	602 (146)	134 (64)
**Adults**	**315 (45)**	**75 (25)**	**311 (59)**	**86 (33)**	**523 (100)**	**117 (73)**
Females	300 (34)	78 (27)	301 (55)	82 (31)	512 (109)	134 (77)
Males	348 (51)	69 (20)	342 (66)	97 (38)	558 (61)	69 (22)

#### Effects of age and sex on the CR timing

**Fig 6:**
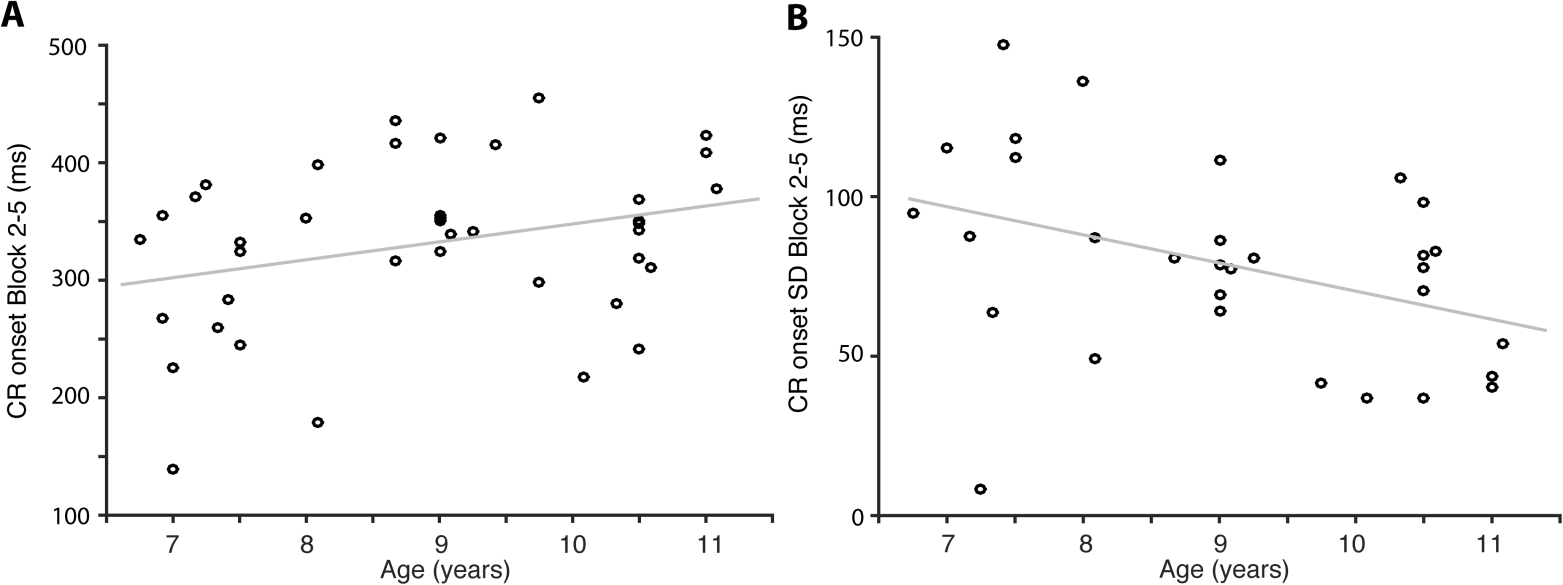
The older the children, the larger the delay and the smaller the variability of their CR onsets. **(**A) Scatterplot illustrating the mean onset latency in blocks 2–5 as a function of age. (B) Scatterplot illustrating the mean SD of the onset latency in blocks 2–5 as a function of age.

Older children and adults produced CRs that were more closely-timed to the US than younger children. Among the children, age correlates positively with CR onset latency (r = 0.347, p = 0.028, n = 40), and negatively with the variability of the CR onset latency (r = -0.398, p = 0.020, n = 34), in blocks 2–5 only (Fig 6). Standard linear regression analyses, while not repeated measures ANOVA on the first five blocks, show effects of age on the CR onset latency (ß = 0.347, R^2^ = 0.121, F(1, 38) = 5.216, p = 0.028), and on the CR onset variability (ß = -0.398, R^2^ = 0.159, F(1, 32) = 6.038, p = 0.020), in blocks 2–5. No correlations between age and any CR latency measure show among the adults.

Standard linear regression analysis, while not repeated measures ANOVA on the first five blocks, shows an effect of sex on the CR onset latencies in blocks 2–5 (ß = 0.498, R^2^ = 0.248, F(1, 24) = 7.923, p = 0.010), where the adult men produced CRs with later onsets than the women. There is also some effect of sex during CS alone trials on the CR peak latency among the children (ß = 0.400, R^2^ = 0.160, F(1, 36) = 6.873, p = 0.013), with later peaks among boys than girls, and on the CR peak latency variability among the adults (ß = -0.405, R^2^ = 0.164, F(1, 21) = 4.128, p = 0.055), with less variability among men than women.

### UR production and timing

The US consistently elicited URs in both children and adults. While the UR latency was not affected by training, it was affected by the age of the subjects. Specifically, the children’s URs started on average 15 ms later (t_52.07_, p = 0.000, 95% CI[10, 18]), and the children’s UR peaks were on average 28 ms later (t_64.228_, p = 0.000, 95% CI[21, 37]), than the adults’ in blocks 2–5 (Table 5). Moreover, among the children, age correlates negatively with the UR onset (r = -0.303, p = 0.051, n = 42; ß = -0.303, R^2^ = 0.092, F(1, 40) = 4.039, p = 0.051), and peak (r = -0.330, p = 0.033, n = 42; ß = -0.330, R^2^ = 0.109, F(1, 40) = 4.878, p = 0.033). No effect of sex on the UR latency is found among the children. No correlations between age and UR onset or peak latencies, or effects of age and sex on the UR latencies are found among the adults.

**Table 5:** Average UR onset latency mean (SD) and UR peak latency mean (SD) in ms after CS onset in paired trials with CS-US ISI of 485 ms.

		Children			Adults		
		All	Females	Males	All	Females	Males
**Block 1**	UR onset latency	**542 (11)**	542 (13)	543 (8)	**527 (13)**	528 (16)	526 (5)
	UR peak latency	**596 (25)**	591 (27)	601 (22)	**568 (23)**	567 (27)	570 (12)
**Block 2**	UR onset latency	**539 (10)**	539 (10)	538 (9)	**525 (8)**	525 (10)	526 (5)
	UR peak latency	**592 (22)**	589 (26)	596 (17)	**563 (14)**	561 (15)	567 (12)
**Block 3**	UR onset latency	**540 (11)**	541 (12)	539 (8)	**528 (10)**	529 (12)	526 (5)
	UR peak latency	**594 (21)**	591 (26)	596 (15)	**567 (16)**	567 (18)	569 (13)
**Block 4**	UR onset latency	**542 (10)**	545 (9)	539 (10)	**527 (8)**	526 (10)	529 (2)
	UR peak latency	**595 (17)**	595 (19)	594 (15)	**567 (14)**	563 (12)	577 (12)
**Block 5**	UR onset latency	**540 (10)**	541 (12)	538 (6)	**526 (10)**	527 (12)	523 (4)
	UR peak latency	**597 (19)**	595 (21)	599 (16)	**565 (14)**	563 (17)	569 (6)

## Discussion

This study demonstrates that as children get older their performance in eyeblink conditioning improves. Older children produced more CRs with better and less variable timing, and they also had earlier UR onsets and peaks. Adults showed earlier UR onsets and peaks than the children. Consistent with earlier results [39], adults reached higher rates of CRs than the children. Our school aged children never reached the same level as the adults, as infants earlier have been observed to do [44]. We did not find any effects of age among the adult participants in our study. Yet, since the majority of the adults in this study were 20–30 years old, this is in line with results of other studies on adults that have reported a drop in performance in eyeblink conditioning only above 60 years of age [41–43]. This declining performance in older adults has been attributed to age related degeneration of the cerebellum [47,48].

In addition to the age effects, our study shows that females produced more CRs than males both among the children and adults. Sex differences are present in a variety of sensory and motor tasks [49–52]. Animal studies show that adult females outperform age-matched males on a number of different learning tasks including classical conditioning [53]. Sexual dimorphism of brain development, triggered by sex hormones may potentially contribute to these differences [54–56], although other factors, including genetic, social, and environmental factors, could also influence learning and consequently contribute to our results [53,55,57]. In humans, cerebellar white and gray matter develop at different rates in girls and boys during childhood up to pre-puberty [58]. Developmental processes, whichever their causes, could potentially explain the fact that the older (9 years or older) girls in our sample performed similar to the women and the older boys performed similar to the men, whereas the younger (below 9 years old) girls and boys produced fewer CRs. However, even though the females showed greater CR production, the increase during the session compared to the first block was not significantly greater among any of the sexes. Above this, the adult men had better timed CRs than the women during later parts of the acquisition. Post training, the boys showed later CR peaks than the girls, and the men showed less CR peak variability than the women. Sensitivity to the UR might be one factor that has an effect on the acquisition, and that perhaps differs between the sexes.

### Limitations

It is legitimate to ask if the participants were properly conditioned. After training the children produced CRs on ~30% of the trials and the adults reached ~50% CRs. While this is low, these levels are above chance and they are similar to those reported in other studies on humans [40,41,59–61]. A longer training period would probably have resulted in more CRs. However, to sit still for long sessions of eyeblink conditioning is generally hard, especially for young children. Another option would be to split the training into several shorter sessions, which has been observed to result in more CRs, for adults and for infants [44]. On the other hand, in this and other studies, much of the learning occurs in the first block [62]. Extra training might have caused the age and sex effects to level out. Indeed, male rats, despite their initial disadvantage, perform on par with females after a few days of training [53]. Some caution about the sex effects among the adults is also warranted since we only tested eight adult men.

The learning curves and percentage of CRs reached by different participants were highly variable. While some individuals produced close to 100% CRs, others barely produced any CRs at all. Similarly, some individuals showed incremental numbers of CRs, while others had flat, or even negative learning curves. In other words, the relatively smooth learning curves, in this and other studies, is often not representative of the individuals in different groups.

The background sound of the movie, although soft, may have affected individuals differently, and could easily have been omitted to better control the test situation. Though, perhaps not without any impact on the concentration or motivation for some of the participants. A disadvantage in this study is that not all children were trained with identical protocols. However, despite protocol variations we did not find any statistical differences in percentage of CRs in either paired or CS alone trials between children trained with different protocols. We therefore considered grouping of the children justified although we cannot rule out potential impact on the results.

## Conclusions

Our results demonstrate that performance in eyeblink conditioning improves during development in childhood. This suggests that it may be possible to use performance in eyeblink conditioning as a measure of cerebellar maturity, or at least brain maturity. Moreover, our results indicate that sex is an important variable when it comes to eyeblink conditioning in humans. If our goal is to use eyeblink conditioning to explore cerebellar dysfunction in at-risk groups such as ADHD, ASD, FAS and SLI, disorders that are often more prevalent in males [63–65], it is important to consider the age and sex effects demonstrated here. Above that, we must be careful not to misinterpret immaturity as a disorder.

## Supporting information

S1 Dataset The raw data behind the variables that forms the basis for the conclusions drawn in this article. (PDF)
