# Supplementary material for: Performance in eyeblink conditioning is age and sex dependent
Source: PLoS One. 2017 May 18;12(5):e0177849. doi: 10.1371/journal.pone.0177849 (PMC5436819; doi:10.1371/journal.pone.0177849)
Supplement: S1 Dataset — (PDF) [file pone.0177849.s001.pdf]

Age	Sex	validity	trial_type	CR	CR_onset	CR_peakamp	CR_peaktime	Tonestart in trial_ms
124	F	valid	us only	no CR	0	0	0	501
124	F	invalid	us only	0	0	0	0	501
124	F	valid	cs only	CR	998	1.018	1210	501
124	F	valid	cs only	no CR	0	0	0	501
124	F	valid	paired	CR	566	0.991	711	501
124	F	invalid	paired	0	0	0	0	501
124	F	invalid	paired	0	0	0	0	501
124	F	invalid	paired	0	0	0	0	501
124	F	valid	paired	no CR	0	0	0	501
124	F	valid	paired	no CR	0	0	0	501
124	F	valid	paired	no CR	0	0	0	501
124	F	invalid	paired	0	0	0	0	501
124	F	valid	paired	no CR	0	0	0	501
124	F	invalid	paired	0	0	0	0	501
124	F	valid	paired	no CR	0	0	0	501
124	F	valid	paired	no CR	0	0	0	501
124	F	valid	paired	CR	807	0.395	942	501
124	F	invalid	paired	0	0	0	0	501
124	F	valid	paired	CR	750	0.029	779	501
124	F	valid	paired	CR	758	0.089	911	501
124	F	valid	paired	no CR	0	0	0	501
124	F	valid	paired	no CR	0	0	0	501
124	F	valid	paired	CR	609	0.541	759	501
124	F	invalid	paired	0	0	0	0	501
124	F	valid	paired	no CR	0	0	0	501
124	F	valid	paired	no CR	0	0	0	501
124	F	invalid	paired	0	0	0	0	501
124	F	valid	paired	no CR	0	0	0	501
124	F	invalid	paired	0	0	0	0	501
124	F	valid	paired	no CR	0	0	0	501
124	F	valid	paired	no CR	0	0	0	501
124	F	valid	paired	CR	917	0.433	1013	501
124	F	valid	paired	no CR	0	0	0	501
124	F	valid	paired	no CR	0	0	0	501
124	F	invalid	paired	0	0	0	0	501
124	F	invalid	paired	0	0	0	0	501
124	F	invalid	paired	0	0	0	0	501
124	F	valid	paired	no CR	0	0	0	501
124	F	invalid	paired	0	0	0	0	501
124	F	valid	cs only	CR	767	0.853	1152	501
124	F	invalid	paired	0	0	0	0	501
124	F	valid	paired	no CR	0	0	0	501
124	F	valid	paired	no CR	0	0	0	501
124	F	invalid	paired	0	0	0	0	501
124	F	valid	paired	CR	555	0.556	649	501
124	F	valid	paired	no CR	0	0	0	501
124	F	valid	paired	no CR	0	0	0	501
124	F	valid	paired	CR	864	0.608	1034	501
124	F	valid	paired	CR	542	0.593	703	501

124	F	valid	cs only	no CR	0		0	0	501
124	F	valid	paired	no CR	0		0	0	501
124	F	valid	paired	CR	651	0.454		769	501
124	F	valid	cs only	CR	633	0.613		795	501
124	F	invalid	cs only	0	0		0	0	501
124	F	valid	paired	CR	766	0.055		1024	501
124	F	invalid	paired	0	0		0	0	501
124	F	valid	paired	no CR	0		0	0	501
124	F	valid	paired	no CR	0		0	0	501
124	F	valid	paired	no CR	0		0	0	501
124	F	valid	paired	no CR	0		0	0	501
124	F	valid	paired	CR	843	0.016		962	501
124	F	valid	cs only	no CR	0		0	0	501
124	F	valid	cs only	no CR	0		0	0	501
124	F	valid	cs only	CR	1196	0.162		1428	501
124	F	valid	cs only	CR	796	0.402		883	501
124	F	valid	cs only	CR	958	0.056		1049	501
124	F	valid	cs only	CR	751	0.473		884	501
124	F	valid	cs only	no CR	0		0	0	501
124	F	valid	cs only	CR	884	0.669		1311	501
124	F	valid	us only	no CR	0		0	0	501
126	F	valid	us only	no CR	0		0	0	501
126	F	valid	us only	no CR	0		0	0	501
126	F	valid	cs only	CR	941	0.982		1217	501
126	F	valid	cs only	no CR	0		0	0	501
126	F	valid	paired	no CR	0		0	0	501
126	F	valid	paired	CR	591	0.217		665	501
126	F	invalid	paired	0	0		0	0	501
126	F	invalid	paired	0	0		0	0	501
126	F	valid	paired	no CR	0		0	0	501
126	F	valid	paired	no CR	0		0	0	501
126	F	valid	paired	CR	932	0.324		1028	501
126	F	valid	paired	CR	586	1.225		968	501
126	F	valid	paired	CR	770	0.778		1002	501
126	F	valid	paired	no CR	0		0	0	501
126	F	valid	paired	no CR	0		0	0	501
126	F	valid	paired	no CR	0		0	0	501
126	F	valid	paired	no CR	0		0	0	501
126	F	valid	paired	CR	864	0.322		1020	501
126	F	valid	paired	CR	890	0.267		1022	501
126	F	valid	paired	CR	908	0.562		1040	501
126	F	valid	paired	no CR	0		0	0	501
126	F	valid	paired	CR	941	0.238		1020	501
126	F	valid	paired	CR	890	0.548		1028	501
126	F	valid	paired	no CR	0		0	0	501
126	F	valid	paired	CR	840	0.504		1032	501
126	F	valid	paired	CR	860	0.543		1007	501
126	F	valid	paired	no CR	0		0	0	501
126	F	valid	paired	CR	704	1.01		971	501
126	F	valid	paired	CR	758	0.485		911	501

126	F	valid	paired	CR	784	0.618		906	501
126	F	valid	paired	CR	966	0.265		1030	501
126	F	valid	paired	CR	933	0.557		1017	501
126	F	valid	paired	CR	915	0.684		1031	501
126	F	valid	paired	CR	757	0.503		885	501
126	F	valid	paired	no CR	0		0	0	501
126	F	valid	paired	CR	930	0.061		1024	501
126	F	valid	paired	CR	879	0.631		956	501
126	F	valid	paired	CR	941	0.768		1043	501
126	F	valid	paired	CR	827		1	932	501
126	F	valid	cs only	CR	915	0.963		981	501
126	F	valid	paired	CR	873	0.849		1020	501
126	F	valid	paired	CR	804	1.188		1009	501
126	F	valid	paired	CR	907	1.331		981	501
126	F	valid	paired	CR	603	0.911		896	501
126	F	valid	paired	CR	629	0.874		921	501
126	F	valid	paired	CR	845	0.502		987	501
126	F	valid	paired	CR	924	0.487		1008	501
126	F	valid	paired	CR	782	0.546		936	501
126	F	valid	paired	CR	909	1.021		1031	501
126	F	valid	cs only	CR	828	1.569		1025	501
126	F	valid	paired	CR	796	1.482		1034	501
126	F	valid	paired	CR	813	0.543		952	501
126	F	valid	cs only	CR	743	1.031		975	501
126	F	valid	cs only	CR	802	0.511		915	501
126	F	valid	paired	CR	736	0.619		908	501
126	F	valid	paired	CR	781	1.34		1009	501
126	F	valid	paired	no CR	0		0	0	501
126	F	valid	paired	CR	829	0.703		998	501
126	F	valid	paired	no CR	0		0	0	501
126	F	valid	paired	CR	807	0.638		901	501
126	F	valid	paired	no CR	0		0	0	501
126	F	valid	cs only	CR	855	0.652		1039	501
126	F	valid	cs only	CR	1010	0.335		1199	501
126	F	valid	cs only	CR	851	0.603		1007	501
126	F	valid	cs only	CR	956	0.542		1043	501
126	F	valid	cs only	CR	894	0.649		990	501
126	F	valid	cs only	CR	819	0.553		954	501
126	F	valid	cs only	no CR	0		0	0	501
126	F	valid	cs only	CR	909	0.73		992	501
126	F	valid	us only	no CR	0		0	0	501
126	M	valid	us only	no CR	0		0	0	501
126	M	valid	us only	no CR	0		0	0	501
126	M	valid	cs only	CR	926	1.842		1051	501
126	M	valid	cs only	no CR	0		0	0	501
126	M	valid	paired	no CR	0		0	0	501
126	M	valid	paired	no CR	0		0	0	501
126	M	valid	paired	no CR	0		0	0	501
126	M	valid	paired	CR	808	0.508		911	501
126	M	valid	paired	no CR	0		0	0	501

126	M	valid	paired	no CR	0		0	0	501
126	M	valid	paired	no CR	0		0	0	501
126	M	valid	paired	CR	817	0.517		910	501
126	M	valid	paired	no CR	0		0	0	501
126	M	valid	paired	no CR	0		0	0	501
126	M	valid	paired	no CR	0		0	0	501
126	M	valid	paired	no CR	0		0	0	501
126	M	valid	paired	no CR	0		0	0	501
126	M	valid	paired	no CR	0		0	0	501
126	M	valid	paired	no CR	0		0	0	501
126	M	valid	paired	no CR	0		0	0	501
126	M	valid	paired	CR	752	0.137		822	501
126	M	valid	paired	CR	890	0.144		960	501
126	M	valid	paired	no CR	0		0	0	501
126	M	valid	paired	no CR	0		0	0	501
126	M	valid	paired	no CR	0		0	0	501
126	M	valid	paired	no CR	0		0	0	501
126	M	valid	paired	CR	560	0.175		661	501
126	M	valid	paired	no CR	0		0	0	501
126	M	valid	paired	CR	727	0.602		851	501
126	M	valid	paired	no CR	0		0	0	501
126	M	valid	paired	no CR	0		0	0	501
126	M	valid	paired	no CR	0		0	0	501
126	M	valid	paired	no CR	0		0	0	501
126	M	valid	paired	no CR	0		0	0	501
126	M	valid	paired	no CR	0		0	0	501
126	M	valid	paired	no CR	0		0	0	501
126	M	valid	paired	no CR	0		0	0	501
126	M	valid	paired	no CR	0		0	0	501
126	M	valid	paired	no CR	0		0	0	501
126	M	valid	cs only	CR	617	0.307		1106	501
126	M	valid	paired	no CR	0		0	0	501
126	M	valid	paired	CR	527	0.8		628	501
126	M	valid	paired	CR	545	0.692		797	501
126	M	valid	paired	no CR	0		0	0	501
126	M	valid	paired	no CR	0		0	0	501
126	M	valid	paired	no CR	0		0	0	501
126	M	valid	paired	CR	954	0.049		1017	501
126	M	valid	paired	no CR	0		0	0	501
126	M	valid	paired	no CR	0		0	0	501
126	M	valid	cs only	CR	806	0.484		1339	501
126	M	valid	paired	no CR	0		0	0	501
126	M	valid	paired	no CR	0		0	0	501
126	M	valid	cs only	no CR	0		0	0	501
126	M	valid	cs only	no CR	0		0	0	501
126	M	valid	paired	no CR	0		0	0	501
126	M	valid	paired	CR	898	0.186		1032	501
126	M	valid	paired	CR	896	0.614		1026	501
126	M	valid	paired	no CR	0		0	0	501
126	M	invalid	paired	0	0		0	0	501

126	M	valid	paired	CR	919	0.016		1002	501
126	M	valid	paired	no CR	0		0	0	501
126	M	valid	cs only	CR	767	0.71		905	501
126	M	valid	cs only	CR	1055	0.218		1236	501
126	M	valid	cs only	no CR	0		0	0	501
126	M	valid	cs only	CR	891	0.613		1075	501
126	M	valid	cs only	CR	522	0.225		911	501
126	M	valid	cs only	no CR	0		0	0	501
126	M	valid	cs only	CR	1140	0.291		1495	501
126	M	valid	cs only	CR	1166	0.122		1275	501
126	M	valid	us only	no CR	0		0	0	501
132	F	valid	us only	no CR	0		0	0	501
132	F	invalid	us only	0	0		0	0	501
132	F	invalid	cs only	0	0		0	0	501
132	F	valid	cs only	CR	1190	0.032		1377	501
132	F	valid	paired	no CR	0		0	0	501
132	F	valid	paired	CR	975	0.212		1022	501
132	F	valid	paired	no CR	0		0	0	501
132	F	valid	paired	no CR	0		0	0	501
132	F	valid	paired	no CR	0		0	0	501
132	F	valid	paired	no CR	0		0	0	501
132	F	valid	paired	no CR	0		0	0	501
132	F	valid	paired	no CR	0		0	0	501
132	F	valid	paired	no CR	0		0	0	501
132	F	valid	paired	no CR	0		0	0	501
132	F	valid	paired	no CR	0		0	0	501
132	F	invalid	paired	0	0		0	0	501
132	F	valid	paired	no CR	0		0	0	501
132	F	valid	paired	no CR	0		0	0	501
132	F	valid	paired	no CR	0		0	0	501
132	F	valid	paired	no CR	0		0	0	501
132	F	invalid	paired	0	0		0	0	501
132	F	valid	paired	no CR	0		0	0	501
132	F	valid	paired	no CR	0		0	0	501
132	F	valid	paired	no CR	0		0	0	501
132	F	valid	paired	no CR	0		0	0	501
132	F	valid	paired	no CR	0		0	0	501
132	F	valid	paired	no CR	0		0	0	501
132	F	valid	paired	no CR	0		0	0	501
132	F	valid	paired	no CR	0		0	0	501
132	F	valid	paired	no CR	0		0	0	501
132	F	invalid	paired	0	0		0	0	501
132	F	valid	paired	CR	930	0.57		1028	501
132	F	valid	paired	no CR	0		0	0	501
132	F	valid	paired	no CR	0		0	0	501
132	F	valid	paired	no CR	0		0	0	501
132	F	valid	paired	CR	580	0.769		681	501
132	F	valid	paired	no CR	0		0	0	501
132	F	valid	paired	no CR	0		0	0	501
132	F	invalid	paired	0	0		0	0	501

132	F	valid	cs only	CR	1032	0.802		1179	501
132	F	valid	paired	CR	523	0.698		595	501
132	F	valid	paired	no CR	0		0	0	501
132	F	valid	paired	no CR	0		0	0	501
132	F	valid	paired	CR	856	0.8		906	501
132	F	valid	paired	CR	956	0.305		1015	501
132	F	valid	paired	no CR	0		0	0	501
132	F	valid	paired	CR	917	0.637		1020	501
132	F	invalid	paired	0	0		0	0	501
132	F	valid	paired	CR	939	0.388		1022	501
132	F	valid	cs only	CR	1196	0.557		1260	501
132	F	valid	paired	CR	721	0.451		780	501
132	F	valid	paired	CR	894	0.959		1043	501
132	F	valid	cs only	CR	895	0.768		1002	501
132	F	valid	cs only	no CR	0		0	0	501
132	F	valid	paired	CR	886	0.651		1024	501
132	F	invalid	paired	0	0		0	0	501
132	F	valid	paired	no CR	0		0	0	501
132	F	valid	paired	no CR	0		0	0	501
132	F	valid	paired	CR	898	0.887		1030	501
132	F	valid	paired	no CR	0		0	0	501
132	F	valid	paired	no CR	0		0	0	501
132	F	valid	cs only	CR	855	0.163		1077	501
132	F	valid	cs only	CR	978	0.973		1069	501
132	F	valid	cs only	CR	741	0.544		1117	501
132	F	valid	cs only	CR	536	0.805		698	501
132	F	valid	cs only	no CR	0		0	0	501
132	F	valid	cs only	no CR	0		0	0	501
132	F	valid	cs only	CR	917	0.437		1039	501
132	F	valid	cs only	CR	820	0.434		877	501
132	F	valid	us only	no CR	0		0	0	501
117	M	valid	us only	no CR	0		0	0	501
117	M	valid	us only	no CR	0		0	0	501
117	M	valid	cs only	CR	905	0.452		969	501
117	M	valid	cs only	no CR	0		0	0	501
117	M	valid	paired	no CR	0		0	0	501
117	M	valid	paired	no CR	0		0	0	501
117	M	valid	paired	no CR	0		0	0	501
117	M	valid	paired	no CR	0		0	0	501
117	M	valid	paired	no CR	0		0	0	501
117	M	valid	paired	no CR	0		0	0	501
117	M	valid	paired	no CR	0		0	0	501
117	M	valid	paired	no CR	0		0	0	501
117	M	valid	paired	no CR	0		0	0	501
117	M	valid	paired	no CR	0		0	0	501
117	M	valid	paired	no CR	0		0	0	501
117	M	valid	paired	CR	898	0.025		1020	501
117	M	valid	paired	CR	971	0.232		1037	501
117	M	valid	paired	no CR	0		0	0	501
117	M	valid	paired	no CR	0		0	0	501

117	M	valid	paired	no CR	0		0	0	501
117	M	valid	paired	no CR	0		0	0	501
117	M	valid	paired	no CR	0		0	0	501
117	M	valid	paired	no CR	0		0	0	501
117	M	valid	paired	no CR	0		0	0	501
117	M	valid	paired	no CR	0		0	0	501
117	M	valid	paired	CR	941	0.63		1022	501
117	M	valid	paired	no CR	0		0	0	501
117	M	valid	paired	no CR	0		0	0	501
117	M	invalid	paired	0	0		0	0	501
117	M	valid	paired	no CR	0		0	0	501
117	M	valid	paired	no CR	0		0	0	501
117	M	valid	paired	no CR	0		0	0	501
117	M	valid	paired	CR	954	0.333		1024	501
117	M	valid	paired	no CR	0		0	0	501
117	M	valid	paired	no CR	0		0	0	501
117	M	valid	paired	no CR	0		0	0	501
117	M	valid	paired	no CR	0		0	0	501
117	M	valid	paired	no CR	0		0	0	501
117	M	valid	paired	no CR	0		0	0	501
117	M	valid	cs only	no CR	0		0	0	501
117	M	valid	paired	no CR	0		0	0	501
117	M	valid	paired	no CR	0		0	0	501
117	M	valid	paired	no CR	0		0	0	501
117	M	valid	paired	no CR	0		0	0	501
117	M	invalid	paired	0	0		0	0	501
117	M	valid	paired	no CR	0		0	0	501
117	M	valid	paired	no CR	0		0	0	501
117	M	valid	paired	no CR	0		0	0	501
117	M	valid	paired	no CR	0		0	0	501
117	M	valid	cs only	no CR	0		0	0	501
117	M	valid	paired	no CR	0		0	0	501
117	M	valid	paired	no CR	0		0	0	501
117	M	valid	cs only	no CR	0		0	0	501
117	M	valid	cs only	no CR	0		0	0	501
117	M	valid	paired	no CR	0		0	0	501
117	M	valid	paired	no CR	0		0	0	501
117	M	valid	paired	no CR	0		0	0	501
117	M	valid	paired	no CR	0		0	0	501
117	M	invalid	paired	0	0		0	0	501
117	M	valid	paired	no CR	0		0	0	501
117	M	valid	paired	no CR	0		0	0	501
117	M	valid	cs only	no CR	0		0	0	501
117	M	valid	cs only	CR	1192	0.203		1313	501
117	M	valid	cs only	no CR	0		0	0	501
117	M	invalid	cs only	0	0		0	0	501
117	M	valid	cs only	no CR	0		0	0	501
117	M	valid	cs only	CR	1183	0.334		1290	501
117	M	valid	cs only	CR	1130	0.042		1194	501
117	M	valid	cs only	CR	1161	0.355		1245	501

117	M	valid	us only	no CR	0		0	0	501
88	F	valid	us only	no CR	0		0	0	501
88	F	valid	us only	no CR	0		0	0	501
88	F	valid	cs only	CR	962	0.125		1186	501
88	F	valid	cs only	no CR	0		0	0	501
88	F	valid	paired	no CR	0		0	0	501
88	F	valid	paired	no CR	0		0	0	501
88	F	valid	paired	no CR	0		0	0	501
88	F	valid	paired	no CR	0		0	0	501
88	F	valid	paired	no CR	0		0	0	501
88	F	valid	paired	no CR	0		0	0	501
88	F	valid	paired	CR	689	0.817		793	501
88	F	invalid	paired	0	0		0	0	501
88	F	valid	paired	no CR	0		0	0	501
88	F	valid	paired	CR	695	0.677		759	501
88	F	valid	paired	no CR	0		0	0	501
88	F	valid	paired	CR	534	0.124		752	501
88	F	valid	paired	CR	685	0.615		766	501
88	F	valid	paired	no CR	0		0	0	501
88	F	valid	paired	CR	566	0.804		667	501
88	F	valid	paired	CR	556	1.059		652	501
88	F	valid	paired	no CR	0		0	0	501
88	F	valid	paired	no CR	0		0	0	501
88	F	valid	paired	no CR	0		0	0	501
88	F	valid	paired	CR	536	1.03		636	501
88	F	valid	paired	CR	743	0.241		1021	501
88	F	valid	paired	CR	916	0.862		1028	501
88	F	valid	paired	no CR	0		0	0	501
88	F	valid	paired	no CR	0		0	0	501
88	F	invalid	paired	0	0		0	0	501
88	F	valid	paired	no CR	0		0	0	501
88	F	valid	paired	no CR	0		0	0	501
88	F	valid	paired	no CR	0		0	0	501
88	F	valid	paired	no CR	0		0	0	501
88	F	valid	paired	no CR	0		0	0	501
88	F	valid	paired	no CR	0		0	0	501
88	F	valid	paired	no CR	0		0	0	501
88	F	valid	paired	no CR	0		0	0	501
88	F	valid	paired	no CR	0		0	0	501
88	F	valid	paired	no CR	0		0	0	501
88	F	invalid	cs only	0	0		0	0	501
88	F	valid	paired	no CR	0		0	0	501
88	F	valid	paired	CR	662	0.055		692	501
88	F	valid	paired	no CR	0		0	0	501
88	F	valid	paired	no CR	0		0	0	501
88	F	valid	paired	CR	545	0.823		579	501
88	F	valid	paired	no CR	0		0	0	501
88	F	valid	paired	no CR	0		0	0	501
88	F	valid	paired	CR	590	0.565		673	501
88	F	valid	paired	no CR	0		0	0	501

88	F	valid	cs only	no CR	0		0	0	501
88	F	valid	paired	no CR	0		0	0	501
88	F	valid	paired	no CR	0		0	0	501
88	F	valid	cs only	CR	1194	0.699		1313	501
88	F	valid	cs only	CR	716	0.805		862	501
88	F	valid	paired	no CR	0		0	0	501
88	F	valid	paired	no CR	0		0	0	501
88	F	valid	paired	CR	605	0.562		697	501
88	F	valid	paired	no CR	0		0	0	501
88	F	valid	paired	no CR	0		0	0	501
88	F	valid	paired	no CR	0		0	0	501
88	F	valid	paired	no CR	0		0	0	501
88	F	valid	cs only	CR	1051	0.79		1196	501
88	F	valid	cs only	no CR	0		0	0	501
88	F	valid	cs only	CR	1132	0.892		1337	501
88	F	valid	cs only	no CR	0		0	0	501
88	F	valid	cs only	CR	570	0.806		712	501
88	F	valid	cs only	no CR	0		0	0	501
88	F	valid	cs only	no CR	0		0	0	501
88	F	valid	cs only	CR	1156	0.85		1428	501
88	F	valid	us only	no CR	0		0	0	501
108	M	valid	us only	no CR	0		0	0	501
108	M	valid	us only	no CR	0		0	0	501
108	M	valid	cs only	no CR	0		0	0	501
108	M	valid	cs only	no CR	0		0	0	501
108	M	valid	paired	no CR	0		0	0	501
108	M	invalid	paired	0	0		0	0	501
108	M	valid	paired	no CR	0		0	0	501
108	M	valid	paired	no CR	0		0	0	501
108	M	valid	paired	no CR	0		0	0	501
108	M	valid	paired	CR	648	0.793		774	501
108	M	invalid	paired	0	0		0	0	501
108	M	valid	paired	no CR	0		0	0	501
108	M	valid	paired	no CR	0		0	0	501
108	M	valid	paired	CR	521	0.569		595	501
108	M	valid	paired	CR	836	0.414		946	501
108	M	valid	paired	no CR	0		0	0	501
108	M	valid	paired	CR	856	0.362		927	501
108	M	valid	paired	CR	962	0.21		1026	501
108	M	valid	paired	no CR	0		0	0	501
108	M	valid	paired	no CR	0		0	0	501
108	M	invalid	paired	0	0		0	0	501
108	M	valid	paired	no CR	0		0	0	501
108	M	invalid	paired	0	0		0	0	501
108	M	valid	paired	no CR	0		0	0	501
108	M	valid	paired	no CR	0		0	0	501
108	M	valid	paired	no CR	0		0	0	501
108	M	valid	paired	CR	752	0.594		939	501
108	M	valid	paired	no CR	0		0	0	501
108	M	valid	paired	CR	908	0.369		1030	501

108	M	valid	paired	no CR	0		0	0	501
108	M	valid	paired	no CR	0		0	0	501
108	M	valid	paired	no CR	0		0	0	501
108	M	valid	paired	CR	948	0.624		1021	501
108	M	valid	paired	CR	547	0.798		651	501
108	M	valid	paired	no CR	0		0	0	501
108	M	valid	paired	CR	684	0.865		945	501
108	M	valid	paired	CR	769	0.688		895	501
108	M	valid	paired	CR	831	0.692		962	501
108	M	valid	paired	CR	836	0.68		977	501
108	M	valid	cs only	no CR	0		0	0	501
108	M	valid	paired	CR	866	0.479		1024	501
108	M	valid	paired	CR	950	0.549		1028	501
108	M	valid	paired	no CR	0		0	0	501
108	M	valid	paired	CR	938	0.378		997	501
108	M	valid	paired	no CR	0		0	0	501
108	M	valid	paired	CR	966	0.076		1041	501
108	M	valid	paired	CR	828	0.696		1047	501
108	M	valid	paired	CR	556	0.731		616	501
108	M	valid	paired	CR	786	0.438		859	501
108	M	valid	cs only	CR	947	0.127		1156	501
108	M	valid	paired	CR	845	0.703		930	501
108	M	valid	paired	CR	932	0.789		1064	501
108	M	valid	cs only	no CR	0		0	0	501
108	M	valid	cs only	CR	920	0.906		1160	501
108	M	valid	paired	CR	717	0.484		796	501
108	M	valid	paired	no CR	0		0	0	501
108	M	valid	paired	CR	794	0.781		943	501
108	M	valid	paired	no CR	0		0	0	501
108	M	valid	paired	no CR	0		0	0	501
108	M	valid	paired	CR	976	0.443		1028	501
108	M	valid	paired	CR	560	0.754		692	501
108	M	valid	cs only	CR	972	0.594		1087	501
108	M	valid	cs only	no CR	0		0	0	501
108	M	valid	cs only	CR	1192	0.558		1328	501
108	M	valid	cs only	no CR	0		0	0	501
108	M	valid	cs only	no CR	0		0	0	501
108	M	invalid	cs only	0	0		0	0	501
108	M	valid	cs only	CR	882	0.793		1107	501
108	M	valid	cs only	no CR	0		0	0	501
108	M	valid	us only	no CR	0		0	0	501
117	M	valid	us only	no CR	0		0	0	501
117	M	valid	us only	no CR	0		0	0	501
117	M	valid	cs only	no CR	0		0	0	501
117	M	valid	cs only	no CR	0		0	0	501
117	M	valid	paired	no CR	0		0	0	501
117	M	valid	paired	no CR	0		0	0	501
117	M	invalid	paired	0	0		0	0	501
117	M	valid	paired	no CR	0		0	0	501
117	M	valid	paired	no CR	0		0	0	501

117	M	valid	paired	no CR	0		0	0	501
117	M	valid	paired	no CR	0		0	0	501
117	M	invalid	paired	0	0		0	0	501
117	M	invalid	paired	0	0		0	0	501
117	M	valid	paired	no CR	0		0	0	501
117	M	valid	paired	CR	543	0.619		633	501
117	M	valid	paired	no CR	0		0	0	501
117	M	valid	paired	CR	742	0.582		826	501
117	M	valid	paired	no CR	0		0	0	501
117	M	valid	paired	CR	655	0.535		752	501
117	M	valid	paired	CR	545	0.657		623	501
117	M	valid	paired	CR	926	0.018		1005	501
117	M	valid	paired	no CR	0		0	0	501
117	M	valid	paired	no CR	0		0	0	501
117	M	valid	paired	CR	709	0.804		784	501
117	M	valid	paired	CR	939	0.146		1017	501
117	M	invalid	paired	0	0		0	0	501
117	M	valid	paired	CR	707	0.054		747	501
117	M	valid	paired	CR	940	0.133		1007	501
117	M	valid	paired	CR	781	0.285		1011	501
117	M	valid	paired	no CR	0		0	0	501
117	M	valid	paired	CR	907	0.785		1025	501
117	M	invalid	paired	0	0		0	0	501
117	M	valid	paired	CR	852	0.419		994	501
117	M	valid	paired	no CR	0		0	0	501
117	M	invalid	paired	0	0		0	0	501
117	M	valid	paired	no CR	0		0	0	501
117	M	valid	paired	no CR	0		0	0	501
117	M	valid	paired	no CR	0		0	0	501
117	M	valid	paired	no CR	0		0	0	501
117	M	invalid	cs only	0	0		0	0	501
117	M	invalid	paired	0	0		0	0	501
117	M	valid	paired	CR	864	0.579		973	501
117	M	invalid	paired	0	0		0	0	501
117	M	invalid	paired	0	0		0	0	501
117	M	valid	paired	CR	890	0.404		979	501
117	M	invalid	paired	0	0		0	0	501
117	M	valid	paired	CR	863	0.943		984	501
117	M	invalid	paired	0	0		0	0	501
117	M	valid	paired	CR	870	0.126		1017	501
117	M	valid	cs only	CR	857	1.296		941	501
117	M	valid	paired	CR	798	0.699		888	501
117	M	valid	paired	no CR	0		0	0	501
117	M	valid	cs only	CR	664	0.7		917	501
117	M	valid	cs only	CR	962	0.676		1148	501
117	M	valid	paired	CR	897	0.66		1012	501
117	M	invalid	paired	0	0		0	0	501
117	M	valid	paired	no CR	0		0	0	501
117	M	invalid	paired	0	0		0	0	501
117	M	valid	paired	no CR	0		0	0	501

117	M	valid	paired	CR	534	0.728		607	501
117	M	valid	paired	no CR	0		0	0	501
117	M	valid	cs only	CR	1007	1.028		1171	501
117	M	valid	cs only	no CR	0		0	0	501
117	M	valid	cs only	CR	1055	0.833		1168	501
117	M	valid	cs only	no CR	0		0	0	501
117	M	valid	cs only	no CR	0		0	0	501
117	M	valid	cs only	no CR	0		0	0	501
117	M	valid	cs only	no CR	0		0	0	501
117	M	valid	cs only	CR	779	0.61		851	501
117	M	valid	us only	no CR	0		0	0	501
108	F	valid	us only	no CR	0		0	0	501
108	F	invalid	us only	0	0		0	0	501
108	F	valid	cs only	no CR	0		0	0	501
108	F	valid	cs only	CR	555	0.35		1404	501
108	F	invalid	paired	0	0		0	0	501
108	F	valid	paired	no CR	0		0	0	501
108	F	valid	paired	no CR	0		0	0	501
108	F	valid	paired	no CR	0		0	0	501
108	F	valid	paired	no CR	0		0	0	501
108	F	valid	paired	no CR	0		0	0	501
108	F	valid	paired	no CR	0		0	0	501
108	F	valid	paired	CR	962	0.449		1043	501
108	F	valid	paired	no CR	0		0	0	501
108	F	invalid	paired	0	0		0	0	501
108	F	valid	paired	no CR	0		0	0	501
108	F	valid	paired	no CR	0		0	0	501
108	F	valid	paired	no CR	0		0	0	501
108	F	valid	paired	CR	924	0.094		975	501
108	F	valid	paired	no CR	0		0	0	501
108	F	valid	paired	no CR	0		0	0	501
108	F	valid	paired	CR	651	0.384		731	501
108	F	invalid	paired	0	0		0	0	501
108	F	valid	paired	CR	604	0.119		1011	501
108	F	valid	paired	CR	919	0.356		980	501
108	F	valid	paired	CR	928	0.242		964	501
108	F	valid	paired	no CR	0		0	0	501
108	F	valid	paired	no CR	0		0	0	501
108	F	valid	paired	no CR	0		0	0	501
108	F	invalid	paired	no CR	0		0	0	501
108	F	valid	paired	CR	918	0.241		964	501
108	F	valid	paired	CR	956	0.293		1028	501
108	F	valid	paired	no CR	0		0	0	501
108	F	valid	paired	no CR	0		0	0	501
108	F	valid	paired	no CR	0		0	0	501
108	F	valid	paired	CR	894	0.068		971	501
108	F	valid	paired	no CR	0		0	0	501
108	F	valid	paired	CR	899	0.108		1020	501
108	F	invalid	paired	0	0		0	0	501
108	F	valid	paired	no CR	0		0	0	501

108	F	valid	cs only	CR	918	0.428		1096	501
108	F	valid	paired	no CR	0		0	0	501
108	F	valid	paired	CR	938	0.344		998	501
108	F	valid	paired	CR	954	0.206		1028	501
108	F	invalid	paired	0	0		0	0	501
108	F	valid	paired	CR	888	0.041		1060	501
108	F	valid	paired	CR	896	0.362		972	501
108	F	valid	paired	no CR	0		0	0	501
108	F	valid	paired	no CR	0		0	0	501
108	F	valid	paired	no CR	0		0	0	501
108	F	valid	cs only	no CR	0		0	0	501
108	F	valid	paired	no CR	0		0	0	501
108	F	valid	paired	CR	880	0.713		977	501
108	F	invalid	cs only	0	0		0	0	501
108	F	valid	cs only	CR	1196	0.153		1309	501
108	F	valid	paired	no CR	0		0	0	501
108	F	valid	paired	CR	909	0.12		983	501
108	F	valid	paired	CR	623	0.534		978	501
108	F	valid	paired	no CR	0		0	0	501
108	F	valid	paired	CR	907	0.672		955	501
108	F	valid	paired	no CR	0		0	0	501
108	F	valid	paired	CR	667	0.334		943	501
108	F	invalid	cs only	0	0		0	0	501
108	F	valid	cs only	CR	896	0.847		1039	501
108	F	valid	cs only	CR	836	0.708		969	501
108	F	valid	cs only	CR	1020	0.243		1092	501
108	F	valid	cs only	CR	615	0.557		1108	501
108	F	valid	cs only	CR	710	0.593		821	501
108	F	valid	cs only	no CR	0		0	0	501
108	F	invalid	cs only	0	0		0	0	501
108	F	invalid	us only	0	0		0	0	501
90	M	valid	us only	no CR	0		0	0	501
90	M	valid	us only	no CR	0		0	0	501
90	M	invalid	cs only	0	0		0	0	501
90	M	valid	cs only	no CR	0		0	0	501
90	M	valid	paired	no CR	0		0	0	501
90	M	valid	paired	no CR	0		0	0	501
90	M	valid	paired	no CR	0		0	0	501
90	M	valid	paired	no CR	0		0	0	501
90	M	valid	paired	no CR	0		0	0	501
90	M	valid	paired	CR	939	0.058		1011	501
90	M	valid	paired	no CR	0		0	0	501
90	M	valid	paired	no CR	0		0	0	501
90	M	valid	paired	no CR	0		0	0	501
90	M	valid	paired	no CR	0		0	0	501
90	M	valid	paired	no CR	0		0	0	501
90	M	valid	paired	no CR	0		0	0	501
90	M	valid	paired	no CR	0		0	0	501
90	M	valid	paired	no CR	0		0	0	501
90	M	valid	paired	CR	543	0.047		562	501

90	M	valid	paired	no CR	0		0	0	501
90	M	valid	paired	no CR	0		0	0	501
90	M	valid	paired	no CR	0		0	0	501
90	M	valid	paired	no CR	0		0	0	501
90	M	valid	paired	CR	582	1.014		692	501
90	M	valid	paired	no CR	0		0	0	501
90	M	valid	paired	no CR	0		0	0	501
90	M	valid	paired	CR	568	0.63		637	501
90	M	valid	paired	no CR	0		0	0	501
90	M	valid	paired	no CR	0		0	0	501
90	M	valid	paired	no CR	0		0	0	501
90	M	valid	paired	no CR	0		0	0	501
90	M	valid	paired	no CR	0		0	0	501
90	M	valid	paired	no CR	0		0	0	501
90	M	valid	paired	no CR	0		0	0	501
90	M	valid	paired	no CR	0		0	0	501
90	M	valid	paired	no CR	0		0	0	501
90	M	valid	paired	CR	973	0.703		1017	501
90	M	valid	paired	no CR	0		0	0	501
90	M	valid	paired	no CR	0		0	0	501
90	M	valid	cs only	CR	1043	0.692		1132	501
90	M	valid	paired	no CR	0		0	0	501
90	M	valid	paired	no CR	0		0	0	501
90	M	invalid	paired	0	0		0	0	501
90	M	valid	paired	CR	692	0.328		855	501
90	M	valid	paired	CR	664	0.028		702	501
90	M	valid	paired	no CR	0		0	0	501
90	M	valid	paired	no CR	0		0	0	501
90	M	invalid	paired	0	0		0	0	501
90	M	valid	paired	no CR	0		0	0	501
90	M	valid	cs only	CR	1026	0.718		1125	501
90	M	valid	paired	no CR	0		0	0	501
90	M	valid	paired	no CR	0		0	0	501
90	M	invalid	cs only	0	0		0	0	501
90	M	valid	cs only	CR	1101	0.696		1386	501
90	M	valid	paired	CR	930	0.563		1013	501
90	M	valid	paired	CR	549	0.441		768	501
90	M	valid	paired	no CR	0		0	0	501
90	M	valid	paired	no CR	0		0	0	501
90	M	valid	paired	no CR	0		0	0	501
90	M	valid	paired	CR	849	0.814		1012	501
90	M	valid	paired	no CR	0		0	0	501
90	M	valid	cs only	CR	833	0.589		1124	501
90	M	valid	cs only	CR	905	0.925		1049	501
90	M	valid	cs only	CR	1179	0.679		1281	501
90	M	valid	cs only	CR	956	0.309		1075	501
90	M	valid	cs only	no CR	0		0	0	501
90	M	valid	cs only	no CR	0		0	0	501
90	M	valid	cs only	CR	1011	0.574		1062	501
90	M	invalid	cs only	0	0		0	0	501

90	M	valid	us only	no CR	0		0	0	501
127	M	valid	us only	no CR	0		0	0	501
127	M	valid	us only	no CR	0		0	0	501
127	M	valid	cs only	CR	950	0.809		1008	501
127	M	invalid	cs only	0	0		0	0	501
127	M	invalid	paired	0	0		0	0	501
127	M	valid	paired	CR	608	0.771		681	501
127	M	invalid	paired	0	0		0	0	501
127	M	invalid	paired	0	0		0	0	501
127	M	valid	paired	CR	920	0.71		1019	501
127	M	valid	paired	no CR	0		0	0	501
127	M	valid	paired	CR	816	0.732		988	501
127	M	valid	paired	CR	690	0.704		776	501
127	M	valid	paired	CR	532	0.815		593	501
127	M	invalid	paired	0	0		0	0	501
127	M	valid	paired	no CR	0		0	0	501
127	M	invalid	paired	0	0		0	0	501
127	M	valid	paired	no CR	0		0	0	501
127	M	valid	paired	CR	750	0.584		828	501
127	M	valid	paired	CR	866	0.203		922	501
127	M	valid	paired	no CR	0		0	0	501
127	M	valid	paired	CR	572	0.802		642	501
127	M	valid	paired	no CR	0		0	0	501
127	M	valid	paired	CR	878	0.423		960	501
127	M	valid	paired	CR	830	0.775		1028	501
127	M	valid	paired	CR	941	0.263		1024	501
127	M	valid	paired	CR	704	0.533		996	501
127	M	valid	paired	CR	790	0.589		840	501
127	M	valid	paired	no CR	0		0	0	501
127	M	invalid	paired	0	0		0	0	501
127	M	invalid	paired	0	0		0	0	501
127	M	valid	paired	no CR	0		0	0	501
127	M	valid	paired	CR	721	0.846		911	501
127	M	invalid	paired	0	0		0	0	501
127	M	valid	paired	CR	895	0.195		1011	501
127	M	valid	paired	no CR	0		0	0	501
127	M	valid	paired	no CR	0		0	0	501
127	M	valid	paired	CR	886	0.709		994	501
127	M	invalid	paired	0	0		0	0	501
127	M	valid	paired	CR	843	0.648		972	501
127	M	invalid	cs only	0	0		0	0	501
127	M	valid	paired	no CR	0		0	0	501
127	M	valid	paired	CR	739	0.812		996	501
127	M	valid	paired	CR	830	0.01		896	501
127	M	valid	paired	CR	538	0.824		633	501
127	M	invalid	paired	0	0		0	0	501
127	M	valid	paired	CR	837	0.895		1008	501
127	M	valid	paired	CR	804	0.612		874	501
127	M	valid	paired	CR	914	0.548		1004	501
127	M	invalid	paired	0	0		0	0	501

127	M	valid	cs only	CR	960	0.017	1047	501
127	M	invalid	paired	0	0	0	0	501
127	M	valid	paired	CR	619	0.832	710	501
127	M	valid	cs only	CR	855	0.012	1025	501
127	M	invalid	cs only	0	0	0	0	501
127	M	invalid	paired	0	0	0	0	501
127	M	valid	paired	CR	815	0.751	1017	501
127	M	invalid	paired	0	0	0	0	501
127	M	valid	paired	CR	843	-0.058	930	501
127	M	valid	paired	CR	877	0.647	1015	501
127	M	invalid	paired	0	0	0	0	501
127	M	invalid	paired	0	0	0	0	501
127	M	valid	cs only	CR	646	0.561	706	501
127	M	invalid	cs only	0	0	0	0	501
127	M	valid	cs only	CR	873	0.852	1209	501
127	M	valid	cs only	CR	692	0.591	926	501
127	M	invalid	cs only	0	0	0	0	501
127	M	valid	cs only	CR	599	0.149	1059	501
127	M	valid	cs only	no CR	0	0	0	501
127	M	invalid	cs only	0	0	0	0	501
127	M	invalid	us only	0	0	0	0	501
126	F	valid	us only	no CR	0	0	0	501
126	F	valid	us only	no CR	0	0	0	501
126	F	valid	cs only	no CR	0	0	0	501
126	F	valid	cs only	no CR	0	0	0	501
126	F	valid	paired	no CR	0	0	0	501
126	F	valid	paired	no CR	0	0	0	501
126	F	valid	paired	no CR	0	0	0	501
126	F	valid	paired	no CR	0	0	0	501
126	F	valid	paired	no CR	0	0	0	501
126	F	valid	paired	CR	754	0.68	837	501
126	F	valid	paired	no CR	0	0	0	501
126	F	valid	paired	no CR	0	0	0	501
126	F	valid	paired	no CR	0	0	0	501
126	F	valid	paired	CR	946	0.218	1000	501
126	F	valid	paired	no CR	0	0	0	501
126	F	valid	paired	no CR	0	0	0	501
126	F	valid	paired	no CR	0	0	0	501
126	F	valid	paired	CR	917	1	968	501
126	F	valid	paired	no CR	0	0	0	501
126	F	valid	paired	CR	927	0.363	998	501
126	F	valid	paired	no CR	0	0	0	501
126	F	valid	paired	no CR	0	0	0	501
126	F	valid	paired	no CR	0	0	0	501
126	F	valid	paired	no CR	0	0	0	501
126	F	valid	paired	no CR	0	0	0	501
126	F	valid	paired	no CR	0	0	0	501
126	F	valid	paired	no CR	0	0	0	501
126	F	valid	paired	no CR	0	0	0	501
126	F	valid	paired	no CR	0	0	0	501

126	F	valid	paired	CR	863	0.229		945	501
126	F	valid	paired	CR	870	0.923		954	501
126	F	valid	paired	no CR	0		0	0	501
126	F	valid	paired	no CR	0		0	0	501
126	F	valid	paired	no CR	0		0	0	501
126	F	valid	paired	no CR	0		0	0	501
126	F	valid	paired	no CR	0		0	0	501
126	F	valid	paired	no CR	0		0	0	501
126	F	valid	paired	no CR	0		0	0	501
126	F	valid	paired	no CR	0		0	0	501
126	F	valid	cs only	CR	1014	0.832		1288	501
126	F	valid	paired	CR	942	0.555		1030	501
126	F	valid	paired	no CR	0		0	0	501
126	F	valid	paired	no CR	0		0	0	501
126	F	valid	paired	no CR	0		0	0	501
126	F	valid	paired	no CR	0		0	0	501
126	F	invalid	paired	0	0		0	0	501
126	F	valid	paired	no CR	0		0	0	501
126	F	valid	paired	no CR	0		0	0	501
126	F	valid	paired	no CR	0		0	0	501
126	F	valid	cs only	CR	685	1.019		1145	501
126	F	valid	paired	CR	953	1.157		1022	501
126	F	valid	paired	CR	858	1.094		931	501
126	F	valid	cs only	CR	891	0.758		968	501
126	F	valid	cs only	CR	923	1.071		1052	501
126	F	valid	paired	no CR	0		0	0	501
126	F	valid	paired	no CR	0		0	0	501
126	F	valid	paired	CR	600	0.767		665	501
126	F	valid	paired	no CR	0		0	0	501
126	F	valid	paired	CR	848	0.958		947	501
126	F	valid	paired	CR	912	0.714		943	501
126	F	valid	paired	CR	861	1.054		1016	501
126	F	valid	cs only	no CR	0		0	0	501
126	F	valid	cs only	CR	968	0.165		1006	501
126	F	valid	cs only	CR	1106	0.965		1225	501
126	F	valid	cs only	no CR	0		0	0	501
126	F	valid	cs only	CR	1181	0.722		1268	501
126	F	valid	cs only	no CR	0		0	0	501
126	F	valid	cs only	no CR	0		0	0	501
126	F	valid	cs only	no CR	0		0	0	501
126	F	valid	us only	no CR	0		0	0	501
133	F	invalid	us only	0	0		0	0	501
133	F	invalid	us only	0	0		0	0	501
133	F	valid	cs only	CR	1073	0.735		1123	501
133	F	valid	cs only	no CR	0		0	0	501
133	F	invalid	paired	0	0		0	0	501
133	F	valid	paired	CR	583	0.701		988	501
133	F	valid	paired	no CR	0		0	0	501
133	F	invalid	paired	no CR	0		0	0	501
133	F	valid	paired	no CR	0		0	0	501

133	F	valid	paired	CR	941	1.23		994	501
133	F	valid	paired	CR	917	1.175		996	501
133	F	invalid	paired	no CR	0		0	0	501
133	F	valid	paired	no CR	0		0	0	501
133	F	invalid	paired	0	0		0	0	501
133	F	valid	paired	CR	742	1.479		866	501
133	F	valid	paired	CR	945	1.382		1018	501
133	F	valid	paired	CR	854	1.254		1025	501
133	F	valid	paired	no CR	0		0	0	501
133	F	valid	paired	CR	979	0.503		1020	501
133	F	valid	paired	CR	949	1.044		1033	501
133	F	invalid	paired	0	0		0	0	501
133	F	valid	paired	CR	905	0.651		996	501
133	F	valid	paired	CR	926	1.292		980	501
133	F	invalid	paired	0	0		0	0	501
133	F	valid	paired	CR	886	0.93		962	501
133	F	valid	paired	CR	759	0.977		974	501
133	F	valid	paired	CR	844	0.998		899	501
133	F	invalid	paired	0	0		0	0	501
133	F	valid	paired	CR	882	1.121		936	501
133	F	valid	paired	CR	876	1.095		938	501
133	F	valid	paired	CR	514	1.218		587	501
133	F	valid	paired	CR	890	1.1		965	501
133	F	valid	paired	CR	833	1.119		882	501
133	F	valid	paired	CR	953	1.016		1006	501
133	F	valid	paired	CR	891	0.902		946	501
133	F	invalid	paired	0	0		0	0	501
133	F	valid	paired	CR	809	1.583		862	501
133	F	valid	paired	CR	855	1.128		997	501
133	F	valid	paired	CR	898	1.13		931	501
133	F	invalid	cs only	0	0		0	0	501
133	F	valid	paired	no CR	0		0	0	501
133	F	invalid	paired	0	0		0	0	501
133	F	valid	paired	CR	887	0.835		924	501
133	F	valid	paired	CR	877	0.802		946	501
133	F	valid	paired	no CR	0		0	0	501
133	F	valid	paired	no CR	0		0	0	501
133	F	valid	paired	CR	893	0.969		922	501
133	F	valid	paired	CR	853	0.84		881	501
133	F	valid	paired	CR	880	0.827		906	501
133	F	valid	cs only	CR	821	0.654		846	501
133	F	invalid	paired	no CR	0		0	0	501
133	F	valid	paired	CR	923	0.881		953	501
133	F	valid	cs only	CR	931	1.171		960	501
133	F	invalid	cs only	0	0		0	0	501
133	F	invalid	paired	0	0		0	0	501
133	F	valid	paired	CR	848	0.959		878	501
133	F	valid	paired	CR	750	0.702		845	501
133	F	invalid	paired	0	0		0	0	501
133	F	valid	paired	CR	985	1.209		1013	501

133	F	valid	paired	CR	892	0.855		975	501
133	F	valid	paired	no CR	0		0	0	501
133	F	valid	cs only	CR	564	1.226		886	501
133	F	valid	cs only	CR	909	1.272		953	501
133	F	valid	cs only	CR	845	1.121		895	501
133	F	valid	cs only	CR	867	1.198		949	501
133	F	valid	cs only	CR	1106	1.165		1149	501
133	F	valid	cs only	CR	925	1.099		960	501
133	F	invalid	cs only	0	0		0	0	501
133	F	valid	cs only	CR	854		1	958	501
133	F	valid	us only	no CR	0		0	0	501
126	F	invalid	us only	0	0		0	0	501
126	F	valid	us only	no CR	0		0	0	501
126	F	invalid	cs only	0	0		0	0	501
126	F	valid	cs only	CR	757	0.615		866	501
126	F	valid	paired	CR	858	0.08		892	501
126	F	valid	paired	no CR	0		0	0	501
126	F	valid	paired	no CR	0		0	0	501
126	F	valid	paired	no CR	0		0	0	501
126	F	valid	paired	no CR	0		0	0	501
126	F	valid	paired	no CR	0		0	0	501
126	F	valid	paired	no CR	0		0	0	501
126	F	valid	paired	CR	885	0.064		996	501
126	F	valid	paired	no CR	0		0	0	501
126	F	valid	paired	no CR	0		0	0	501
126	F	valid	paired	CR	905	0.288		983	501
126	F	valid	paired	no CR	0		0	0	501
126	F	valid	paired	CR	800	0.316		995	501
126	F	valid	paired	CR	885	0.336		957	501
126	F	valid	paired	CR	592	0.575		932	501
126	F	valid	paired	no CR	0		0	0	501
126	F	valid	paired	no CR	0		0	0	501
126	F	valid	paired	no CR	0		0	0	501
126	F	invalid	paired	0	0		0	0	501
126	F	valid	paired	no CR	0		0	0	501
126	F	valid	paired	no CR	0		0	0	501
126	F	invalid	paired	0	0		0	0	501
126	F	valid	paired	no CR	0		0	0	501
126	F	invalid	paired	0	0		0	0	501
126	F	valid	paired	CR	824	0.763		985	501
126	F	valid	paired	CR	773	0.796		1026	501
126	F	valid	paired	CR	904	0.911		1016	501
126	F	valid	paired	no CR	0		0	0	501
126	F	valid	paired	CR	812	0.888		983	501
126	F	valid	paired	CR	663	1.044		1007	501
126	F	valid	paired	CR	943	0.695		988	501
126	F	valid	paired	CR	911	0.561		1026	501
126	F	valid	paired	no CR	0		0	0	501
126	F	valid	paired	no CR	0		0	0	501
126	F	valid	paired	CR	785	0.839		1016	501

126	F	valid	cs only	CR	812	0.766		983	501
126	F	valid	paired	no CR	0		0	0	501
126	F	valid	paired	no CR	0		0	0	501
126	F	valid	paired	CR	663	1.005		915	501
126	F	valid	paired	no CR	0		0	0	501
126	F	valid	paired	CR	758	0.839		939	501
126	F	valid	paired	no CR	0		0	0	501
126	F	valid	paired	no CR	0		0	0	501
126	F	valid	paired	no CR	0		0	0	501
126	F	valid	paired	CR	822	0.919		998	501
126	F	valid	cs only	CR	885	0.782		1037	501
126	F	valid	paired	no CR	0		0	0	501
126	F	valid	paired	CR	532	0.14		961	501
126	F	valid	cs only	no CR	0		0	0	501
126	F	valid	cs only	CR	892	0.748		1062	501
126	F	valid	paired	no CR	0		0	0	501
126	F	valid	paired	no CR	0		0	0	501
126	F	valid	paired	CR	808	0.819		993	501
126	F	valid	paired	CR	922	0.098		1011	501
126	F	valid	paired	no CR	0		0	0	501
126	F	invalid	paired	0	0		0	0	501
126	F	valid	paired	CR	890	0.701		936	501
126	F	valid	cs only	CR	984	0.43		1042	501
126	F	valid	cs only	CR	941	0.925		992	501
126	F	valid	cs only	CR	536	0.765		1005	501
126	F	valid	cs only	CR	550	0.917		1054	501
126	F	valid	cs only	CR	902	0.807		1009	501
126	F	valid	cs only	CR	845	0.729		911	501
126	F	valid	cs only	no CR	0		0	0	501
126	F	valid	cs only	CR	846	0.78		957	501
126	F	valid	us only	no CR	0		0	0	501
90	F	valid	us only	no CR	0		0	0	501
90	F	valid	us only	CR	529	0.375		603	501
90	F	invalid	cs only	0	0		0	0	501
90	F	valid	cs only	no CR	0		0	0	501
90	F	valid	paired	no CR	0		0	0	501
90	F	invalid	paired	0	0		0	0	501
90	F	valid	paired	no CR	0		0	0	501
90	F	valid	paired	no CR	0		0	0	501
90	F	valid	paired	no CR	0		0	0	501
90	F	valid	paired	no CR	0		0	0	501
90	F	valid	paired	CR	547	0.424		584	501
90	F	valid	paired	no CR	0		0	0	501
90	F	valid	paired	no CR	0		0	0	501
90	F	valid	paired	no CR	0		0	0	501
90	F	valid	paired	no CR	0		0	0	501
90	F	invalid	paired	0	0		0	0	501
90	F	valid	paired	no CR	0		0	0	501
90	F	valid	paired	no CR	0		0	0	501
90	F	valid	paired	CR	620	0.182		669	501

90	F	valid	paired	CR	943	0.336		1037	501
90	F	valid	paired	no CR	0		0	0	501
90	F	valid	paired	no CR	0		0	0	501
90	F	valid	paired	no CR	0		0	0	501
90	F	valid	paired	no CR	0		0	0	501
90	F	valid	paired	CR	715	0.288		756	501
90	F	valid	paired	no CR	0		0	0	501
90	F	valid	paired	no CR	0		0	0	501
90	F	valid	paired	no CR	0		0	0	501
90	F	valid	paired	CR	703	0.544		821	501
90	F	valid	paired	no CR	0		0	0	501
90	F	valid	paired	no CR	0		0	0	501
90	F	valid	paired	no CR	0		0	0	501
90	F	valid	paired	CR	956	0.064		1022	501
90	F	invalid	paired	0	0		0	0	501
90	F	valid	paired	no CR	0		0	0	501
90	F	valid	paired	no CR	0		0	0	501
90	F	valid	paired	no CR	0		0	0	501
90	F	valid	paired	no CR	0		0	0	501
90	F	valid	paired	no CR	0		0	0	501
90	F	valid	cs only	no CR	0		0	0	501
90	F	valid	paired	no CR	0		0	0	501
90	F	valid	paired	no CR	0		0	0	501
90	F	valid	paired	no CR	0		0	0	501
90	F	valid	paired	no CR	0		0	0	501
90	F	valid	paired	no CR	0		0	0	501
90	F	valid	paired	no CR	0		0	0	501
90	F	valid	paired	no CR	0		0	0	501
90	F	valid	paired	no CR	0		0	0	501
90	F	valid	paired	no CR	0		0	0	501
90	F	valid	cs only	no CR	0		0	0	501
90	F	valid	paired	no CR	0		0	0	501
90	F	valid	paired	no CR	0		0	0	501
90	F	invalid	cs only	0	0		0	0	501
90	F	valid	cs only	CR	995	0.613		1071	501
90	F	invalid	paired	0	0		0	0	501
90	F	valid	paired	no CR	0		0	0	501
90	F	valid	paired	no CR	0		0	0	501
90	F	valid	paired	no CR	0		0	0	501
90	F	valid	paired	no CR	0		0	0	501
90	F	valid	paired	no CR	0		0	0	501
90	F	valid	paired	no CR	0		0	0	501
90	F	valid	cs only	CR	1070	0.662		1143	501
90	F	valid	cs only	CR	1050	0.48		1147	501
90	F	valid	cs only	CR	837	0.328		898	501
90	F	valid	cs only	CR	932	0.146		1071	501
90	F	valid	cs only	no CR	0		0	0	501
90	F	valid	cs only	CR	1176	0.38		1303	501
90	F	valid	cs only	CR	1181	0.249		1313	501
90	F	valid	cs only	no CR	0		0	0	501

90	F	valid	us only	CR	851	0.467		933	501
108	M	valid	us only	no CR	0		0	0	501
108	M	valid	us only	no CR	0		0	0	501
108	M	valid	cs only	no CR	0		0	0	501
108	M	valid	cs only	CR	1079	0.407		1144	501
108	M	valid	paired	no CR	0		0	0	501
108	M	valid	paired	no CR	0		0	0	501
108	M	valid	paired	CR	865	0.444		987	501
108	M	valid	paired	no CR	0		0	0	501
108	M	valid	paired	CR	523	0.53		624	501
108	M	valid	paired	no CR	0		0	0	501
108	M	valid	paired	CR	596	0.302		664	501
108	M	valid	paired	no CR	0		0	0	501
108	M	valid	paired	no CR	0		0	0	501
108	M	valid	paired	no CR	0		0	0	501
108	M	valid	paired	no CR	0		0	0	501
108	M	valid	paired	CR	951	0.852		1049	501
108	M	valid	paired	no CR	0		0	0	501
108	M	valid	paired	no CR	0		0	0	501
108	M	valid	paired	CR	546	0.456		627	501
108	M	valid	paired	no CR	0		0	0	501
108	M	valid	paired	no CR	0		0	0	501
108	M	valid	paired	CR	945	0.533		1032	501
108	M	valid	paired	no CR	0		0	0	501
108	M	valid	paired	no CR	0		0	0	501
108	M	valid	paired	no CR	0		0	0	501
108	M	valid	paired	no CR	0		0	0	501
108	M	valid	paired	CR	909	0.522		1024	501
108	M	valid	paired	no CR	0		0	0	501
108	M	valid	paired	CR	898	0.386		1026	501
108	M	valid	paired	no CR	0		0	0	501
108	M	valid	paired	no CR	0		0	0	501
108	M	valid	paired	no CR	0		0	0	501
108	M	valid	paired	no CR	0		0	0	501
108	M	valid	paired	no CR	0		0	0	501
108	M	valid	paired	no CR	0		0	0	501
108	M	valid	paired	no CR	0		0	0	501
108	M	valid	paired	no CR	0		0	0	501
108	M	invalid	paired	0	0		0	0	501
108	M	valid	paired	no CR	0		0	0	501
108	M	valid	cs only	CR	816	0.389		928	501
108	M	valid	paired	CR	966	0.257		1045	501
108	M	valid	paired	no CR	0		0	0	501
108	M	valid	paired	no CR	0		0	0	501
108	M	valid	paired	CR	615	0.41		735	501
108	M	valid	paired	no CR	0		0	0	501
108	M	invalid	paired	0	0		0	0	501
108	M	valid	paired	no CR	0		0	0	501
108	M	valid	paired	CR	631	0.19		1020	501
108	M	valid	paired	CR	939	0.063		1020	501

108	M	valid	cs only	no CR	0		0	0	501
108	M	valid	paired	no CR	0		0	0	501
108	M	valid	paired	no CR	0		0	0	501
108	M	valid	cs only	CR	882	0.729		1100	501
108	M	valid	cs only	CR	1035	0.514		1139	501
108	M	valid	paired	no CR	0		0	0	501
108	M	valid	paired	no CR	0		0	0	501
108	M	valid	paired	no CR	0		0	0	501
108	M	valid	paired	CR	951	0.146		1020	501
108	M	valid	paired	no CR	0		0	0	501
108	M	valid	paired	no CR	0		0	0	501
108	M	valid	paired	no CR	0		0	0	501
108	M	valid	cs only	no CR	0		0	0	501
108	M	valid	cs only	no CR	0		0	0	501
108	M	valid	cs only	CR	1086	0.145		1233	501
108	M	valid	cs only	no CR	0		0	0	501
108	M	valid	cs only	no CR	0		0	0	501
108	M	valid	cs only	CR	1077	0.222		1181	501
108	M	valid	cs only	CR	881	0.141		962	501
108	M	valid	cs only	CR	946	0.352		1031	501
108	M	valid	us only	no CR	0		0	0	501
104	M	valid	us only	no CR	0		0	0	501
104	M	valid	us only	no CR	0		0	0	501
104	M	valid	cs only	no CR	0		0	0	501
104	M	invalid	cs only	0	0		0	0	501
104	M	valid	paired	no CR	0		0	0	501
104	M	valid	paired	no CR	0		0	0	501
104	M	valid	paired	no CR	0		0	0	501
104	M	valid	paired	CR	532	0.336		604	501
104	M	valid	paired	CR	577	0.878		719	501
104	M	valid	paired	no CR	0		0	0	501
104	M	valid	paired	no CR	0		0	0	501
104	M	valid	paired	CR	936	0.398		999	501
104	M	valid	paired	no CR	0		0	0	501
104	M	valid	paired	no CR	0		0	0	501
104	M	valid	paired	no CR	0		0	0	501
104	M	valid	paired	no CR	0		0	0	501
104	M	valid	paired	no CR	0		0	0	501
104	M	invalid	paired	0	0		0	0	501
104	M	valid	paired	no CR	0		0	0	501
104	M	valid	paired	no CR	0		0	0	501
104	M	valid	paired	no CR	0		0	0	501
104	M	invalid	paired	0	0		0	0	501
104	M	valid	paired	no CR	0		0	0	501
104	M	valid	paired	no CR	0		0	0	501
104	M	valid	paired	no CR	0		0	0	501
104	M	valid	paired	no CR	0		0	0	501
104	M	valid	paired	no CR	0		0	0	501
104	M	valid	paired	no CR	0		0	0	501
104	M	valid	paired	CR	519	0.684		583	501

104	M	valid	paired	CR	939	0.039		1015	501
104	M	invalid	paired	0	0		0	0	501
104	M	valid	paired	no CR	0		0	0	501
104	M	valid	paired	no CR	0		0	0	501
104	M	valid	paired	CR	572	0.192		672	501
104	M	valid	paired	no CR	0		0	0	501
104	M	valid	paired	no CR	0		0	0	501
104	M	valid	paired	no CR	0		0	0	501
104	M	invalid	paired	0	0		0	0	501
104	M	valid	paired	no CR	0		0	0	501
104	M	valid	cs only	CR	996	0.656		1140	501
104	M	valid	paired	no CR	0		0	0	501
104	M	valid	paired	no CR	0		0	0	501
104	M	valid	paired	CR	572	0.225		660	501
104	M	valid	paired	no CR	0		0	0	501
104	M	valid	paired	no CR	0		0	0	501
104	M	invalid	paired	no CR	0		0	0	501
104	M	valid	paired	CR	597	0.515		703	501
104	M	valid	paired	no CR	0		0	0	501
104	M	valid	paired	CR	569	0.915		683	501
104	M	valid	cs only	no CR	0		0	0	501
104	M	invalid	paired	0	0		0	0	501
104	M	valid	paired	no CR	0		0	0	501
104	M	valid	cs only	CR	1060	-0.028		1288	501
104	M	valid	cs only	CR	1028	0.339		1118	501
104	M	valid	paired	no CR	0		0	0	501
104	M	valid	paired	no CR	0		0	0	501
104	M	valid	paired	no CR	0		0	0	501
104	M	valid	paired	no CR	0		0	0	501
104	M	valid	paired	CR	596	0.438		659	501
104	M	valid	paired	no CR	0		0	0	501
104	M	valid	paired	no CR	0		0	0	501
104	M	valid	cs only	CR	915	0.347		1033	501
104	M	valid	cs only	CR	842	0.354		1213	501
104	M	valid	cs only	CR	1026	0.176		1139	501
104	M	valid	cs only	CR	997	0.462		1125	501
104	M	valid	cs only	CR	1002	0.027		1117	501
104	M	valid	cs only	no CR	0		0	0	501
104	M	valid	cs only	CR	1013	0.048		1094	501
104	M	valid	cs only	CR	922	-0.003		1064	501
104	M	valid	us only	no CR	0		0	0	501
97	M	invalid	us only	0	0		0	0	501
97	M	valid	us only	CR	551	0.76		697	501
97	M	valid	cs only	CR	775	0.52		870	501
97	M	valid	cs only	no CR	0		0	0	501
97	M	valid	paired	no CR	0		0	0	501
97	M	valid	paired	no CR	0		0	0	501
97	M	valid	paired	no CR	0		0	0	501
97	M	valid	paired	no CR	0		0	0	501
97	M	invalid	paired	0	0		0	0	501

97	M	valid	paired	CR	550	0.394		632	501
97	M	valid	paired	no CR	0		0	0	501
97	M	valid	paired	no CR	0		0	0	501
97	M	invalid	paired	0	0		0	0	501
97	M	valid	paired	no CR	0		0	0	501
97	M	invalid	paired	0	0		0	0	501
97	M	valid	paired	no CR	0		0	0	501
97	M	valid	paired	no CR	0		0	0	501
97	M	valid	paired	no CR	0		0	0	501
97	M	valid	paired	no CR	0		0	0	501
97	M	valid	paired	no CR	0		0	0	501
97	M	valid	paired	no CR	0		0	0	501
97	M	valid	paired	no CR	0		0	0	501
97	M	valid	paired	CR	579	0.444		671	501
97	M	valid	paired	no CR	0		0	0	501
97	M	valid	paired	no CR	0		0	0	501
97	M	valid	paired	CR	881	0.026		913	501
97	M	valid	paired	no CR	0		0	0	501
97	M	valid	paired	no CR	0		0	0	501
97	M	valid	paired	no CR	0		0	0	501
97	M	valid	paired	no CR	0		0	0	501
97	M	valid	paired	no CR	0		0	0	501
97	M	valid	paired	no CR	0		0	0	501
97	M	valid	paired	CR	512	0.505		623	501
97	M	valid	paired	CR	532	0.274		623	501
97	M	valid	paired	CR	740	0.415		902	501
97	M	valid	paired	no CR	0		0	0	501
97	M	valid	paired	no CR	0		0	0	501
97	M	valid	paired	no CR	0		0	0	501
97	M	valid	paired	CR	851	0.059		883	501
97	M	valid	cs only	CR	617	0.553		782	501
97	M	valid	paired	no CR	0		0	0	501
97	M	valid	paired	no CR	0		0	0	501
97	M	valid	paired	no CR	0		0	0	501
97	M	valid	paired	no CR	0		0	0	501
97	M	valid	paired	no CR	0		0	0	501
97	M	valid	paired	no CR	0		0	0	501
97	M	valid	paired	no CR	0		0	0	501
97	M	valid	paired	no CR	0		0	0	501
97	M	valid	paired	no CR	0		0	0	501
97	M	valid	cs only	no CR	0		0	0	501
97	M	valid	paired	no CR	0		0	0	501
97	M	invalid	paired	0	0		0	0	501
97	M	valid	cs only	CR	1165	0.993		1441	501
97	M	valid	cs only	no CR	0		0	0	501
97	M	invalid	paired	0	0		0	0	501
97	M	valid	paired	no CR	0		0	0	501
97	M	valid	paired	no CR	0		0	0	501
97	M	valid	paired	no CR	0		0	0	501
97	M	valid	paired	CR	919	0.038		979	501

97	M	valid	paired	CR	753	0.442		914	501
97	M	valid	paired	no CR	0		0	0	501
97	M	valid	cs only	CR	1193	0.498		1284	501
97	M	invalid	cs only	no CR	0		0	0	501
97	M	invalid	cs only	0	0		0	0	501
97	M	valid	cs only	no CR	0		0	0	501
97	M	valid	cs only	no CR	0		0	0	501
97	M	valid	cs only	CR	638	0.385		894	501
97	M	valid	cs only	no CR	0		0	0	501
97	M	valid	cs only	no CR	0		0	0	501
97	M	valid	us only	no CR	0		0	0	501
121	M	valid	us only	no CR	0		0	0	501
121	M	valid	us only	no CR	0		0	0	501
121	M	valid	cs only	no CR	0		0	0	501
121	M	invalid	cs only	0	0		0	0	501
121	M	valid	paired	no CR	0		0	0	501
121	M	valid	paired	no CR	0		0	0	501
121	M	valid	paired	no CR	0		0	0	501
121	M	valid	paired	CR	667	0.108		723	501
121	M	valid	paired	no CR	0		0	0	501
121	M	valid	paired	no CR	0		0	0	501
121	M	valid	paired	no CR	0		0	0	501
121	M	valid	paired	no CR	0		0	0	501
121	M	valid	paired	no CR	0		0	0	501
121	M	invalid	paired	0	0		0	0	501
121	M	valid	paired	no CR	0		0	0	501
121	M	invalid	paired	0	0		0	0	501
121	M	valid	paired	no CR	0		0	0	501
121	M	valid	paired	no CR	0		0	0	501
121	M	valid	paired	no CR	0		0	0	501
121	M	valid	paired	no CR	0		0	0	501
121	M	valid	paired	no CR	0		0	0	501
121	M	valid	paired	no CR	0		0	0	501
121	M	valid	paired	no CR	0		0	0	501
121	M	valid	paired	no CR	0		0	0	501
121	M	valid	paired	no CR	0		0	0	501
121	M	valid	paired	no CR	0		0	0	501
121	M	valid	paired	no CR	0		0	0	501
121	M	valid	paired	no CR	0		0	0	501
121	M	valid	paired	no CR	0		0	0	501
121	M	valid	paired	no CR	0		0	0	501
121	M	valid	paired	no CR	0		0	0	501
121	M	valid	paired	no CR	0		0	0	501
121	M	valid	paired	no CR	0		0	0	501
121	M	valid	paired	CR	628	0.605		1002	501
121	M	valid	paired	no CR	0		0	0	501
121	M	valid	paired	no CR	0		0	0	501
121	M	valid	paired	no CR	0		0	0	501
121	M	valid	paired	no CR	0		0	0	501
121	M	valid	paired	no CR	0		0	0	501

121	M	valid	cs only	no CR	0		0	0	501
121	M	valid	paired	CR	833	0.495		958	501
121	M	valid	paired	no CR	0		0	0	501
121	M	valid	paired	no CR	0		0	0	501
121	M	valid	paired	CR	781	0.203		885	501
121	M	valid	paired	no CR	0		0	0	501
121	M	valid	paired	no CR	0		0	0	501
121	M	valid	paired	no CR	0		0	0	501
121	M	valid	paired	no CR	0		0	0	501
121	M	valid	paired	no CR	0		0	0	501
121	M	invalid	cs only	0	0		0	0	501
121	M	valid	paired	no CR	0		0	0	501
121	M	valid	paired	no CR	0		0	0	501
121	M	valid	cs only	CR	1182	1.455		1342	501
121	M	valid	cs only	no CR	0		0	0	501
121	M	valid	paired	no CR	0		0	0	501
121	M	valid	paired	no CR	0		0	0	501
121	M	valid	paired	no CR	0		0	0	501
121	M	valid	paired	CR	750	0.049		849	501
121	M	valid	paired	CR	715	0.317		924	501
121	M	valid	paired	no CR	0		0	0	501
121	M	valid	paired	CR	929	0.431		1023	501
121	M	invalid	cs only	0	0		0	0	501
121	M	valid	cs only	CR	1058	0.078		1390	501
121	M	valid	cs only	no CR	0		0	0	501
121	M	valid	cs only	CR	984	0.175		1088	501
121	M	valid	cs only	CR	550	0.242		1205	501
121	M	valid	cs only	no CR	0		0	0	501
121	M	valid	cs only	CR	1097	0.107		1130	501
121	M	valid	cs only	no CR	0		0	0	501
121	M	valid	us only	no CR	0		0	0	501
84	M	valid	us only	no CR	0		0	0	501
84	M	valid	us only	CR	792	0.742		899	501
84	M	valid	cs only	CR	968	0.589		1029	501
84	M	valid	cs only	no CR	0		0	0	501
84	M	valid	paired	no CR	0		0	0	501
84	M	invalid	paired	0	0		0	0	501
84	M	invalid	paired	0	0		0	0	501
84	M	valid	paired	no CR	0		0	0	501
84	M	valid	paired	no CR	0		0	0	501
84	M	valid	paired	no CR	0		0	0	501
84	M	valid	paired	no CR	0		0	0	501
84	M	invalid	paired	0	0		0	0	501
84	M	valid	paired	no CR	0		0	0	501
84	M	invalid	paired	0	0		0	0	501
84	M	valid	paired	no CR	0		0	0	501
84	M	valid	paired	no CR	0		0	0	501
84	M	valid	paired	no CR	0		0	0	501
84	M	valid	paired	CR	917	0.448		1003	501
84	M	valid	paired	CR	656	1.009		959	501

84	M	valid	paired	CR	641	0.152		1030	501
84	M	invalid	paired	0	0		0	0	501
84	M	valid	paired	CR	770	0.098		830	501
84	M	valid	paired	no CR	0		0	0	501
84	M	invalid	paired	0	0		0	0	501
84	M	valid	paired	no CR	0		0	0	501
84	M	invalid	paired	0	0		0	0	501
84	M	invalid	paired	0	0		0	0	501
84	M	invalid	paired	0	0		0	0	501
84	M	valid	paired	no CR	0		0	0	501
84	M	valid	paired	no CR	0		0	0	501
84	M	valid	paired	CR	574	0.497		646	501
84	M	invalid	paired	0	0		0	0	501
84	M	valid	paired	no CR	0		0	0	501
84	M	valid	paired	no CR	0		0	0	501
84	M	valid	paired	no CR	0		0	0	501
84	M	invalid	paired	0	0		0	0	501
84	M	valid	paired	CR	531	0.655		640	501
84	M	valid	paired	CR	608	0.869		740	501
84	M	valid	paired	no CR	0		0	0	501
84	M	valid	cs only	CR	715	0.427		808	501
84	M	valid	paired	no CR	0		0	0	501
84	M	valid	paired	no CR	0		0	0	501
84	M	valid	paired	no CR	0		0	0	501
84	M	valid	paired	no CR	0		0	0	501
84	M	valid	paired	no CR	0		0	0	501
84	M	invalid	paired	0	0		0	0	501
84	M	valid	paired	CR	777	0.648		948	501
84	M	valid	paired	no CR	0		0	0	501
84	M	valid	paired	no CR	0		0	0	501
84	M	valid	cs only	no CR	0		0	0	501
84	M	valid	paired	no CR	0		0	0	501
84	M	valid	paired	CR	890	0.421		949	501
84	M	invalid	cs only	0	0		0	0	501
84	M	valid	cs only	no CR	0		0	0	501
84	M	invalid	paired	0	0		0	0	501
84	M	valid	paired	no CR	0		0	0	501
84	M	valid	paired	CR	909	0.013		985	501
84	M	valid	paired	no CR	0		0	0	501
84	M	valid	paired	no CR	0		0	0	501
84	M	valid	paired	no CR	0		0	0	501
84	M	valid	paired	no CR	0		0	0	501
84	M	valid	cs only	CR	677	0.724		1176	501
84	M	valid	cs only	CR	755	0.623		877	501
84	M	invalid	cs only	0	0		0	0	501
84	M	valid	cs only	CR	1051	0.446		1109	501
84	M	valid	cs only	no CR	0		0	0	501
84	M	valid	cs only	no CR	0		0	0	501
84	M	valid	cs only	CR	692	0.403		1205	501
84	M	valid	cs only	CR	899	0.331		1085	501

84	M	invalid	us only	0	0		0	0	501
126	M	valid	us only	no CR	0		0	0	501
126	M	valid	us only	no CR	0		0	0	501
126	M	valid	cs only	no CR	0		0	0	501
126	M	valid	cs only	CR	1013	0.583		1096	501
126	M	valid	paired	no CR	0		0	0	501
126	M	valid	paired	no CR	0		0	0	501
126	M	valid	paired	no CR	0		0	0	501
126	M	valid	paired	CR	915	1.408		1023	501
126	M	valid	paired	CR	750	0.029		1020	501
126	M	valid	paired	CR	794	1.444		958	501
126	M	valid	paired	CR	905	0.035		1011	501
126	M	valid	paired	no CR	0		0	0	501
126	M	valid	paired	CR	760	0.854		1007	501
126	M	valid	paired	CR	764	0.154		1011	501
126	M	valid	paired	CR	926	0.053		1013	501
126	M	valid	paired	CR	627	0.11		919	501
126	M	valid	paired	CR	885	0.091		954	501
126	M	valid	paired	CR	793	0.153		940	501
126	M	valid	paired	CR	811	0.106		949	501
126	M	valid	paired	no CR	0		0	0	501
126	M	valid	paired	CR	874	0.268		942	501
126	M	valid	paired	no CR	0		0	0	501
126	M	valid	paired	CR	928	0.295		1005	501
126	M	valid	paired	CR	936	0.256		992	501
126	M	valid	paired	CR	858	0.283		1017	501
126	M	valid	paired	CR	860	0.104		934	501
126	M	valid	paired	CR	941	0.089		1026	501
126	M	valid	paired	no CR	0		0	0	501
126	M	valid	paired	CR	857	0.244		986	501
126	M	valid	paired	CR	883	0.139		1020	501
126	M	valid	paired	no CR	0		0	0	501
126	M	valid	paired	no CR	0		0	0	501
126	M	valid	paired	CR	851	0.261		1010	501
126	M	valid	paired	CR	877	0.467		953	501
126	M	valid	paired	CR	871	0.176		995	501
126	M	valid	paired	no CR	0		0	0	501
126	M	valid	paired	CR	769	0.659		963	501
126	M	valid	paired	CR	849	0.559		1022	501
126	M	valid	paired	CR	837	0.329		958	501
126	M	valid	cs only	CR	858	0.204		960	501
126	M	valid	paired	CR	934	0.149		1017	501
126	M	valid	paired	CR	930	0.297		1022	501
126	M	valid	paired	CR	824	0.565		984	501
126	M	valid	paired	CR	928	0.146		1020	501
126	M	valid	paired	CR	854	0.393		908	501
126	M	valid	paired	CR	954	0.257		1024	501
126	M	valid	paired	CR	837	0.295		1018	501
126	M	valid	paired	CR	964	0.067		1024	501
126	M	valid	paired	no CR	0		0	0	501

126	M	valid	cs only	CR	925	0.433		1025	501
126	M	valid	paired	no CR	0		0	0	501
126	M	valid	paired	CR	611	0.254		907	501
126	M	valid	cs only	CR	869	0.459		1103	501
126	M	valid	cs only	CR	934	0.137		1158	501
126	M	valid	paired	CR	943	0.122		1020	501
126	M	valid	paired	CR	815	0.369		922	501
126	M	valid	paired	CR	960	0.141		1019	501
126	M	valid	paired	CR	822	0.482		959	501
126	M	valid	paired	CR	898	0.508		1022	501
126	M	valid	paired	CR	797	0.5		952	501
126	M	valid	paired	no CR	0		0	0	501
126	M	valid	cs only	CR	947	0.049		1015	501
126	M	valid	cs only	CR	749	0.694		1172	501
126	M	valid	cs only	CR	864	0.324		975	501
126	M	valid	cs only	CR	837	0.239		967	501
126	M	valid	cs only	CR	921	0.129		1007	501
126	M	valid	cs only	CR	855	0.08		917	501
126	M	valid	cs only	CR	749	0.135		1024	501
126	M	valid	cs only	no CR	0		0	0	501
126	M	valid	us only	no CR	0		0	0	501
132	F	valid	us only	no CR	0		0	0	501
132	F	valid	us only	no CR	0		0	0	501
132	F	valid	cs only	no CR	0		0	0	501
132	F	valid	cs only	CR	1108	0.687		1154	501
132	F	valid	paired	no CR	0		0	0	501
132	F	valid	paired	no CR	0		0	0	501
132	F	valid	paired	CR	681	0.85		753	501
132	F	valid	paired	no CR	0		0	0	501
132	F	invalid	paired	0	0		0	0	501
132	F	invalid	paired	0	0		0	0	501
132	F	valid	paired	CR	890	0.775		1003	501
132	F	valid	paired	no CR	0		0	0	501
132	F	valid	paired	no CR	0		0	0	501
132	F	valid	paired	no CR	0		0	0	501
132	F	valid	paired	CR	926	0.747		1000	501
132	F	valid	paired	no CR	0		0	0	501
132	F	valid	paired	CR	969	0.625		1029	501
132	F	invalid	paired	0	0		0	0	501
132	F	valid	paired	CR	948	0.558		1026	501
132	F	valid	paired	CR	912	0.685		1023	501
132	F	invalid	paired	0	0		0	0	501
132	F	valid	paired	no CR	0		0	0	501
132	F	valid	paired	CR	821	0.587		959	501
132	F	valid	paired	no CR	0		0	0	501
132	F	valid	paired	CR	540	0.53		623	501
132	F	valid	paired	CR	920	0.203		994	501
132	F	valid	paired	no CR	0		0	0	501
132	F	valid	paired	CR	896	0.486		1054	501
132	F	valid	paired	CR	900	0.558		987	501

132	F	valid	paired	no CR	0		0	0	501
132	F	valid	paired	no CR	0		0	0	501
132	F	valid	paired	CR	895	0.706		1002	501
132	F	valid	paired	no CR	0		0	0	501
132	F	valid	paired	CR	867	0.901		958	501
132	F	invalid	paired	0	0		0	0	501
132	F	valid	paired	no CR	0		0	0	501
132	F	valid	paired	no CR	0		0	0	501
132	F	valid	paired	CR	901	0.806		1020	501
132	F	valid	paired	no CR	0		0	0	501
132	F	valid	cs only	no CR	0		0	0	501
132	F	valid	paired	no CR	0		0	0	501
132	F	valid	paired	no CR	0		0	0	501
132	F	valid	paired	no CR	0		0	0	501
132	F	valid	paired	no CR	0		0	0	501
132	F	valid	paired	CR	884	0.532		967	501
132	F	valid	paired	no CR	0		0	0	501
132	F	valid	paired	no CR	0		0	0	501
132	F	valid	paired	no CR	0		0	0	501
132	F	valid	paired	no CR	0		0	0	501
132	F	valid	cs only	CR	982	0.703		1126	501
132	F	valid	paired	no CR	0		0	0	501
132	F	invalid	paired	0	0		0	0	501
132	F	valid	cs only	CR	1007	0.114		1183	501
132	F	valid	cs only	CR	867	0.46		1002	501
132	F	valid	paired	CR	741	0.159		849	501
132	F	valid	paired	CR	919	0.099		1028	501
132	F	valid	paired	CR	895	0.458		948	501
132	F	valid	paired	CR	897	0.689		956	501
132	F	valid	paired	no CR	0		0	0	501
132	F	valid	paired	CR	911	0.707		990	501
132	F	valid	paired	CR	970	0.969		1036	501
132	F	valid	cs only	CR	935	0.158		1163	501
132	F	valid	cs only	CR	552	0.731		966	501
132	F	valid	cs only	CR	955	0.703		1088	501
132	F	valid	cs only	CR	745	0.907		977	501
132	F	valid	cs only	CR	894	0.964		1080	501
132	F	valid	cs only	no CR	0		0	0	501
132	F	valid	cs only	CR	917	0.248		1080	501
132	F	invalid	cs only	0	0		0	0	501
132	F	invalid	us only	0	0		0	0	501
126	F	valid	us only	no CR	0		0	0	501
126	F	invalid	us only	0	0		0	0	501
126	F	valid	cs only	CR	1012	0.296		1073	501
126	F	valid	cs only	no CR	0		0	0	501
126	F	valid	paired	CR	945	0.436		1049	501
126	F	valid	paired	CR	837	0.561		916	501
126	F	valid	paired	no CR	0		0	0	501
126	F	invalid	paired	0	0		0	0	501
126	F	valid	paired	CR	651	0.409		746	501

126	F	valid	paired	CR	909	0.493		1032	501
126	F	valid	paired	CR	898	0.613		1011	501
126	F	valid	paired	CR	939	0.724		1045	501
126	F	invalid	paired	0	0		0	0	501
126	F	valid	paired	CR	941	0.383		1032	501
126	F	invalid	paired	0	0		0	0	501
126	F	valid	paired	CR	868	0.431		933	501
126	F	invalid	paired	0	0		0	0	501
126	F	valid	paired	CR	588	0.437		737	501
126	F	valid	paired	CR	847	0.629		991	501
126	F	valid	paired	CR	849	0.349		951	501
126	F	valid	paired	no CR	0		0	0	501
126	F	valid	paired	no CR	0		0	0	501
126	F	invalid	paired	0	0		0	0	501
126	F	valid	paired	CR	847	0.805		964	501
126	F	valid	paired	CR	815	0.793		995	501
126	F	valid	paired	CR	879	0.622		975	501
126	F	invalid	paired	0	0		0	0	501
126	F	valid	paired	CR	945	0.632		1051	501
126	F	valid	paired	CR	755	0.754		873	501
126	F	valid	paired	CR	719	0.478		820	501
126	F	valid	paired	no CR	0		0	0	501
126	F	valid	paired	CR	898	0.493		1007	501
126	F	valid	paired	CR	548	0.4		619	501
126	F	valid	paired	CR	876	0.719		943	501
126	F	valid	paired	CR	900	0.867		977	501
126	F	valid	paired	CR	537	0.515		602	501
126	F	valid	paired	CR	627	0.464		719	501
126	F	valid	paired	CR	798	0.427		874	501
126	F	valid	paired	CR	849	0.563		956	501
126	F	valid	cs only	CR	766	0.738		896	501
126	F	valid	paired	CR	709	0.665		855	501
126	F	valid	paired	CR	857	0.671		951	501
126	F	valid	paired	CR	956	0.64		1047	501
126	F	valid	paired	CR	846	0.774		957	501
126	F	valid	paired	CR	930	0.649		1030	501
126	F	invalid	paired	0	0		0	0	501
126	F	valid	paired	CR	785	0.521		910	501
126	F	valid	paired	CR	772	0.751		984	501
126	F	invalid	paired	0	0		0	0	501
126	F	valid	cs only	CR	557	0.781		1069	501
126	F	valid	paired	CR	928	0.663		1045	501
126	F	valid	paired	CR	896	0.498		1008	501
126	F	invalid	cs only	0	0		0	0	501
126	F	valid	cs only	CR	873	0.708		1053	501
126	F	valid	paired	CR	802	0.505		875	501
126	F	valid	paired	CR	738	0.854		993	501
126	F	valid	paired	CR	861	0.625		939	501
126	F	invalid	paired	0	0		0	0	501
126	F	valid	paired	CR	949	0.017		1007	501

126	F	valid	paired	CR	843	0.591		969	501
126	F	valid	paired	CR	826	0.708		999	501
126	F	valid	cs only	CR	882	0.623		1005	501
126	F	valid	cs only	CR	852	0.544		938	501
126	F	valid	cs only	CR	886	0.654		1047	501
126	F	valid	cs only	CR	938	0.844		1030	501
126	F	valid	cs only	CR	766	0.546		851	501
126	F	valid	cs only	CR	777	0.59		890	501
126	F	valid	cs only	CR	777	0.682		1030	501
126	F	valid	cs only	CR	762	0.424		899	501
126	F	valid	us only	no CR	0		0	0	501
87	M	valid	us only	no CR	0		0	0	501
87	M	valid	us only	no CR	0		0	0	501
87	M	valid	cs only	no CR	0		0	0	501
87	M	valid	paired	no CR	0		0	0	501
87	M	valid	paired	no CR	0		0	0	501
87	M	valid	paired	no CR	0		0	0	501
87	M	valid	paired	no CR	0		0	0	501
87	M	valid	paired	no CR	0		0	0	501
87	M	valid	paired	CR	822	0.035		971	501
87	M	valid	paired	no CR	0		0	0	501
87	M	valid	paired	no CR	0		0	0	501
87	M	valid	paired	no CR	0		0	0	501
87	M	valid	paired	no CR	0		0	0	501
87	M	valid	paired	no CR	0		0	0	501
87	M	valid	paired	CR	888	0.026		902	501
87	M	valid	paired	CR	834	0.107		909	501
87	M	valid	paired	no CR	0		0	0	501
87	M	valid	paired	CR	852	0.167		911	501
87	M	valid	paired	no CR	0		0	0	501
87	M	valid	paired	no CR	0		0	0	501
87	M	invalid	paired	0	0		0	0	501
87	M	valid	paired	no CR	0		0	0	501
87	M	valid	paired	no CR	0		0	0	501
87	M	valid	paired	CR	913	0.29		991	501
87	M	valid	paired	no CR	0		0	0	501
87	M	valid	paired	CR	925	0.257		994	501
87	M	valid	paired	no CR	0		0	0	501
87	M	valid	paired	no CR	0		0	0	501
87	M	valid	paired	no CR	0		0	0	501
87	M	valid	paired	CR	932	0.04		971	501
87	M	valid	paired	no CR	0		0	0	501
87	M	valid	paired	no CR	0		0	0	501
87	M	invalid	paired	0	0		0	0	501
87	M	valid	paired	no CR	0		0	0	501
87	M	valid	paired	no CR	0		0	0	501
87	M	valid	paired	CR	809	0.094		864	501
87	M	valid	paired	no CR	0		0	0	501
87	M	valid	paired	no CR	0		0	0	501
87	M	valid	paired	no CR	0		0	0	501

87	M	valid	paired	no CR	0		0	0	501
87	M	valid	paired	no CR	0		0	0	501
87	M	valid	paired	CR	949	0.034		1020	501
87	M	invalid	paired	0	0		0	0	501
87	M	valid	paired	CR	687	0.075		1022	501
87	M	valid	paired	no CR	0		0	0	501
87	M	valid	paired	no CR	0		0	0	501
87	M	valid	paired	no CR	0		0	0	501
87	M	valid	paired	CR	915	0.024		943	501
87	M	valid	paired	no CR	0		0	0	501
87	M	valid	cs only	CR	873	0.574		1325	501
87	M	valid	paired	no CR	0		0	0	501
87	M	valid	paired	no CR	0		0	0	501
87	M	valid	paired	no CR	0		0	0	501
87	M	valid	paired	CR	823	0.575		1022	501
87	M	valid	paired	no CR	0		0	0	501
87	M	valid	paired	no CR	0		0	0	501
87	M	valid	paired	no CR	0		0	0	501
87	M	valid	paired	no CR	0		0	0	501
87	M	valid	paired	no CR	0		0	0	501
87	M	valid	cs only	CR	862	0.789		1142	501
87	M	valid	paired	CR	769	0.774		895	501
87	M	valid	paired	no CR	0		0	0	501
87	M	valid	paired	CR	802	0.484		955	501
87	M	valid	paired	CR	738	0.152		997	501
87	M	valid	paired	CR	873	0.072		1024	501
87	M	valid	paired	CR	868	0.34		1030	501
87	M	valid	paired	CR	864	0.06		911	501
87	M	valid	paired	CR	868	0.067		1024	501
87	M	valid	paired	CR	877	0.111		1015	501
87	M	valid	cs only	CR	803	0.239		1101	501
87	M	valid	cs only	CR	966	0.209		1103	501
87	M	valid	paired	CR	971	0.129		1022	501
87	M	valid	cs only	no CR	0		0	0	501
87	M	valid	cs only	no CR	0		0	0	501
87	M	valid	paired	CR	905	0.143		979	501
87	M	valid	paired	CR	805	0.157		933	501
87	M	valid	cs only	no CR	0		0	0	501
87	M	valid	cs only	CR	1044	0.304		1107	501
87	M	valid	paired	CR	858	0.606		997	501
87	M	valid	cs only	CR	1002	0.71		1100	501
87	M	valid	paired	no CR	0		0	0	501
87	M	valid	cs only	CR	1047	0.129		1110	501
87	M	valid	paired	CR	877	0.036		892	501
87	M	valid	cs only	CR	1002	0.06		1154	501
87	M	valid	paired	no CR	0		0	0	501
87	M	valid	cs only	CR	946	0.304		1303	501
87	M	valid	cs only	CR	868	0.707		1145	501
87	M	valid	paired	no CR	0		0	0	501
87	M	valid	cs only	CR	1049	0.655		1428	501

87	M	valid	cs only	CR	1072	0.559		1210	501
87	M	valid	cs only	no CR	0		0	0	501
87	M	valid	paired	no CR	0		0	0	501
87	M	valid	paired	no CR	0		0	0	501
87	M	valid	paired	no CR	0		0	0	501
87	M	valid	paired	no CR	0		0	0	501
87	M	valid	cs only	CR	860	0.263		947	501
87	M	valid	cs only	CR	775	0.291		1438	501
87	M	valid	cs only	no CR	0		0	0	501
87	M	valid	cs only	CR	993	0.398		1280	501
87	M	valid	us only	no CR	0		0	0	501
89	F	invalid	us only	0	0		0	0	501
89	F	valid	us only	CR	598	1.16		715	501
89	F	valid	cs only	no CR	0		0	0	501
89	F	invalid	paired	0	0		0	0	501
89	F	valid	paired	no CR	0		0	0	501
89	F	valid	paired	CR	526	0.79		607	501
89	F	invalid	paired	0	0		0	0	501
89	F	valid	paired	no CR	0		0	0	501
89	F	valid	paired	no CR	0		0	0	501
89	F	valid	paired	no CR	0		0	0	501
89	F	valid	paired	CR	747	1.114		823	501
89	F	valid	paired	CR	627	0.475		677	501
89	F	valid	paired	no CR	0		0	0	501
89	F	valid	paired	no CR	0		0	0	501
89	F	valid	paired	no CR	0		0	0	501
89	F	valid	paired	no CR	0		0	0	501
89	F	valid	paired	no CR	0		0	0	501
89	F	valid	paired	no CR	0		0	0	501
89	F	valid	paired	CR	921	0.625		985	501
89	F	valid	paired	no CR	0		0	0	501
89	F	valid	paired	no CR	0		0	0	501
89	F	valid	paired	no CR	0		0	0	501
89	F	valid	paired	no CR	0		0	0	501
89	F	valid	paired	no CR	0		0	0	501
89	F	valid	paired	no CR	0		0	0	501
89	F	valid	paired	no CR	0		0	0	501
89	F	valid	paired	CR	801	0.344		858	501
89	F	invalid	paired	0	0		0	0	501
89	F	valid	paired	no CR	0		0	0	501
89	F	valid	paired	no CR	0		0	0	501
89	F	valid	paired	no CR	0		0	0	501
89	F	valid	paired	no CR	0		0	0	501
89	F	valid	paired	no CR	0		0	0	501
89	F	valid	paired	no CR	0		0	0	501
89	F	valid	paired	no CR	0		0	0	501
89	F	valid	paired	no CR	0		0	0	501
89	F	invalid	paired	0	0		0	0	501
89	F	valid	paired	no CR	0		0	0	501
89	F	valid	paired	no CR	0		0	0	501

89	F	valid	paired	no CR	0	0	0	501
89	F	valid	paired	no CR	0	0	0	501
89	F	valid	paired	no CR	0	0	0	501
89	F	valid	paired	no CR	0	0	0	501
89	F	valid	paired	no CR	0	0	0	501
89	F	valid	paired	no CR	0	0	0	501
89	F	valid	paired	no CR	0	0	0	501
89	F	valid	paired	no CR	0	0	0	501
89	F	valid	paired	no CR	0	0	0	501
89	F	valid	paired	no CR	0	0	0	501
89	F	valid	cs only	no CR	0	0	0	501
89	F	valid	paired	no CR	0	0	0	501
89	F	valid	paired	no CR	0	0	0	501
89	F	valid	paired	no CR	0	0	0	501
89	F	valid	paired	no CR	0	0	0	501
89	F	valid	paired	no CR	0	0	0	501
89	F	valid	paired	no CR	0	0	0	501
89	F	valid	paired	no CR	0	0	0	501
89	F	valid	paired	no CR	0	0	0	501
89	F	valid	paired	no CR	0	0	0	501
89	F	valid	cs only	no CR	0	0	0	501
89	F	invalid	paired	0	0	0	0	501
89	F	valid	paired	no CR	0	0	0	501
89	F	valid	paired	no CR	0	0	0	501
89	F	valid	paired	no CR	0	0	0	501
89	F	valid	paired	no CR	0	0	0	501
89	F	valid	paired	no CR	0	0	0	501
89	F	valid	paired	no CR	0	0	0	501
89	F	valid	paired	no CR	0	0	0	501
89	F	valid	paired	no CR	0	0	0	501
89	F	valid	cs only	no CR	0	0	0	501
89	F	valid	cs only	no CR	0	0	0	501
89	F	valid	paired	no CR	0	0	0	501
89	F	valid	cs only	no CR	0	0	0	501
89	F	valid	cs only	no CR	0	0	0	501
89	F	valid	paired	CR	566	0.747	711	501
89	F	valid	paired	no CR	0	0	0	501
89	F	valid	cs only	no CR	0	0	0	501
89	F	invalid	cs only	0	0	0	0	501
89	F	valid	paired	no CR	0	0	0	501
89	F	valid	cs only	no CR	0	0	0	501
89	F	valid	paired	no CR	0	0	0	501
89	F	valid	cs only	no CR	0	0	0	501
89	F	valid	paired	CR	577	0.613	665	501
89	F	valid	cs only	CR	922	0.074	968	501
89	F	valid	paired	no CR	0	0	0	501
89	F	valid	cs only	no CR	0	0	0	501
89	F	valid	cs only	no CR	0	0	0	501
89	F	valid	paired	no CR	0	0	0	501
89	F	valid	cs only	no CR	0	0	0	501

89	F	valid	cs only	no CR	0		0	0	501
89	F	valid	cs only	no CR	0		0	0	501
89	F	valid	paired	no CR	0		0	0	501
89	F	valid	paired	no CR	0		0	0	501
89	F	valid	paired	no CR	0		0	0	501
89	F	valid	paired	no CR	0		0	0	501
89	F	valid	cs only	no CR	0		0	0	501
89	F	valid	cs only	no CR	0		0	0	501
89	F	valid	cs only	no CR	0		0	0	501
89	F	invalid	cs only	0	0		0	0	501
89	F	valid	us only	no CR	0		0	0	501
84	M	valid	us only	no CR	0		0	0	501
84	M	valid	us only	no CR	0		0	0	501
84	M	invalid	cs only	0	0		0	0	501
84	M	valid	paired	no CR	0		0	0	501
84	M	valid	paired	no CR	0		0	0	501
84	M	valid	paired	no CR	0		0	0	501
84	M	valid	paired	no CR	0		0	0	501
84	M	valid	paired	no CR	0		0	0	501
84	M	valid	paired	no CR	0		0	0	501
84	M	valid	paired	no CR	0		0	0	501
84	M	valid	paired	no CR	0		0	0	501
84	M	valid	paired	no CR	0		0	0	501
84	M	valid	paired	no CR	0		0	0	501
84	M	valid	paired	no CR	0		0	0	501
84	M	valid	paired	CR	581	0.075		1037	501
84	M	valid	paired	CR	541	0.481		642	501
84	M	valid	paired	no CR	0		0	0	501
84	M	valid	paired	no CR	0		0	0	501
84	M	valid	paired	no CR	0		0	0	501
84	M	valid	paired	no CR	0		0	0	501
84	M	valid	paired	no CR	0		0	0	501
84	M	valid	paired	no CR	0		0	0	501
84	M	valid	paired	no CR	0		0	0	501
84	M	valid	paired	no CR	0		0	0	501
84	M	valid	paired	CR	639	0.607		774	501
84	M	valid	paired	no CR	0		0	0	501
84	M	valid	paired	no CR	0		0	0	501
84	M	valid	paired	no CR	0		0	0	501
84	M	valid	paired	no CR	0		0	0	501
84	M	valid	paired	no CR	0		0	0	501
84	M	valid	paired	no CR	0		0	0	501
84	M	valid	paired	no CR	0		0	0	501
84	M	valid	paired	no CR	0		0	0	501
84	M	valid	paired	no CR	0		0	0	501
84	M	valid	paired	no CR	0		0	0	501
84	M	valid	paired	no CR	0		0	0	501
84	M	valid	paired	no CR	0		0	0	501
84	M	valid	paired	no CR	0		0	0	501
84	M	invalid	paired	0	0		0	0	501

84	M	valid	paired	no CR	0		0	0	501
84	M	valid	paired	no CR	0		0	0	501
84	M	valid	paired	no CR	0		0	0	501
84	M	valid	paired	no CR	0		0	0	501
84	M	valid	paired	no CR	0		0	0	501
84	M	valid	paired	no CR	0		0	0	501
84	M	valid	paired	no CR	0		0	0	501
84	M	valid	paired	no CR	0		0	0	501
84	M	valid	paired	no CR	0		0	0	501
84	M	valid	paired	no CR	0		0	0	501
84	M	valid	cs only	no CR	0		0	0	501
84	M	valid	paired	no CR	0		0	0	501
84	M	valid	paired	no CR	0		0	0	501
84	M	valid	paired	CR	912	0.544		1009	501
84	M	invalid	paired	0	0		0	0	501
84	M	valid	paired	no CR	0		0	0	501
84	M	valid	paired	no CR	0		0	0	501
84	M	valid	paired	no CR	0		0	0	501
84	M	valid	paired	no CR	0		0	0	501
84	M	valid	paired	no CR	0		0	0	501
84	M	valid	cs only	no CR	0		0	0	501
84	M	valid	paired	CR	521	0.279		581	501
84	M	invalid	paired	0	0		0	0	501
84	M	invalid	paired	0	0		0	0	501
84	M	valid	paired	no CR	0		0	0	501
84	M	valid	paired	no CR	0		0	0	501
84	M	valid	paired	no CR	0		0	0	501
84	M	valid	paired	no CR	0		0	0	501
84	M	valid	paired	no CR	0		0	0	501
84	M	valid	paired	no CR	0		0	0	501
84	M	valid	cs only	no CR	0		0	0	501
84	M	valid	cs only	no CR	0		0	0	501
84	M	valid	paired	no CR	0		0	0	501
84	M	invalid	cs only	0	0		0	0	501
84	M	invalid	cs only	0	0		0	0	501
84	M	valid	paired	no CR	0		0	0	501
84	M	valid	paired	no CR	0		0	0	501
84	M	invalid	cs only	0	0		0	0	501
84	M	invalid	cs only	0	0		0	0	501
84	M	valid	paired	no CR	0		0	0	501
84	M	valid	cs only	no CR	0		0	0	501
84	M	valid	paired	no CR	0		0	0	501
84	M	valid	cs only	no CR	0		0	0	501
84	M	valid	paired	no CR	0		0	0	501
84	M	valid	cs only	no CR	0		0	0	501
84	M	valid	paired	no CR	0		0	0	501
84	M	valid	cs only	CR	777	0.248		1436	501
84	M	valid	cs only	no CR	0		0	0	501
84	M	valid	paired	no CR	0		0	0	501
84	M	valid	cs only	no CR	0		0	0	501

84	M	valid	cs only	no CR	0	0	0	501
84	M	valid	cs only	no CR	0	0	0	501
84	M	valid	paired	CR	721	0.134	1001	501
84	M	valid	paired	CR	750	0.122	1039	501
84	M	valid	paired	no CR	0	0	0	501
84	M	valid	paired	no CR	0	0	0	501
84	M	valid	cs only	CR	960	0.768	1308	501
84	M	invalid	cs only	0	0	0	0	501
84	M	valid	cs only	no CR	0	0	0	501
84	M	valid	cs only	CR	805	0.243	1139	501
84	M	valid	us only	no CR	0	0	0	501
81	F	valid	us only	CR	752	1.061	891	501
81	F	valid	us only	no CR	0	0	0	501
81	F	valid	cs only	CR	1100	1.1	1392	501
81	F	valid	paired	no CR	0	0	0	501
81	F	valid	paired	no CR	0	0	0	501
81	F	valid	paired	no CR	0	0	0	501
81	F	valid	paired	CR	800	0.067	898	501
81	F	valid	paired	CR	829	0.539	965	501
81	F	valid	paired	no CR	0	0	0	501
81	F	valid	paired	CR	862	0.791	988	501
81	F	invalid	paired	0	0	0	0	501
81	F	invalid	paired	0	0	0	0	501
81	F	valid	paired	CR	641	0.854	768	501
81	F	valid	paired	CR	860	0.967	944	501
81	F	valid	paired	no CR	0	0	0	501
81	F	valid	paired	no CR	0	0	0	501
81	F	valid	paired	CR	777	1.304	1007	501
81	F	valid	paired	no CR	0	0	0	501
81	F	valid	paired	CR	843	0.896	960	501
81	F	valid	paired	no CR	0	0	0	501
81	F	valid	paired	CR	947	0.056	1039	501
81	F	valid	paired	no CR	0	0	0	501
81	F	valid	paired	no CR	0	0	0	501
81	F	valid	paired	CR	776	0.11	882	501
81	F	valid	paired	no CR	0	0	0	501
81	F	valid	paired	CR	945	0.115	1026	501
81	F	valid	paired	CR	954	0.201	1020	501
81	F	valid	paired	CR	694	1.177	1022	501
81	F	valid	paired	no CR	0	0	0	501
81	F	valid	paired	CR	875	0.931	1028	501
81	F	valid	paired	CR	905	0.898	1026	501
81	F	valid	paired	CR	909	0.187	1047	501
81	F	valid	paired	no CR	0	0	0	501
81	F	valid	paired	no CR	0	0	0	501
81	F	valid	paired	no CR	0	0	0	501
81	F	valid	paired	CR	702	0.358	1017	501
81	F	valid	paired	CR	836	0.73	987	501
81	F	valid	paired	CR	866	0.3	1034	501
81	F	valid	paired	no CR	0	0	0	501

81	F	valid	paired	no CR	0		0	0	501
81	F	valid	paired	no CR	0		0	0	501
81	F	valid	paired	CR	792	0.979		968	501
81	F	valid	paired	CR	889	0.996		971	501
81	F	valid	paired	no CR	0		0	0	501
81	F	valid	paired	no CR	0		0	0	501
81	F	valid	paired	CR	867	1.024		954	501
81	F	valid	paired	no CR	0		0	0	501
81	F	valid	paired	CR	939	1.127		1026	501
81	F	valid	paired	no CR	0		0	0	501
81	F	valid	cs only	no CR	0		0	0	501
81	F	valid	paired	no CR	0		0	0	501
81	F	valid	paired	no CR	0		0	0	501
81	F	valid	paired	no CR	0		0	0	501
81	F	valid	paired	no CR	0		0	0	501
81	F	valid	paired	CR	856	1.042		1015	501
81	F	valid	paired	CR	868	1.141		994	501
81	F	invalid	paired	0	0		0	0	501
81	F	valid	paired	CR	873	0.279		1017	501
81	F	valid	paired	CR	926	0.888		1011	501
81	F	invalid	cs only	0	0		0	0	501
81	F	invalid	paired	0	0		0	0	501
81	F	valid	paired	no CR	0		0	0	501
81	F	valid	paired	no CR	0		0	0	501
81	F	valid	paired	CR	898	0.914		984	501
81	F	valid	paired	no CR	0		0	0	501
81	F	valid	paired	CR	819	0.934		984	501
81	F	valid	paired	no CR	0		0	0	501
81	F	valid	paired	CR	890	0.529		1007	501
81	F	valid	paired	CR	741	0.712		1017	501
81	F	valid	cs only	CR	1080	0.975		1189	501
81	F	valid	cs only	CR	738	0.468		1054	501
81	F	valid	paired	no CR	0		0	0	501
81	F	invalid	cs only	0	0		0	0	501
81	F	valid	cs only	CR	1098	0.745		1245	501
81	F	valid	paired	no CR	0		0	0	501
81	F	valid	paired	CR	674	1.16		872	501
81	F	invalid	cs only	0	0		0	0	501
81	F	valid	cs only	no CR	0		0	0	501
81	F	valid	paired	CR	805	0.855		936	501
81	F	invalid	cs only	0	0		0	0	501
81	F	valid	paired	CR	945	0.05		1011	501
81	F	valid	cs only	no CR	0		0	0	501
81	F	valid	paired	no CR	0		0	0	501
81	F	valid	cs only	CR	962	0.999		1386	501
81	F	valid	paired	no CR	0		0	0	501
81	F	valid	cs only	CR	871	1.167		1112	501
81	F	valid	cs only	no CR	0		0	0	501
81	F	valid	paired	no CR	0		0	0	501
81	F	invalid	cs only	0	0		0	0	501

81	F	valid	cs only	CR	1190	1.029		1418	501
81	F	valid	cs only	CR	832	0.951		1088	501
81	F	valid	paired	no CR	0		0	0	501
81	F	valid	paired	CR	803	0.983		915	501
81	F	valid	paired	no CR	0		0	0	501
81	F	valid	paired	CR	902	0.029		1022	501
81	F	valid	cs only	CR	879	1.114		976	501
81	F	valid	cs only	no CR	0		0	0	501
81	F	valid	cs only	CR	998	0.473		1085	501
81	F	valid	cs only	CR	862	0.869		1011	501
81	F	valid	us only	CR	662	0.724		855	501
83	M	invalid	us only	0	0		0	0	501
83	M	valid	us only	no CR	0		0	0	501
83	M	valid	cs only	no CR	0		0	0	501
83	M	valid	paired	no CR	0		0	0	501
83	M	valid	paired	no CR	0		0	0	501
83	M	valid	paired	no CR	0		0	0	501
83	M	valid	paired	no CR	0		0	0	501
83	M	valid	paired	CR	764	0.541		868	501
83	M	valid	paired	no CR	0		0	0	501
83	M	valid	paired	no CR	0		0	0	501
83	M	valid	paired	CR	766	0.073		800	501
83	M	invalid	paired	0	0		0	0	501
83	M	valid	paired	no CR	0		0	0	501
83	M	invalid	paired	0	0		0	0	501
83	M	valid	paired	no CR	0		0	0	501
83	M	valid	paired	CR	787	0.051		990	501
83	M	valid	paired	no CR	0		0	0	501
83	M	valid	paired	CR	526	0.445		621	501
83	M	valid	paired	no CR	0		0	0	501
83	M	valid	paired	no CR	0		0	0	501
83	M	valid	paired	no CR	0		0	0	501
83	M	valid	paired	no CR	0		0	0	501
83	M	valid	paired	no CR	0		0	0	501
83	M	valid	paired	no CR	0		0	0	501
83	M	valid	paired	no CR	0		0	0	501
83	M	valid	paired	no CR	0		0	0	501
83	M	invalid	paired	0	0		0	0	501
83	M	valid	paired	no CR	0		0	0	501
83	M	valid	paired	no CR	0		0	0	501
83	M	valid	paired	no CR	0		0	0	501
83	M	valid	paired	no CR	0		0	0	501
83	M	invalid	paired	0	0		0	0	501
83	M	valid	paired	no CR	0		0	0	501
83	M	valid	paired	no CR	0		0	0	501
83	M	valid	paired	CR	768	0.706		835	501
83	M	valid	paired	no CR	0		0	0	501
83	M	valid	paired	no CR	0		0	0	501
83	M	valid	paired	no CR	0		0	0	501
83	M	valid	paired	no CR	0		0	0	501

83	M	valid	paired	no CR	0		0	0	501
83	M	valid	paired	no CR	0		0	0	501
83	M	valid	paired	CR	892	0.036		977	501
83	M	valid	paired	CR	551	0.069		1037	501
83	M	valid	paired	no CR	0		0	0	501
83	M	valid	paired	no CR	0		0	0	501
83	M	valid	paired	no CR	0		0	0	501
83	M	valid	paired	no CR	0		0	0	501
83	M	valid	paired	no CR	0		0	0	501
83	M	valid	paired	no CR	0		0	0	501
83	M	valid	cs only	no CR	0		0	0	501
83	M	valid	paired	no CR	0		0	0	501
83	M	valid	paired	no CR	0		0	0	501
83	M	valid	paired	no CR	0		0	0	501
83	M	valid	paired	no CR	0		0	0	501
83	M	valid	paired	CR	853	0.054		888	501
83	M	valid	paired	no CR	0		0	0	501
83	M	valid	paired	no CR	0		0	0	501
83	M	invalid	paired	0	0		0	0	501
83	M	valid	paired	no CR	0		0	0	501
83	M	invalid	cs only	0	0		0	0	501
83	M	valid	paired	no CR	0		0	0	501
83	M	valid	paired	no CR	0		0	0	501
83	M	invalid	paired	0	0		0	0	501
83	M	valid	paired	no CR	0		0	0	501
83	M	valid	paired	no CR	0		0	0	501
83	M	valid	paired	no CR	0		0	0	501
83	M	valid	paired	no CR	0		0	0	501
83	M	valid	paired	no CR	0		0	0	501
83	M	valid	paired	no CR	0		0	0	501
83	M	valid	cs only	no CR	0		0	0	501
83	M	valid	cs only	CR	945	0.019		1025	501
83	M	valid	paired	no CR	0		0	0	501
83	M	valid	cs only	no CR	0		0	0	501
83	M	valid	cs only	CR	866	0.594		992	501
83	M	valid	paired	no CR	0		0	0	501
83	M	valid	paired	no CR	0		0	0	501
83	M	valid	cs only	no CR	0		0	0	501
83	M	valid	cs only	no CR	0		0	0	501
83	M	valid	paired	no CR	0		0	0	501
83	M	invalid	cs only	0	0		0	0	501
83	M	valid	paired	no CR	0		0	0	501
83	M	valid	cs only	no CR	0		0	0	501
83	M	valid	paired	no CR	0		0	0	501
83	M	valid	cs only	no CR	0		0	0	501
83	M	valid	paired	no CR	0		0	0	501
83	M	valid	cs only	no CR	0		0	0	501
83	M	valid	cs only	no CR	0		0	0	501
83	M	valid	paired	no CR	0		0	0	501
83	M	valid	cs only	no CR	0		0	0	501

83	M	valid	cs only	CR	586	0.512		724	501
83	M	valid	cs only	CR	581	0.728		1289	501
83	M	valid	paired	no CR	0		0	0	501
83	M	valid	paired	no CR	0		0	0	501
83	M	valid	paired	no CR	0		0	0	501
83	M	valid	paired	no CR	0		0	0	501
83	M	valid	cs only	CR	890	0.072		1273	501
83	M	valid	cs only	no CR	0		0	0	501
83	M	valid	cs only	no CR	0		0	0	501
83	M	valid	cs only	no CR	0		0	0	501
83	M	invalid	us only	0	0		0	0	501
90	M	invalid	us only	0	0		0	0	501
90	M	valid	us only	no CR	0		0	0	501
90	M	valid	cs only	no CR	0		0	0	501
90	M	valid	paired	CR	700	0.514		1006	501
90	M	valid	paired	no CR	0		0	0	501
90	M	invalid	paired	0	0		0	0	501
90	M	valid	paired	no CR	0		0	0	501
90	M	valid	paired	no CR	0		0	0	501
90	M	valid	paired	no CR	0		0	0	501
90	M	valid	paired	no CR	0		0	0	501
90	M	valid	paired	no CR	0		0	0	501
90	M	valid	paired	no CR	0		0	0	501
90	M	invalid	paired	0	0		0	0	501
90	M	valid	paired	no CR	0		0	0	501
90	M	valid	paired	no CR	0		0	0	501
90	M	valid	paired	no CR	0		0	0	501
90	M	valid	paired	no CR	0		0	0	501
90	M	valid	paired	no CR	0		0	0	501
90	M	invalid	paired	0	0		0	0	501
90	M	valid	paired	no CR	0		0	0	501
90	M	invalid	paired	0	0		0	0	501
90	M	valid	paired	no CR	0		0	0	501
90	M	valid	paired	no CR	0		0	0	501
90	M	valid	paired	no CR	0		0	0	501
90	M	valid	paired	no CR	0		0	0	501
90	M	invalid	paired	0	0		0	0	501
90	M	valid	paired	no CR	0		0	0	501
90	M	invalid	paired	0	0		0	0	501
90	M	valid	paired	CR	677	0.071		745	501
90	M	valid	paired	no CR	0		0	0	501
90	M	valid	paired	no CR	0		0	0	501
90	M	valid	paired	CR	745	0.25		818	501
90	M	valid	paired	CR	904	0.275		998	501
90	M	valid	paired	no CR	0		0	0	501
90	M	valid	paired	no CR	0		0	0	501
90	M	valid	paired	no CR	0		0	0	501
90	M	valid	paired	no CR	0		0	0	501
90	M	valid	paired	no CR	0		0	0	501
90	M	invalid	paired	0	0		0	0	501

90	M	invalid	paired	0	0		0	0	501
90	M	valid	paired	no CR	0		0	0	501
90	M	invalid	paired	0	0		0	0	501
90	M	valid	paired	no CR	0		0	0	501
90	M	valid	paired	no CR	0		0	0	501
90	M	valid	paired	no CR	0		0	0	501
90	M	valid	paired	no CR	0		0	0	501
90	M	valid	paired	no CR	0		0	0	501
90	M	valid	paired	no CR	0		0	0	501
90	M	valid	paired	CR	519	0.233		617	501
90	M	valid	cs only	CR	930	0.07		1201	501
90	M	valid	paired	no CR	0		0	0	501
90	M	valid	paired	no CR	0		0	0	501
90	M	valid	paired	no CR	0		0	0	501
90	M	valid	paired	no CR	0		0	0	501
90	M	valid	paired	CR	526	0.184		643	501
90	M	valid	paired	no CR	0		0	0	501
90	M	invalid	paired	0	0		0	0	501
90	M	valid	paired	CR	954	0.301		1041	501
90	M	valid	paired	no CR	0		0	0	501
90	M	valid	cs only	no CR	0		0	0	501
90	M	valid	paired	no CR	0		0	0	501
90	M	valid	paired	no CR	0		0	0	501
90	M	valid	paired	no CR	0		0	0	501
90	M	valid	paired	CR	809	0.105		1009	501
90	M	invalid	paired	0	0		0	0	501
90	M	valid	paired	no CR	0		0	0	501
90	M	valid	paired	no CR	0		0	0	501
90	M	valid	paired	no CR	0		0	0	501
90	M	valid	paired	CR	973	0.072		998	501
90	M	valid	cs only	no CR	0		0	0	501
90	M	valid	cs only	no CR	0		0	0	501
90	M	valid	paired	CR	760	0.042		800	501
90	M	valid	cs only	CR	736	0.032		796	501
90	M	valid	cs only	no CR	0		0	0	501
90	M	valid	paired	no CR	0		0	0	501
90	M	valid	paired	no CR	0		0	0	501
90	M	valid	cs only	no CR	0		0	0	501
90	M	valid	cs only	no CR	0		0	0	501
90	M	valid	paired	no CR	0		0	0	501
90	M	invalid	cs only	0	0		0	0	501
90	M	valid	paired	no CR	0		0	0	501
90	M	valid	cs only	no CR	0		0	0	501
90	M	valid	paired	no CR	0		0	0	501
90	M	valid	cs only	CR	855	0.024		1025	501
90	M	valid	paired	no CR	0		0	0	501
90	M	valid	cs only	no CR	0		0	0	501
90	M	valid	cs only	no CR	0		0	0	501
90	M	valid	paired	no CR	0		0	0	501
90	M	valid	cs only	no CR	0		0	0	501

90	M	valid	cs only	CR	1192	0.62	1505	501
90	M	invalid	cs only	0	0	0	0	501
90	M	valid	paired	no CR	0	0	0	501
90	M	valid	paired	no CR	0	0	0	501
90	M	valid	paired	no CR	0	0	0	501
90	M	valid	paired	no CR	0	0	0	501
90	M	valid	cs only	no CR	0	0	0	501
90	M	valid	cs only	no CR	0	0	0	501
90	M	valid	cs only	CR	1124	0.518	1467	501
90	M	valid	cs only	no CR	0	0	0	501
90	M	valid	us only	CR	686	0.467	907	501
88	F	valid	us only	no CR	0	0	0	501
88	F	valid	us only	no CR	0	0	0	501
88	F	valid	cs only	CR	1373	0.779	1558	501
88	F	valid	paired	no CR	0	0	0	501
88	F	valid	paired	no CR	0	0	0	501
88	F	valid	paired	no CR	0	0	0	501
88	F	valid	paired	no CR	0	0	0	501
88	F	valid	paired	no CR	0	0	0	501
88	F	valid	paired	no CR	0	0	0	501
88	F	valid	paired	no CR	0	0	0	501
88	F	valid	paired	no CR	0	0	0	501
88	F	valid	paired	no CR	0	0	0	501
88	F	valid	paired	no CR	0	0	0	501
88	F	valid	paired	no CR	0	0	0	501
88	F	valid	paired	no CR	0	0	0	501
88	F	valid	paired	no CR	0	0	0	501
88	F	valid	paired	no CR	0	0	0	501
88	F	valid	paired	no CR	0	0	0	501
88	F	valid	paired	no CR	0	0	0	501
88	F	valid	paired	no CR	0	0	0	501
88	F	valid	paired	no CR	0	0	0	501
88	F	valid	paired	no CR	0	0	0	501
88	F	valid	paired	no CR	0	0	0	501
88	F	valid	paired	no CR	0	0	0	501
88	F	valid	paired	no CR	0	0	0	501
88	F	valid	paired	no CR	0	0	0	501
88	F	valid	paired	no CR	0	0	0	501
88	F	valid	paired	no CR	0	0	0	501
88	F	valid	paired	no CR	0	0	0	501
88	F	valid	paired	no CR	0	0	0	501
88	F	valid	paired	no CR	0	0	0	501
88	F	valid	paired	no CR	0	0	0	501
88	F	valid	paired	CR	557	0.41	673	501
88	F	valid	paired	no CR	0	0	0	501
88	F	valid	paired	no CR	0	0	0	501
88	F	valid	paired	no CR	0	0	0	501
88	F	valid	paired	no CR	0	0	0	501
88	F	valid	paired	no CR	0	0	0	501
88	F	valid	paired	no CR	0	0	0	501
88	F	valid	paired	no CR	0	0	0	501

88	F	valid	paired	no CR	0		0	0	501
88	F	valid	paired	CR	628	0.071		679	501
88	F	valid	paired	no CR	0		0	0	501
88	F	valid	paired	no CR	0		0	0	501
88	F	valid	paired	no CR	0		0	0	501
88	F	valid	paired	no CR	0		0	0	501
88	F	valid	paired	no CR	0		0	0	501
88	F	valid	paired	no CR	0		0	0	501
88	F	invalid	paired	0	0		0	0	501
88	F	valid	paired	no CR	0		0	0	501
88	F	valid	cs only	no CR	0		0	0	501
88	F	valid	paired	CR	645	0.021		668	501
88	F	valid	paired	CR	750	0.042		836	501
88	F	valid	paired	no CR	0		0	0	501
88	F	valid	paired	no CR	0		0	0	501
88	F	valid	paired	no CR	0		0	0	501
88	F	valid	paired	no CR	0		0	0	501
88	F	valid	paired	CR	951	0.518		1062	501
88	F	valid	paired	no CR	0		0	0	501
88	F	valid	paired	no CR	0		0	0	501
88	F	valid	cs only	no CR	0		0	0	501
88	F	valid	paired	no CR	0		0	0	501
88	F	valid	paired	no CR	0		0	0	501
88	F	valid	paired	no CR	0		0	0	501
88	F	valid	paired	no CR	0		0	0	501
88	F	valid	paired	CR	753	0.035		981	501
88	F	valid	paired	no CR	0		0	0	501
88	F	valid	paired	no CR	0		0	0	501
88	F	valid	paired	no CR	0		0	0	501
88	F	valid	paired	no CR	0		0	0	501
88	F	invalid	cs only	0	0		0	0	501
88	F	valid	cs only	no CR	0		0	0	501
88	F	valid	paired	no CR	0		0	0	501
88	F	valid	cs only	no CR	0		0	0	501
88	F	valid	cs only	no CR	0		0	0	501
88	F	valid	paired	no CR	0		0	0	501
88	F	valid	paired	no CR	0		0	0	501
88	F	valid	cs only	CR	1111	0.483		1366	501
88	F	valid	cs only	no CR	0		0	0	501
88	F	invalid	paired	0	0		0	0	501
88	F	valid	cs only	no CR	0		0	0	501
88	F	valid	paired	no CR	0		0	0	501
88	F	valid	cs only	no CR	0		0	0	501
88	F	valid	paired	no CR	0		0	0	501
88	F	valid	cs only	no CR	0		0	0	501
88	F	valid	paired	no CR	0		0	0	501
88	F	valid	cs only	no CR	0		0	0	501
88	F	valid	cs only	no CR	0		0	0	501
88	F	valid	paired	no CR	0		0	0	501
88	F	valid	cs only	no CR	0		0	0	501

88	F	valid	cs only	no CR	0		0	0	501
88	F	valid	cs only	no CR	0		0	0	501
88	F	valid	paired	CR	637	0.466		783	501
88	F	valid	paired	no CR	0		0	0	501
88	F	valid	paired	no CR	0		0	0	501
88	F	valid	paired	no CR	0		0	0	501
88	F	valid	cs only	no CR	0		0	0	501
88	F	valid	cs only	no CR	0		0	0	501
88	F	valid	cs only	no CR	0		0	0	501
88	F	valid	cs only	CR	662	0.749		824	501
88	F	valid	us only	no CR	0		0	0	501
86	F	invalid	us only	0	0		0	0	501
86	F	valid	us only	CR	749	1.241		850	501
86	F	valid	cs only	no CR	0		0	0	501
86	F	valid	paired	no CR	0		0	0	501
86	F	valid	paired	no CR	0		0	0	501
86	F	valid	paired	no CR	0		0	0	501
86	F	valid	paired	no CR	0		0	0	501
86	F	valid	paired	no CR	0		0	0	501
86	F	valid	paired	no CR	0		0	0	501
86	F	valid	paired	no CR	0		0	0	501
86	F	valid	paired	no CR	0		0	0	501
86	F	valid	paired	no CR	0		0	0	501
86	F	valid	paired	no CR	0		0	0	501
86	F	valid	paired	no CR	0		0	0	501
86	F	valid	paired	no CR	0		0	0	501
86	F	valid	paired	no CR	0		0	0	501
86	F	valid	paired	no CR	0		0	0	501
86	F	valid	paired	no CR	0		0	0	501
86	F	valid	paired	no CR	0		0	0	501
86	F	valid	paired	no CR	0		0	0	501
86	F	valid	paired	no CR	0		0	0	501
86	F	valid	paired	no CR	0		0	0	501
86	F	valid	paired	no CR	0		0	0	501
86	F	valid	paired	no CR	0		0	0	501
86	F	valid	paired	no CR	0		0	0	501
86	F	valid	paired	no CR	0		0	0	501
86	F	valid	paired	no CR	0		0	0	501
86	F	valid	paired	CR	928	0.892		1011	501
86	F	valid	paired	no CR	0		0	0	501
86	F	valid	paired	CR	945	0.025		979	501
86	F	valid	paired	CR	928	0.093		1022	501
86	F	valid	paired	CR	979	0.593		1039	501
86	F	valid	paired	no CR	0		0	0	501
86	F	valid	paired	CR	662	0.345		727	501
86	F	valid	paired	CR	971	0.04		1039	501
86	F	valid	paired	CR	875	0.565		1032	501
86	F	valid	paired	no CR	0		0	0	501
86	F	valid	paired	no CR	0		0	0	501
86	F	valid	paired	no CR	0		0	0	501
86	F	valid	paired	no CR	0		0	0	501

86	F	valid	paired	CR	764	0.677		1015	501
86	F	valid	paired	no CR	0		0	0	501
86	F	valid	paired	CR	910	0.759		981	501
86	F	valid	paired	CR	866	0.881		1011	501
86	F	valid	paired	CR	832	0.368		1028	501
86	F	valid	paired	CR	897	0.489		1011	501
86	F	valid	paired	CR	824	0.223		1013	501
86	F	valid	paired	no CR	0		0	0	501
86	F	valid	paired	no CR	0		0	0	501
86	F	valid	paired	no CR	0		0	0	501
86	F	valid	cs only	CR	1186	0.599		1339	501
86	F	valid	paired	no CR	0		0	0	501
86	F	valid	paired	no CR	0		0	0	501
86	F	valid	paired	CR	960	0.791		1026	501
86	F	valid	paired	no CR	0		0	0	501
86	F	valid	paired	CR	856	0.042		890	501
86	F	valid	paired	CR	672	0.07		783	501
86	F	valid	paired	no CR	0		0	0	501
86	F	valid	paired	no CR	0		0	0	501
86	F	valid	paired	CR	951	0.806		1037	501
86	F	valid	cs only	CR	883	0.056		1343	501
86	F	valid	paired	CR	988	0.036		1015	501
86	F	valid	paired	no CR	0		0	0	501
86	F	valid	paired	no CR	0		0	0	501
86	F	valid	paired	no CR	0		0	0	501
86	F	valid	paired	CR	538	0.8		631	501
86	F	valid	paired	CR	946	0.741		1008	501
86	F	valid	paired	CR	911	0.781		1008	501
86	F	valid	paired	no CR	0		0	0	501
86	F	valid	paired	no CR	0		0	0	501
86	F	valid	cs only	CR	891	0.893		995	501
86	F	valid	cs only	CR	861	1.014		950	501
86	F	valid	paired	no CR	0		0	0	501
86	F	valid	cs only	no CR	0		0	0	501
86	F	valid	cs only	no CR	0		0	0	501
86	F	valid	paired	CR	539	0.762		673	501
86	F	valid	paired	no CR	0		0	0	501
86	F	valid	cs only	no CR	0		0	0	501
86	F	valid	cs only	CR	971	1.039		1428	501
86	F	valid	paired	no CR	0		0	0	501
86	F	valid	cs only	CR	878	0.495		1270	501
86	F	valid	paired	no CR	0		0	0	501
86	F	valid	cs only	no CR	0		0	0	501
86	F	valid	paired	CR	941	1.081		1049	501
86	F	valid	cs only	CR	1052	0.651		1107	501
86	F	valid	paired	CR	875	0.516		941	501
86	F	valid	cs only	no CR	0		0	0	501
86	F	valid	cs only	CR	849	0.555		949	501
86	F	valid	paired	no CR	0		0	0	501
86	F	valid	cs only	CR	988	0.359		1079	501

86	F	valid	cs only	no CR	0		0	0	501
86	F	valid	cs only	CR	927	0.909		1093	501
86	F	valid	paired	no CR	0		0	0	501
86	F	valid	paired	CR	863	1.127		976	501
86	F	valid	paired	no CR	0		0	0	501
86	F	valid	paired	no CR	0		0	0	501
86	F	valid	cs only	CR	542	0.573		614	501
86	F	valid	cs only	CR	1007	0.078		1166	501
86	F	valid	cs only	CR	815	0.521		938	501
86	F	valid	cs only	CR	894	0.951		1001	501
86	F	valid	us only	no CR	0		0	0	501
97	M	invalid	us only	0	0		0	0	501
97	M	valid	us only	no CR	0		0	0	501
97	M	valid	cs only	no CR	0		0	0	501
97	M	valid	paired	no CR	0		0	0	501
97	M	valid	paired	no CR	0		0	0	501
97	M	valid	paired	no CR	0		0	0	501
97	M	valid	paired	no CR	0		0	0	501
97	M	invalid	paired	no CR	0		0	0	501
97	M	invalid	paired	no CR	0		0	0	501
97	M	valid	paired	no CR	0		0	0	501
97	M	valid	paired	CR	928	0.323		1034	501
97	M	valid	paired	CR	875	0.788		976	501
97	M	valid	paired	CR	822	0.141		1032	501
97	M	valid	paired	no CR	0		0	0	501
97	M	valid	paired	no CR	0		0	0	501
97	M	valid	paired	CR	926	0.096		1026	501
97	M	valid	paired	CR	587	0.622		707	501
97	M	valid	paired	no CR	0		0	0	501
97	M	valid	paired	CR	864	0.077		1030	501
97	M	valid	paired	no CR	0		0	0	501
97	M	valid	paired	CR	888	0.118		1026	501
97	M	valid	paired	CR	954	0.041		1056	501
97	M	valid	paired	no CR	0		0	0	501
97	M	valid	paired	no CR	0		0	0	501
97	M	valid	paired	CR	851	0.035		998	501
97	M	valid	paired	CR	832	0.152		1032	501
97	M	valid	paired	CR	866	0.065		998	501
97	M	valid	paired	CR	894	0.082		1022	501
97	M	valid	paired	no CR	0		0	0	501
97	M	valid	paired	CR	951	0.308		1031	501
97	M	valid	paired	CR	874	0.175		1030	501
97	M	valid	paired	CR	923	0.454		1022	501
97	M	valid	paired	CR	553	0.868		679	501
97	M	valid	paired	CR	849	0.58		1041	501
97	M	valid	paired	CR	890	0.254		1037	501
97	M	valid	paired	CR	962	0.26		1032	501
97	M	valid	paired	no CR	0		0	0	501
97	M	valid	paired	no CR	0		0	0	501
97	M	valid	paired	no CR	0		0	0	501

97	M	valid	paired	CR	896	0.594		1026	501
97	M	valid	paired	no CR	0		0	0	501
97	M	valid	paired	no CR	0		0	0	501
97	M	valid	paired	no CR	0		0	0	501
97	M	valid	paired	no CR	0		0	0	501
97	M	valid	paired	CR	848	0.782		967	501
97	M	valid	paired	no CR	0		0	0	501
97	M	valid	paired	CR	926	0.494		1032	501
97	M	valid	paired	CR	868	0.133		1017	501
97	M	valid	paired	CR	919	0.322		1020	501
97	M	valid	cs only	CR	915	0.77		1073	501
97	M	valid	paired	no CR	0		0	0	501
97	M	valid	paired	CR	763	0.76		869	501
97	M	valid	paired	CR	892	0.879		1030	501
97	M	valid	paired	CR	907	0.631		1034	501
97	M	valid	paired	CR	883	0.518		1039	501
97	M	valid	paired	CR	813	1.021		1011	501
97	M	valid	paired	CR	805	0.592		1016	501
97	M	valid	paired	CR	849	0.928		1017	501
97	M	valid	paired	CR	885	0.189		1043	501
97	M	valid	cs only	CR	1201	0.847		1580	501
97	M	valid	paired	CR	562	0.451		1026	501
97	M	valid	paired	CR	856	1.051		1028	501
97	M	valid	paired	CR	789	0.883		998	501
97	M	valid	paired	CR	750	0.893		1009	501
97	M	valid	paired	CR	939	0.55		1026	501
97	M	valid	paired	CR	879	0.942		1000	501
97	M	valid	paired	CR	902	0.482		1016	501
97	M	valid	paired	CR	894	0.523		1034	501
97	M	valid	paired	CR	836	0.891		953	501
97	M	valid	cs only	CR	1172	1.244		1682	501
97	M	valid	cs only	CR	1086	0.735		1182	501
97	M	valid	paired	CR	824	0.604		996	501
97	M	valid	cs only	no CR	0		0	0	501
97	M	valid	cs only	CR	1183	0.444		1362	501
97	M	valid	paired	CR	898	0.333		1024	501
97	M	valid	paired	CR	930	0.448		1013	501
97	M	valid	cs only	CR	1000	0.93		1168	501
97	M	valid	cs only	CR	890	0.909		1398	501
97	M	valid	paired	CR	917	0.716		1022	501
97	M	valid	cs only	no CR	0		0	0	501
97	M	valid	paired	no CR	0		0	0	501
97	M	valid	cs only	CR	841	0.649		1422	501
97	M	valid	paired	CR	788	0.756		933	501
97	M	valid	cs only	CR	822	0.789		1200	501
97	M	valid	paired	no CR	0		0	0	501
97	M	valid	cs only	CR	827	0.87		1317	501
97	M	valid	cs only	CR	975	0.883		1069	501
97	M	valid	paired	CR	787	0.754		988	501
97	M	valid	cs only	CR	911	0.689		1049	501

97	M	valid	cs only	CR	839	0.857		1371	501
97	M	valid	cs only	CR	700	0.106		1223	501
97	M	valid	paired	no CR	0		0	0	501
97	M	valid	paired	CR	928	0.767		1020	501
97	M	valid	paired	CR	817	0.768		958	501
97	M	valid	paired	CR	881	0.569		1020	501
97	M	valid	cs only	CR	809	0.806		928	501
97	M	valid	cs only	CR	784	0.946		1386	501
97	M	valid	cs only	CR	912	0.781		1375	501
97	M	valid	cs only	CR	852	0.859		1232	501
97	M	valid	us only	no CR	0		0	0	501
104	M	valid	us only	no CR	0		0	0	501
104	M	valid	us only	no CR	0		0	0	501
104	M	valid	cs only	CR	752	0.617		854	501
104	M	valid	paired	no CR	0		0	0	501
104	M	valid	paired	no CR	0		0	0	501
104	M	valid	paired	no CR	0		0	0	501
104	M	valid	paired	no CR	0		0	0	501
104	M	valid	paired	CR	888	0.577		981	501
104	M	valid	paired	no CR	0		0	0	501
104	M	valid	paired	no CR	0		0	0	501
104	M	valid	paired	no CR	0		0	0	501
104	M	valid	paired	no CR	0		0	0	501
104	M	valid	paired	no CR	0		0	0	501
104	M	valid	paired	no CR	0		0	0	501
104	M	valid	paired	no CR	0		0	0	501
104	M	valid	paired	no CR	0		0	0	501
104	M	valid	paired	no CR	0		0	0	501
104	M	valid	paired	no CR	0		0	0	501
104	M	valid	paired	no CR	0		0	0	501
104	M	valid	paired	no CR	0		0	0	501
104	M	valid	paired	no CR	0		0	0	501
104	M	valid	paired	no CR	0		0	0	501
104	M	valid	paired	no CR	0		0	0	501
104	M	valid	paired	no CR	0		0	0	501
104	M	valid	paired	no CR	0		0	0	501
104	M	valid	paired	no CR	0		0	0	501
104	M	valid	paired	no CR	0		0	0	501
104	M	valid	paired	no CR	0		0	0	501
104	M	valid	paired	no CR	0		0	0	501
104	M	valid	paired	no CR	0		0	0	501
104	M	valid	paired	no CR	0		0	0	501
104	M	valid	paired	no CR	0		0	0	501
104	M	valid	paired	no CR	0		0	0	501
104	M	valid	paired	no CR	0		0	0	501
104	M	valid	paired	no CR	0		0	0	501
104	M	valid	paired	no CR	0		0	0	501
104	M	valid	paired	no CR	0		0	0	501
104	M	valid	paired	no CR	0		0	0	501
104	M	valid	paired	no CR	0		0	0	501

104	M	valid	paired	CR	539	0.868		684	501
104	M	valid	paired	no CR	0		0	0	501
104	M	valid	paired	no CR	0		0	0	501
104	M	valid	paired	no CR	0		0	0	501
104	M	valid	paired	CR	917	0.309		1024	501
104	M	valid	paired	no CR	0		0	0	501
104	M	valid	paired	no CR	0		0	0	501
104	M	valid	paired	no CR	0		0	0	501
104	M	invalid	paired	0	0		0	0	501
104	M	valid	paired	no CR	0		0	0	501
104	M	valid	cs only	no CR	0		0	0	501
104	M	valid	paired	no CR	0		0	0	501
104	M	valid	paired	no CR	0		0	0	501
104	M	valid	paired	no CR	0		0	0	501
104	M	valid	paired	no CR	0		0	0	501
104	M	valid	paired	no CR	0		0	0	501
104	M	valid	paired	no CR	0		0	0	501
104	M	valid	paired	no CR	0		0	0	501
104	M	valid	paired	no CR	0		0	0	501
104	M	valid	paired	no CR	0		0	0	501
104	M	valid	cs only	no CR	0		0	0	501
104	M	valid	paired	no CR	0		0	0	501
104	M	valid	paired	no CR	0		0	0	501
104	M	valid	paired	no CR	0		0	0	501
104	M	valid	paired	no CR	0		0	0	501
104	M	valid	paired	no CR	0		0	0	501
104	M	valid	paired	no CR	0		0	0	501
104	M	valid	paired	no CR	0		0	0	501
104	M	valid	paired	no CR	0		0	0	501
104	M	valid	paired	no CR	0		0	0	501
104	M	valid	cs only	no CR	0		0	0	501
104	M	valid	cs only	no CR	0		0	0	501
104	M	valid	paired	CR	612	0.676		780	501
104	M	valid	cs only	no CR	0		0	0	501
104	M	valid	cs only	no CR	0		0	0	501
104	M	valid	paired	no CR	0		0	0	501
104	M	valid	paired	no CR	0		0	0	501
104	M	valid	cs only	no CR	0		0	0	501
104	M	valid	cs only	no CR	0		0	0	501
104	M	invalid	paired	0	0		0	0	501
104	M	valid	cs only	no CR	0		0	0	501
104	M	valid	paired	CR	905	0.108		980	501
104	M	valid	cs only	no CR	0		0	0	501
104	M	valid	paired	no CR	0		0	0	501
104	M	valid	cs only	CR	1173	0.164		1288	501
104	M	valid	paired	no CR	0		0	0	501
104	M	valid	cs only	no CR	0		0	0	501
104	M	valid	cs only	no CR	0		0	0	501
104	M	valid	paired	no CR	0		0	0	501
104	M	valid	cs only	CR	630	0.072		1284	501

104	M	valid	cs only	CR	785	0.024		1025	501
104	M	valid	cs only	no CR	0		0	0	501
104	M	valid	paired	no CR	0		0	0	501
104	M	valid	paired	no CR	0		0	0	501
104	M	valid	paired	no CR	0		0	0	501
104	M	valid	paired	no CR	0		0	0	501
104	M	valid	cs only	no CR	0		0	0	501
104	M	valid	cs only	no CR	0		0	0	501
104	M	valid	cs only	no CR	0		0	0	501
104	M	valid	cs only	CR	1345	0.034		1407	501
104	M	valid	us only	no CR	0		0	0	501
96	F	valid	us only	no CR	0		0	0	501
96	F	valid	us only	no CR	0		0	0	501
96	F	valid	cs only	CR	1166	0.085		1303	501
96	F	valid	paired	no CR	0		0	0	501
96	F	valid	paired	no CR	0		0	0	501
96	F	valid	paired	no CR	0		0	0	501
96	F	valid	paired	no CR	0		0	0	501
96	F	valid	paired	no CR	0		0	0	501
96	F	invalid	paired	0	0		0	0	501
96	F	valid	paired	no CR	0		0	0	501
96	F	valid	paired	CR	898	0.652		991	501
96	F	invalid	paired	0	0		0	0	501
96	F	valid	paired	no CR	0		0	0	501
96	F	valid	paired	no CR	0		0	0	501
96	F	valid	paired	no CR	0		0	0	501
96	F	invalid	paired	0	0		0	0	501
96	F	valid	paired	no CR	0		0	0	501
96	F	valid	paired	CR	673	0.204		1045	501
96	F	valid	paired	no CR	0		0	0	501
96	F	valid	paired	CR	521	0.329		589	501
96	F	valid	paired	no CR	0		0	0	501
96	F	valid	paired	CR	752	0.193		1041	501
96	F	valid	paired	CR	745	0.342		1041	501
96	F	valid	paired	no CR	0		0	0	501
96	F	valid	paired	no CR	0		0	0	501
96	F	valid	paired	no CR	0		0	0	501
96	F	valid	paired	no CR	0		0	0	501
96	F	valid	paired	no CR	0		0	0	501
96	F	valid	paired	CR	538	0.16		1015	501
96	F	valid	paired	CR	944	0.257		1011	501
96	F	invalid	paired	0	0		0	0	501
96	F	valid	paired	no CR	0		0	0	501
96	F	valid	paired	no CR	0		0	0	501
96	F	valid	paired	no CR	0		0	0	501
96	F	valid	paired	no CR	0		0	0	501
96	F	valid	paired	no CR	0		0	0	501
96	F	valid	paired	no CR	0		0	0	501
96	F	invalid	paired	0	0		0	0	501
96	F	valid	paired	CR	949	0.089		1034	501

96	F	valid	paired	no CR	0		0	0	501
96	F	valid	paired	no CR	0		0	0	501
96	F	invalid	paired	0	0		0	0	501
96	F	invalid	paired	0	0		0	0	501
96	F	invalid	paired	0	0		0	0	501
96	F	valid	paired	no CR	0		0	0	501
96	F	valid	paired	no CR	0		0	0	501
96	F	valid	paired	no CR	0		0	0	501
96	F	valid	paired	no CR	0		0	0	501
96	F	valid	paired	no CR	0		0	0	501
96	F	valid	cs only	CR	960	0.204		1128	501
96	F	valid	paired	no CR	0		0	0	501
96	F	valid	paired	CR	717	0.058		962	501
96	F	valid	paired	CR	945	0.377		992	501
96	F	valid	paired	no CR	0		0	0	501
96	F	valid	paired	CR	902	0.222		1030	501
96	F	invalid	paired	0	0		0	0	501
96	F	valid	paired	no CR	0		0	0	501
96	F	valid	paired	no CR	0		0	0	501
96	F	valid	paired	no CR	0		0	0	501
96	F	valid	cs only	CR	1352	0.443		1418	501
96	F	valid	paired	no CR	0		0	0	501
96	F	valid	paired	no CR	0		0	0	501
96	F	invalid	paired	0	0		0	0	501
96	F	valid	paired	CR	800	0.143		1051	501
96	F	invalid	paired	0	0		0	0	501
96	F	invalid	paired	0	0		0	0	501
96	F	valid	paired	no CR	0		0	0	501
96	F	valid	paired	CR	924	0.462		1026	501
96	F	valid	paired	no CR	0		0	0	501
96	F	valid	cs only	CR	1218	0.107		1290	501
96	F	valid	cs only	CR	999	0.837		1355	501
96	F	invalid	paired	0	0		0	0	501
96	F	valid	cs only	CR	1008	1.078		1389	501
96	F	valid	cs only	CR	931	0.236		1186	501
96	F	valid	paired	no CR	0		0	0	501
96	F	valid	paired	no CR	0		0	0	501
96	F	invalid	cs only	0	0		0	0	501
96	F	valid	cs only	no CR	0		0	0	501
96	F	valid	paired	no CR	0		0	0	501
96	F	valid	cs only	no CR	0		0	0	501
96	F	valid	paired	no CR	0		0	0	501
96	F	valid	cs only	no CR	0		0	0	501
96	F	valid	paired	no CR	0		0	0	501
96	F	valid	cs only	CR	585	0.46		641	501
96	F	invalid	paired	0	0		0	0	501
96	F	valid	cs only	no CR	0		0	0	501
96	F	valid	cs only	no CR	0		0	0	501
96	F	valid	paired	no CR	0		0	0	501
96	F	valid	cs only	no CR	0		0	0	501

96	F	valid	cs only	CR	693	0.294		744	501
96	F	valid	cs only	no CR	0		0	0	501
96	F	valid	paired	no CR	0		0	0	501
96	F	valid	paired	no CR	0		0	0	501
96	F	invalid	paired	0	0		0	0	501
96	F	valid	paired	no CR	0		0	0	501
96	F	valid	cs only	CR	1256	0.327		1484	501
96	F	valid	cs only	no CR	0		0	0	501
96	F	valid	cs only	no CR	0		0	0	501
96	F	valid	cs only	CR	623	0.628		700	501
96	F	valid	us only	no CR	0		0	0	501
94	F	valid	us only	no CR	0		0	0	501
94	F	valid	us only	no CR	0		0	0	501
94	F	valid	cs only	no CR	0		0	0	501
94	F	valid	paired	CR	700	0.058		721	501
94	F	valid	paired	no CR	0		0	0	501
94	F	valid	paired	no CR	0		0	0	501
94	F	valid	paired	no CR	0		0	0	501
94	F	valid	paired	no CR	0		0	0	501
94	F	valid	paired	no CR	0		0	0	501
94	F	valid	paired	no CR	0		0	0	501
94	F	valid	paired	no CR	0		0	0	501
94	F	valid	paired	no CR	0		0	0	501
94	F	valid	paired	CR	527	0.79		634	501
94	F	valid	paired	no CR	0		0	0	501
94	F	valid	paired	no CR	0		0	0	501
94	F	valid	paired	no CR	0		0	0	501
94	F	valid	paired	CR	570	0.742		782	501
94	F	valid	paired	no CR	0		0	0	501
94	F	valid	paired	no CR	0		0	0	501
94	F	valid	paired	no CR	0		0	0	501
94	F	valid	paired	no CR	0		0	0	501
94	F	valid	paired	no CR	0		0	0	501
94	F	valid	paired	no CR	0		0	0	501
94	F	valid	paired	CR	582	0.193		670	501
94	F	valid	paired	no CR	0		0	0	501
94	F	valid	paired	no CR	0		0	0	501
94	F	valid	paired	no CR	0		0	0	501
94	F	valid	paired	no CR	0		0	0	501
94	F	valid	paired	no CR	0		0	0	501
94	F	valid	paired	no CR	0		0	0	501
94	F	valid	paired	no CR	0		0	0	501
94	F	valid	paired	no CR	0		0	0	501
94	F	valid	paired	no CR	0		0	0	501
94	F	valid	paired	no CR	0		0	0	501
94	F	valid	paired	no CR	0		0	0	501
94	F	valid	paired	no CR	0		0	0	501
94	F	valid	paired	no CR	0		0	0	501
94	F	valid	paired	no CR	0		0	0	501
94	F	invalid	paired	0	0		0	0	501

94	F	valid	paired	no CR	0		0	0	501
94	F	valid	paired	no CR	0		0	0	501
94	F	valid	paired	no CR	0		0	0	501
94	F	valid	paired	no CR	0		0	0	501
94	F	valid	paired	no CR	0		0	0	501
94	F	valid	paired	no CR	0		0	0	501
94	F	valid	paired	no CR	0		0	0	501
94	F	valid	paired	no CR	0		0	0	501
94	F	valid	paired	no CR	0		0	0	501
94	F	valid	paired	CR	545	0.662		766	501
94	F	valid	cs only	no CR	0		0	0	501
94	F	valid	paired	CR	947	0.042		1013	501
94	F	valid	paired	no CR	0		0	0	501
94	F	valid	paired	no CR	0		0	0	501
94	F	valid	paired	no CR	0		0	0	501
94	F	valid	paired	no CR	0		0	0	501
94	F	valid	paired	no CR	0		0	0	501
94	F	invalid	paired	0	0		0	0	501
94	F	valid	paired	no CR	0		0	0	501
94	F	valid	paired	CR	617	0.288		711	501
94	F	valid	cs only	no CR	0		0	0	501
94	F	valid	paired	no CR	0		0	0	501
94	F	valid	paired	CR	804	1.76		973	501
94	F	valid	paired	CR	966	0.153		1026	501
94	F	valid	paired	CR	932	0.293		1026	501
94	F	valid	paired	CR	750	0.096		832	501
94	F	valid	paired	no CR	0		0	0	501
94	F	valid	paired	no CR	0		0	0	501
94	F	valid	paired	no CR	0		0	0	501
94	F	valid	paired	no CR	0		0	0	501
94	F	valid	cs only	no CR	0		0	0	501
94	F	valid	cs only	no CR	0		0	0	501
94	F	valid	paired	no CR	0		0	0	501
94	F	valid	cs only	no CR	0		0	0	501
94	F	valid	cs only	CR	1121	0.343		1180	501
94	F	valid	paired	no CR	0		0	0	501
94	F	valid	paired	no CR	0		0	0	501
94	F	valid	cs only	CR	759	0.175		997	501
94	F	valid	cs only	no CR	0		0	0	501
94	F	valid	paired	no CR	0		0	0	501
94	F	valid	cs only	CR	1077	0.355		1146	501
94	F	valid	paired	no CR	0		0	0	501
94	F	valid	cs only	no CR	0		0	0	501
94	F	valid	paired	no CR	0		0	0	501
94	F	valid	cs only	CR	910	0.269		1396	501
94	F	valid	paired	CR	979	0.113		1032	501
94	F	valid	cs only	no CR	0		0	0	501
94	F	valid	cs only	CR	1401	0.819		1562	501
94	F	valid	paired	no CR	0		0	0	501
94	F	valid	cs only	no CR	0		0	0	501

94	F	valid	cs only	no CR	0		0	0	501
94	F	valid	cs only	CR	1175	0.84		1318	501
94	F	valid	paired	no CR	0		0	0	501
94	F	valid	paired	no CR	0		0	0	501
94	F	valid	paired	no CR	0		0	0	501
94	F	valid	paired	no CR	0		0	0	501
94	F	valid	cs only	no CR	0		0	0	501
94	F	valid	cs only	CR	763	0.844		931	501
94	F	valid	cs only	no CR	0		0	0	501
94	F	valid	cs only	CR	1013	1.184		1511	501
94	F	valid	us only	no CR	0		0	0	501
104	F	valid	us only	no CR	0		0	0	501
104	F	valid	us only	no CR	0		0	0	501
104	F	valid	cs only	CR	1843	1.559		1431	501
104	F	valid	paired	no CR	0		0	0	501
104	F	valid	paired	no CR	0		0	0	501
104	F	valid	paired	CR	933	0.792		1004	501
104	F	valid	paired	CR	777	0.805		949	501
104	F	valid	paired	CR	935	1.093		1026	501
104	F	valid	paired	no CR	0		0	0	501
104	F	valid	paired	CR	842	1.452		932	501
104	F	valid	paired	CR	873	0.375		1004	501
104	F	valid	paired	CR	915	0.132		1000	501
104	F	valid	paired	CR	900	0.845		1016	501
104	F	valid	paired	no CR	0		0	0	501
104	F	valid	paired	CR	585	0.956		964	501
104	F	valid	paired	CR	869	1.126		983	501
104	F	valid	paired	CR	816	0.393		980	501
104	F	valid	paired	CR	750	1.11		1007	501
104	F	valid	paired	CR	750	1.331		995	501
104	F	valid	paired	CR	585	0.906		1010	501
104	F	valid	paired	CR	909	1.027		1007	501
104	F	valid	paired	CR	884	0.995		976	501
104	F	valid	paired	CR	707	1.352		966	501
104	F	valid	paired	CR	875	0.877		965	501
104	F	valid	paired	no CR	0		0	0	501
104	F	valid	paired	CR	882	0.953		1022	501
104	F	valid	paired	CR	820	1.225		960	501
104	F	valid	paired	no CR	0		0	0	501
104	F	invalid	paired	no CR	0		0	0	501
104	F	valid	paired	no CR	0		0	0	501
104	F	valid	paired	CR	927	0.798		1016	501
104	F	valid	paired	CR	941	0.111		1020	501
104	F	valid	paired	CR	948	0.741		1019	501
104	F	valid	paired	CR	584	1.035		1016	501
104	F	valid	paired	CR	776	0.986		927	501
104	F	valid	paired	CR	815	0.953		1002	501
104	F	valid	paired	CR	778	0.53		997	501
104	F	valid	paired	CR	823	1.24		972	501
104	F	valid	paired	CR	829	1.072		998	501

104	F	invalid	paired	no CR	0		0	0	501
104	F	valid	paired	CR	797	0.99		927	501
104	F	valid	paired	CR	769	1.046		1016	501
104	F	valid	paired	no CR	0		0	0	501
104	F	valid	paired	CR	532	0.452		953	501
104	F	valid	paired	CR	799	1.024		902	501
104	F	valid	paired	CR	631	1.126		876	501
104	F	valid	paired	no CR	0		0	0	501
104	F	valid	paired	CR	602	0.876		933	501
104	F	valid	paired	CR	909	0.973		992	501
104	F	valid	cs only	CR	536	1.242		1066	501
104	F	valid	paired	CR	828	0.2		960	501
104	F	valid	paired	CR	933	1.481		1022	501
104	F	valid	paired	no CR	0		0	0	501
104	F	valid	paired	CR	542	1.957		939	501
104	F	invalid	paired	no CR	0		0	0	501
104	F	invalid	paired	0	0		0	0	501
104	F	valid	paired	CR	790	0.876		1022	501
104	F	valid	paired	CR	775	1.364		865	501
104	F	valid	paired	CR	619	1.195		966	501
104	F	valid	cs only	CR	1828	0.935		980	501
104	F	valid	paired	CR	822	0.734		899	501
104	F	valid	paired	CR	758	1.122		936	501
104	F	valid	paired	CR	895	1.094		1018	501
104	F	valid	paired	CR	642	0.877		936	501
104	F	valid	paired	CR	780	1.084		1009	501
104	F	valid	paired	CR	854	0.903		931	501
104	F	valid	paired	CR	855	1.35		969	501
104	F	valid	paired	CR	859	1.25		1027	501
104	F	valid	paired	no CR	0		0	0	501
104	F	valid	cs only	CR	779	0.823		903	501
104	F	valid	cs only	CR	871	1.024		983	501
104	F	valid	paired	CR	544	1.276		917	501
104	F	valid	cs only	CR	531	1.215		918	501
104	F	valid	cs only	CR	608	1.133		924	501
104	F	valid	paired	no CR	0		0	0	501
104	F	valid	paired	no CR	0		0	0	501
104	F	valid	cs only	CR	851	1.216		963	501
104	F	valid	cs only	CR	560	1.413		966	501
104	F	invalid	paired	0	0		0	0	501
104	F	valid	cs only	CR	803	0.788		882	501
104	F	invalid	paired	no CR	0		0	0	501
104	F	valid	cs only	CR	793	0.67		903	501
104	F	valid	paired	CR	682	0.568		1008	501
104	F	valid	cs only	CR	622	0.618		960	501
104	F	valid	paired	no CR	0		0	0	501
104	F	valid	cs only	no CR	0		0	0	501
104	F	invalid	cs only	0	0		0	0	501
104	F	valid	paired	CR	813	0.233		945	501
104	F	valid	cs only	CR	734	0.938		924	501

104	F	valid	cs only	CR	848	0.858		935	501
104	F	valid	cs only	CR	573	0.876		1004	501
104	F	valid	paired	CR	697	1.172		921	501
104	F	valid	paired	CR	843	0.197		979	501
104	F	valid	paired	CR	816	1.157		911	501
104	F	valid	paired	no CR	0		0	0	501
104	F	valid	cs only	no CR	0		0	0	501
104	F	valid	cs only	CR	816	0.361		1465	501
104	F	valid	cs only	CR	2001	1.942		724	501
104	F	valid	cs only	CR	872	0.8		978	501
104	F	valid	us only	no CR	0		0	0	501
113	F	valid	us only	no CR	0		0	0	501
113	F	valid	us only	no CR	0		0	0	501
113	F	valid	cs only	no CR	0		0	0	501
113	F	valid	paired	no CR	0		0	0	501
113	F	valid	paired	no CR	0		0	0	501
113	F	valid	paired	no CR	0		0	0	501
113	F	valid	paired	no CR	0		0	0	501
113	F	valid	paired	no CR	0		0	0	501
113	F	valid	paired	no CR	0		0	0	501
113	F	valid	paired	no CR	0		0	0	501
113	F	valid	paired	no CR	0		0	0	501
113	F	valid	paired	no CR	0		0	0	501
113	F	valid	paired	no CR	0		0	0	501
113	F	valid	paired	no CR	0		0	0	501
113	F	valid	paired	no CR	0		0	0	501
113	F	valid	paired	no CR	0		0	0	501
113	F	valid	paired	no CR	0		0	0	501
113	F	invalid	paired	0	0		0	0	501
113	F	valid	paired	CR	915	0.161		1032	501
113	F	valid	paired	no CR	0		0	0	501
113	F	valid	paired	no CR	0		0	0	501
113	F	valid	paired	no CR	0		0	0	501
113	F	valid	paired	no CR	0		0	0	501
113	F	valid	paired	no CR	0		0	0	501
113	F	valid	paired	no CR	0		0	0	501
113	F	valid	paired	no CR	0		0	0	501
113	F	valid	paired	no CR	0		0	0	501
113	F	valid	paired	no CR	0		0	0	501
113	F	valid	paired	no CR	0		0	0	501
113	F	valid	paired	CR	530	0.79		619	501
113	F	valid	paired	no CR	0		0	0	501
113	F	valid	paired	no CR	0		0	0	501
113	F	valid	paired	no CR	0		0	0	501
113	F	valid	paired	no CR	0		0	0	501
113	F	valid	paired	no CR	0		0	0	501
113	F	valid	paired	no CR	0		0	0	501
113	F	valid	paired	no CR	0		0	0	501
113	F	valid	paired	no CR	0		0	0	501
113	F	valid	paired	no CR	0		0	0	501

113	F	valid	paired	no CR	0	0	0	501
113	F	valid	paired	no CR	0	0	0	501
113	F	valid	paired	no CR	0	0	0	501
113	F	valid	paired	no CR	0	0	0	501
113	F	valid	paired	CR	843	0.097	915	501
113	F	valid	paired	no CR	0	0	0	501
113	F	valid	paired	no CR	0	0	0	501
113	F	valid	paired	no CR	0	0	0	501
113	F	valid	paired	no CR	0	0	0	501
113	F	valid	paired	no CR	0	0	0	501
113	F	valid	cs only	no CR	0	0	0	501
113	F	valid	paired	no CR	0	0	0	501
113	F	valid	paired	no CR	0	0	0	501
113	F	valid	paired	no CR	0	0	0	501
113	F	valid	paired	no CR	0	0	0	501
113	F	valid	paired	no CR	0	0	0	501
113	F	valid	paired	no CR	0	0	0	501
113	F	valid	paired	no CR	0	0	0	501
113	F	valid	paired	no CR	0	0	0	501
113	F	valid	paired	no CR	0	0	0	501
113	F	valid	cs only	no CR	0	0	0	501
113	F	invalid	paired	0	0	0	0	501
113	F	valid	paired	no CR	0	0	0	501
113	F	valid	paired	no CR	0	0	0	501
113	F	valid	paired	no CR	0	0	0	501
113	F	valid	paired	no CR	0	0	0	501
113	F	valid	paired	no CR	0	0	0	501
113	F	valid	paired	no CR	0	0	0	501
113	F	valid	paired	no CR	0	0	0	501
113	F	valid	paired	no CR	0	0	0	501
113	F	valid	cs only	CR	815	0.208	908	501
113	F	valid	cs only	no CR	0	0	0	501
113	F	valid	paired	no CR	0	0	0	501
113	F	valid	cs only	no CR	0	0	0	501
113	F	valid	cs only	no CR	0	0	0	501
113	F	valid	paired	CR	851	0.081	898	501
113	F	valid	paired	no CR	0	0	0	501
113	F	valid	cs only	CR	1066	0.048	1318	501
113	F	invalid	cs only	0	0	0	0	501
113	F	valid	paired	no CR	0	0	0	501
113	F	valid	cs only	no CR	0	0	0	501
113	F	valid	paired	no CR	0	0	0	501
113	F	valid	cs only	no CR	0	0	0	501
113	F	valid	paired	no CR	0	0	0	501
113	F	valid	cs only	no CR	0	0	0	501
113	F	valid	paired	no CR	0	0	0	501
113	F	valid	cs only	no CR	0	0	0	501
113	F	valid	cs only	no CR	0	0	0	501
113	F	valid	paired	no CR	0	0	0	501
113	F	valid	cs only	no CR	0	0	0	501

113	F	valid	cs only	no CR	0	0	0	501
113	F	valid	cs only	no CR	0	0	0	501
113	F	valid	paired	no CR	0	0	0	501
113	F	valid	paired	no CR	0	0	0	501
113	F	valid	paired	no CR	0	0	0	501
113	F	valid	paired	no CR	0	0	0	501
113	F	valid	cs only	no CR	0	0	0	501
113	F	valid	cs only	CR	1473	0.005	1526	501
113	F	invalid	cs only	0	0	0	0	501
113	F	valid	cs only	no CR	0	0	0	501
113	F	valid	us only	no CR	0	0	0	501
108	F	valid	us only	no CR	0	0	0	501
108	F	valid	us only	no CR	0	0	0	501
108	F	valid	cs only	CR	922	0.104	964	501
108	F	valid	paired	no CR	0	0	0	501
108	F	valid	paired	no CR	0	0	0	501
108	F	invalid	paired	0	0	0	0	501
108	F	valid	paired	no CR	0	0	0	501
108	F	valid	paired	no CR	0	0	0	501
108	F	valid	paired	CR	750	1.663	992	501
108	F	valid	paired	no CR	0	0	0	501
108	F	valid	paired	no CR	0	0	0	501
108	F	valid	paired	CR	750	1.9	811	501
108	F	valid	paired	no CR	0	0	0	501
108	F	valid	paired	no CR	0	0	0	501
108	F	valid	paired	no CR	0	0	0	501
108	F	valid	paired	CR	687	1.631	796	501
108	F	valid	paired	no CR	0	0	0	501
108	F	valid	paired	no CR	0	0	0	501
108	F	valid	paired	CR	890	1.318	997	501
108	F	valid	paired	CR	818	0.374	899	501
108	F	valid	paired	no CR	0	0	0	501
108	F	valid	paired	CR	528	0.74	568	501
108	F	valid	paired	no CR	0	0	0	501
108	F	invalid	paired	0	0	0	0	501
108	F	invalid	paired	0	0	0	0	501
108	F	valid	paired	no CR	0	0	0	501
108	F	valid	paired	no CR	0	0	0	501
108	F	valid	paired	no CR	0	0	0	501
108	F	valid	paired	no CR	0	0	0	501
108	F	valid	paired	CR	941	0.666	989	501
108	F	valid	paired	no CR	0	0	0	501
108	F	valid	paired	CR	913	0.309	998	501
108	F	valid	paired	no CR	0	0	0	501
108	F	valid	paired	no CR	0	0	0	501
108	F	valid	paired	no CR	0	0	0	501
108	F	valid	paired	no CR	0	0	0	501
108	F	valid	paired	no CR	0	0	0	501
108	F	valid	paired	no CR	0	0	0	501
108	F	valid	paired	CR	887	0.169	937	501

108	F	valid	paired	no CR	0		0	0	501
108	F	valid	paired	CR	750	-0.037		968	501
108	F	valid	paired	no CR	0		0	0	501
108	F	valid	paired	no CR	0		0	0	501
108	F	valid	paired	no CR	0		0	0	501
108	F	valid	paired	no CR	0		0	0	501
108	F	valid	paired	no CR	0		0	0	501
108	F	valid	paired	no CR	0		0	0	501
108	F	valid	paired	no CR	0		0	0	501
108	F	valid	paired	CR	711	0.544		777	501
108	F	valid	cs only	CR	855	1.805		1117	501
108	F	valid	paired	no CR	0		0	0	501
108	F	valid	paired	no CR	0		0	0	501
108	F	valid	paired	no CR	0		0	0	501
108	F	valid	paired	no CR	0		0	0	501
108	F	valid	paired	no CR	0		0	0	501
108	F	valid	paired	no CR	0		0	0	501
108	F	valid	paired	no CR	0		0	0	501
108	F	valid	paired	no CR	0		0	0	501
108	F	valid	paired	no CR	0		0	0	501
108	F	valid	cs only	no CR	0		0	0	501
108	F	valid	paired	no CR	0		0	0	501
108	F	valid	paired	no CR	0		0	0	501
108	F	valid	paired	CR	677	0.408		732	501
108	F	valid	paired	no CR	0		0	0	501
108	F	valid	paired	no CR	0		0	0	501
108	F	valid	paired	no CR	0		0	0	501
108	F	valid	paired	no CR	0		0	0	501
108	F	valid	paired	CR	530	0.401		607	501
108	F	invalid	paired	0	0		0	0	501
108	F	valid	cs only	no CR	0		0	0	501
108	F	valid	cs only	no CR	0		0	0	501
108	F	valid	paired	no CR	0		0	0	501
108	F	valid	cs only	no CR	0		0	0	501
108	F	valid	cs only	no CR	0		0	0	501
108	F	valid	paired	no CR	0		0	0	501
108	F	invalid	paired	0	0		0	0	501
108	F	valid	cs only	CR	1201	2.04		1773	501
108	F	valid	cs only	CR	1198	2.002		1765	501
108	F	valid	paired	no CR	0		0	0	501
108	F	valid	cs only	no CR	0		0	0	501
108	F	valid	paired	no CR	0		0	0	501
108	F	valid	cs only	no CR	0		0	0	501
108	F	valid	paired	no CR	0		0	0	501
108	F	valid	cs only	no CR	0		0	0	501
108	F	valid	paired	no CR	0		0	0	501
108	F	valid	cs only	no CR	0		0	0	501
108	F	valid	cs only	CR	515	1.908		589	501
108	F	valid	paired	no CR	0		0	0	501
108	F	valid	cs only	no CR	0		0	0	501

108	F	valid	cs only	no CR	0		0	0	501
108	F	valid	cs only	CR	890	1.173		1065	501
108	F	valid	paired	no CR	0		0	0	501
108	F	valid	paired	no CR	0		0	0	501
108	F	valid	paired	no CR	0		0	0	501
108	F	valid	paired	CR	544	0.713		648	501
108	F	valid	cs only	no CR	0		0	0	501
108	F	valid	cs only	no CR	0		0	0	501
108	F	valid	cs only	CR	585	1.754		747	501
108	F	valid	cs only	no CR	0		0	0	501
108	F	valid	us only	no CR	0		0	0	501
109	F	invalid	us only	0	0		0	0	501
109	F	valid	us only	no CR	0		0	0	501
109	F	valid	cs only	CR	1201	1.073		1284	501
109	F	valid	paired	CR	864	1.04		946	501
109	F	valid	paired	CR	938	1.193		1017	501
109	F	valid	paired	CR	981	0.724		1015	501
109	F	valid	paired	CR	890	0.824		1026	501
109	F	valid	paired	CR	859	1.108		962	501
109	F	valid	paired	CR	602	1.021		890	501
109	F	valid	paired	CR	834	0.095		1024	501
109	F	valid	paired	CR	815	0.027		922	501
109	F	valid	paired	CR	812	1.224		937	501
109	F	valid	paired	CR	729	1.293		793	501
109	F	valid	paired	CR	897	1.22		977	501
109	F	valid	paired	no CR	0		0	0	501
109	F	valid	paired	CR	920	1.279		986	501
109	F	valid	paired	CR	916	1.099		973	501
109	F	valid	paired	CR	522	1.447		917	501
109	F	valid	paired	CR	964	0.913		1009	501
109	F	valid	paired	CR	863	0.987		948	501
109	F	valid	paired	CR	866	0.971		974	501
109	F	valid	paired	CR	859	0.99		900	501
109	F	valid	paired	CR	540	1.015		1011	501
109	F	valid	paired	CR	739	0.975		837	501
109	F	valid	paired	CR	752	0.992		891	501
109	F	valid	paired	CR	843	1.348		946	501
109	F	valid	paired	CR	581	0.022		643	501
109	F	valid	paired	CR	826	0.978		998	501
109	F	valid	paired	CR	811	0.928		899	501
109	F	valid	paired	CR	882	0.908		997	501
109	F	valid	paired	CR	764	0.831		916	501
109	F	valid	paired	CR	961	0.282		1010	501
109	F	valid	paired	CR	931		1	967	501
109	F	valid	paired	CR	604	0.89		769	501
109	F	invalid	paired	0	0		0	0	501
109	F	valid	paired	CR	834	0.982		901	501
109	F	valid	paired	no CR	0		0	0	501
109	F	valid	paired	CR	783	0.931		878	501
109	F	valid	paired	CR	868	0.805		962	501

109	F	valid	paired	CR	870	0.888		971	501
109	F	valid	paired	no CR	0		0	0	501
109	F	valid	paired	CR	773	0.974		876	501
109	F	valid	paired	CR	930	0.276		1017	501
109	F	valid	paired	CR	841	0.974		1002	501
109	F	valid	paired	CR	530	1.285		941	501
109	F	valid	paired	CR	805	0.919		891	501
109	F	valid	paired	CR	799	1.05		984	501
109	F	valid	paired	CR	771	0.973		929	501
109	F	valid	paired	CR	832	0.932		914	501
109	F	valid	cs only	CR	943	1.011		1497	501
109	F	valid	paired	CR	852	0.524		939	501
109	F	valid	paired	CR	827	0.866		1000	501
109	F	valid	paired	CR	796	1.039		894	501
109	F	valid	paired	CR	871	0.927		905	501
109	F	valid	paired	CR	807	0.547		943	501
109	F	valid	paired	CR	728	1.005		849	501
109	F	invalid	paired	0	0		0	0	501
109	F	valid	paired	CR	831	0.9		961	501
109	F	valid	paired	CR	836	0.498		1020	501
109	F	valid	cs only	CR	1003	0.853		1093	501
109	F	valid	paired	CR	876	0.886		999	501
109	F	valid	paired	CR	847	0.276		928	501
109	F	valid	paired	no CR	0		0	0	501
109	F	valid	paired	CR	679	0.965		944	501
109	F	valid	paired	no CR	0		0	0	501
109	F	valid	paired	no CR	0		0	0	501
109	F	valid	paired	CR	915	0.082		973	501
109	F	valid	paired	no CR	0		0	0	501
109	F	valid	paired	CR	824	0.862		964	501
109	F	valid	cs only	no CR	0		0	0	501
109	F	valid	cs only	no CR	0		0	0	501
109	F	valid	paired	no CR	0		0	0	501
109	F	valid	cs only	CR	868	0.956		1314	501
109	F	valid	cs only	CR	802	1.037		1494	501
109	F	valid	paired	no CR	0		0	0	501
109	F	valid	paired	no CR	0		0	0	501
109	F	valid	cs only	CR	788	0.955		931	501
109	F	valid	cs only	CR	894	0.99		997	501
109	F	valid	paired	CR	792	0.836		932	501
109	F	valid	cs only	CR	770	1.465		1462	501
109	F	valid	paired	CR	831	0.957		955	501
109	F	valid	cs only	CR	832	0.964		982	501
109	F	valid	paired	no CR	0		0	0	501
109	F	valid	cs only	CR	933	0.934		1211	501
109	F	valid	paired	CR	819	0.392		911	501
109	F	valid	cs only	CR	769	1.041		1014	501
109	F	valid	cs only	no CR	0		0	0	501
109	F	valid	paired	no CR	0		0	0	501
109	F	valid	cs only	CR	946	1.118		1253	501

109	F	valid	cs only	no CR	0		0	0	501
109	F	valid	cs only	CR	884	0.954		1276	501
109	F	valid	paired	no CR	0		0	0	501
109	F	valid	paired	CR	902	1.214		967	501
109	F	valid	paired	no CR	0		0	0	501
109	F	valid	paired	CR	767	0.59		884	501
109	F	valid	cs only	CR	887	1.089		1033	501
109	F	valid	cs only	CR	581	0.967		1290	501
109	F	invalid	cs only	0	0		0	0	501
109	F	valid	cs only	CR	823	1.062		1308	501
109	F	valid	us only	no CR	0		0	0	501
111	M	valid	us only	no CR	0		0	0	501
111	M	valid	us only	no CR	0		0	0	501
111	M	invalid	cs only	0	0		0	0	501
111	M	valid	paired	CR	926	0.108		1004	501
111	M	valid	paired	no CR	0		0	0	501
111	M	valid	paired	no CR	0		0	0	501
111	M	valid	paired	CR	899	0.366		969	501
111	M	valid	paired	no CR	0		0	0	501
111	M	valid	paired	no CR	0		0	0	501
111	M	valid	paired	no CR	0		0	0	501
111	M	valid	paired	no CR	0		0	0	501
111	M	valid	paired	no CR	0		0	0	501
111	M	valid	paired	CR	872	0.347		940	501
111	M	valid	paired	no CR	0		0	0	501
111	M	valid	paired	no CR	0		0	0	501
111	M	valid	paired	CR	804	0.147		920	501
111	M	valid	paired	no CR	0		0	0	501
111	M	valid	paired	no CR	0		0	0	501
111	M	valid	paired	CR	878	1.044		1002	501
111	M	valid	paired	CR	711	0.595		834	501
111	M	valid	paired	CR	898	0.206		1017	501
111	M	valid	paired	CR	877	0.064		1037	501
111	M	valid	paired	CR	845	1.097		1016	501
111	M	valid	paired	no CR	0		0	0	501
111	M	valid	paired	no CR	0		0	0	501
111	M	valid	paired	CR	886	0.893		990	501
111	M	valid	paired	no CR	0		0	0	501
111	M	valid	paired	no CR	0		0	0	501
111	M	valid	paired	no CR	0		0	0	501
111	M	valid	paired	no CR	0		0	0	501
111	M	valid	paired	CR	934	0.399		1020	501
111	M	valid	paired	CR	911	0.103		1031	501
111	M	valid	paired	CR	809	0.739		937	501
111	M	valid	paired	no CR	0		0	0	501
111	M	valid	paired	no CR	0		0	0	501
111	M	valid	paired	CR	519	1.015		994	501
111	M	valid	paired	CR	743	0.928		985	501
111	M	valid	paired	CR	827	0.349		917	501
111	M	valid	paired	CR	536	1.236		947	501

111	M	valid	paired	CR	547	0.107		1002	501
111	M	valid	paired	CR	826	0.777		1008	501
111	M	valid	paired	no CR	0		0	0	501
111	M	valid	paired	no CR	0		0	0	501
111	M	valid	paired	no CR	0		0	0	501
111	M	valid	paired	CR	691	0.32		844	501
111	M	valid	paired	no CR	0		0	0	501
111	M	valid	paired	no CR	0		0	0	501
111	M	valid	paired	CR	870	0.277		1017	501
111	M	valid	paired	no CR	0		0	0	501
111	M	valid	cs only	CR	985	0.699		1184	501
111	M	valid	paired	CR	775	0.715		976	501
111	M	valid	paired	CR	917	0.772		1011	501
111	M	valid	paired	no CR	0		0	0	501
111	M	valid	paired	CR	883	0.623		1017	501
111	M	valid	paired	CR	751	0.882		1007	501
111	M	valid	paired	CR	824	0.377		962	501
111	M	valid	paired	CR	936	0.689		1009	501
111	M	valid	paired	no CR	0		0	0	501
111	M	valid	paired	CR	798	0.311		932	501
111	M	valid	cs only	CR	834	0.249		1277	501
111	M	valid	paired	no CR	0		0	0	501
111	M	valid	paired	no CR	0		0	0	501
111	M	valid	paired	CR	905	0.491		1017	501
111	M	valid	paired	CR	750	0.395		1013	501
111	M	valid	paired	CR	927	0.247		995	501
111	M	valid	paired	CR	853	0.729		1024	501
111	M	valid	paired	no CR	0		0	0	501
111	M	valid	paired	CR	786	0.502		961	501
111	M	invalid	paired	0	0		0	0	501
111	M	valid	cs only	CR	967	0.934		1235	501
111	M	valid	cs only	CR	833	0.381		915	501
111	M	valid	paired	CR	928	0.652		1026	501
111	M	valid	cs only	CR	888	0.878		1430	501
111	M	valid	cs only	CR	843	1.398		1495	501
111	M	valid	paired	no CR	0		0	0	501
111	M	valid	paired	CR	765	0.44		855	501
111	M	valid	cs only	CR	598	0.304		688	501
111	M	valid	cs only	CR	1002	0.79		1275	501
111	M	valid	paired	CR	859	0.462		1018	501
111	M	valid	cs only	CR	979	0.778		1528	501
111	M	valid	paired	no CR	0		0	0	501
111	M	valid	cs only	CR	1143	0.644		1871	501
111	M	valid	paired	CR	619	0.312		821	501
111	M	valid	cs only	no CR	0		0	0	501
111	M	valid	paired	CR	832	0.059		958	501
111	M	valid	cs only	CR	1260	0.031		1335	501
111	M	valid	cs only	CR	695	0.325		1026	501
111	M	valid	paired	CR	519	0.48		579	501
111	M	invalid	cs only	0	0		0	0	501

111	M	valid	cs only	CR	717	0.817		1065	501
111	M	valid	cs only	CR	1192	0.793		1618	501
111	M	valid	paired	no CR	0		0	0	501
111	M	valid	paired	no CR	0		0	0	501
111	M	valid	paired	no CR	0		0	0	501
111	M	valid	paired	CR	877	0.313		994	501
111	M	valid	cs only	CR	1094	0.747		1486	501
111	M	valid	cs only	CR	1064	0.858		1146	501
111	M	valid	cs only	CR	555	0.082		717	501
111	M	valid	cs only	CR	753	0.923		1018	501
111	M	valid	us only	no CR	0		0	0	501
108	F	invalid	us only	0	0		0	0	501
108	F	valid	us only	no CR	0		0	0	501
108	F	invalid	cs only	0	0		0	0	501
108	F	invalid	paired	0	0		0	0	501
108	F	invalid	paired	0	0		0	0	501
108	F	invalid	paired	0	0		0	0	501
108	F	invalid	paired	no CR	0		0	0	501
108	F	valid	paired	CR	949	0.667		1004	501
108	F	valid	paired	CR	919	0.651		978	501
108	F	valid	paired	no CR	0		0	0	501
108	F	valid	paired	no CR	0		0	0	501
108	F	invalid	paired	0	0		0	0	501
108	F	invalid	paired	0	0		0	0	501
108	F	valid	paired	CR	912	0.879		994	501
108	F	valid	paired	CR	612	0.72		667	501
108	F	valid	paired	no CR	0		0	0	501
108	F	valid	paired	CR	660	1.009		830	501
108	F	valid	paired	CR	871	0.791		945	501
108	F	valid	paired	CR	658	0.625		829	501
108	F	valid	paired	CR	879	0.7		967	501
108	F	valid	paired	CR	765	0.646		895	501
108	F	valid	paired	CR	849	0.739		948	501
108	F	valid	paired	no CR	0		0	0	501
108	F	valid	paired	CR	794	0.547		867	501
108	F	valid	paired	CR	791	0.689		933	501
108	F	valid	paired	CR	808	0.68		883	501
108	F	invalid	paired	0	0		0	0	501
108	F	invalid	paired	no CR	0		0	0	501
108	F	valid	paired	no CR	0		0	0	501
108	F	invalid	paired	0	0		0	0	501
108	F	valid	paired	CR	891	0.688		1009	501
108	F	valid	paired	no CR	0		0	0	501
108	F	valid	paired	CR	743	0.726		877	501
108	F	valid	paired	CR	922	0.553		985	501
108	F	valid	paired	CR	803	0.654		888	501
108	F	valid	paired	CR	834	0.724		936	501
108	F	valid	paired	CR	910	0.65		956	501
108	F	valid	paired	CR	913	0.387		971	501
108	F	valid	paired	no CR	0		0	0	501

108	F	valid	paired	CR	898	0.629		1019	501
108	F	valid	paired	no CR	0		0	0	501
108	F	valid	paired	CR	918	0.685		949	501
108	F	valid	paired	CR	900	0.436		943	501
108	F	valid	paired	CR	835	0.532		866	501
108	F	valid	paired	no CR	0		0	0	501
108	F	invalid	paired	0	0		0	0	501
108	F	invalid	paired	0	0		0	0	501
108	F	valid	paired	CR	967	0.631		1032	501
108	F	valid	paired	CR	890	0.587		971	501
108	F	valid	cs only	CR	732	1.273		826	501
108	F	valid	paired	CR	515	0.667		859	501
108	F	valid	paired	CR	876	0.727		941	501
108	F	valid	paired	CR	697	0.649		872	501
108	F	valid	paired	no CR	0		0	0	501
108	F	valid	paired	CR	622	0.677		760	501
108	F	invalid	paired	0	0		0	0	501
108	F	valid	paired	CR	958	0.617		1002	501
108	F	valid	paired	CR	723	0.631		993	501
108	F	valid	paired	CR	750	0.33		936	501
108	F	valid	cs only	CR	1177	0.613		1439	501
108	F	valid	paired	CR	791	0.817		1000	501
108	F	valid	paired	no CR	0		0	0	501
108	F	valid	paired	CR	884	0.544		984	501
108	F	valid	paired	CR	786	0.596		850	501
108	F	valid	paired	CR	777	0.719		822	501
108	F	valid	paired	CR	844	0.606		914	501
108	F	invalid	paired	0	0		0	0	501
108	F	valid	paired	CR	787	0.433		909	501
108	F	valid	paired	CR	750	0.508		830	501
108	F	valid	cs only	CR	824	0.836		983	501
108	F	valid	cs only	CR	830	0.532		884	501
108	F	valid	paired	CR	894	0.617		963	501
108	F	valid	cs only	CR	851	0.571		915	501
108	F	valid	cs only	CR	777	0.758		857	501
108	F	invalid	paired	0	0		0	0	501
108	F	valid	paired	no CR	0		0	0	501
108	F	valid	cs only	CR	853	0.875		1181	501
108	F	valid	cs only	CR	808	0.744		1237	501
108	F	valid	paired	CR	510	0.658		877	501
108	F	valid	cs only	CR	996	0.643		1078	501
108	F	valid	paired	CR	625	0.621		826	501
108	F	valid	cs only	CR	855	-0.149		1025	501
108	F	invalid	paired	0	0		0	0	501
108	F	valid	cs only	CR	791	0.592		831	501
108	F	invalid	paired	0	0		0	0	501
108	F	valid	cs only	CR	844	0.623		897	501
108	F	valid	cs only	CR	854	0.682		906	501
108	F	valid	paired	CR	724	0.711		866	501
108	F	invalid	cs only	0	0		0	0	501

108	F	invalid	cs only	0	0		0	0	501
108	F	valid	cs only	CR	760	1.131		935	501
108	F	invalid	paired	no CR	0		0	0	501
108	F	invalid	paired	no CR	0		0	0	501
108	F	valid	paired	CR	768	0.775		915	501
108	F	valid	paired	CR	566	0.782		908	501
108	F	invalid	cs only	0	0		0	0	501
108	F	valid	cs only	CR	840	0.583		930	501
108	F	invalid	cs only	0	0		0	0	501
108	F	valid	cs only	CR	576	0.599		863	501
108	F	valid	us only	no CR	0		0	0	501
83	M	valid	us only	no CR	0		0	0	501
83	M	valid	us only	no CR	0		0	0	501
83	M	valid	cs only	no CR	0		0	0	501
83	M	valid	paired	no CR	0		0	0	501
83	M	valid	paired	no CR	0		0	0	501
83	M	valid	paired	no CR	0		0	0	501
83	M	valid	paired	no CR	0		0	0	501
83	M	valid	paired	no CR	0		0	0	501
83	M	valid	paired	no CR	0		0	0	501
83	M	valid	paired	no CR	0		0	0	501
83	M	valid	paired	no CR	0		0	0	501
83	M	valid	paired	no CR	0		0	0	501
83	M	invalid	paired	0	0		0	0	501
83	M	valid	paired	no CR	0		0	0	501
83	M	valid	paired	no CR	0		0	0	501
83	M	valid	paired	no CR	0		0	0	501
83	M	valid	paired	no CR	0		0	0	501
83	M	valid	paired	no CR	0		0	0	501
83	M	valid	paired	no CR	0		0	0	501
83	M	valid	paired	CR	560	0.981		660	501
83	M	valid	paired	no CR	0		0	0	501
83	M	valid	paired	no CR	0		0	0	501
83	M	valid	paired	no CR	0		0	0	501
83	M	invalid	paired	0	0		0	0	501
83	M	valid	paired	no CR	0		0	0	501
83	M	invalid	paired	0	0		0	0	501
83	M	valid	paired	no CR	0		0	0	501
83	M	valid	paired	no CR	0		0	0	501
83	M	valid	paired	no CR	0		0	0	501
83	M	valid	paired	no CR	0		0	0	501
83	M	valid	paired	no CR	0		0	0	501
83	M	valid	paired	no CR	0		0	0	501
83	M	valid	paired	no CR	0		0	0	501
83	M	invalid	paired	0	0		0	0	501
83	M	invalid	paired	0	0		0	0	501
83	M	valid	paired	no CR	0		0	0	501
83	M	valid	paired	CR	855	0.728		955	501
83	M	valid	paired	no CR	0		0	0	501
83	M	valid	paired	no CR	0		0	0	501

83	M	valid	paired	no CR	0		0	0	501
83	M	valid	paired	no CR	0		0	0	501
83	M	valid	paired	no CR	0		0	0	501
83	M	valid	paired	no CR	0		0	0	501
83	M	valid	paired	no CR	0		0	0	501
83	M	valid	paired	no CR	0		0	0	501
83	M	valid	paired	no CR	0		0	0	501
83	M	valid	paired	no CR	0		0	0	501
83	M	valid	paired	no CR	0		0	0	501
83	M	valid	paired	no CR	0		0	0	501
83	M	valid	cs only	no CR	0		0	0	501
83	M	valid	paired	no CR	0		0	0	501
83	M	valid	paired	no CR	0		0	0	501
83	M	valid	paired	no CR	0		0	0	501
83	M	valid	paired	no CR	0		0	0	501
83	M	valid	paired	CR	591	0.108		791	501
83	M	valid	paired	no CR	0		0	0	501
83	M	valid	paired	no CR	0		0	0	501
83	M	valid	paired	no CR	0		0	0	501
83	M	valid	paired	no CR	0		0	0	501
83	M	valid	cs only	CR	855	0.632		1450	501
83	M	valid	paired	no CR	0		0	0	501
83	M	valid	paired	CR	750	0.249		1017	501
83	M	valid	paired	no CR	0		0	0	501
83	M	valid	paired	no CR	0		0	0	501
83	M	valid	paired	no CR	0		0	0	501
83	M	valid	paired	no CR	0		0	0	501
83	M	valid	paired	no CR	0		0	0	501
83	M	valid	paired	no CR	0		0	0	501
83	M	valid	paired	no CR	0		0	0	501
83	M	valid	cs only	no CR	0		0	0	501
83	M	valid	cs only	no CR	0		0	0	501
83	M	valid	paired	CR	834	0.351		1024	501
83	M	invalid	cs only	0	0		0	0	501
83	M	valid	cs only	no CR	0		0	0	501
83	M	valid	paired	no CR	0		0	0	501
83	M	valid	paired	no CR	0		0	0	501
83	M	valid	cs only	CR	540	0.068		1025	501
83	M	valid	cs only	no CR	0		0	0	501
83	M	valid	paired	no CR	0		0	0	501
83	M	valid	cs only	no CR	0		0	0	501
83	M	valid	paired	no CR	0		0	0	501
83	M	invalid	cs only	0	0		0	0	501
83	M	valid	paired	CR	690	1.033		947	501
83	M	valid	cs only	no CR	0		0	0	501
83	M	valid	paired	no CR	0		0	0	501
83	M	valid	cs only	no CR	0		0	0	501
83	M	valid	cs only	no CR	0		0	0	501
83	M	valid	paired	no CR	0		0	0	501
83	M	valid	cs only	CR	930	0.101		1079	501

83	M	valid	cs only	no CR	0		0	0	501
83	M	valid	cs only	no CR	0		0	0	501
83	M	valid	paired	no CR	0		0	0	501
83	M	valid	paired	no CR	0		0	0	501
83	M	valid	paired	no CR	0		0	0	501
83	M	valid	paired	no CR	0		0	0	501
83	M	valid	cs only	no CR	0		0	0	501
83	M	valid	cs only	no CR	0		0	0	501
83	M	valid	cs only	no CR	0		0	0	501
83	M	valid	cs only	no CR	0		0	0	501
83	M	valid	us only	no CR	0		0	0	501
310	F	valid	us only	no CR	0		0	0	501
310	F	valid	cs only	CR	574	0.309		788	501
310	F	valid	paired	CR	790	0.259		880	501
310	F	valid	paired	CR	577	0.216		886	501
310	F	valid	paired	CR	666	0.453		737	501
310	F	valid	paired	CR	668	0.414		933	501
310	F	invalid	paired	0	0		0	0	501
310	F	valid	paired	CR	686	0.812		877	501
310	F	valid	paired	CR	825	0.195		922	501
310	F	valid	paired	CR	630	0.266		676	501
310	F	valid	paired	CR	671	0.164		961	501
310	F	valid	paired	no CR	0		0	0	501
310	F	valid	paired	CR	843	0.65		993	501
310	F	valid	paired	CR	707	0.908		971	501
310	F	valid	paired	CR	903	0.108		989	501
310	F	invalid	paired	0	0		0	0	501
310	F	valid	paired	CR	797	0.309		839	501
310	F	valid	paired	CR	802	0.603		993	501
310	F	invalid	paired	0	0		0	0	501
310	F	valid	paired	CR	825	0.14		997	501
310	F	valid	paired	CR	820	0.782		997	501
310	F	valid	paired	CR	792	0.298		919	501
310	F	valid	paired	CR	805	0.417		978	501
310	F	valid	paired	CR	718	0.453		831	501
310	F	valid	paired	CR	788	0.398		937	501
310	F	valid	paired	CR	835	0.321		924	501
310	F	valid	paired	CR	819	0.341		1005	501
310	F	valid	paired	CR	796	0.183		899	501
310	F	valid	paired	CR	796	0.907		926	501
310	F	invalid	paired	0	0		0	0	501
310	F	valid	paired	CR	581	0.563		986	501
310	F	valid	paired	CR	891	0.402		982	501
310	F	valid	paired	CR	751	0.346		890	501
310	F	valid	paired	CR	825	0.463		961	501
310	F	valid	paired	CR	835	0.435		909	501
310	F	valid	paired	no CR	0		0	0	501
310	F	invalid	paired	0	0		0	0	501
310	F	invalid	paired	0	0		0	0	501
310	F	valid	paired	CR	845	0.856		1010	501

310	F	valid	cs only	CR	815	0.461		991	501
310	F	valid	paired	no CR	0		0	0	501
310	F	valid	paired	CR	826	0.313		950	501
310	F	invalid	paired	0	0		0	0	501
310	F	valid	paired	CR	848	0.32		939	501
310	F	valid	paired	no CR	0		0	0	501
310	F	valid	paired	CR	792	0.578		939	501
310	F	valid	paired	CR	787	0.645		882	501
310	F	valid	paired	CR	823	0.478		1005	501
310	F	valid	paired	CR	875	0.548		967	501
310	F	valid	cs only	CR	820	0.611		1183	501
310	F	valid	cs only	CR	965	0.192		1103	501
310	F	valid	paired	CR	566	0.389		607	501
310	F	valid	cs only	CR	807	0.308		1028	501
310	F	valid	cs only	CR	826	0.11		1128	501
310	F	invalid	paired	0	0		0	0	501
310	F	valid	paired	CR	826	0.148		899	501
310	F	valid	cs only	CR	799	0.288		998	501
310	F	valid	cs only	no CR	0		0	0	501
310	F	valid	paired	no CR	0		0	0	501
310	F	valid	cs only	no CR	0		0	0	501
310	F	valid	paired	no CR	0		0	0	501
310	F	valid	cs only	CR	980	0.568		1318	501
310	F	invalid	paired	0	0		0	0	501
310	F	invalid	cs only	0	0		0	0	501
310	F	valid	paired	CR	923	0.172		990	501
310	F	invalid	cs only	0	0		0	0	501
310	F	valid	cs only	no CR	0		0	0	501
310	F	valid	paired	no CR	0		0	0	501
310	F	valid	cs only	CR	694	0.508		898	501
310	F	valid	cs only	no CR	0		0	0	501
310	F	valid	cs only	CR	930	0.171		1061	501
310	F	invalid	paired	0	0		0	0	501
310	F	valid	paired	CR	745	0.819		954	501
310	F	valid	paired	CR	773	0.175		837	501
310	F	invalid	paired	0	0		0	0	501
310	F	valid	cs only	CR	747	0.571		835	501
310	F	invalid	cs only	0	0		0	0	501
310	F	valid	cs only	CR	848	0.137		907	501
310	F	valid	cs only	no CR	0		0	0	501
310	F	invalid	us only	no CR	0		0	0	501
671	F	valid	us only	no CR	0		0	0	501
671	F	valid	cs only	no CR	0		0	0	501
671	F	valid	paired	no CR	0		0	0	501
671	F	invalid	paired	0	0		0	0	501
671	F	valid	paired	no CR	0		0	0	501
671	F	valid	paired	no CR	0		0	0	501
671	F	valid	paired	no CR	0		0	0	501
671	F	valid	paired	no CR	0		0	0	501
671	F	valid	paired	no CR	0		0	0	501

671	F	valid	paired	no CR	0		0	0	501
671	F	valid	paired	no CR	0		0	0	501
671	F	valid	paired	CR	762	0.231		890	501
671	F	valid	paired	no CR	0		0	0	501
671	F	valid	paired	no CR	0		0	0	501
671	F	valid	paired	no CR	0		0	0	501
671	F	valid	paired	CR	772	0.209		930	501
671	F	invalid	paired	0	0		0	0	501
671	F	valid	paired	CR	756	0.247		954	501
671	F	valid	paired	CR	818	0.198		885	501
671	F	valid	paired	CR	709	0.149		875	501
671	F	valid	paired	no CR	0		0	0	501
671	F	valid	paired	CR	863	0.197		1016	501
671	F	invalid	paired	0	0		0	0	501
671	F	valid	paired	CR	839	0.354		983	501
671	F	valid	paired	CR	916	0.116		963	501
671	F	valid	paired	no CR	0		0	0	501
671	F	valid	paired	CR	735	0.426		877	501
671	F	valid	paired	no CR	0		0	0	501
671	F	valid	paired	CR	863	0.193		946	501
671	F	valid	paired	CR	865	0.222		1031	501
671	F	valid	paired	no CR	0		0	0	501
671	F	valid	paired	no CR	0		0	0	501
671	F	valid	paired	no CR	0		0	0	501
671	F	valid	paired	no CR	0		0	0	501
671	F	valid	paired	no CR	0		0	0	501
671	F	valid	paired	no CR	0		0	0	501
671	F	valid	paired	CR	834	0.222		1012	501
671	F	valid	paired	no CR	0		0	0	501
671	F	valid	paired	no CR	0		0	0	501
671	F	valid	cs only	CR	803	0.249		937	501
671	F	valid	paired	CR	840	0.151		906	501
671	F	valid	paired	no CR	0		0	0	501
671	F	valid	paired	CR	783	0.179		848	501
671	F	invalid	paired	0	0		0	0	501
671	F	valid	paired	no CR	0		0	0	501
671	F	valid	paired	no CR	0		0	0	501
671	F	valid	paired	CR	762	0.214		882	501
671	F	invalid	paired	0	0		0	0	501
671	F	valid	paired	CR	862	0.16		974	501
671	F	valid	cs only	CR	906	0.244		954	501
671	F	valid	cs only	no CR	0		0	0	501
671	F	valid	paired	no CR	0		0	0	501
671	F	valid	cs only	no CR	0		0	0	501
671	F	valid	cs only	CR	1084	0.284		1223	501
671	F	valid	paired	no CR	0		0	0	501
671	F	valid	paired	no CR	0		0	0	501
671	F	valid	cs only	no CR	0		0	0	501
671	F	valid	cs only	no CR	0		0	0	501
671	F	valid	paired	no CR	0		0	0	501

671	F	valid	cs only	CR	1133	0.28		1212	501
671	F	valid	paired	CR	908	0.138		966	501
671	F	valid	cs only	CR	975	0.194		1091	501
671	F	valid	paired	no CR	0		0	0	501
671	F	valid	cs only	CR	950	0.379		1066	501
671	F	valid	paired	no CR	0		0	0	501
671	F	valid	cs only	CR	1106	0.216		1288	501
671	F	valid	cs only	no CR	0		0	0	501
671	F	valid	paired	no CR	0		0	0	501
671	F	invalid	cs only	0	0		0	0	501
671	F	valid	cs only	no CR	0		0	0	501
671	F	valid	cs only	no CR	0		0	0	501
671	F	valid	paired	no CR	0		0	0	501
671	F	valid	paired	no CR	0		0	0	501
671	F	invalid	paired	0	0		0	0	501
671	F	valid	paired	CR	922	0.167		1021	501
671	F	valid	cs only	CR	837	0.527		1112	501
671	F	valid	cs only	no CR	0		0	0	501
671	F	valid	cs only	no CR	0		0	0	501
671	F	valid	cs only	no CR	0		0	0	501
671	F	valid	us only	no CR	0		0	0	501
325	M	valid	us only	no CR	0		0	0	501
325	M	valid	cs only	CR	521	0.462		611	501
325	M	valid	paired	no CR	0		0	0	501
325	M	valid	paired	CR	944	0.102		990	501
325	M	valid	paired	CR	887	0.105		973	501
325	M	valid	paired	no CR	0		0	0	501
325	M	valid	paired	no CR	0		0	0	501
325	M	valid	paired	CR	862	0.15		934	501
325	M	valid	paired	CR	965	0.094		1042	501
325	M	valid	paired	CR	852	0.457		1005	501
325	M	valid	paired	CR	772	0.781		1017	501
325	M	valid	paired	CR	967	0.056		1014	501
325	M	valid	paired	CR	920	0.073		993	501
325	M	invalid	paired	0	0		0	0	501
325	M	valid	paired	CR	774	0.234		1012	501
325	M	valid	paired	CR	790	0.112		846	501
325	M	valid	paired	CR	869	0.063		926	501
325	M	valid	paired	CR	814	0.165		932	501
325	M	valid	paired	CR	856	0.469		1001	501
325	M	valid	paired	CR	897	0.782		990	501
325	M	valid	paired	CR	899	0.057		1003	501
325	M	valid	paired	CR	907	0.178		989	501
325	M	valid	paired	CR	939	0.145		1020	501
325	M	valid	paired	CR	812	0.137		984	501
325	M	valid	paired	CR	841	0.468		1012	501
325	M	valid	paired	no CR	0		0	0	501
325	M	valid	paired	CR	937	0.155		1020	501
325	M	valid	paired	CR	868	0.125		927	501
325	M	valid	paired	no CR	0		0	0	501

325	M	valid	paired	CR	946	0.288		1016	501
325	M	valid	paired	CR	901	0.277		1012	501
325	M	valid	paired	CR	767	0.132		869	501
325	M	valid	paired	CR	909	0.269		1001	501
325	M	valid	paired	no CR	0		0	0	501
325	M	valid	paired	CR	818	1.162		1027	501
325	M	valid	paired	no CR	0		0	0	501
325	M	valid	paired	CR	907	1.439		1016	501
325	M	valid	paired	CR	788	1.3		1029	501
325	M	valid	paired	CR	875	0.263		1018	501
325	M	valid	cs only	CR	731	1.115		1084	501
325	M	valid	paired	CR	766	0.91		983	501
325	M	invalid	paired	0	0		0	0	501
325	M	valid	paired	CR	830	0.128		918	501
325	M	valid	paired	no CR	0		0	0	501
325	M	valid	paired	CR	784	1.077		988	501
325	M	valid	paired	CR	788	0.064		848	501
325	M	valid	paired	no CR	0		0	0	501
325	M	valid	paired	CR	810	0.139		972	501
325	M	valid	paired	CR	803	0.1		977	501
325	M	valid	cs only	CR	1079	0.863		1428	501
325	M	valid	cs only	CR	980	0.863		1240	501
325	M	valid	paired	no CR	0		0	0	501
325	M	valid	cs only	CR	812	1.073		1005	501
325	M	valid	cs only	CR	944	0.619		1134	501
325	M	valid	paired	no CR	0		0	0	501
325	M	valid	paired	no CR	0		0	0	501
325	M	valid	cs only	CR	743	0.135		817	501
325	M	valid	cs only	no CR	0		0	0	501
325	M	invalid	paired	0	0		0	0	501
325	M	valid	cs only	CR	948	0.779		1208	501
325	M	invalid	paired	0	0		0	0	501
325	M	valid	cs only	CR	946	0.225		1072	501
325	M	valid	paired	CR	778	0.132		892	501
325	M	valid	cs only	CR	877	0.133		952	501
325	M	valid	paired	CR	950	0.084		980	501
325	M	valid	cs only	CR	1025	0.064		1114	501
325	M	valid	cs only	CR	1057	0.075		1199	501
325	M	invalid	paired	0	0		0	0	501
325	M	valid	cs only	CR	594	0.147		809	501
325	M	valid	cs only	CR	1099	0.089		1248	501
325	M	valid	cs only	no CR	0		0	0	501
325	M	valid	paired	no CR	0		0	0	501
325	M	valid	paired	CR	750	0.049		800	501
325	M	valid	paired	CR	856	0.061		903	501
325	M	valid	paired	no CR	0		0	0	501
325	M	valid	cs only	CR	938	0.162		1031	501
325	M	valid	cs only	CR	1003	0.103		1109	501
325	M	valid	cs only	CR	984	0.123		1133	501
325	M	valid	cs only	CR	916	0.232		1011	501

325	M	valid	us only	no CR	0		0	0	501
397	M	valid	us only	no CR	0		0	0	501
397	M	valid	cs only	no CR	0		0	0	501
397	M	valid	paired	no CR	0		0	0	501
397	M	valid	paired	no CR	0		0	0	501
397	M	valid	paired	no CR	0		0	0	501
397	M	valid	paired	no CR	0		0	0	501
397	M	valid	paired	no CR	0		0	0	501
397	M	valid	paired	no CR	0		0	0	501
397	M	valid	paired	CR	860	0.042		907	501
397	M	valid	paired	no CR	0		0	0	501
397	M	valid	paired	no CR	0		0	0	501
397	M	valid	paired	CR	896	0.118		1007	501
397	M	valid	paired	no CR	0		0	0	501
397	M	invalid	paired	0	0		0	0	501
397	M	valid	paired	no CR	0		0	0	501
397	M	valid	paired	CR	800	0.102		863	501
397	M	valid	paired	CR	946	0.136		1015	501
397	M	valid	paired	no CR	0		0	0	501
397	M	valid	paired	no CR	0		0	0	501
397	M	valid	paired	no CR	0		0	0	501
397	M	valid	paired	CR	948	0.03		1012	501
397	M	valid	paired	CR	927	0.17		1005	501
397	M	valid	paired	CR	875	0.048		922	501
397	M	valid	paired	no CR	0		0	0	501
397	M	valid	paired	CR	880	0.032		937	501
397	M	valid	paired	no CR	0		0	0	501
397	M	invalid	paired	0	0		0	0	501
397	M	valid	paired	CR	899	0.109		974	501
397	M	valid	paired	CR	750	0.069		858	501
397	M	valid	paired	CR	871	0.056		958	501
397	M	valid	paired	no CR	0		0	0	501
397	M	valid	paired	CR	841	0.077		980	501
397	M	valid	paired	CR	888	0.043		944	501
397	M	valid	paired	CR	956	0.039		997	501
397	M	invalid	paired	0	0		0	0	501
397	M	invalid	paired	0	0		0	0	501
397	M	valid	paired	CR	935	0.055		993	501
397	M	valid	paired	no CR	0		0	0	501
397	M	valid	paired	CR	946	0.045		1008	501
397	M	valid	cs only	no CR	0		0	0	501
397	M	invalid	paired	no CR	0		0	0	501
397	M	invalid	paired	no CR	0		0	0	501
397	M	invalid	paired	no CR	0		0	0	501
397	M	valid	paired	no CR	0		0	0	501
397	M	valid	paired	CR	924	0.027		990	501
397	M	valid	paired	no CR	0		0	0	501
397	M	valid	paired	CR	894	0.013		924	501
397	M	valid	paired	CR	862	0.072		965	501
397	M	valid	paired	CR	858	0.126		1010	501

397	M	valid	cs only	CR	1037	0.076		1114	501
397	M	valid	cs only	CR	1084	0.058		1202	501
397	M	valid	paired	no CR	0		0	0	501
397	M	valid	cs only	CR	760	0.086		1031	501
397	M	valid	cs only	CR	897	0.048		948	501
397	M	valid	paired	no CR	0		0	0	501
397	M	valid	paired	no CR	0		0	0	501
397	M	valid	cs only	no CR	0		0	0	501
397	M	valid	cs only	CR	855	0.026		931	501
397	M	valid	paired	CR	835	0.06		892	501
397	M	valid	cs only	no CR	0		0	0	501
397	M	invalid	paired	0	0		0	0	501
397	M	valid	cs only	no CR	0		0	0	501
397	M	valid	paired	no CR	0		0	0	501
397	M	valid	cs only	no CR	0		0	0	501
397	M	valid	paired	no CR	0		0	0	501
397	M	invalid	cs only	0	0		0	0	501
397	M	valid	cs only	CR	1001	0.024		1082	501
397	M	valid	paired	CR	554	0.157		705	501
397	M	valid	cs only	no CR	0		0	0	501
397	M	valid	cs only	no CR	0		0	0	501
397	M	valid	cs only	no CR	0		0	0	501
397	M	valid	paired	no CR	0		0	0	501
397	M	valid	paired	no CR	0		0	0	501
397	M	valid	paired	CR	973	0.022		1005	501
397	M	valid	paired	no CR	0		0	0	501
397	M	valid	cs only	no CR	0		0	0	501
397	M	valid	cs only	no CR	0		0	0	501
397	M	valid	cs only	no CR	0		0	0	501
397	M	valid	cs only	CR	855	0.032		952	501
397	M	valid	us only	no CR	0		0	0	501
285	M	valid	us only	no CR	0		0	0	501
285	M	valid	cs only	no CR	0		0	0	501
285	M	valid	paired	no CR	0		0	0	501
285	M	invalid	paired	0	0		0	0	501
285	M	valid	paired	no CR	0		0	0	501
285	M	invalid	paired	0	0		0	0	501
285	M	valid	paired	no CR	0		0	0	501
285	M	valid	paired	no CR	0		0	0	501
285	M	valid	paired	no CR	0		0	0	501
285	M	valid	paired	CR	901	0.025		973	501
285	M	valid	paired	CR	909	0.052		990	501
285	M	valid	paired	CR	799	0.986		867	501
285	M	valid	paired	CR	568	0.554		595	501
285	M	valid	paired	CR	824	0.056		886	501
285	M	valid	paired	no CR	0		0	0	501
285	M	valid	paired	CR	827	0.516		860	501
285	M	invalid	paired	0	0		0	0	501
285	M	valid	paired	no CR	0		0	0	501
285	M	valid	paired	CR	750	0.284		931	501

285	M	valid	paired	no CR	0		0	0	501
285	M	valid	paired	CR	868	0.109		1003	501
285	M	valid	paired	no CR	0		0	0	501
285	M	valid	paired	CR	828	0.353		988	501
285	M	valid	paired	CR	886	0.126		973	501
285	M	valid	paired	no CR	0		0	0	501
285	M	valid	paired	CR	899	0.157		961	501
285	M	valid	paired	CR	831	0.295		980	501
285	M	valid	paired	no CR	0		0	0	501
285	M	valid	paired	CR	688	0.224		984	501
285	M	valid	paired	no CR	0		0	0	501
285	M	valid	paired	no CR	0		0	0	501
285	M	valid	paired	no CR	0		0	0	501
285	M	valid	paired	no CR	0		0	0	501
285	M	invalid	paired	0	0		0	0	501
285	M	valid	paired	no CR	0		0	0	501
285	M	valid	paired	no CR	0		0	0	501
285	M	valid	paired	CR	674	0.124		892	501
285	M	invalid	paired	0	0		0	0	501
285	M	valid	paired	no CR	0		0	0	501
285	M	valid	cs only	CR	1174	0.051		1268	501
285	M	valid	paired	no CR	0		0	0	501
285	M	valid	paired	no CR	0		0	0	501
285	M	valid	paired	no CR	0		0	0	501
285	M	valid	paired	CR	712	0.231		997	501
285	M	valid	paired	CR	916	0.04		1022	501
285	M	invalid	paired	0	0		0	0	501
285	M	valid	paired	CR	865	0.148		1020	501
285	M	valid	paired	CR	899	0.053		937	501
285	M	valid	paired	CR	938	0.102		973	501
285	M	invalid	cs only	0	0		0	0	501
285	M	invalid	cs only	0	0		0	0	501
285	M	invalid	paired	0	0		0	0	501
285	M	valid	cs only	no CR	0		0	0	501
285	M	valid	cs only	no CR	0		0	0	501
285	M	valid	paired	CR	835	0.135		886	501
285	M	valid	paired	CR	571	0.101		624	501
285	M	valid	cs only	CR	732	0.071		781	501
285	M	invalid	cs only	0	0		0	0	501
285	M	valid	paired	no CR	0		0	0	501
285	M	invalid	cs only	0	0		0	0	501
285	M	valid	paired	no CR	0		0	0	501
285	M	valid	cs only	no CR	0		0	0	501
285	M	valid	paired	CR	850	0.165		894	501
285	M	valid	cs only	CR	960	0.125		1077	501
285	M	valid	paired	no CR	0		0	0	501
285	M	valid	cs only	CR	835	0.326		1103	501
285	M	valid	cs only	CR	839	0.561		922	501
285	M	valid	paired	CR	828	0.033		907	501
285	M	invalid	cs only	0	0		0	0	501

285	M	valid	cs only	CR	963	0.198		1132	501
285	M	valid	cs only	no CR	0		0	0	501
285	M	valid	paired	CR	711	0.774		787	501
285	M	valid	paired	CR	865	0.077		986	501
285	M	valid	paired	no CR	0		0	0	501
285	M	valid	paired	CR	833	0.177		898	501
285	M	valid	cs only	CR	933	0.115		964	501
285	M	valid	cs only	CR	809	0.055		854	501
285	M	valid	cs only	CR	939	0.245		1090	501
285	M	valid	cs only	CR	1189	0.707		1431	501
285	M	valid	us only	no CR	0		0	0	501
247	M	valid	us only	no CR	0		0	0	501
247	M	valid	cs only	no CR	0		0	0	501
247	M	valid	paired	no CR	0		0	0	501
247	M	valid	paired	CR	668	0.649		1036	501
247	M	valid	paired	CR	677	0.018		718	501
247	M	valid	paired	CR	593	0.267		836	501
247	M	valid	paired	CR	805	0.364		831	501
247	M	valid	paired	CR	698	0.259		872	501
247	M	valid	paired	no CR	0		0	0	501
247	M	valid	paired	CR	852	0.562		946	501
247	M	valid	paired	CR	909	0.432		1002	501
247	M	invalid	paired	0	0		0	0	501
247	M	valid	paired	no CR	0		0	0	501
247	M	valid	paired	CR	901	0.107		940	501
247	M	valid	paired	CR	865	0.2		904	501
247	M	valid	paired	no CR	0		0	0	501
247	M	valid	paired	no CR	0		0	0	501
247	M	valid	paired	no CR	0		0	0	501
247	M	valid	paired	no CR	0		0	0	501
247	M	valid	paired	CR	933	0.058		1014	501
247	M	valid	paired	CR	822	0.406		934	501
247	M	valid	paired	no CR	0		0	0	501
247	M	valid	paired	CR	749	0.488		953	501
247	M	valid	paired	CR	926	0.363		1010	501
247	M	valid	paired	CR	848	0.61		1012	501
247	M	valid	paired	no CR	0		0	0	501
247	M	valid	paired	CR	941	0.258		996	501
247	M	valid	paired	no CR	0		0	0	501
247	M	valid	paired	CR	815	0.248		848	501
247	M	valid	paired	CR	778	0.228		824	501
247	M	valid	paired	no CR	0		0	0	501
247	M	valid	paired	no CR	0		0	0	501
247	M	valid	paired	CR	941	0.092		1012	501
247	M	invalid	paired	0	0		0	0	501
247	M	valid	paired	CR	820	0.641		912	501
247	M	valid	paired	no CR	0		0	0	501
247	M	valid	paired	CR	787	0.528		969	501
247	M	invalid	paired	0	0		0	0	501
247	M	valid	paired	CR	949	0.194		1002	501

247	M	valid	cs only	CR	1189	0.228		1283	501
247	M	valid	paired	CR	773	0.27		823	501
247	M	valid	paired	no CR	0		0	0	501
247	M	valid	paired	CR	870	0.349		956	501
247	M	valid	paired	CR	912	0.217		932	501
247	M	valid	paired	CR	935	0.089		984	501
247	M	valid	paired	no CR	0		0	0	501
247	M	valid	paired	no CR	0		0	0	501
247	M	valid	paired	CR	824	0.628		1001	501
247	M	valid	paired	CR	826	0.307		941	501
247	M	valid	cs only	CR	664	0.379		1167	501
247	M	valid	cs only	CR	980	0.36		1018	501
247	M	valid	paired	no CR	0		0	0	501
247	M	valid	cs only	CR	855	0.759		1025	501
247	M	valid	cs only	CR	997	0.162		1082	501
247	M	valid	paired	no CR	0		0	0	501
247	M	valid	paired	CR	732	0.192		792	501
247	M	invalid	cs only	0	0		0	0	501
247	M	valid	cs only	CR	973	0.385		1074	501
247	M	valid	paired	no CR	0		0	0	501
247	M	valid	cs only	CR	1076	0.309		1126	501
247	M	valid	paired	CR	912	0.187		997	501
247	M	valid	cs only	CR	581	0.424		677	501
247	M	valid	paired	no CR	0		0	0	501
247	M	valid	cs only	CR	855	0.37		1044	501
247	M	valid	paired	no CR	0		0	0	501
247	M	valid	cs only	CR	777	0.284		1110	501
247	M	valid	cs only	CR	1165	1.06		1300	501
247	M	valid	paired	no CR	0		0	0	501
247	M	valid	cs only	CR	855	0.167		924	501
247	M	valid	cs only	CR	935	0.24		1025	501
247	M	valid	cs only	CR	534	1.444		622	501
247	M	valid	paired	no CR	0		0	0	501
247	M	valid	paired	CR	630	0.523		942	501
247	M	invalid	paired	0	0		0	0	501
247	M	valid	paired	CR	914	0.094		967	501
247	M	valid	cs only	CR	596	0.563		1084	501
247	M	valid	cs only	CR	855	0.525		986	501
247	M	invalid	cs only	0	0		0	0	501
247	M	invalid	cs only	0	0		0	0	501
247	M	valid	us only	no CR	0		0	0	501
268	M	valid	us only	no CR	0		0	0	501
268	M	valid	cs only	no CR	0		0	0	501
268	M	valid	paired	no CR	0		0	0	501
268	M	valid	paired	no CR	0		0	0	501
268	M	valid	paired	no CR	0		0	0	501
268	M	valid	paired	no CR	0		0	0	501
268	M	valid	paired	no CR	0		0	0	501
268	M	valid	paired	CR	839	0.068		1008	501
268	M	valid	paired	CR	803	0.219		988	501

268	M	valid	paired	CR	711	0.205		1011	501
268	M	valid	paired	CR	750	0.092		909	501
268	M	valid	paired	CR	816	0.05		929	501
268	M	valid	paired	no CR	0		0	0	501
268	M	valid	paired	CR	545	0.204		984	501
268	M	valid	paired	CR	728	0.149		1000	501
268	M	valid	paired	CR	901	0.024		976	501
268	M	valid	paired	CR	771	0.096		1010	501
268	M	valid	paired	CR	773	0.072		976	501
268	M	valid	paired	CR	558	0.185		1006	501
268	M	valid	paired	CR	899	0.422		1012	501
268	M	valid	paired	no CR	0		0	0	501
268	M	valid	paired	CR	709	0.038		758	501
268	M	valid	paired	CR	831	0.116		873	501
268	M	valid	paired	no CR	0		0	0	501
268	M	valid	paired	CR	845	0.183		1009	501
268	M	valid	paired	CR	918	0.083		997	501
268	M	valid	paired	CR	684	0.14		830	501
268	M	valid	paired	CR	869	0.076		999	501
268	M	valid	paired	no CR	0		0	0	501
268	M	valid	paired	CR	807	0.602		975	501
268	M	valid	paired	no CR	0		0	0	501
268	M	valid	paired	no CR	0		0	0	501
268	M	valid	paired	no CR	0		0	0	501
268	M	valid	paired	CR	679	0.339		1018	501
268	M	valid	paired	no CR	0		0	0	501
268	M	valid	paired	CR	924	0.095		1005	501
268	M	valid	paired	CR	713	0.171		938	501
268	M	valid	paired	CR	818	0.319		1022	501
268	M	valid	paired	CR	683	0.539		1022	501
268	M	valid	cs only	CR	579	0.143		1121	501
268	M	valid	paired	CR	534	0.102		955	501
268	M	valid	paired	CR	856	0.119		923	501
268	M	valid	paired	CR	737	0.217		1016	501
268	M	valid	paired	no CR	0		0	0	501
268	M	valid	paired	CR	713	0.193		802	501
268	M	valid	paired	CR	792	0.097		867	501
268	M	valid	paired	CR	750	0.078		800	501
268	M	valid	paired	no CR	0		0	0	501
268	M	valid	paired	CR	899	0.119		986	501
268	M	valid	cs only	CR	675	0.127		799	501
268	M	valid	cs only	CR	995	0.104		1128	501
268	M	valid	paired	no CR	0		0	0	501
268	M	valid	cs only	CR	1075	0.331		1410	501
268	M	valid	cs only	CR	789	0.078		862	501
268	M	valid	paired	CR	788	0.082		880	501
268	M	valid	paired	CR	704	0.102		1002	501
268	M	valid	cs only	CR	788	0.092		1057	501
268	M	valid	cs only	CR	636	0.152		1067	501
268	M	valid	paired	CR	794	0.063		912	501

268	M	valid	cs only	CR	822	0.057		858	501
268	M	valid	paired	CR	593	0.104		1021	501
268	M	valid	cs only	CR	720	0.043		997	501
268	M	valid	paired	no CR	0		0	0	501
268	M	valid	cs only	CR	845	0.133		1089	501
268	M	valid	paired	CR	750	0.05		841	501
268	M	valid	cs only	CR	793	0.868		1150	501
268	M	valid	cs only	CR	855	0.045		1050	501
268	M	valid	paired	CR	801	0.067		856	501
268	M	valid	cs only	CR	728	0.162		1057	501
268	M	valid	cs only	CR	941	0.109		1092	501
268	M	valid	cs only	CR	855	0.079		1116	501
268	M	valid	paired	no CR	0		0	0	501
268	M	valid	paired	no CR	0		0	0	501
268	M	valid	paired	CR	750	0.091		800	501
268	M	valid	paired	CR	750	0.058		818	501
268	M	valid	cs only	CR	718	0.156		1174	501
268	M	valid	cs only	no CR	0		0	0	501
268	M	valid	cs only	no CR	0		0	0	501
268	M	valid	cs only	CR	538	0.094		1014	501
268	M	valid	us only	no CR	0		0	0	501
320	F	valid	us only	no CR	0		0	0	501
320	F	valid	cs only	CR	901	1.177		1053	501
320	F	valid	paired	no CR	0		0	0	501
320	F	valid	paired	CR	786	0.181		818	501
320	F	valid	paired	CR	863	0.068		884	501
320	F	valid	paired	CR	826	1.137		988	501
320	F	valid	paired	no CR	0		0	0	501
320	F	invalid	paired	0	0		0	0	501
320	F	valid	paired	CR	652	0.994		715	501
320	F	valid	paired	no CR	0		0	0	501
320	F	valid	paired	CR	726	0.987		831	501
320	F	valid	paired	CR	851	0.325		1024	501
320	F	valid	paired	no CR	0		0	0	501
320	F	valid	paired	CR	946	0.235		995	501
320	F	valid	paired	CR	882	0.23		1003	501
320	F	valid	paired	CR	952	0.054		1016	501
320	F	valid	paired	CR	828	0.106		989	501
320	F	valid	paired	CR	928	0.699		1008	501
320	F	valid	paired	CR	979	0.151		1013	501
320	F	valid	paired	CR	897	0.245		1029	501
320	F	valid	paired	CR	774	0.952		1012	501
320	F	valid	paired	CR	756	0.143		933	501
320	F	valid	paired	CR	541	0.163		574	501
320	F	valid	paired	CR	798	0.638		931	501
320	F	valid	paired	no CR	0		0	0	501
320	F	valid	paired	CR	860	0.393		951	501
320	F	valid	paired	no CR	0		0	0	501
320	F	valid	paired	CR	894	0.908		1022	501
320	F	valid	paired	no CR	0		0	0	501

320	F	valid	paired	CR	839	0.402		1031	501
320	F	valid	paired	no CR	0		0	0	501
320	F	valid	paired	no CR	0		0	0	501
320	F	valid	paired	CR	640	0.841		698	501
320	F	valid	paired	CR	805	0.222		993	501
320	F	valid	paired	no CR	0		0	0	501
320	F	valid	paired	no CR	0		0	0	501
320	F	valid	paired	CR	873	0.998		1013	501
320	F	valid	paired	CR	603	1.003		919	501
320	F	valid	paired	no CR	0		0	0	501
320	F	valid	cs only	CR	822	0.977		1018	501
320	F	invalid	paired	0	0		0	0	501
320	F	valid	paired	no CR	0		0	0	501
320	F	valid	paired	no CR	0		0	0	501
320	F	valid	paired	CR	551	1.105		701	501
320	F	valid	paired	CR	753	1.027		790	501
320	F	valid	paired	CR	830	0.929		928	501
320	F	invalid	paired	0	0		0	0	501
320	F	valid	paired	CR	776	0.977		869	501
320	F	valid	paired	CR	769	0.901		850	501
320	F	valid	cs only	CR	691	0.869		718	501
320	F	invalid	cs only	0	0		0	0	501
320	F	valid	paired	CR	690	0.979		773	501
320	F	valid	cs only	CR	675	0.972		747	501
320	F	valid	cs only	CR	771	0.877		837	501
320	F	valid	paired	no CR	0		0	0	501
320	F	valid	paired	CR	882	0.98		964	501
320	F	valid	cs only	CR	688	0.936		784	501
320	F	valid	cs only	CR	684	1.035		788	501
320	F	valid	paired	CR	782	0.797		822	501
320	F	valid	cs only	CR	711	0.917		803	501
320	F	valid	paired	CR	809	0.89		852	501
320	F	valid	cs only	CR	703	1.192		966	501
320	F	valid	paired	CR	774	0.91		824	501
320	F	valid	cs only	CR	783	0.897		852	501
320	F	valid	paired	CR	757	0.916		842	501
320	F	valid	cs only	CR	772	0.941		845	501
320	F	valid	cs only	CR	765	0.924		838	501
320	F	valid	paired	CR	775	0.894		853	501
320	F	valid	cs only	CR	735	0.913		882	501
320	F	valid	cs only	CR	700	0.933		852	501
320	F	valid	cs only	CR	844	0.842		900	501
320	F	valid	paired	CR	824	0.909		918	501
320	F	invalid	paired	0	0		0	0	501
320	F	valid	paired	CR	569	0.948		634	501
320	F	valid	paired	CR	735	0.965		819	501
320	F	valid	cs only	CR	802	0.995		971	501
320	F	valid	cs only	CR	658	0.921		777	501
320	F	valid	cs only	CR	657	0.966		875	501
320	F	valid	cs only	CR	740	0.846		777	501

320	F	valid	us only	no CR	0		0	0	501
410	F	valid	us only	no CR	0		0	0	501
410	F	valid	cs only	CR	1165	1.763		1266	501
410	F	valid	paired	no CR	0		0	0	501
410	F	invalid	paired	0	0		0	0	501
410	F	valid	paired	no CR	0		0	0	501
410	F	valid	paired	no CR	0		0	0	501
410	F	valid	paired	no CR	0		0	0	501
410	F	valid	paired	no CR	0		0	0	501
410	F	valid	paired	no CR	0		0	0	501
410	F	valid	paired	no CR	0		0	0	501
410	F	valid	paired	no CR	0		0	0	501
410	F	valid	paired	no CR	0		0	0	501
410	F	valid	paired	no CR	0		0	0	501
410	F	invalid	paired	0	0		0	0	501
410	F	valid	paired	CR	617	0.604		773	501
410	F	valid	paired	CR	528	1.477		586	501
410	F	valid	paired	no CR	0		0	0	501
410	F	valid	paired	no CR	0		0	0	501
410	F	valid	paired	no CR	0		0	0	501
410	F	valid	paired	no CR	0		0	0	501
410	F	valid	paired	no CR	0		0	0	501
410	F	valid	paired	no CR	0		0	0	501
410	F	valid	paired	no CR	0		0	0	501
410	F	valid	paired	no CR	0		0	0	501
410	F	valid	paired	no CR	0		0	0	501
410	F	valid	paired	no CR	0		0	0	501
410	F	valid	paired	CR	811	0.092		843	501
410	F	invalid	paired	0	0		0	0	501
410	F	valid	paired	no CR	0		0	0	501
410	F	valid	paired	CR	865	0.055		1018	501
410	F	valid	paired	no CR	0		0	0	501
410	F	valid	paired	no CR	0		0	0	501
410	F	invalid	paired	0	0		0	0	501
410	F	invalid	paired	0	0		0	0	501
410	F	valid	paired	no CR	0		0	0	501
410	F	valid	paired	no CR	0		0	0	501
410	F	invalid	paired	0	0		0	0	501
410	F	valid	paired	no CR	0		0	0	501
410	F	valid	paired	CR	828	0.09		882	501
410	F	valid	cs only	CR	855	2.058		1082	501
410	F	valid	paired	no CR	0		0	0	501
410	F	valid	paired	CR	903	1.815		1001	501
410	F	valid	paired	CR	901	0.043		986	501
410	F	valid	paired	no CR	0		0	0	501
410	F	invalid	paired	0	0		0	0	501
410	F	valid	paired	no CR	0		0	0	501
410	F	valid	paired	CR	890	0.088		1001	501
410	F	valid	paired	CR	807	0.111		1005	501
410	F	valid	paired	CR	901	0.088		995	501

410	F	valid	cs only	CR	527	0.872		608	501
410	F	valid	cs only	CR	1016	1.468		1093	501
410	F	valid	paired	no CR	0		0	0	501
410	F	invalid	cs only	0	0		0	0	501
410	F	valid	cs only	CR	1189	1.67		1323	501
410	F	valid	paired	CR	517	1.31		589	501
410	F	valid	paired	no CR	0		0	0	501
410	F	valid	cs only	no CR	0		0	0	501
410	F	valid	cs only	no CR	0		0	0	501
410	F	valid	paired	CR	641	0.126		761	501
410	F	valid	cs only	no CR	0		0	0	501
410	F	valid	paired	no CR	0		0	0	501
410	F	valid	cs only	no CR	0		0	0	501
410	F	valid	paired	no CR	0		0	0	501
410	F	valid	cs only	CR	1016	1.517		1242	501
410	F	valid	paired	no CR	0		0	0	501
410	F	valid	cs only	CR	1067	0.954		1117	501
410	F	valid	cs only	no CR	0		0	0	501
410	F	valid	paired	no CR	0		0	0	501
410	F	valid	cs only	CR	799	0.738		948	501
410	F	valid	cs only	CR	1045	1.417		1426	501
410	F	invalid	cs only	0	0		0	0	501
410	F	valid	paired	no CR	0		0	0	501
410	F	valid	paired	CR	671	0.158		735	501
410	F	valid	paired	CR	858	0.115		952	501
410	F	valid	paired	no CR	0		0	0	501
410	F	valid	cs only	no CR	0		0	0	501
410	F	valid	cs only	no CR	0		0	0	501
410	F	valid	cs only	no CR	0		0	0	501
410	F	valid	cs only	no CR	0		0	0	501
410	F	valid	us only	no CR	0		0	0	501
291	F	valid	us only	no CR	0		0	0	501
291	F	invalid	cs only	0	0		0	0	501
291	F	valid	paired	no CR	0		0	0	501
291	F	invalid	paired	0	0		0	0	501
291	F	valid	paired	no CR	0		0	0	501
291	F	valid	paired	CR	583	0.647		639	501
291	F	valid	paired	no CR	0		0	0	501
291	F	valid	paired	CR	700	0.682		757	501
291	F	invalid	paired	0	0		0	0	501
291	F	valid	paired	CR	801	0.795		1025	501
291	F	valid	paired	CR	809	0.225		988	501
291	F	valid	paired	no CR	0		0	0	501
291	F	valid	paired	no CR	0		0	0	501
291	F	valid	paired	no CR	0		0	0	501
291	F	valid	paired	CR	905	0.033		1010	501
291	F	valid	paired	no CR	0		0	0	501
291	F	valid	paired	no CR	0		0	0	501
291	F	valid	paired	CR	826	0.387		1012	501
291	F	valid	paired	CR	903	0.207		1022	501

291	F	valid	paired	CR	809	0.542		960	501
291	F	valid	paired	CR	807	0.303		1033	501
291	F	valid	paired	CR	918	0.087		1025	501
291	F	valid	paired	CR	648	0.723		729	501
291	F	valid	paired	CR	865	0.102		1012	501
291	F	valid	paired	CR	769	0.26		1018	501
291	F	valid	paired	CR	956	0.087		1027	501
291	F	valid	paired	CR	899	0.571		1031	501
291	F	valid	paired	CR	956	0.057		1022	501
291	F	valid	paired	CR	969	0.057		1012	501
291	F	valid	paired	CR	848	0.578		990	501
291	F	valid	paired	CR	858	0.116		1012	501
291	F	valid	paired	no CR	0		0	0	501
291	F	valid	paired	CR	841	0.588		1012	501
291	F	valid	paired	no CR	0		0	0	501
291	F	valid	paired	CR	882	0.494		1031	501
291	F	valid	paired	CR	888	0.355		1012	501
291	F	valid	paired	CR	865	0.421		1018	501
291	F	valid	paired	no CR	0		0	0	501
291	F	valid	paired	CR	894	0.123		993	501
291	F	valid	cs only	CR	830	0.671		1106	501
291	F	valid	paired	CR	664	0.114		1005	501
291	F	valid	paired	no CR	0		0	0	501
291	F	valid	paired	CR	831	0.287		1025	501
291	F	valid	paired	no CR	0		0	0	501
291	F	valid	paired	CR	844	0.317		959	501
291	F	valid	paired	no CR	0		0	0	501
291	F	valid	paired	no CR	0		0	0	501
291	F	invalid	paired	0	0		0	0	501
291	F	valid	paired	CR	831	0.028		907	501
291	F	valid	cs only	CR	749	0.371		1096	501
291	F	valid	cs only	CR	521	0.686		642	501
291	F	valid	paired	no CR	0		0	0	501
291	F	valid	cs only	CR	944	0.034		1057	501
291	F	valid	cs only	CR	935	0.092		1093	501
291	F	invalid	paired	0	0		0	0	501
291	F	valid	paired	no CR	0		0	0	501
291	F	valid	cs only	CR	532	0.819		625	501
291	F	valid	cs only	no CR	0		0	0	501
291	F	valid	paired	no CR	0		0	0	501
291	F	valid	cs only	CR	855	0.055		1057	501
291	F	valid	paired	CR	944	0.052		1016	501
291	F	valid	cs only	no CR	0		0	0	501
291	F	valid	paired	CR	628	0.07		784	501
291	F	valid	cs only	CR	575	0.284		991	501
291	F	valid	paired	CR	813	0.163		891	501
291	F	valid	cs only	CR	878	0.134		1038	501
291	F	valid	cs only	CR	678	0.683		760	501
291	F	valid	paired	no CR	0		0	0	501
291	F	valid	cs only	CR	564	0.562		658	501

291	F	valid	cs only	CR	855	0.064	941	501
291	F	valid	cs only	CR	855	0.099	926	501
291	F	valid	paired	no CR	0	0	0	501
291	F	valid	paired	CR	882	0.081	986	501
291	F	valid	paired	no CR	0	0	0	501
291	F	valid	paired	CR	890	0.049	1008	501
291	F	valid	cs only	CR	1072	0.157	1214	501
291	F	valid	cs only	CR	876	0.222	1110	501
291	F	valid	cs only	CR	841	0.199	1074	501
291	F	valid	cs only	no CR	0	0	0	501
291	F	valid	us only	no CR	0	0	0	501
263	M	valid	us only	no CR	0	0	0	501
263	M	valid	cs only	no CR	0	0	0	501
263	M	valid	paired	CR	559	0.597	630	501
263	M	valid	paired	no CR	0	0	0	501
263	M	valid	paired	no CR	0	0	0	501
263	M	valid	paired	CR	643	0.446	735	501
263	M	invalid	paired	0	0	0	0	501
263	M	valid	paired	no CR	0	0	0	501
263	M	valid	paired	no CR	0	0	0	501
263	M	valid	paired	no CR	0	0	0	501
263	M	valid	paired	CR	590	0.917	657	501
263	M	invalid	paired	0	0	0	0	501
263	M	valid	paired	CR	807	1.051	915	501
263	M	valid	paired	no CR	0	0	0	501
263	M	valid	paired	CR	959	0.107	999	501
263	M	valid	paired	no CR	0	0	0	501
263	M	valid	paired	CR	913	0.211	955	501
263	M	valid	paired	CR	875	0.216	1017	501
263	M	invalid	paired	0	0	0	0	501
263	M	valid	paired	CR	848	0.141	1020	501
263	M	valid	paired	CR	862	0.715	884	501
263	M	valid	paired	no CR	0	0	0	501
263	M	valid	paired	no CR	0	0	0	501
263	M	valid	paired	CR	823	0.921	916	501
263	M	valid	paired	no CR	0	0	0	501
263	M	valid	paired	no CR	0	0	0	501
263	M	valid	paired	CR	836	0.598	880	501
263	M	valid	paired	CR	826	0.963	980	501
263	M	valid	paired	no CR	0	0	0	501
263	M	valid	paired	CR	939	0.017	995	501
263	M	valid	paired	CR	965	0.098	1003	501
263	M	valid	paired	no CR	0	0	0	501
263	M	valid	paired	CR	937	-0.066	990	501
263	M	valid	paired	CR	967	0.249	1016	501
263	M	valid	paired	CR	942	0.941	1016	501
263	M	valid	paired	CR	971	0.042	1020	501
263	M	valid	paired	CR	973	0.1	1005	501
263	M	valid	paired	CR	871	0.24	994	501
263	M	valid	paired	no CR	0	0	0	501

263	M	invalid	cs only	0	0		0	0	501
263	M	valid	paired	no CR	0		0	0	501
263	M	valid	paired	no CR	0		0	0	501
263	M	valid	paired	no CR	0		0	0	501
263	M	valid	paired	CR	811	0.104		982	501
263	M	valid	paired	no CR	0		0	0	501
263	M	valid	paired	CR	862	0.651		923	501
263	M	valid	paired	no CR	0		0	0	501
263	M	valid	paired	CR	834	0.892		986	501
263	M	valid	paired	CR	824	0.854		997	501
263	M	valid	cs only	CR	919	1.017		1042	501
263	M	valid	cs only	CR	1079	0.932		1152	501
263	M	valid	paired	CR	881	1.043		982	501
263	M	invalid	cs only	0	0		0	0	501
263	M	valid	cs only	CR	1064	0.952		1129	501
263	M	valid	paired	no CR	0		0	0	501
263	M	valid	paired	no CR	0		0	0	501
263	M	valid	cs only	CR	758	0.912		1063	501
263	M	valid	cs only	CR	973	0.892		1074	501
263	M	valid	paired	no CR	0		0	0	501
263	M	valid	cs only	CR	607	0.957		901	501
263	M	valid	paired	CR	807	1.011		914	501
263	M	valid	cs only	CR	825	0.939		888	501
263	M	invalid	paired	0	0		0	0	501
263	M	valid	cs only	CR	878	0.929		976	501
263	M	valid	paired	CR	687	1.078		911	501
263	M	valid	cs only	CR	809	1.531		909	501
263	M	valid	cs only	CR	957	1.002		1008	501
263	M	valid	paired	CR	809	1.022		873	501
263	M	valid	cs only	CR	866	1.044		899	501
263	M	valid	cs only	CR	880	0.992		916	501
263	M	valid	cs only	CR	803	1.024		869	501
263	M	valid	paired	CR	776	0.919		843	501
263	M	valid	paired	CR	940	0.979		1010	501
263	M	valid	paired	CR	858	1.065		956	501
263	M	valid	paired	CR	933	1.108		975	501
263	M	valid	cs only	CR	758	1.017		826	501
263	M	valid	cs only	CR	773	0.979		877	501
263	M	valid	cs only	CR	901	0.951		999	501
263	M	valid	cs only	CR	840	0.872		888	501
263	M	valid	us only	no CR	0		0	0	501
290	F	valid	us only	no CR	0		0	0	501
290	F	valid	cs only	no CR	0		0	0	501
290	F	invalid	paired	0	0		0	0	501
290	F	valid	paired	no CR	0		0	0	501
290	F	valid	paired	no CR	0		0	0	501
290	F	valid	paired	no CR	0		0	0	501
290	F	valid	paired	CR	969	0.245		1014	501
290	F	valid	paired	CR	830	0.196		881	501
290	F	valid	paired	CR	779	0.015		1014	501

290	F	invalid	paired	0	0		0	0	501
290	F	valid	paired	no CR	0		0	0	501
290	F	valid	paired	CR	912	0.132		1012	501
290	F	valid	paired	CR	950	0.046		1008	501
290	F	valid	paired	no CR	0		0	0	501
290	F	valid	paired	CR	980	0.128		1022	501
290	F	valid	paired	CR	613	0.13		677	501
290	F	valid	paired	CR	958	0.004		995	501
290	F	valid	paired	CR	594	0.396		669	501
290	F	valid	paired	no CR	0		0	0	501
290	F	invalid	paired	0	0		0	0	501
290	F	invalid	paired	0	0		0	0	501
290	F	valid	paired	CR	629	0.107		1033	501
290	F	valid	paired	CR	886	0.074		952	501
290	F	valid	paired	CR	985	0.173		1024	501
290	F	valid	paired	CR	950	0.117		1022	501
290	F	valid	paired	CR	931	0.018		1001	501
290	F	valid	paired	CR	952	0.069		1025	501
290	F	valid	paired	CR	765	0.12		809	501
290	F	valid	paired	CR	897	0.22		951	501
290	F	valid	paired	no CR	0		0	0	501
290	F	valid	paired	CR	848	0.393		1024	501
290	F	invalid	paired	0	0		0	0	501
290	F	valid	paired	CR	583	0.368		1022	501
290	F	valid	paired	CR	903	0.043		952	501
290	F	valid	paired	no CR	0		0	0	501
290	F	valid	paired	no CR	0		0	0	501
290	F	valid	paired	no CR	0		0	0	501
290	F	valid	paired	CR	971	0.19		1016	501
290	F	valid	paired	CR	897	0.273		945	501
290	F	valid	cs only	CR	862	0.375		1043	501
290	F	valid	paired	no CR	0		0	0	501
290	F	invalid	paired	0	0		0	0	501
290	F	valid	paired	no CR	0		0	0	501
290	F	valid	paired	CR	773	0.242		877	501
290	F	invalid	paired	0	0		0	0	501
290	F	valid	paired	CR	781	0.179		946	501
290	F	invalid	paired	0	0		0	0	501
290	F	valid	paired	CR	636	0.269		963	501
290	F	valid	paired	no CR	0		0	0	501
290	F	valid	cs only	CR	855	-0.003		912	501
290	F	valid	cs only	CR	1027	0.083		1071	501
290	F	valid	paired	no CR	0		0	0	501
290	F	valid	cs only	CR	905	0.294		999	501
290	F	valid	cs only	CR	1071	0.475		1212	501
290	F	valid	paired	no CR	0		0	0	501
290	F	valid	paired	no CR	0		0	0	501
290	F	valid	cs only	CR	1005	0.199		1059	501
290	F	valid	cs only	CR	1130	0.136		1154	501
290	F	valid	paired	no CR	0		0	0	501

290	F	valid	cs only	CR	980	0.083		1012	501
290	F	valid	paired	no CR	0		0	0	501
290	F	invalid	cs only	0	0		0	0	501
290	F	valid	paired	CR	956	-0.013		984	501
290	F	valid	cs only	CR	1005	0.173		1099	501
290	F	valid	paired	CR	703	0.151		785	501
290	F	valid	cs only	CR	1027	0.341		1145	501
290	F	valid	cs only	CR	965	0.161		1095	501
290	F	valid	paired	CR	944	0.136		990	501
290	F	invalid	cs only	0	0		0	0	501
290	F	valid	cs only	CR	899	0.091		969	501
290	F	invalid	cs only	no CR	0		0	0	501
290	F	invalid	paired	0	0		0	0	501
290	F	invalid	paired	0	0		0	0	501
290	F	valid	paired	CR	957	0.152		977	501
290	F	valid	paired	CR	901	0.136		941	501
290	F	valid	cs only	CR	1043	0.107		1059	501
290	F	invalid	cs only	0	0		0	0	501
290	F	invalid	cs only	0	0		0	0	501
290	F	valid	cs only	CR	993	0.191		1065	501
290	F	valid	us only	no CR	0		0	0	501
532	F	invalid	us only	0	0		0	0	501
532	F	invalid	cs only	0	0		0	0	501
532	F	valid	paired	no CR	0		0	0	501
532	F	invalid	paired	0	0		0	0	501
532	F	valid	paired	CR	961	0.053		1012	501
532	F	valid	paired	CR	654	0.782		901	501
532	F	invalid	paired	0	0		0	0	501
532	F	valid	paired	CR	796	0.629		916	501
532	F	valid	paired	CR	794	0.649		1013	501
532	F	valid	paired	CR	705	0.084		976	501
532	F	invalid	paired	0	0		0	0	501
532	F	invalid	paired	0	0		0	0	501
532	F	valid	paired	CR	746	0.657		901	501
532	F	invalid	paired	0	0		0	0	501
532	F	valid	paired	CR	833	0.351		878	501
532	F	valid	paired	CR	861	0.308		963	501
532	F	valid	paired	CR	771	1.065		836	501
532	F	invalid	paired	0	0		0	0	501
532	F	valid	paired	CR	728	0.225		1007	501
532	F	valid	paired	CR	826	0.328		1014	501
532	F	valid	paired	CR	691	0.856		942	501
532	F	valid	paired	no CR	0		0	0	501
532	F	valid	paired	CR	707	0.814		755	501
532	F	invalid	paired	0	0		0	0	501
532	F	valid	paired	CR	813	0.768		970	501
532	F	invalid	paired	0	0		0	0	501
532	F	valid	paired	no CR	0		0	0	501
532	F	valid	paired	CR	685	0.742		896	501
532	F	valid	paired	CR	719	0.683		1008	501

532	F	valid	paired	CR	877	0.581		1010	501
532	F	valid	paired	CR	730	0.744		932	501
532	F	valid	paired	CR	820	0.728		912	501
532	F	invalid	paired	0	0		0	0	501
532	F	valid	paired	no CR	0		0	0	501
532	F	invalid	paired	0	0		0	0	501
532	F	valid	paired	CR	931	0.123		1022	501
532	F	valid	paired	CR	955	0.809		1012	501
532	F	valid	paired	CR	720	0.648		946	501
532	F	invalid	paired	0	0		0	0	501
532	F	invalid	cs only	0	0		0	0	501
532	F	valid	paired	CR	543	0.975		986	501
532	F	valid	paired	CR	755	0.629		934	501
532	F	valid	paired	CR	796	0.648		1031	501
532	F	valid	paired	no CR	0		0	0	501
532	F	valid	paired	CR	687	0.76		1027	501
532	F	valid	paired	CR	735	0.82		1021	501
532	F	valid	paired	CR	536	0.755		963	501
532	F	valid	paired	CR	811	0.961		963	501
532	F	valid	paired	CR	764	0.732		955	501
532	F	valid	cs only	CR	795	0.927		870	501
532	F	valid	cs only	CR	784	0.677		944	501
532	F	valid	paired	CR	839	0.595		969	501
532	F	valid	cs only	CR	611	0.976		898	501
532	F	valid	cs only	CR	748	0.74		1156	501
532	F	valid	paired	CR	818	0.144		978	501
532	F	valid	paired	CR	806	0.639		1003	501
532	F	valid	cs only	CR	839	0.678		995	501
532	F	invalid	cs only	0	0		0	0	501
532	F	valid	paired	CR	769	0.539		921	501
532	F	valid	cs only	CR	1013	0.841		1109	501
532	F	invalid	paired	0	0		0	0	501
532	F	invalid	cs only	0	0		0	0	501
532	F	valid	paired	CR	865	0.238		925	501
532	F	valid	cs only	CR	941	0.573		1004	501
532	F	invalid	paired	0	0		0	0	501
532	F	invalid	cs only	0	0		0	0	501
532	F	valid	cs only	CR	894	0.464		961	501
532	F	invalid	paired	0	0		0	0	501
532	F	valid	cs only	CR	764	0.602		841	501
532	F	invalid	cs only	0	0		0	0	501
532	F	invalid	cs only	0	0		0	0	501
532	F	valid	paired	no CR	0		0	0	501
532	F	valid	paired	CR	873	0.978		945	501
532	F	invalid	paired	0	0		0	0	501
532	F	invalid	paired	0	0		0	0	501
532	F	valid	cs only	CR	823	0.8		938	501
532	F	valid	cs only	CR	927	0.675		1046	501
532	F	invalid	cs only	0	0		0	0	501
532	F	valid	cs only	CR	601	0.474		1444	501

532	F	valid	us only	no CR	0		0	0	501
424	F	valid	us only	no CR	0		0	0	501
424	F	valid	cs only	CR	870	0.855		932	501
424	F	valid	paired	CR	899	0.639		986	501
424	F	valid	paired	CR	846	1.039		892	501
424	F	valid	paired	no CR	0		0	0	501
424	F	invalid	paired	0	0		0	0	501
424	F	valid	paired	no CR	0		0	0	501
424	F	valid	paired	CR	750	1.037		790	501
424	F	valid	paired	CR	657	1.001		734	501
424	F	valid	paired	CR	672	0.891		1025	501
424	F	valid	paired	CR	909	0.518		980	501
424	F	valid	paired	CR	839	0.372		880	501
424	F	valid	paired	no CR	0		0	0	501
424	F	valid	paired	CR	699	0.438		741	501
424	F	invalid	paired	0	0		0	0	501
424	F	valid	paired	CR	769	0.679		806	501
424	F	valid	paired	CR	893	0.936		950	501
424	F	valid	paired	CR	958	0.067		1005	501
424	F	invalid	paired	0	0		0	0	501
424	F	valid	paired	no CR	0		0	0	501
424	F	valid	paired	CR	807	0.975		920	501
424	F	valid	paired	CR	884	0.957		961	501
424	F	valid	paired	CR	952	0.395		1029	501
424	F	valid	paired	CR	699	0.61		769	501
424	F	invalid	paired	0	0		0	0	501
424	F	valid	paired	CR	711	0.94		926	501
424	F	valid	paired	CR	870	0.956		967	501
424	F	invalid	paired	0	0		0	0	501
424	F	valid	paired	CR	894	0.841		1042	501
424	F	valid	paired	CR	937	0.966		1030	501
424	F	invalid	paired	0	0		0	0	501
424	F	valid	paired	CR	870	0.818		920	501
424	F	valid	paired	CR	911	0.847		965	501
424	F	valid	paired	CR	856	0.865		1037	501
424	F	invalid	paired	0	0		0	0	501
424	F	valid	paired	no CR	0		0	0	501
424	F	valid	paired	no CR	0		0	0	501
424	F	valid	paired	CR	661	0.834		805	501
424	F	valid	paired	CR	715	0.968		867	501
424	F	invalid	cs only	0	0		0	0	501
424	F	valid	paired	no CR	0		0	0	501
424	F	valid	paired	CR	838	0.983		910	501
424	F	valid	paired	CR	848	0.917		909	501
424	F	valid	paired	CR	791	0.986		896	501
424	F	valid	paired	CR	899	0.948		952	501
424	F	invalid	paired	0	0		0	0	501
424	F	valid	paired	CR	716	0.946		862	501
424	F	valid	paired	CR	737	0.966		875	501
424	F	invalid	paired	0	0		0	0	501

424	F	valid	cs only	CR	833	1.083		945	501
424	F	valid	cs only	CR	894	0.974		967	501
424	F	valid	paired	CR	961	0.79		1042	501
424	F	invalid	cs only	0	0		0	0	501
424	F	valid	cs only	CR	835	0.975		907	501
424	F	valid	paired	CR	926	0.954		1002	501
424	F	invalid	paired	0	0		0	0	501
424	F	invalid	cs only	0	0		0	0	501
424	F	valid	cs only	CR	657	0.978		837	501
424	F	valid	paired	CR	939	0.42		1016	501
424	F	valid	cs only	CR	863	0.96		944	501
424	F	valid	paired	CR	754	0.9		872	501
424	F	valid	cs only	CR	620	0.949		869	501
424	F	invalid	paired	0	0		0	0	501
424	F	valid	cs only	CR	809	0.779		877	501
424	F	valid	paired	CR	709	0.894		771	501
424	F	valid	cs only	CR	637	0.912		796	501
424	F	valid	cs only	CR	711	0.761		809	501
424	F	invalid	paired	0	0		0	0	501
424	F	valid	cs only	CR	730	0.804		818	501
424	F	invalid	cs only	0	0		0	0	501
424	F	invalid	cs only	0	0		0	0	501
424	F	valid	paired	CR	912	0.949		965	501
424	F	valid	paired	CR	752	0.974		849	501
424	F	invalid	paired	0	0		0	0	501
424	F	valid	paired	CR	855	0.878		918	501
424	F	valid	cs only	CR	816	0.971		907	501
424	F	invalid	cs only	0	0		0	0	501
424	F	valid	cs only	CR	828	0.899		892	501
424	F	valid	cs only	CR	869	0.973		935	501
424	F	valid	us only	no CR	0		0	0	501
307	M	valid	us only	no CR	0		0	0	501
307	M	valid	cs only	CR	792	0.777		871	501
307	M	valid	paired	no CR	0		0	0	501
307	M	valid	paired	no CR	0		0	0	501
307	M	valid	paired	no CR	0		0	0	501
307	M	valid	paired	no CR	0		0	0	501
307	M	valid	paired	no CR	0		0	0	501
307	M	valid	paired	no CR	0		0	0	501
307	M	valid	paired	CR	566	0.257		1007	501
307	M	valid	paired	no CR	0		0	0	501
307	M	invalid	paired	0	0		0	0	501
307	M	valid	paired	no CR	0		0	0	501
307	M	valid	paired	no CR	0		0	0	501
307	M	valid	paired	no CR	0		0	0	501
307	M	valid	paired	no CR	0		0	0	501
307	M	valid	paired	no CR	0		0	0	501
307	M	valid	paired	no CR	0		0	0	501
307	M	valid	paired	no CR	0		0	0	501
307	M	valid	paired	no CR	0		0	0	501

307	M	valid	paired	no CR	0		0	0	501
307	M	valid	paired	CR	899	0.161		984	501
307	M	valid	paired	CR	897	0.154		1018	501
307	M	valid	paired	CR	588	0.986		689	501
307	M	valid	paired	no CR	0		0	0	501
307	M	valid	paired	no CR	0		0	0	501
307	M	valid	paired	no CR	0		0	0	501
307	M	valid	paired	no CR	0		0	0	501
307	M	valid	paired	no CR	0		0	0	501
307	M	valid	paired	no CR	0		0	0	501
307	M	valid	paired	no CR	0		0	0	501
307	M	valid	paired	no CR	0		0	0	501
307	M	valid	paired	no CR	0		0	0	501
307	M	valid	paired	CR	828	0.236		892	501
307	M	valid	paired	no CR	0		0	0	501
307	M	valid	paired	CR	973	0.044		1014	501
307	M	valid	paired	no CR	0		0	0	501
307	M	valid	paired	no CR	0		0	0	501
307	M	valid	paired	no CR	0		0	0	501
307	M	invalid	paired	0	0		0	0	501
307	M	valid	cs only	CR	771	0.149		1114	501
307	M	invalid	paired	0	0		0	0	501
307	M	valid	paired	no CR	0		0	0	501
307	M	valid	paired	no CR	0		0	0	501
307	M	invalid	paired	0	0		0	0	501
307	M	valid	paired	no CR	0		0	0	501
307	M	valid	paired	no CR	0		0	0	501
307	M	valid	paired	no CR	0		0	0	501
307	M	valid	paired	no CR	0		0	0	501
307	M	valid	paired	no CR	0		0	0	501
307	M	valid	cs only	no CR	0		0	0	501
307	M	valid	cs only	no CR	0		0	0	501
307	M	valid	paired	no CR	0		0	0	501
307	M	valid	cs only	no CR	0		0	0	501
307	M	valid	cs only	no CR	0		0	0	501
307	M	valid	paired	no CR	0		0	0	501
307	M	valid	paired	no CR	0		0	0	501
307	M	valid	cs only	no CR	0		0	0	501
307	M	valid	cs only	no CR	0		0	0	501
307	M	valid	paired	no CR	0		0	0	501
307	M	valid	cs only	no CR	0		0	0	501
307	M	valid	paired	no CR	0		0	0	501
307	M	valid	cs only	no CR	0		0	0	501
307	M	valid	paired	CR	643	0.177		1012	501
307	M	valid	cs only	no CR	0		0	0	501
307	M	valid	paired	no CR	0		0	0	501
307	M	valid	cs only	no CR	0		0	0	501
307	M	valid	cs only	no CR	0		0	0	501
307	M	valid	paired	no CR	0		0	0	501
307	M	valid	cs only	no CR	0		0	0	501

307	M	valid	cs only	no CR	0		0	0	501
307	M	valid	cs only	no CR	0		0	0	501
307	M	valid	paired	no CR	0		0	0	501
307	M	valid	paired	no CR	0		0	0	501
307	M	valid	paired	no CR	0		0	0	501
307	M	valid	paired	no CR	0		0	0	501
307	M	valid	cs only	no CR	0		0	0	501
307	M	valid	cs only	no CR	0		0	0	501
307	M	valid	cs only	no CR	0		0	0	501
307	M	valid	cs only	no CR	0		0	0	501
307	M	valid	us only	no CR	0		0	0	501
273	F	valid	us only	no CR	0		0	0	501
273	F	valid	cs only	no CR	0		0	0	501
273	F	valid	paired	no CR	0		0	0	501
273	F	valid	paired	no CR	0		0	0	501
273	F	valid	paired	no CR	0		0	0	501
273	F	valid	paired	no CR	0		0	0	501
273	F	valid	paired	no CR	0		0	0	501
273	F	valid	paired	no CR	0		0	0	501
273	F	valid	paired	no CR	0		0	0	501
273	F	valid	paired	no CR	0		0	0	501
273	F	valid	paired	no CR	0		0	0	501
273	F	valid	paired	no CR	0		0	0	501
273	F	valid	paired	CR	758	0.057		875	501
273	F	valid	paired	no CR	0		0	0	501
273	F	valid	paired	CR	760	0.167		904	501
273	F	valid	paired	no CR	0		0	0	501
273	F	valid	paired	no CR	0		0	0	501
273	F	valid	paired	CR	792	0.203		927	501
273	F	valid	paired	CR	780	0.1		958	501
273	F	valid	paired	no CR	0		0	0	501
273	F	valid	paired	no CR	0		0	0	501
273	F	valid	paired	no CR	0		0	0	501
273	F	valid	paired	CR	837	0.04		926	501
273	F	valid	paired	no CR	0		0	0	501
273	F	valid	paired	no CR	0		0	0	501
273	F	valid	paired	CR	703	0.136		997	501
273	F	valid	paired	CR	750	0.055		800	501
273	F	valid	paired	no CR	0		0	0	501
273	F	valid	paired	no CR	0		0	0	501
273	F	valid	paired	CR	839	0.239		1012	501
273	F	valid	paired	no CR	0		0	0	501
273	F	valid	paired	no CR	0		0	0	501
273	F	valid	paired	no CR	0		0	0	501
273	F	valid	paired	no CR	0		0	0	501
273	F	valid	paired	CR	914	0.092		999	501
273	F	valid	paired	no CR	0		0	0	501
273	F	valid	paired	no CR	0		0	0	501
273	F	valid	paired	CR	801	0.133		972	501
273	F	valid	paired	CR	858	0.106		951	501

273	F	valid	cs only	CR	996	0.181		1136	501
273	F	valid	paired	CR	547	0.163		592	501
273	F	valid	paired	no CR	0		0	0	501
273	F	valid	paired	no CR	0		0	0	501
273	F	valid	paired	no CR	0		0	0	501
273	F	valid	paired	no CR	0		0	0	501
273	F	valid	paired	CR	750	0.047		800	501
273	F	valid	paired	no CR	0		0	0	501
273	F	valid	paired	no CR	0		0	0	501
273	F	valid	paired	CR	909	0.087		952	501
273	F	valid	cs only	CR	971	0.065		1103	501
273	F	valid	cs only	no CR	0		0	0	501
273	F	valid	paired	no CR	0		0	0	501
273	F	valid	cs only	CR	607	0.101		1025	501
273	F	valid	cs only	CR	1025	0.094		1074	501
273	F	valid	paired	no CR	0		0	0	501
273	F	valid	paired	CR	914	0.05		1003	501
273	F	valid	cs only	no CR	0		0	0	501
273	F	valid	cs only	CR	613	0.165		1054	501
273	F	valid	paired	no CR	0		0	0	501
273	F	valid	cs only	CR	628	0.143		728	501
273	F	valid	paired	no CR	0		0	0	501
273	F	valid	cs only	CR	691	0.998		939	501
273	F	valid	paired	CR	777	0.159		923	501
273	F	valid	cs only	CR	758	0.095		1061	501
273	F	valid	paired	no CR	0		0	0	501
273	F	valid	cs only	no CR	0		0	0	501
273	F	valid	cs only	CR	784	0.146		1063	501
273	F	valid	paired	no CR	0		0	0	501
273	F	valid	cs only	no CR	0		0	0	501
273	F	valid	cs only	CR	701	0.084		754	501
273	F	valid	cs only	no CR	0		0	0	501
273	F	valid	paired	no CR	0		0	0	501
273	F	valid	paired	no CR	0		0	0	501
273	F	invalid	paired	0	0		0	0	501
273	F	valid	paired	no CR	0		0	0	501
273	F	valid	cs only	CR	888	0.162		1064	501
273	F	valid	cs only	CR	636	0.132		1069	501
273	F	valid	cs only	CR	638	0.11		1056	501
273	F	valid	cs only	CR	855	0.096		950	501
273	F	valid	us only	no CR	0		0	0	501
259	F	valid	us only	no CR	0		0	0	501
259	F	valid	cs only	CR	572	0.388		632	501
259	F	invalid	paired	0	0		0	0	501
259	F	valid	paired	CR	779	1.474		826	501
259	F	invalid	paired	0	0		0	0	501
259	F	invalid	paired	0	0		0	0	501
259	F	valid	paired	CR	722	1.475		800	501
259	F	valid	paired	CR	713	1.685		937	501
259	F	valid	paired	CR	651	1.153		694	501

259	F	valid	paired	CR		636	1.273		745	501
259	F	valid	paired	CR		692	1.297		719	501
259	F	valid	paired	CR		656	1.198		720	501
259	F	valid	paired	CR		712	1.325		779	501
259	F	valid	paired	CR		723	1.181		759	501
259	F	valid	paired	CR		630	1.098		706	501
259	F	valid	paired	CR		668	1.371		731	501
259	F	valid	paired	CR		751	1.313		827	501
259	F	valid	paired	CR		684	1.227		722	501
259	F	valid	paired	CR		688	1.013		765	501
259	F	valid	paired	CR		711	1.243		769	501
259	F	valid	paired	CR		828	0.998		870	501
259	F	valid	paired	CR		745	1.152		802	501
259	F	valid	paired	CR		884	1.359		940	501
259	F	valid	paired	CR		715	1.068		786	501
259	F	valid	paired	CR		724	1.202		766	501
259	F	valid	paired	CR		615	1.222		684	501
259	F	invalid	paired		0	0		0	0	501
259	F	valid	paired	CR		687	1.083		719	501
259	F	valid	paired	CR		793	1.159		827	501
259	F	valid	paired	CR		691	1.119		712	501
259	F	valid	paired	CR		690	1.237		724	501
259	F	valid	paired	CR		678	1.11		730	501
259	F	valid	paired	CR		665	1.155		692	501
259	F	valid	paired	CR		623	1.262		708	501
259	F	invalid	paired		0	0		0	0	501
259	F	valid	paired	CR		679	1.346		720	501
259	F	valid	paired	CR		687	1.24		723	501
259	F	valid	paired	CR		675	1.177		711	501
259	F	valid	paired	CR		677	1.025		722	501
259	F	invalid	cs only		0	0		0	0	501
259	F	valid	paired	CR		654	1.155		724	501
259	F	valid	paired	CR		696	1.262		738	501
259	F	valid	paired	CR		560	0.993		624	501
259	F	valid	paired	CR		688	1.218		722	501
259	F	valid	paired	CR		698	1.305		764	501
259	F	valid	paired	CR		700	0.947		835	501
259	F	valid	paired	CR		784	1.158		867	501
259	F	valid	paired	CR		655	0.984		696	501
259	F	valid	paired	CR		711	1.07		749	501
259	F	valid	cs only	CR		773	0.957		840	501
259	F	valid	cs only	CR		725	0.962		756	501
259	F	valid	paired	CR		696	1.022		725	501
259	F	valid	cs only	CR		694	1.142		737	501
259	F	valid	cs only	CR		667	1.03		747	501
259	F	invalid	paired		0	0		0	0	501
259	F	valid	paired	CR		692	0.786		739	501
259	F	valid	cs only	CR		799	0.675		858	501
259	F	valid	cs only	CR		617	0.996		715	501
259	F	invalid	paired		0	0		0	0	501

259	F	invalid	cs only	0	0		0	0	501
259	F	valid	paired	CR	607	1.061		893	501
259	F	valid	cs only	CR	541	0.871		683	501
259	F	valid	paired	CR	596	1.018		662	501
259	F	valid	cs only	CR	677	1.066		713	501
259	F	valid	paired	CR	737	1.045		795	501
259	F	valid	cs only	CR	674	0.995		713	501
259	F	valid	cs only	CR	693	1.024		728	501
259	F	invalid	paired	0	0		0	0	501
259	F	valid	cs only	CR	696	1.123		727	501
259	F	valid	cs only	CR	741	0.973		786	501
259	F	valid	cs only	CR	708	1.001		745	501
259	F	valid	paired	CR	705	0.955		799	501
259	F	valid	paired	CR	763	1.073		812	501
259	F	valid	paired	CR	684	1.079		769	501
259	F	valid	paired	CR	737	1.01		826	501
259	F	valid	cs only	CR	746	0.817		781	501
259	F	invalid	cs only	0	0		0	0	501
259	F	invalid	cs only	0	0		0	0	501
259	F	valid	cs only	CR	701	0.978		755	501
259	F	valid	us only	no CR	0		0	0	501
341	F	valid	us only	no CR	0		0	0	501
341	F	valid	cs only	CR	1156	1.178		1375	501
341	F	valid	paired	no CR	0		0	0	501
341	F	valid	paired	CR	807	0.394		916	501
341	F	valid	paired	CR	759	0.116		842	501
341	F	valid	paired	CR	670	0.26		954	501
341	F	valid	paired	CR	560	0.384		860	501
341	F	valid	paired	CR	792	0.288		960	501
341	F	valid	paired	CR	809	0.145		1037	501
341	F	valid	paired	CR	720	0.521		934	501
341	F	valid	paired	CR	718	0.3		854	501
341	F	valid	paired	CR	841	0.36		1005	501
341	F	valid	paired	CR	663	0.724		848	501
341	F	valid	paired	CR	845	0.028		903	501
341	F	valid	paired	CR	824	0.877		962	501
341	F	valid	paired	CR	737	0.544		933	501
341	F	valid	paired	CR	681	1.12		954	501
341	F	valid	paired	CR	754	0.346		965	501
341	F	valid	paired	CR	696	0.815		880	501
341	F	valid	paired	CR	742	0.272		842	501
341	F	valid	paired	CR	555	1.294		948	501
341	F	valid	paired	no CR	0		0	0	501
341	F	valid	paired	CR	862	0.637		911	501
341	F	valid	paired	CR	832	0.188		882	501
341	F	valid	paired	CR	906	0.368		975	501
341	F	valid	paired	CR	765	1.112		1003	501
341	F	valid	paired	CR	760	0.836		1014	501
341	F	valid	paired	no CR	0		0	0	501
341	F	valid	paired	CR	824	0.893		998	501

341	F	valid	paired	CR	679	0.666		970	501
341	F	valid	paired	CR	747	0.184		1006	501
341	F	valid	paired	CR	901	0.249		980	501
341	F	valid	paired	CR	874	0.115		968	501
341	F	valid	paired	CR	875	1.009		952	501
341	F	valid	paired	CR	749	0.411		1008	501
341	F	valid	paired	CR	774	0.32		1001	501
341	F	valid	paired	CR	832	0.263		1011	501
341	F	valid	paired	CR	841	0.618		990	501
341	F	valid	paired	CR	741	0.75		939	501
341	F	valid	cs only	CR	858	0.796		1197	501
341	F	valid	paired	CR	904	0.164		966	501
341	F	valid	paired	CR	951	0.972		1041	501
341	F	valid	paired	no CR	0		0	0	501
341	F	valid	paired	CR	680	0.303		998	501
341	F	valid	paired	CR	749	1.106		980	501
341	F	valid	paired	CR	761	1.063		1005	501
341	F	valid	paired	CR	818	0.854		1010	501
341	F	invalid	paired	0	0		0	0	501
341	F	valid	paired	CR	585	0.565		988	501
341	F	valid	cs only	CR	1157	1.357		1462	501
341	F	valid	cs only	no CR	0		0	0	501
341	F	valid	paired	CR	771	0.425		867	501
341	F	valid	cs only	CR	1150	0.758		1300	501
341	F	valid	cs only	CR	739	1.114		1020	501
341	F	valid	paired	CR	745	0.471		1010	501
341	F	valid	paired	CR	669	0.343		1014	501
341	F	invalid	cs only	0	0		0	0	501
341	F	valid	cs only	CR	688	1.358		1162	501
341	F	invalid	paired	0	0		0	0	501
341	F	valid	cs only	no CR	0		0	0	501
341	F	valid	paired	CR	808	0.802		855	501
341	F	valid	cs only	CR	705	0.879		835	501
341	F	valid	paired	CR	961	0.21		1012	501
341	F	valid	cs only	CR	882	1.043		1098	501
341	F	invalid	paired	0	0		0	0	501
341	F	valid	cs only	CR	864	1.393		1189	501
341	F	valid	cs only	CR	1724	0.833		1425	501
341	F	valid	paired	no CR	0		0	0	501
341	F	valid	cs only	CR	783	1.07		993	501
341	F	valid	cs only	CR	527	0.928		594	501
341	F	invalid	cs only	0	0		0	0	501
341	F	valid	paired	CR	758	0.392		1031	501
341	F	valid	paired	no CR	0		0	0	501
341	F	valid	paired	CR	871	0.453		951	501
341	F	valid	paired	CR	875	0.483		960	501
341	F	invalid	cs only	0	0		0	0	501
341	F	valid	cs only	CR	608	1.009		1130	501
341	F	valid	cs only	CR	918	0.249		1543	501
341	F	valid	cs only	CR	752	1.196		1262	501

341	F	valid	us only	no CR	0		0	0	501
300	F	invalid	us only	0	0		0	0	501
300	F	valid	cs only	CR	793	0.389		935	501
300	F	invalid	paired	0	0		0	0	501
300	F	valid	paired	CR	767	0.428		1018	501
300	F	valid	paired	CR	720	0.914		923	501
300	F	valid	paired	CR	737	0.705		986	501
300	F	valid	paired	CR	660	0.677		967	501
300	F	invalid	paired	0	0		0	0	501
300	F	valid	paired	no CR	0		0	0	501
300	F	valid	paired	CR	787	0.844		1034	501
300	F	valid	paired	CR	779	0.92		1028	501
300	F	valid	paired	CR	679	0.86		869	501
300	F	valid	paired	CR	720	0.953		1008	501
300	F	valid	paired	CR	681	0.977		1019	501
300	F	invalid	paired	0	0		0	0	501
300	F	valid	paired	CR	701	0.86		950	501
300	F	valid	paired	CR	770	0.47		848	501
300	F	invalid	paired	0	0		0	0	501
300	F	valid	paired	CR	831	0.528		1018	501
300	F	valid	paired	CR	711	0.982		980	501
300	F	valid	paired	CR	747	0.962		976	501
300	F	valid	paired	no CR	0		0	0	501
300	F	invalid	paired	0	0		0	0	501
300	F	valid	paired	CR	764	0.96		1016	501
300	F	valid	paired	CR	849	0.448		930	501
300	F	valid	paired	CR	671	0.911		840	501
300	F	valid	paired	CR	792	1.036		999	501
300	F	valid	paired	CR	659	1.02		881	501
300	F	valid	paired	CR	728	0.976		934	501
300	F	valid	paired	CR	760	0.864		975	501
300	F	valid	paired	CR	843	0.853		976	501
300	F	valid	paired	CR	825	0.942		1000	501
300	F	valid	paired	CR	804	0.996		1016	501
300	F	valid	paired	CR	695	0.845		810	501
300	F	valid	paired	CR	742	0.958		997	501
300	F	valid	paired	CR	910	0.308		1012	501
300	F	valid	paired	CR	821	0.984		975	501
300	F	valid	paired	no CR	0		0	0	501
300	F	valid	paired	CR	743	0.949		835	501
300	F	invalid	cs only	0	0		0	0	501
300	F	valid	paired	no CR	0		0	0	501
300	F	valid	paired	CR	564	0.958		831	501
300	F	valid	paired	CR	813	1.091		1017	501
300	F	valid	paired	CR	742	0.948		917	501
300	F	valid	paired	CR	904	1.045		1032	501
300	F	valid	paired	CR	827	0.971		1013	501
300	F	valid	paired	CR	786	0.947		923	501
300	F	valid	paired	CR	873	0.982		986	501
300	F	valid	paired	CR	711	0.965		968	501

300	F	valid	cs only	CR	916	0.371		1059	501
300	F	valid	cs only	no CR	0		0	0	501
300	F	valid	paired	no CR	0		0	0	501
300	F	valid	cs only	CR	771	0.828		892	501
300	F	valid	cs only	CR	773	0.91		976	501
300	F	valid	paired	CR	704	0.44		845	501
300	F	invalid	paired	0	0		0	0	501
300	F	valid	cs only	CR	739	0.991		1067	501
300	F	valid	cs only	CR	963	0.896		1108	501
300	F	valid	paired	no CR	0		0	0	501
300	F	valid	cs only	CR	889	1.05		1124	501
300	F	invalid	paired	0	0		0	0	501
300	F	valid	cs only	CR	885	1.031		1097	501
300	F	valid	paired	CR	892	0.574		1010	501
300	F	valid	cs only	CR	772		1	1094	501
300	F	valid	paired	CR	939	0.855		1036	501
300	F	valid	cs only	CR	872	1.158		1095	501
300	F	valid	cs only	CR	787	0.887		1140	501
300	F	valid	paired	CR	862	0.999		1028	501
300	F	valid	cs only	CR	709	1.12		1178	501
300	F	invalid	cs only	0	0		0	0	501
300	F	valid	cs only	CR	655	0.531		764	501
300	F	valid	paired	no CR	0		0	0	501
300	F	valid	paired	CR	889	0.777		977	501
300	F	valid	paired	CR	761	0.915		967	501
300	F	valid	paired	CR	845	0.934		956	501
300	F	valid	cs only	CR	1002	0.928		1180	501
300	F	valid	cs only	CR	829	1.074		1084	501
300	F	valid	cs only	CR	865	0.973		1089	501
300	F	valid	cs only	CR	777	0.876		1176	501
300	F	valid	us only	no CR	0		0	0	501
380	F	valid	us only	no CR	0		0	0	501
380	F	valid	cs only	no CR	0		0	0	501
380	F	invalid	paired	0	0		0	0	501
380	F	invalid	paired	0	0		0	0	501
380	F	valid	paired	no CR	0		0	0	501
380	F	valid	paired	no CR	0		0	0	501
380	F	valid	paired	CR	761	0.111		880	501
380	F	valid	paired	CR	969	0.077		999	501
380	F	valid	paired	CR	773	0.124		958	501
380	F	valid	paired	CR	903	0.514		1016	501
380	F	valid	paired	no CR	0		0	0	501
380	F	valid	paired	no CR	0		0	0	501
380	F	valid	paired	no CR	0		0	0	501
380	F	valid	paired	CR	855	0.752		979	501
380	F	valid	paired	CR	843	0.576		963	501
380	F	valid	paired	CR	933	0.248		1010	501
380	F	valid	paired	CR	939	0.687		1012	501
380	F	valid	paired	CR	854	0.284		990	501
380	F	valid	paired	CR	865	0.298		1005	501

380	F	valid	paired	CR	647	0.381		708	501
380	F	valid	paired	CR	869	0.199		988	501
380	F	valid	paired	CR	644	0.463		1012	501
380	F	valid	paired	CR	909	0.716		1016	501
380	F	valid	paired	no CR	0		0	0	501
380	F	valid	paired	no CR	0		0	0	501
380	F	valid	paired	CR	924	0.372		1016	501
380	F	valid	paired	no CR	0		0	0	501
380	F	valid	paired	CR	703	0.465		828	501
380	F	valid	paired	CR	805	0.692		1010	501
380	F	valid	paired	CR	822	0.11		870	501
380	F	valid	paired	no CR	0		0	0	501
380	F	valid	paired	no CR	0		0	0	501
380	F	valid	paired	no CR	0		0	0	501
380	F	valid	paired	CR	690	0.693		988	501
380	F	valid	paired	CR	856	0.17		1003	501
380	F	valid	paired	CR	808	0.139		877	501
380	F	valid	paired	CR	750	0.382		877	501
380	F	valid	paired	CR	922	0.482		1012	501
380	F	invalid	paired	0	0		0	0	501
380	F	valid	cs only	no CR	0		0	0	501
380	F	valid	paired	CR	525	0.283		1016	501
380	F	invalid	paired	0	0		0	0	501
380	F	valid	paired	no CR	0		0	0	501
380	F	invalid	paired	0	0		0	0	501
380	F	valid	paired	CR	747	0.71		836	501
380	F	invalid	paired	0	0		0	0	501
380	F	valid	paired	CR	916	0.175		1008	501
380	F	valid	paired	CR	782	0.21		1016	501
380	F	valid	paired	CR	781	0.131		889	501
380	F	invalid	cs only	0	0		0	0	501
380	F	valid	cs only	CR	1090	0.859		1247	501
380	F	valid	paired	CR	750	0.069		845	501
380	F	valid	cs only	CR	873	0.769		1355	501
380	F	valid	cs only	CR	1007	0.104		1102	501
380	F	valid	paired	CR	677	0.419		766	501
380	F	valid	paired	CR	845	0.865		1010	501
380	F	valid	cs only	CR	894	0.748		1217	501
380	F	valid	cs only	no CR	0		0	0	501
380	F	valid	paired	no CR	0		0	0	501
380	F	valid	cs only	no CR	0		0	0	501
380	F	valid	paired	no CR	0		0	0	501
380	F	valid	cs only	CR	1003	0.082		1099	501
380	F	valid	paired	CR	671	0.359		741	501
380	F	invalid	cs only	0	0		0	0	501
380	F	valid	paired	CR	790	0.059		903	501
380	F	valid	cs only	CR	701	0.58		785	501
380	F	valid	cs only	CR	872	0.675		1265	501
380	F	valid	paired	no CR	0		0	0	501
380	F	invalid	cs only	0	0		0	0	501

380	F	valid	cs only	CR	670	0.51		720	501
380	F	valid	cs only	CR	759	0.233		1127	501
380	F	valid	paired	no CR	0		0	0	501
380	F	valid	paired	CR	752	0.297		910	501
380	F	valid	paired	CR	771	0.12		907	501
380	F	valid	paired	CR	709	0.32		1018	501
380	F	valid	cs only	CR	841	0.413		1116	501
380	F	valid	cs only	CR	720	0.07		779	501
380	F	valid	cs only	CR	828	0.492		1014	501
380	F	valid	cs only	no CR	0		0	0	501
380	F	valid	us only	no CR	0		0	0	501
336	F	valid	us only	no CR	0		0	0	501
336	F	valid	cs only	no CR	0		0	0	501
336	F	valid	paired	CR	907	0.59		1008	501
336	F	valid	paired	CR	896	0.255		997	501
336	F	valid	paired	CR	841	0.158		1001	501
336	F	valid	paired	no CR	0		0	0	501
336	F	valid	paired	CR	675	0.124		997	501
336	F	valid	paired	CR	784	1.037		892	501
336	F	valid	paired	CR	628	1.143		886	501
336	F	valid	paired	CR	880	0.891		954	501
336	F	valid	paired	no CR	0		0	0	501
336	F	valid	paired	CR	818	0.989		994	501
336	F	valid	paired	CR	875	0.524		952	501
336	F	invalid	paired	0	0		0	0	501
336	F	invalid	paired	0	0		0	0	501
336	F	valid	paired	CR	681	0.532		944	501
336	F	valid	paired	CR	858	0.728		983	501
336	F	valid	paired	no CR	0		0	0	501
336	F	valid	paired	CR	916	0.938		997	501
336	F	valid	paired	CR	920	0.51		961	501
336	F	valid	paired	CR	814	0.961		990	501
336	F	valid	paired	CR	803	0.53		1018	501
336	F	valid	paired	CR	806	0.825		913	501
336	F	valid	paired	CR	785	1.099		953	501
336	F	valid	paired	no CR	0		0	0	501
336	F	valid	paired	CR	769	0.934		937	501
336	F	valid	paired	CR	840	0.202		869	501
336	F	valid	paired	CR	699	1.05		824	501
336	F	valid	paired	CR	807	1.076		979	501
336	F	valid	paired	CR	780	0.632		1029	501
336	F	valid	paired	no CR	0		0	0	501
336	F	valid	paired	CR	720	0.62		980	501
336	F	invalid	paired	0	0		0	0	501
336	F	valid	paired	CR	856	0.881		970	501
336	F	valid	paired	CR	943	0.893		1025	501
336	F	valid	paired	CR	777	0.957		858	501
336	F	valid	paired	no CR	0		0	0	501
336	F	valid	paired	CR	789	1.025		873	501
336	F	valid	paired	CR	862	0.766		1012	501

336	F	invalid	cs only	0	0		0	0	501
336	F	valid	paired	CR	896	0.954		980	501
336	F	valid	paired	no CR	0		0	0	501
336	F	valid	paired	CR	758	1.073		862	501
336	F	valid	paired	CR	900	0.901		1014	501
336	F	valid	paired	CR	799	0.435		969	501
336	F	valid	paired	no CR	0		0	0	501
336	F	valid	paired	CR	607	1.06		860	501
336	F	valid	paired	CR	727	0.773		779	501
336	F	valid	paired	CR	657	0.559		1018	501
336	F	valid	cs only	CR	900	0.349		1010	501
336	F	valid	cs only	no CR	0		0	0	501
336	F	valid	paired	no CR	0		0	0	501
336	F	valid	cs only	CR	1165	0.26		1266	501
336	F	valid	cs only	CR	855	0.465		1005	501
336	F	valid	paired	no CR	0		0	0	501
336	F	valid	paired	no CR	0		0	0	501
336	F	valid	cs only	CR	796	0.481		882	501
336	F	valid	cs only	no CR	0		0	0	501
336	F	valid	paired	no CR	0		0	0	501
336	F	valid	cs only	CR	699	0.657		1323	501
336	F	valid	paired	CR	890	1.003		997	501
336	F	valid	cs only	CR	921	0.247		980	501
336	F	valid	paired	CR	965	0.127		1010	501
336	F	valid	cs only	CR	890	0.384		1001	501
336	F	valid	paired	CR	773	0.954		1025	501
336	F	valid	cs only	CR	661	0.626		1102	501
336	F	valid	cs only	CR	718	0.162		1067	501
336	F	valid	paired	no CR	0		0	0	501
336	F	valid	cs only	CR	757	0.388		963	501
336	F	valid	cs only	CR	889	1.006		938	501
336	F	valid	cs only	CR	710	1.07		777	501
336	F	valid	paired	no CR	0		0	0	501
336	F	valid	paired	CR	709	0.98		870	501
336	F	valid	paired	CR	884	0.838		926	501
336	F	invalid	paired	0	0		0	0	501
336	F	valid	cs only	CR	781	0.686		875	501
336	F	valid	cs only	CR	631	0.722		760	501
336	F	valid	cs only	CR	904	1.063		1007	501
336	F	valid	cs only	CR	661	0.958		868	501
336	F	valid	us only	no CR	0		0	0	501
327	F	valid	us only	no CR	0		0	0	501
327	F	valid	cs only	CR	786	1.644		939	501
327	F	valid	paired	no CR	0		0	0	501
327	F	valid	paired	no CR	0		0	0	501
327	F	valid	paired	CR	677	0.818		786	501
327	F	invalid	paired	0	0		0	0	501
327	F	valid	paired	CR	829	0.635		967	501
327	F	valid	paired	no CR	0		0	0	501
327	F	valid	paired	CR	905	0.931		958	501

327	F	valid	paired	no CR	0		0	0	501
327	F	valid	paired	CR	873	1.227		961	501
327	F	valid	paired	CR	733	1.428		881	501
327	F	valid	paired	CR	758	1.048		1014	501
327	F	valid	paired	no CR	0		0	0	501
327	F	valid	paired	CR	875	2.211		990	501
327	F	valid	paired	CR	882	1.108		1024	501
327	F	valid	paired	CR	781	0.62		1014	501
327	F	valid	paired	CR	722	1.762		929	501
327	F	valid	paired	CR	873	0.47		935	501
327	F	valid	paired	no CR	0		0	0	501
327	F	valid	paired	CR	816	2.427		990	501
327	F	valid	paired	CR	724	2.36		956	501
327	F	valid	paired	CR	888	2.248		1010	501
327	F	valid	paired	CR	890	1.147		1014	501
327	F	valid	paired	CR	750	1.997		892	501
327	F	valid	paired	no CR	0		0	0	501
327	F	valid	paired	CR	750	1.942		905	501
327	F	valid	paired	CR	750	0.611		837	501
327	F	valid	paired	CR	799	2.563		935	501
327	F	valid	paired	no CR	0		0	0	501
327	F	valid	paired	CR	856	1.031		1022	501
327	F	valid	paired	CR	897	0.507		1010	501
327	F	valid	paired	CR	880	0.426		909	501
327	F	valid	paired	CR	918	0.862		1003	501
327	F	valid	paired	no CR	0		0	0	501
327	F	valid	paired	CR	871	1.861		941	501
327	F	valid	paired	CR	946	0.268		982	501
327	F	valid	paired	CR	926	0.678		1001	501
327	F	valid	paired	CR	959	1.184		1012	501
327	F	valid	cs only	CR	976	1.857		1101	501
327	F	valid	paired	no CR	0		0	0	501
327	F	valid	paired	no CR	0		0	0	501
327	F	valid	paired	CR	856	1.653		986	501
327	F	valid	paired	CR	831	1.573		924	501
327	F	valid	paired	no CR	0		0	0	501
327	F	valid	paired	CR	848	1.813		937	501
327	F	valid	paired	no CR	0		0	0	501
327	F	valid	paired	CR	826	1.301		888	501
327	F	valid	paired	CR	884	0.478		933	501
327	F	valid	cs only	CR	948	0.306		1018	501
327	F	valid	cs only	CR	892		1	1013	501
327	F	valid	paired	no CR	0		0	0	501
327	F	valid	cs only	CR	912	0.612		1075	501
327	F	valid	cs only	CR	996	0.582		1077	501
327	F	valid	paired	no CR	0		0	0	501
327	F	invalid	paired	0	0		0	0	501
327	F	valid	cs only	CR	855	0.831		1089	501
327	F	valid	cs only	CR	909	1.331		1088	501
327	F	valid	paired	no CR	0		0	0	501

327	F	valid	cs only	CR	732	0.357		1066	501
327	F	valid	paired	no CR	0		0	0	501
327	F	valid	cs only	CR	871	0.494		941	501
327	F	valid	paired	CR	707	0.44		940	501
327	F	valid	cs only	CR	863	0.379		944	501
327	F	valid	paired	CR	694	0.319		800	501
327	F	valid	cs only	no CR	0		0	0	501
327	F	valid	cs only	CR	1037	1.194		1157	501
327	F	valid	paired	CR	683	1.189		747	501
327	F	valid	cs only	CR	1172	1.932		963	501
327	F	invalid	cs only	0	0		0	0	501
327	F	valid	cs only	CR	860	1.337		1102	501
327	F	valid	paired	no CR	0		0	0	501
327	F	valid	paired	CR	686	0.476		903	501
327	F	valid	paired	CR	909	1.19		980	501
327	F	valid	paired	CR	848	1.499		988	501
327	F	valid	cs only	CR	777	1.714		948	501
327	F	valid	cs only	CR	780	1.207		935	501
327	F	valid	cs only	no CR	0		0	0	501
327	F	valid	cs only	CR	1008	0.705		1071	501
327	F	valid	us only	no CR	0		0	0	501
528	F	valid	us only	no CR	0		0	0	501
528	F	valid	cs only	no CR	0		0	0	501
528	F	valid	paired	no CR	0		0	0	501
528	F	valid	paired	no CR	0		0	0	501
528	F	valid	paired	no CR	0		0	0	501
528	F	valid	paired	no CR	0		0	0	501
528	F	invalid	paired	0	0		0	0	501
528	F	valid	paired	no CR	0		0	0	501
528	F	valid	paired	no CR	0		0	0	501
528	F	valid	paired	no CR	0		0	0	501
528	F	valid	paired	no CR	0		0	0	501
528	F	valid	paired	no CR	0		0	0	501
528	F	valid	paired	CR	833	0.074		929	501
528	F	valid	paired	no CR	0		0	0	501
528	F	valid	paired	CR	775	0.097		909	501
528	F	valid	paired	CR	665	0.297		893	501
528	F	valid	paired	CR	786	0.152		910	501
528	F	valid	paired	CR	665	0.112		1020	501
528	F	valid	paired	no CR	0		0	0	501
528	F	valid	paired	no CR	0		0	0	501
528	F	valid	paired	no CR	0		0	0	501
528	F	valid	paired	no CR	0		0	0	501
528	F	valid	paired	no CR	0		0	0	501
528	F	valid	paired	no CR	0		0	0	501
528	F	valid	paired	CR	796	0.284		881	501
528	F	valid	paired	CR	943	0.12		1019	501
528	F	valid	paired	CR	767	0.429		950	501
528	F	valid	paired	CR	777	0.457		903	501
528	F	valid	paired	CR	805	0.202		987	501

528	F	valid	paired	CR	690	0.217		1014	501
528	F	valid	paired	CR	958	0.08		1003	501
528	F	valid	paired	CR	592	0.364		991	501
528	F	invalid	paired	0	0		0	0	501
528	F	valid	paired	CR	856	0.102		942	501
528	F	valid	paired	CR	931	-0.003		963	501
528	F	valid	paired	no CR	0		0	0	501
528	F	valid	paired	no CR	0		0	0	501
528	F	valid	paired	no CR	0		0	0	501
528	F	valid	paired	CR	771	0.177		922	501
528	F	valid	cs only	CR	756	0.343		1312	501
528	F	valid	paired	no CR	0		0	0	501
528	F	valid	paired	CR	582	0.227		1015	501
528	F	valid	paired	CR	621	0.494		682	501
528	F	valid	paired	CR	814	0.183		880	501
528	F	valid	paired	CR	805	0.136		880	501
528	F	valid	paired	CR	978	0.116		1029	501
528	F	valid	paired	CR	807	0.17		867	501
528	F	valid	paired	CR	738	0.12		880	501
528	F	valid	paired	CR	750	0.065		824	501
528	F	invalid	cs only	0	0		0	0	501
528	F	valid	cs only	no CR	0		0	0	501
528	F	valid	paired	no CR	0		0	0	501
528	F	invalid	cs only	0	0		0	0	501
528	F	valid	cs only	CR	1173	0.258		1219	501
528	F	valid	paired	no CR	0		0	0	501
528	F	valid	paired	no CR	0		0	0	501
528	F	valid	cs only	CR	656	0.187		820	501
528	F	valid	cs only	no CR	0		0	0	501
528	F	valid	paired	no CR	0		0	0	501
528	F	valid	cs only	CR	683	0.178		831	501
528	F	valid	paired	no CR	0		0	0	501
528	F	valid	cs only	CR	699	0.255		818	501
528	F	valid	paired	no CR	0		0	0	501
528	F	invalid	cs only	0	0		0	0	501
528	F	valid	paired	CR	839	0.411		925	501
528	F	valid	cs only	CR	873	0.335		1223	501
528	F	valid	cs only	CR	619	0.693		697	501
528	F	valid	paired	no CR	0		0	0	501
528	F	valid	cs only	no CR	0		0	0	501
528	F	valid	cs only	no CR	0		0	0	501
528	F	valid	cs only	CR	781	0.044		856	501
528	F	valid	paired	CR	646	0.225		755	501
528	F	valid	paired	CR	856	0.214		916	501
528	F	valid	paired	CR	799	0.297		907	501
528	F	valid	paired	CR	750	-0.051		845	501
528	F	valid	cs only	CR	760	0.461		1177	501
528	F	valid	cs only	CR	1166	0.123		1203	501
528	F	valid	cs only	no CR	0		0	0	501
528	F	valid	cs only	no CR	0		0	0	501

528	F	valid	us only	no CR	0		0	0	501
298	F	valid	us only	no CR	0		0	0	501
298	F	valid	cs only	CR	978	0.028		995	501
298	F	valid	paired	no CR	0		0	0	501
298	F	valid	paired	no CR	0		0	0	501
298	F	valid	paired	no CR	0		0	0	501
298	F	valid	paired	CR	703	0.089		1008	501
298	F	valid	paired	CR	795	0.081		973	501
298	F	valid	paired	CR	709	0.083		905	501
298	F	valid	paired	CR	662	0.362		858	501
298	F	valid	paired	CR	799	0.104		937	501
298	F	valid	paired	CR	794	0.085		899	501
298	F	valid	paired	CR	875	0.103		916	501
298	F	valid	paired	CR	617	0.837		1021	501
298	F	valid	paired	no CR	0		0	0	501
298	F	valid	paired	no CR	0		0	0	501
298	F	valid	paired	no CR	0		0	0	501
298	F	valid	paired	no CR	0		0	0	501
298	F	valid	paired	CR	567	0.616		1005	501
298	F	valid	paired	CR	824	0.042		969	501
298	F	valid	paired	CR	860	0.175		1014	501
298	F	valid	paired	no CR	0		0	0	501
298	F	valid	paired	CR	914	0.076		986	501
298	F	valid	paired	no CR	0		0	0	501
298	F	valid	paired	CR	803	0.236		997	501
298	F	valid	paired	CR	879	0.75		1016	501
298	F	valid	paired	no CR	0		0	0	501
298	F	valid	paired	no CR	0		0	0	501
298	F	valid	paired	CR	662	0.22		995	501
298	F	valid	paired	CR	732	0.293		884	501
298	F	valid	paired	no CR	0		0	0	501
298	F	valid	paired	CR	703	0.467		1014	501
298	F	valid	paired	CR	861	0.723		1005	501
298	F	valid	paired	no CR	0		0	0	501
298	F	valid	paired	CR	959	0.303		1012	501
298	F	valid	paired	CR	869	0.064		993	501
298	F	valid	paired	CR	848	0.726		982	501
298	F	valid	paired	CR	944	0.202		1018	501
298	F	invalid	paired	0	0		0	0	501
298	F	valid	paired	CR	925	0.022		958	501
298	F	valid	cs only	CR	748	0.661		1637	501
298	F	invalid	paired	0	0		0	0	501
298	F	valid	paired	no CR	0		0	0	501
298	F	invalid	paired	0	0		0	0	501
298	F	valid	paired	no CR	0		0	0	501
298	F	valid	paired	CR	694	0.886		764	501
298	F	valid	paired	no CR	0		0	0	501
298	F	valid	paired	CR	937	0.061		1008	501
298	F	valid	paired	no CR	0		0	0	501
298	F	valid	paired	CR	813	0.058		871	501

298	F	valid	cs only	CR	857	0.36		1085	501
298	F	valid	cs only	no CR	0		0	0	501
298	F	valid	paired	CR	640	0.348		701	501
298	F	valid	cs only	no CR	0		0	0	501
298	F	valid	cs only	no CR	0		0	0	501
298	F	valid	paired	no CR	0		0	0	501
298	F	valid	paired	no CR	0		0	0	501
298	F	valid	cs only	CR	749	0.052		781	501
298	F	valid	cs only	no CR	0		0	0	501
298	F	valid	paired	no CR	0		0	0	501
298	F	valid	cs only	CR	1008	0.103		1201	501
298	F	valid	paired	no CR	0		0	0	501
298	F	valid	cs only	no CR	0		0	0	501
298	F	valid	paired	no CR	0		0	0	501
298	F	valid	cs only	no CR	0		0	0	501
298	F	invalid	paired	0	0		0	0	501
298	F	valid	cs only	no CR	0		0	0	501
298	F	valid	cs only	no CR	0		0	0	501
298	F	valid	paired	no CR	0		0	0	501
298	F	valid	cs only	no CR	0		0	0	501
298	F	valid	cs only	CR	1057	0.139		1158	501
298	F	valid	cs only	no CR	0		0	0	501
298	F	invalid	paired	0	0		0	0	501
298	F	valid	paired	CR	694	0.036		726	501
298	F	valid	paired	CR	583	0.079		622	501
298	F	valid	paired	no CR	0		0	0	501
298	F	valid	cs only	no CR	0		0	0	501
298	F	valid	cs only	CR	984	0.182		1066	501
298	F	valid	cs only	no CR	0		0	0	501
298	F	valid	cs only	no CR	0		0	0	501
298	F	invalid	us only	0	0		0	0	501
262	F	valid	us only	no CR	0		0	0	501
262	F	valid	cs only	no CR	0		0	0	501
262	F	valid	paired	no CR	0		0	0	501
262	F	invalid	paired	0	0		0	0	501
262	F	valid	paired	no CR	0		0	0	501
262	F	valid	paired	no CR	0		0	0	501
262	F	valid	paired	no CR	0		0	0	501
262	F	valid	paired	no CR	0		0	0	501
262	F	valid	paired	CR	877	0.293		923	501
262	F	valid	paired	no CR	0		0	0	501
262	F	valid	paired	no CR	0		0	0	501
262	F	valid	paired	CR	841	0.339		914	501
262	F	valid	paired	no CR	0		0	0	501
262	F	valid	paired	no CR	0		0	0	501
262	F	valid	paired	no CR	0		0	0	501
262	F	valid	paired	no CR	0		0	0	501
262	F	valid	paired	no CR	0		0	0	501
262	F	valid	paired	no CR	0		0	0	501
262	F	valid	paired	no CR	0		0	0	501

262	F	valid	paired	CR	820	0.134		878	501
262	F	valid	paired	no CR	0		0	0	501
262	F	valid	paired	CR	848	0.085		905	501
262	F	valid	paired	CR	837	0.089		1012	501
262	F	valid	paired	no CR	0		0	0	501
262	F	valid	paired	CR	865	0.128		1035	501
262	F	invalid	paired	0	0		0	0	501
262	F	valid	paired	CR	780	0.223		881	501
262	F	valid	paired	CR	888	0.239		928	501
262	F	invalid	paired	0	0		0	0	501
262	F	valid	paired	CR	829	0.267		891	501
262	F	valid	paired	CR	756	0.27		869	501
262	F	valid	paired	no CR	0		0	0	501
262	F	valid	paired	CR	860	0.109		925	501
262	F	valid	paired	CR	888	0.243		1010	501
262	F	invalid	paired	0	0		0	0	501
262	F	valid	paired	CR	824	0.275		979	501
262	F	invalid	paired	0	0		0	0	501
262	F	invalid	paired	0	0		0	0	501
262	F	valid	paired	CR	809	0.06		935	501
262	F	valid	cs only	CR	844	0.293		934	501
262	F	valid	paired	no CR	0		0	0	501
262	F	valid	paired	no CR	0		0	0	501
262	F	valid	paired	CR	954	0.016		1010	501
262	F	valid	paired	CR	671	0.195		993	501
262	F	valid	paired	CR	841	0.159		978	501
262	F	valid	paired	CR	835	0.218		968	501
262	F	valid	paired	CR	781	0.26		892	501
262	F	valid	paired	no CR	0		0	0	501
262	F	valid	paired	no CR	0		0	0	501
262	F	valid	cs only	CR	805	0.005		871	501
262	F	invalid	cs only	0	0		0	0	501
262	F	valid	paired	no CR	0		0	0	501
262	F	invalid	cs only	0	0		0	0	501
262	F	valid	cs only	no CR	0		0	0	501
262	F	valid	paired	no CR	0		0	0	501
262	F	invalid	paired	0	0		0	0	501
262	F	valid	cs only	no CR	0		0	0	501
262	F	valid	cs only	no CR	0		0	0	501
262	F	valid	paired	no CR	0		0	0	501
262	F	valid	cs only	no CR	0		0	0	501
262	F	valid	paired	no CR	0		0	0	501
262	F	valid	cs only	no CR	0		0	0	501
262	F	valid	paired	no CR	0		0	0	501
262	F	valid	cs only	CR	816	0.328		1086	501
262	F	invalid	paired	0	0		0	0	501
262	F	invalid	cs only	0	0		0	0	501
262	F	valid	cs only	no CR	0		0	0	501
262	F	valid	paired	no CR	0		0	0	501
262	F	invalid	cs only	0	0		0	0	501

262	F	valid	cs only	no CR	0		0	0	501
262	F	valid	cs only	no CR	0		0	0	501
262	F	invalid	paired	0	0		0	0	501
262	F	valid	paired	CR	747	0.166		809	501
262	F	invalid	paired	0	0		0	0	501
262	F	valid	paired	no CR	0		0	0	501
262	F	valid	cs only	CR	1008	0.128		1098	501
262	F	valid	cs only	no CR	0		0	0	501
262	F	invalid	cs only	0	0		0	0	501
262	F	invalid	cs only	0	0		0	0	501
262	F	valid	us only	no CR	0		0	0	501
489	M	valid	us only	no CR	0		0	0	501
489	M	valid	cs only	CR	922	0.054		1025	501
489	M	valid	paired	no CR	0		0	0	501
489	M	valid	paired	no CR	0		0	0	501
489	M	valid	paired	no CR	0		0	0	501
489	M	valid	paired	no CR	0		0	0	501
489	M	valid	paired	no CR	0		0	0	501
489	M	valid	paired	no CR	0		0	0	501
489	M	valid	paired	no CR	0		0	0	501
489	M	valid	paired	no CR	0		0	0	501
489	M	invalid	paired	no CR	0		0	0	501
489	M	valid	paired	no CR	0		0	0	501
489	M	valid	paired	no CR	0		0	0	501
489	M	valid	paired	CR	624	0.074		956	501
489	M	invalid	paired	0	0		0	0	501
489	M	valid	paired	CR	794	1.049		877	501
489	M	valid	paired	CR	953	0.531		981	501
489	M	valid	paired	no CR	0		0	0	501
489	M	valid	paired	no CR	0		0	0	501
489	M	valid	paired	no CR	0		0	0	501
489	M	valid	paired	no CR	0		0	0	501
489	M	invalid	paired	0	0		0	0	501
489	M	valid	paired	CR	682	1.149		713	501
489	M	valid	paired	no CR	0		0	0	501
489	M	valid	paired	no CR	0		0	0	501
489	M	valid	paired	CR	892	0.718		967	501
489	M	valid	paired	no CR	0		0	0	501
489	M	valid	paired	no CR	0		0	0	501
489	M	invalid	paired	0	0		0	0	501
489	M	valid	paired	CR	724	0.077		777	501
489	M	valid	paired	CR	978	0.225		1027	501
489	M	valid	paired	CR	905	0.923		1027	501
489	M	valid	paired	CR	766	0.157		835	501
489	M	valid	paired	no CR	0		0	0	501
489	M	valid	paired	no CR	0		0	0	501
489	M	valid	paired	no CR	0		0	0	501
489	M	valid	paired	CR	935	0.965		1016	501
489	M	valid	paired	CR	646	0.117		971	501
489	M	valid	paired	no CR	0		0	0	501

489	M	valid	cs only	CR	1099	0.191		1153	501
489	M	valid	paired	no CR	0		0	0	501
489	M	valid	paired	no CR	0		0	0	501
489	M	valid	paired	no CR	0		0	0	501
489	M	valid	paired	no CR	0		0	0	501
489	M	valid	paired	no CR	0		0	0	501
489	M	valid	paired	no CR	0		0	0	501
489	M	valid	paired	no CR	0		0	0	501
489	M	valid	paired	no CR	0		0	0	501
489	M	valid	paired	no CR	0		0	0	501
489	M	valid	cs only	CR	957	0.283		1103	501
489	M	valid	cs only	CR	715	0.046		758	501
489	M	valid	paired	no CR	0		0	0	501
489	M	valid	cs only	CR	1071	0.936		1096	501
489	M	valid	cs only	no CR	0		0	0	501
489	M	valid	paired	no CR	0		0	0	501
489	M	valid	paired	no CR	0		0	0	501
489	M	valid	cs only	no CR	0		0	0	501
489	M	valid	cs only	CR	1065	0.949		1095	501
489	M	valid	paired	no CR	0		0	0	501
489	M	valid	cs only	no CR	0		0	0	501
489	M	valid	paired	no CR	0		0	0	501
489	M	valid	cs only	CR	718	0.395		1054	501
489	M	valid	paired	no CR	0		0	0	501
489	M	valid	cs only	CR	1165	0.105		1511	501
489	M	invalid	paired	0	0		0	0	501
489	M	valid	cs only	no CR	0		0	0	501
489	M	valid	cs only	CR	1069	0.746		1114	501
489	M	valid	paired	CR	611	0.846		639	501
489	M	valid	cs only	no CR	0		0	0	501
489	M	valid	cs only	CR	1041	1.151		1101	501
489	M	valid	cs only	no CR	0		0	0	501
489	M	valid	paired	no CR	0		0	0	501
489	M	valid	paired	no CR	0		0	0	501
489	M	valid	paired	no CR	0		0	0	501
489	M	valid	paired	no CR	0		0	0	501
489	M	valid	cs only	CR	889	0.109		1107	501
489	M	valid	cs only	no CR	0		0	0	501
489	M	valid	cs only	CR	635	0.129		697	501
489	M	valid	cs only	no CR	0		0	0	501
489	M	valid	us only	no CR	0		0	0	501

UR	UR_onset	UR1_peakamp	UR1_peaktime
UR	1047	1.233	1096
0	0	0	0
no UR	0	0	0
no UR	0	0	0
UR	1039	1.363	1106
0	0	0	0
0	0	0	0
0	0	0	0
UR	1039	1.097	1094
UR	1034	0.989	1096
UR	1026	1.045	1092
0	0	0	0
UR	1032	1.037	1086
0	0	0	0
UR	1031	1.072	1089
UR	1019	1.011	1091
UR	1049	0.993	1139
0	0	0	0
UR	1021	0.973	1090
UR	1032	0.979	1077
UR	1045	0.926	1110
UR	1017	1.065	1099
UR	1038	0.972	1108
0	0	0	0
UR	1033	1.058	1096
UR	1037	0.937	1110
0	0	0	0
UR	1038	0.984	1145
0	0	0	0
UR	1022	0.983	1103
UR	1039	0.925	1100
UR	1039	0.951	1083
UR	1034	0.883	1098
UR	1034	0.95	1088
0	0	0	0
0	0	0	0
0	0	0	0
UR	1033	1.035	1083
0	0	0	0
no UR	0	0	0
0	0	0	0
UR	1026	0.926	1083
UR	1021	0.943	1102
0	0	0	0
UR	1041	0.987	1110
UR	1031	0.989	1136
UR	1034	0.91	1092
UR	1037	0.911	1092
UR	1039	0.915	1110

no UR	0		0	0
UR	1036	1.033		1113
UR	1024	1.069		1110
no UR	0		0	0
0	0		0	0
UR	1034	1.081		1079
0	0		0	0
UR	1016	0.908		1103
UR	1047	0.751		1110
UR	1030	1.04		1081
UR	1033	0.878		1112
UR	1031	1.125		1104
no UR	0		0	0
no UR	0		0	0
no UR	0		0	0
no UR	0		0	0
no UR	0		0	0
no UR	0		0	0
no UR	0		0	0
no UR	0		0	0
UR	1043	1.002		1110
UR	1045	0.575		1094
UR	1059	0.542		1109
no UR	0		0	0
no UR	0		0	0
UR	1045	0.852		1090
UR	1032	0.617		1112
0	0		0	0
0	0		0	0
UR	1039	0.582		1081
UR	1032	0.542		1073
UR	1049	0.519		1069
UR	1064	2.207		1162
UR	1071	1.015		1160
UR	1035	0.555		1080
UR	1037	0.531		1086
UR	1005	0.551		1070
UR	1039	0.541		1077
UR	1030	0.558		1062
UR	1028	0.596		1081
UR	1105	1.554		1179
UR	1017	0.573		1069
UR	1028	0.752		1079
UR	1041	0.561		1056
UR	1017	0.49		1074
UR	1039	0.511		1045
UR	1030	0.534		1054
UR	1049	0.494		1092
UR	1032	0.991		1036
UR	1062	1.236		1147

UR	1039	1.074	1144
UR	1034	0.498	1066
UR	1056	1.485	1137
UR	1075	1.24	1152
UR	1000	0.313	1015
UR	1047	0.563	1088
UR	1030	0.65	1075
UR	1005	1.636	1126
UR	1058	1.029	1100
UR	1081	1.3	1137
no UR	0	0	0
UR	1086	1.688	1149
UR	1032	1.462	1147
UR	1013	1.336	1034
UR	1032	0.86	1037
UR	1030	1.999	1064
UR	1041	1.191	1132
UR	1079	1.115	1158
UR	1041	0.583	1051
UR	1073	1.909	1122
no UR	0	0	0
UR	1083	1.54	1147
UR	1000	1.363	1090
no UR	0	0	0
no UR	0	0	0
UR	1041	0.497	1051
UR	1015	1.536	1051
UR	1024	0.61	1073
UR	1051	0.626	1088
UR	1045	0.648	1086
UR	1007	0.221	1054
UR	1043	0.569	1086
no UR	0	0	0
no UR	0	0	0
no UR	0	0	0
no UR	0	0	0
no UR	0	0	0
no UR	0	0	0
no UR	0	0	0
no UR	0	0	0
no UR	0	0	0
UR	1058	1.085	1096
UR	1056	1.053	1093
no UR	0	0	0
no UR	0	0	0
UR	1048	0.974	1085
UR	1051	0.234	1086
UR	1052	0.906	1098
UR	1050	1.019	1084
UR	1050	0.964	1092

UR	1054	0.982	1090
UR	1024	0.928	1072
UR	1028	0.94	1086
UR	1057	0.889	1101
UR	1038	0.929	1088
UR	1040	0.966	1091
UR	1021	1.018	1076
UR	1048	0.947	1092
UR	1051	1.038	1089
UR	1047	1.046	1086
UR	1049	0.249	1083
UR	1032	0.237	1066
UR	1058	0.231	1081
UR	1049	0.223	1077
UR	1045	0.205	1081
UR	1043	0.204	1083
UR	1054	0.219	1092
UR	1043	0.208	1077
UR	1045	0.196	1075
UR	1033	1.385	1080
UR	1044	1.321	1095
UR	1046	1.326	1094
UR	1064	0.61	1107
UR	1041	1.403	1084
UR	1054	1.339	1094
UR	1050	1.314	1098
UR	1031	1.203	1092
UR	1041	1.242	1090
UR	1055	1.228	1098
UR	1044	1.176	1102
no UR	0	0	0
UR	1035	1.41	1094
UR	1030	1.322	1078
UR	1028	1.299	1076
UR	1050	1.343	1096
UR	1037	1.4	1079
UR	1014	1.291	1068
UR	1039	1.339	1079
UR	1037	1.405	1087
UR	1053	1.317	1101
no UR	0	0	0
UR	1034	1.328	1076
UR	1053	1.242	1097
no UR	0	0	0
no UR	0	0	0
UR	1049	1.302	1089
UR	1037	1.057	1071
UR	1030	1.045	1075
UR	1039	1.391	1100
0	0	0	0

UR	1045	1.107		1088
UR	1035	1.249		1093
no UR	0		0	0
no UR	0		0	0
no UR	0		0	0
no UR	0		0	0
no UR	0		0	0
no UR	0		0	0
no UR	0		0	0
no UR	0		0	0
UR	954	0.914		1046
UR	1034	0.983		1071
0	0		0	0
0	0		0	0
no UR	0		0	0
UR	1036	1.006		1079
UR	1024	0.447		1054
UR	1034	0.814		1075
UR	1036	0.833		1078
UR	1032	0.789		1074
UR	1029	0.832		1069
UR	1028	0.792		1074
UR	1041	0.483		1075
UR	1036	0.704		1072
UR	1038	0.702		1075
UR	1019	0.75		1065
0	0		0	0
UR	1036	1.095		1076
UR	1035	1.027		1083
UR	1039	1.061		1088
UR	1019	1.131		1078
0	0		0	0
UR	1009	1.037		1067
UR	1025	0.943		1083
UR	1027	1.096		1077
UR	1037	1.187		1082
UR	1022	1.236		1081
UR	1039	1.126		1081
UR	1030	1.147		1072
UR	1022	1.09		1080
UR	1037	1.134		1083
0	0		0	0
UR	1032	0.963		1064
UR	1028	1.135		1078
UR	1033	1.124		1079
UR	1034	1.045		1088
UR	1040	1.413		1107
UR	1038	1.187		1085
UR	1035	1.183		1085
0	0		0	0

no UR	0		0	0
UR	1026	1.311		1092
UR	1033	1.214		1098
UR	1006	1.142		1111
UR	1054	0.745		1110
UR	1017	0.952		1051
UR	1035	1.094		1099
UR	1022	0.91		1054
0	0		0	0
UR	1024	1.034		1060
no UR	0		0	0
UR	1071	0.353		1128
UR	1045	1.122		1073
no UR	0		0	0
no UR	0		0	0
UR	1047	1.014		1110
0	0		0	0
UR	1039	1.053		1109
UR	1037	1.205		1107
UR	1043	1.085		1086
UR	1035	1.26		1103
UR	1031	1.199		1090
no UR	0		0	0
no UR	0		0	0
no UR	0		0	0
no UR	0		0	0
no UR	0		0	0
no UR	0		0	0
no UR	0		0	0
no UR	0		0	0
UR	1042	1.12		1096
UR	1030	1.275		1137
UR	1048	1.418		1138
no UR	0		0	0
no UR	0		0	0
UR	1046	1.313		1149
UR	1048	1.406		1174
UR	1027	1.926		1123
UR	1057	1.465		1166
UR	1060	1.188		1156
UR	1031	1.265		1128
UR	1044	1.279		1153
UR	1047	2.169		1145
UR	1050	1.43		1140
UR	1037	1.26		1150
UR	1050	1.109		1119
UR	1054	1.081		1128
UR	1041	0.585		1069
UR	1029	0.963		1103
UR	1041	1.169		1141

UR	1036	1.09		1143
UR	1033	1.199		1144
UR	1041	1.027		1121
UR	1035	1.228		1144
UR	1037	1.336		1127
UR	1034	1.057		1097
UR	1039	0.773		1060
UR	1028	1.117		1110
UR	1024	0.829		1103
0	0		0	0
UR	1021	1.06		1141
UR	1030	0.761		1110
UR	1035	1.187		1144
UR	1028	0.776		1060
UR	1036	1.165		1148
UR	1033	1.011		1129
UR	1029	0.871		1099
UR	1050	0.817		1120
UR	1030	0.718		1099
UR	1026	0.792		1096
no UR	0		0	0
UR	1037	1.238		1162
UR	1023	0.975		1160
UR	1036	1.195		1146
UR	1025	0.613		1101
0	0		0	0
UR	1024	1.014		1116
UR	1026	0.826		1100
UR	1054	0.257		1122
UR	1030	0.557		1092
no UR	0		0	0
UR	1041	0.877		1125
UR	1043	0.473		1128
no UR	0		0	0
no UR	0		0	0
UR	1040	0.359		1123
UR	1041	0.468		1111
UR	1045	0.548		1110
UR	1015	0.293		1073
0	0		0	0
UR	1045	0.817		1116
UR	1038	0.489		1119
no UR	0		0	0
no UR	0		0	0
no UR	0		0	0
0	0		0	0
no UR	0		0	0
no UR	0		0	0
no UR	0		0	0
no UR	0		0	0

UR	1016	1.127		1163
UR	1027	0.865		1066
UR	1032	1.306		1073
no UR	0		0	0
no UR	0		0	0
UR	1032	1.038		1073
UR	1022	0.625		1063
UR	1025	0.889		1067
UR	1044	1.007		1079
UR	1012	0.898		1075
UR	1043	0.972		1075
UR	1033	0.964		1085
0	0		0	0
UR	1029	1.155		1066
UR	1009	0.82		1068
UR	1027	1.056		1065
UR	1025	0.775		1058
UR	1033	0.847		1079
UR	1039	0.96		1074
UR	1037	0.96		1078
UR	1010	1.102		1075
UR	1020	1.102		1078
UR	1026	0.926		1145
UR	1033	0.897		1070
UR	1037	1.06		1086
UR	1032	0.892		1060
UR	1071	0.983		1126
UR	1035	0.843		1084
UR	1039	1.276		1072
0	0		0	0
UR	1032	0.906		1068
UR	1026	0.767		1078
UR	1023	0.949		1063
UR	1026	0.955		1078
UR	1042	0.968		1078
UR	1016	0.847		1076
UR	1033	0.319		1102
UR	1049	0.755		1107
UR	1037	0.974		1105
UR	1041	0.673		1080
0	0		0	0
UR	1048	1.044		1091
UR	1034	1.122		1087
UR	1033	1.244		1080
UR	1030	0.961		1082
UR	1033	0.969		1116
UR	1034	0.881		1087
UR	1045	0.872		1086
UR	1015	0.847		1084
UR	1051	0.901		1123

no UR	0		0	0
UR	1046	0.659		1093
UR	1044	0.793		1117
no UR	0		0	0
no UR	0		0	0
UR	1016	0.841		1092
UR	1038	0.84		1090
UR	1018	0.809		1080
UR	1041	0.858		1096
UR	1041	0.812		1084
UR	1051	0.853		1113
UR	1033	1.073		1069
no UR	0		0	0
no UR	0		0	0
no UR	0		0	0
no UR	0		0	0
no UR	0		0	0
no UR	0		0	0
no UR	0		0	0
no UR	0		0	0
UR	1040	0.923		1083
UR	1062	1.204		1103
UR	1062	1.106		1104
no UR	0		0	0
no UR	0		0	0
UR	1072	1.147		1116
0	0		0	0
UR	1064	1.115		1104
UR	1065	1.034		1109
UR	1046	1.124		1116
UR	1064	1.077		1103
0	0		0	0
UR	1053	1.172		1105
UR	1065	1.211		1112
UR	1062	0.938		1104
UR	1042	0.938		1113
UR	1056	0.905		1120
UR	1054	0.976		1108
UR	1032	0.893		1147
UR	1039	1.279		1103
UR	1052	1.174		1099
0	0		0	0
UR	1074	0.791		1133
0	0		0	0
UR	1022	0.966		1163
UR	1032	1.062		1090
UR	1059	1.1		1103
UR	1065	0.992		1130
UR	1057	1.131		1098
UR	1043	0.989		1113

UR	1057	1.234		1113
UR	1061	1.116		1103
UR	1063	1.187		1110
UR	1045	0.512		1064
UR	1043	1.151		1091
UR	1088	0.835		1181
UR	1088	1.063		1156
UR	1075	0.93		1154
UR	1065	0.735		1134
UR	1067	0.945		1129
no UR	0		0	0
UR	1028	1.017		1126
UR	1051	0.99		1110
UR	1044	1.717		1107
UR	1069	0.96		1128
UR	1043	1.21		1110
UR	1056	0.991		1110
UR	1077	0.92		1110
UR	1040	0.993		1127
UR	1047	1.104		1099
no UR	0		0	0
UR	1040	1.058		1104
UR	1073	0.929		1110
no UR	0		0	0
no UR	0		0	0
UR	1043	0.953		1092
UR	1047	0.978		1099
UR	1043	1.085		1101
UR	1058	0.983		1100
UR	1038	0.827		1088
UR	1039	0.497		1049
UR	1024	1.181		1087
no UR	0		0	0
no UR	0		0	0
no UR	0		0	0
no UR	0		0	0
no UR	0		0	0
0	0		0	0
no UR	0		0	0
no UR	0		0	0
UR	1042	0.815		1112
UR	1028	0.687		1071
UR	1031	0.726		1076
no UR	0		0	0
no UR	0		0	0
UR	1031	0.742		1075
UR	1032	1.305		1077
0	0		0	0
UR	1037	1.032		1084
UR	1025	1.307		1081

UR	1024	1.136		1074
UR	1026	1.372		1066
0	0		0	0
0	0		0	0
UR	1018	1.061		1059
UR	1027	1.015		1071
UR	1027	0.991		1074
UR	1025	0.993		1081
UR	1025	1.015		1071
UR	1030	0.719		1073
UR	1023	0.856		1064
UR	1016	0.951		1052
UR	1028	0.943		1069
UR	1024	1.146		1083
UR	1022	1.026		1065
UR	1028	0.952		1060
0	0		0	0
UR	1032	1.21		1090
UR	1013	0.881		1043
UR	1029	0.856		1060
UR	1029	0.755		1081
UR	1051	0.898		1066
0	0		0	0
UR	1030	0.865		1067
UR	1019	0.932		1071
0	0		0	0
UR	1009	1.069		1070
UR	1029	1.089		1076
UR	1028	0.982		1070
UR	1014	0.941		1064
0	0		0	0
0	0		0	0
UR	1039	0.846		1064
0	0		0	0
0	0		0	0
UR	1029	0.706		1065
0	0		0	0
UR	1037	0.98		1062
0	0		0	0
UR	1034	0.766		1060
no UR	0		0	0
UR	1030	0.79		1067
UR	1032	0.774		1085
no UR	0		0	0
no UR	0		0	0
UR	1045	0.687		1071
0	0		0	0
UR	1043	0.671		1098
0	0		0	0
UR	1025	1.018		1065

UR	1028	0.918		1060
UR	1030	0.857		1065
no UR	0		0	0
no UR	0		0	0
no UR	0		0	0
no UR	0		0	0
no UR	0		0	0
no UR	0		0	0
no UR	0		0	0
no UR	0		0	0
UR	1030	0.936		1080
UR	1034	1.033		1069
0	0		0	0
no UR	0		0	0
no UR	0		0	0
0	0		0	0
UR	1037	0.726		1077
UR	1028	0.685		1064
UR	1038	0.772		1080
UR	1017	0.817		1058
UR	1017	0.699		1058
UR	1026	0.725		1068
UR	1049	0.674		1066
UR	1028	0.943		1069
0	0		0	0
UR	1030	1.198		1067
UR	1037	1.085		1077
UR	1032	0.641		1079
UR	1020	0.672		1061
UR	1026	0.634		1069
UR	1028	0.98		1080
UR	1045	0.788		1117
0	0		0	0
UR	1027	1.231		1078
UR	1024	0.888		1060
UR	1037	0.855		1122
UR	1015	0.662		1066
UR	1046	0.827		1085
UR	1040	0.781		1078
no UR	0		0	0
UR	1023	0.79		1067
UR	1030	0.887		1062
UR	1021	0.821		1070
UR	1044	0.717		1085
UR	1024	0.751		1076
UR	1039	0.748		1080
UR	1022	1.106		1087
UR	1032	0.784		1076
0	0		0	0
UR	1035	0.801		1083

no UR	0		0	0
UR	1022	1.111		1060
UR	1032	0.909		1064
UR	1034	0.837		1094
0	0		0	0
UR	1069	0.816		1110
UR	1037	0.944		1079
UR	1027	0.966		1085
UR	1003	0.549		1092
UR	1045	0.742		1093
no UR	0		0	0
UR	1036	0.895		1091
UR	1028	0.736		1041
0	0		0	0
no UR	0		0	0
UR	1045	1.001		1084
UR	1030	0.94		1077
UR	1033	1.153		1076
UR	1034	0.956		1082
UR	1026	1.038		1067
UR	1015	1.157		1090
UR	996	2.144		1058
0	0		0	0
no UR	0		0	0
no UR	0		0	0
no UR	0		0	0
no UR	0		0	0
no UR	0		0	0
no UR	0		0	0
0	0		0	0
0	0		0	0
UR	1046	1.148		1087
UR	1058	1.141		1098
0	0		0	0
no UR	0		0	0
UR	1047	0.968		1093
UR	1050	1.309		1091
UR	1057	1.303		1100
UR	1056	1.285		1097
UR	1022	1.135		1149
UR	1058	1.631		1096
UR	1037	1.118		1097
UR	1051	1.114		1096
UR	1050	1.061		1102
UR	1052	0.968		1091
UR	1051	0.96		1113
UR	1050	1.137		1091
UR	1041	1.159		1089
UR	1048	0.973		1099
UR	1050	0.947		1099

UR	1030	1.212		1092
UR	1055	1.08		1132
UR	1053	1.393		1097
UR	1032	1.248		1080
UR	1035	1.126		1091
UR	1049	0.98		1093
UR	1036	0.937		1079
UR	1048	0.839		1088
UR	1051	0.957		1096
UR	1051	0.946		1092
UR	1034	0.882		1105
UR	1034	0.755		1108
UR	1049	0.768		1119
UR	1036	0.912		1079
UR	1049	0.852		1125
UR	1034	0.878		1105
UR	1062	0.907		1098
UR	1020	0.982		1043
UR	1038	0.879		1079
UR	1056	0.985		1093
no UR	0		0	0
UR	1033	0.852		1079
UR	1047	0.736		1092
0	0		0	0
UR	1037	0.867		1140
UR	1021	0.919		1108
UR	1053	0.907		1094
UR	1041	0.792		1110
0	0		0	0
UR	1034	0.792		1086
no UR	0		0	0
UR	1015	0.972		1110
UR	1055	0.915		1100
0	0		0	0
no UR	0		0	0
UR	1015	0.814		1030
UR	1054	0.795		1101
UR	1046	0.657		1101
UR	1034	0.718		1088
UR	1056	0.81		1126
UR	1017	0.804		1017
UR	1051	1.015		1144
no UR	0		0	0
no UR	0		0	0
no UR	0		0	0
no UR	0		0	0
no UR	0		0	0
no UR	0		0	0
no UR	0		0	0
0	0		0	0

UR	1059	0.934		1120
UR	1049	0.999		1081
UR	1042	1.097		1076
no UR	0		0	0
0	0		0	0
0	0		0	0
UR	1035	1.168		1075
0	0		0	0
0	0		0	0
UR	1043	0.915		1079
UR	1005	0.979		1088
UR	1047	1.006		1077
UR	1034	1.039		1068
UR	1030	1.106		1060
0	0		0	0
UR	1037	0.845		1071
0	0		0	0
UR	1022	1.026		1073
UR	1030	0.803		1073
UR	1026	0.83		1071
UR	1000	0.749		1096
UR	1030	1.035		1066
UR	1002	0.93		1083
UR	1039	1.205		1110
UR	1037	1.077		1100
UR	1028	1.068		1088
UR	1039	1.237		1090
UR	1018	1.035		1072
UR	1041	1.03		1075
0	0		0	0
0	0		0	0
UR	1029	0.795		1070
UR	1036	1.094		1098
0	0		0	0
UR	1022	0.988		1100
UR	1020	0.635		1056
UR	1028	0.89		1064
UR	1020	0.876		1073
0	0		0	0
UR	1023	0.522		1025
0	0		0	0
UR	1020	0.853		1066
UR	1020	0.86		1032
UR	1024	0.812		1062
UR	1028	1.054		1098
0	0		0	0
UR	1043	0.907		1096
UR	1022	0.907		1081
UR	1034	0.897		1068
0	0		0	0

no UR	0		0	0
0	0		0	0
UR	1036	1.135		1076
no UR	0		0	0
0	0		0	0
0	0		0	0
UR	1028	0.776		1037
0	0		0	0
UR	1030	0.667		1066
UR	1047	0.77		1094
0	0		0	0
0	0		0	0
no UR	0		0	0
0	0		0	0
no UR	0		0	0
no UR	0		0	0
0	0		0	0
no UR	0		0	0
no UR	0		0	0
0	0		0	0
0	0		0	0
UR	1068	1.335		1097
UR	1076	0.902		1120
no UR	0		0	0
no UR	0		0	0
UR	1061	1.006		1093
UR	1072	0.866		1107
UR	1058	1.179		1092
UR	1040	1.146		1072
UR	1043	1.072		1077
UR	1052	0.993		1115
UR	1045	1.038		1076
UR	1029	1.048		1068
UR	1043	1.098		1075
UR	1034	1.195		1107
UR	1059	1.059		1087
UR	1036	0.99		1078
UR	1035	1.06		1071
UR	1041	1.127		1082
UR	1038	1.029		1072
UR	1028	1.146		1063
UR	1063	1.024		1114
UR	1063	0.986		1106
UR	1028	1.017		1079
UR	1018	1.086		1092
UR	1048	1.642		1089
UR	1046	1.058		1082
UR	1053	0.962		1090
UR	1030	1.011		1068
UR	1019	1.066		1071

UR	1032	1.159		1079
UR	1041	1.015		1084
UR	1064	0.865		1100
UR	1060	0.972		1097
UR	1061	1.005		1096
UR	1070	1.108		1121
UR	1056	0.993		1095
UR	1060	1.028		1145
UR	1049	0.961		1085
UR	1040	0.94		1095
no UR	0		0	0
UR	1056	0.31		1062
UR	1053	0.974		1091
UR	1050	0.976		1083
UR	1033	0.507		1065
UR	1028	0.913		1077
0	0		0	0
UR	1013	0.967		1057
UR	1050	0.945		1096
UR	1051	0.975		1097
no UR	0		0	0
UR	1039	1.157		1022
UR	1035	1.128		1082
no UR	0		0	0
no UR	0		0	0
UR	1054	1.02		1109
UR	1057	1.053		1100
UR	1047	1.038		1104
UR	1023	1.059		1075
UR	1054	1.144		1135
UR	1007	1.009		1047
UR	1056	0.835		1060
no UR	0		0	0
no UR	0		0	0
no UR	0		0	0
no UR	0		0	0
no UR	0		0	0
no UR	0		0	0
no UR	0		0	0
no UR	0		0	0
UR	1052	1.08		1086
0	0		0	0
0	0		0	0
no UR	0		0	0
no UR	0		0	0
0	0		0	0
UR	1020	0.697		1049
UR	1001	1.399		1036
no UR	0		0	0
UR	1037	1.713		1064

UR	1024	0.987		1028
UR	1032	0.734		1037
no UR	0		0	0
UR	1030	1.436		1062
0	0		0	0
UR	1032	0.938		1058
UR	1064	0.892		1073
UR	1058	0.908		1062
UR	1049	1.326		1081
UR	1056	1.29		1077
UR	1064	0.783		1066
0	0		0	0
UR	1031	1.694		1062
UR	1049	0.814		1054
0	0		0	0
UR	1026	0.701		1045
UR	1017	0.697		1030
UR	1013	0.662		1029
0	0		0	0
UR	1011	0.786		1028
UR	1009	0.818		1025
UR	1026	1.464		1059
UR	1030	0.742		1066
UR	1013	0.741		1026
UR	1088	0.716		1126
UR	1084	0.594		1131
0	0		0	0
UR	1100	1.169		1135
UR	1064	0.835		1077
UR	1022	0.859		1034
0	0		0	0
UR	1039	1.243		1060
0	0		0	0
UR	1037	0.618		1051
UR	1022	0.677		1043
UR	1005	0.826		1034
UR	1007	0.793		1188
UR	1017	0.663		1030
UR	1041	0.634		1056
UR	1037	0.694		1051
no UR	0		0	0
no UR	0		0	0
UR	1027	0.796		1034
no UR	0		0	0
0	0		0	0
0	0		0	0
UR	1011	0.756		1020
UR	1022	0.749		1110
0	0		0	0
UR	1049	1.308		1094

UR	1024	0.686		1117
UR	1037	0.871		1063
no UR	0		0	0
no UR	0		0	0
no UR	0		0	0
no UR	0		0	0
no UR	0		0	0
no UR	0		0	0
0	0		0	0
no UR	0		0	0
UR	1056	1.463		1084
0	0		0	0
UR	1028	0.942		1077
0	0		0	0
no UR	0		0	0
UR	1030	1.144		1071
UR	1030	0.826		1069
UR	1031	1.008		1069
UR	1026	1.148		1069
UR	1020	0.987		1066
UR	1028	1.074		1073
UR	1024	1.099		1074
UR	1024	1.016		1067
UR	1027	1.004		1066
UR	1032	1.08		1076
UR	1028	1.082		1072
UR	1031	1.077		1097
UR	1013	1.029		1061
UR	1029	1.008		1109
UR	1025	1.09		1068
UR	1014	1.109		1071
UR	1027	1.13		1066
UR	1013	0.873		1067
0	0		0	0
UR	1039	1.15		1081
UR	1038	1.047		1076
0	0		0	0
UR	1031	0.93		1068
0	0		0	0
UR	1029	1.135		1060
UR	1028	1.108		1110
UR	1116	1.233		1196
UR	1002	0.984		1058
UR	1021	0.924		1107
UR	1037	0.913		1041
UR	1069	0.885		1126
UR	1032	0.844		1090
UR	1026	0.919		1069
UR	1031	1.056		1075
UR	1085	1.251		1203

no UR	0		0	0
UR	1032	1.186		1081
UR	1041	0.928		1085
UR	1011	1.256		1043
UR	1023	0.857		1086
UR	1034	1.14		1083
UR	1035	1.056		1084
UR	1020	1.221		1071
UR	1030	0.978		1073
UR	1064	1.169		1110
no UR	0		0	0
UR	1017	1.118		1069
UR	1031	1.08		1074
no UR	0		0	0
no UR	0		0	0
UR	1032	1.037		1074
UR	1043	0.926		1089
UR	1020	0.799		1037
UR	1021	1.08		1059
UR	1036	0.943		1073
0	0		0	0
UR	1002	1.027		1024
no UR	0		0	0
no UR	0		0	0
no UR	0		0	0
no UR	0		0	0
no UR	0		0	0
no UR	0		0	0
no UR	0		0	0
no UR	0		0	0
UR	1066	1.226		1105
UR	1037	1.193		1090
UR	1039	0.97		1119
0	0		0	0
no UR	0		0	0
UR	1049	0.849		1124
0	0		0	0
UR	1049	1.055		1161
UR	1048	1.197		1126
UR	1060	1.091		1154
UR	1038	1.241		1167
UR	1041	1.116		1105
UR	1051	1.179		1155
UR	1058	1.004		1155
UR	1057	0.862		1139
UR	1052	1.086		1149
0	0		0	0
UR	1052	1.076		1143
UR	1062	1.018		1143
UR	1049	1.004		1168

UR	1041	0.963		1110
UR	1054	0.783		1113
UR	1050	0.987		1147
UR	1048	0.96		1147
UR	1055	0.891		1154
UR	1035	0.822		1108
UR	1055	1.034		1160
UR	1045	1.253		1165
UR	1043	1.019		1098
UR	1044	0.775		1112
UR	1054	0.988		1169
UR	1015	0.785		1110
UR	1049	1.014		1122
UR	1028	0.91		1110
0	0		0	0
UR	1052	0.927		1121
UR	1041	0.844		1140
UR	1050	1.019		1139
UR	1047	0.971		1104
UR	1066	0.251		1110
no UR	0		0	0
UR	1045	1.067		1154
UR	1048	0.927		1138
UR	1048	0.955		1117
UR	1046	0.844		1116
UR	1029	0.916		1107
UR	1035	0.889		1103
UR	1040	0.861		1093
UR	1035	0.815		1106
UR	1050	0.958		1117
no UR	0		0	0
UR	1047	1.01		1122
UR	1053	0.948		1140
0	0		0	0
no UR	0		0	0
0	0		0	0
UR	1055	0.917		1113
UR	1045	0.989		1096
UR	1057	0.979		1105
UR	1055	0.857		1122
UR	1051	0.73		1111
UR	1047	0.816		1098
no UR	0		0	0
no UR	0		0	0
no UR	0		0	0
no UR	0		0	0
no UR	0		0	0
no UR	0		0	0
no UR	0		0	0
no UR	0		0	0

UR	1042	1.09		1112
UR	1033	0.832		1091
UR	1054	0.746		1096
no UR	0		0	0
no UR	0		0	0
UR	1043	0.449		1073
UR	1049	0.804		1081
UR	1062	0.564		1088
UR	1037	0.758		1069
UR	1039	0.783		1075
UR	1049	0.394		1092
UR	1043	0.622		1086
UR	1024	0.742		1062
UR	1033	0.905		1080
UR	1023	0.831		1088
UR	1032	0.914		1062
UR	1051	1.098		1079
UR	1030	1.106		1077
UR	1013	1.019		1068
UR	1027	1.114		1081
UR	1033	0.875		1089
UR	1041	0.836		1077
UR	1037	0.8		1062
UR	1042	0.927		1079
UR	1042	0.903		1119
UR	1034	1.033		1075
UR	1010	0.783		1065
UR	1064	0.571		1086
UR	1053	0.725		1090
UR	1049	0.665		1079
UR	1020	0.663		1077
UR	1023	0.836		1079
UR	1044	0.724		1102
UR	1039	0.722		1082
UR	1043	0.891		1096
UR	1030	0.97		1070
UR	1065	0.717		1121
UR	1027	0.691		1084
0	0		0	0
UR	1039	0.866		1111
no UR	0		0	0
UR	1047	0.449		1083
UR	1050	0.736		1085
UR	1041	0.77		1077
UR	1040	0.581		1132
UR	1019	0.796		1075
0	0		0	0
UR	1025	1.206		1077
UR	1041	1.084		1088
UR	1047	1.28		1110

no UR	0		0	0
UR	1044	0.692		1081
UR	1038	0.642		1091
no UR	0		0	0
no UR	0		0	0
UR	1049	0.854		1083
UR	1038	0.925		1083
UR	1028	0.786		1078
UR	1041	0.65		1069
UR	1032	0.685		1087
UR	1029	1.164		1107
UR	1049	0.804		1091
no UR	0		0	0
no UR	0		0	0
no UR	0		0	0
no UR	0		0	0
no UR	0		0	0
no UR	0		0	0
no UR	0		0	0
no UR	0		0	0
UR	1047	0.746		1083
UR	1041	1.307		1086
UR	1043	1.329		1084
no UR	0		0	0
0	0		0	0
UR	1034	0.787		1088
UR	1032	1.188		1102
UR	1048	1.201		1089
UR	1038	1.321		1089
UR	1043	1.174		1092
UR	1023	1.201		1087
UR	1044	1.212		1085
UR	1050	1.024		1095
UR	1034	1.139		1089
UR	1033	1.019		1085
UR	1039	1.054		1088
UR	1048	1.022		1082
UR	1049	1.014		1084
0	0		0	0
UR	1042	1.032		1081
UR	1020	1.029		1077
UR	1011	0.992		1072
0	0		0	0
UR	1052	1.044		1086
UR	1012	1.057		1071
UR	1042	1.037		1082
UR	1034	0.993		1083
UR	1026	0.991		1083
UR	1028	1.058		1089
UR	1032	1.043		1082

UR	1043	1.063		1081
0	0		0	0
UR	1011	0.994		1081
UR	1054	0.874		1086
UR	1046	0.938		1088
UR	1042	0.946		1085
UR	1027	1.015		1092
UR	1044	1.019		1085
0	0		0	0
UR	1030	0.984		1083
no UR	0		0	0
UR	1010	0.989		1074
UR	1051	0.973		1092
UR	1050	1.034		1091
UR	1035	0.946		1102
UR	1055	1.036		1120
no UR	0		0	0
UR	1027	0.98		1078
UR	1023	0.936		1079
UR	1032	1.144		1073
no UR	0		0	0
0	0		0	0
UR	1044	0.993		1092
no UR	0		0	0
no UR	0		0	0
UR	1021	0.922		1077
UR	1020	0.853		1078
UR	1049	0.995		1092
UR	1009	0.936		1078
UR	1032	0.904		1081
UR	1039	0.957		1087
UR	1043	1.022		1081
no UR	0		0	0
no UR	0		0	0
no UR	0		0	0
no UR	0		0	0
no UR	0		0	0
no UR	0		0	0
no UR	0		0	0
no UR	0		0	0
UR	1051	0.929		1091
0	0		0	0
UR	1001	1.251		1098
no UR	0		0	0
no UR	0		0	0
UR	1046	1.171		1086
UR	1046	1.098		1146
UR	1042	1.123		1092
UR	1056	1.157		1113
0	0		0	0

UR	1036	0.975		1108
UR	1040	1.093		1078
UR	1048	1.234		1093
0	0		0	0
UR	1025	1.577		1098
0	0		0	0
UR	1035	1.128		1086
UR	1030	1.08		1068
UR	1041	1.105		1082
UR	1044	1.11		1087
UR	1018	1.066		1090
UR	1030	1.017		1140
UR	1022	1.053		1116
UR	1049	1.102		1097
UR	1035	1.075		1106
UR	1034	0.861		1078
UR	1037	1.021		1087
UR	1041	1.067		1093
UR	1027	1.07		1087
UR	1027	1.017		1070
UR	1041	0.948		1120
UR	1041	1.103		1152
UR	1035	1.103		1076
UR	1040	1.03		1088
UR	1027	1.019		1070
UR	1046	1.007		1087
UR	1041	1.081		1108
UR	1036	1.062		1086
UR	1048	0.654		1109
UR	1042	1.105		1089
no UR	0		0	0
UR	1036	1.237		1116
UR	1043	0.744		1132
UR	1035	1.051		1097
UR	1024	0.699		1118
UR	1044	0.994		1096
UR	1030	1.008		1081
UR	1038	1.066		1079
UR	1032	0.89		1101
UR	1036	1.002		1104
no UR	0		0	0
UR	1040	1.023		1079
0	0		0	0
no UR	0		0	0
no UR	0		0	0
0	0		0	0
UR	1047	1.073		1120
UR	1027	0.944		1098
UR	1046	0.879		1174
UR	1043	0.998		1095

UR	1047	0.696		1118
UR	1036	0.54		1101
no UR	0		0	0
no UR	0		0	0
0	0		0	0
no UR	0		0	0
no UR	0		0	0
no UR	0		0	0
no UR	0		0	0
no UR	0		0	0
UR	1022	0.881		1086
UR	1046	0.895		1086
UR	1046	0.949		1091
no UR	0		0	0
0	0		0	0
UR	1042	1.021		1101
UR	1052	1.064		1098
UR	1053	1.022		1110
UR	1045	1.109		1151
UR	1029	0.987		1073
UR	1040	1.019		1095
UR	1038	1.015		1083
UR	1052	0.922		1092
UR	1027	0.881		1070
0	0		0	0
UR	1040	0.877		1080
0	0		0	0
UR	1042	0.828		1084
UR	1049	1.108		1097
UR	1042	0.801		1087
UR	1045	0.76		1095
UR	1032	0.779		1083
UR	1045	0.868		1085
UR	1016	0.827		1071
UR	1039	0.85		1087
UR	1046	0.855		1101
UR	1044	0.846		1083
UR	1033	1.119		1090
UR	1014	0.859		1072
UR	1040	0.781		1081
UR	1044	0.729		1091
UR	1045	0.757		1086
UR	1041	0.866		1078
UR	1033	0.812		1084
UR	1026	0.879		1043
UR	1040	0.801		1081
UR	1035	0.785		1088
UR	1045	0.854		1110
UR	1032	0.741		1072
UR	1057	0.688		1130

no UR	0		0	0
UR	1063	0.87		1168
UR	1040	0.941		1128
UR	1037	0.741		1082
UR	1035	0.775		1083
UR	1027	0.958		1070
UR	1036	0.976		1106
UR	1044	0.957		1112
UR	1035	0.923		1110
UR	1039	0.913		1139
0	0		0	0
UR	1023	0.821		1078
UR	1032	0.948		1069
no UR	0		0	0
no UR	0		0	0
UR	1045	1.054		1137
UR	1051	1.053		1123
UR	1048	1.037		1094
UR	1036	1.157		1135
UR	1037	1.121		1074
UR	1028	1.12		1110
UR	1037	0.854		1069
0	0		0	0
no UR	0		0	0
no UR	0		0	0
no UR	0		0	0
no UR	0		0	0
no UR	0		0	0
no UR	0		0	0
no UR	0		0	0
UR	1042	0.792		1079
UR	1053	1.195		1091
UR	1058	0.989		1096
no UR	0		0	0
no UR	0		0	0
UR	988	1.244		1077
0	0		0	0
0	0		0	0
UR	1055	1.063		1086
UR	1043	1.216		1077
UR	1031	1.26		1096
UR	1055	1.196		1089
0	0		0	0
UR	1012	1.214		1088
0	0		0	0
UR	1054	0.479		1117
UR	1058	0.737		1084
UR	1059	0.8		1095
UR	1045	0.863		1135
UR	1081	0.897		1124

UR	1054	1.015		1087
0	0		0	0
UR	1043	0.957		1085
UR	1056	0.82		1094
0	0		0	0
UR	1049	0.832		1097
0	0		0	0
0	0		0	0
0	0		0	0
UR	1053	0.74		1127
UR	1046	0.752		1084
UR	1052	0.672		1099
0	0		0	0
UR	1040	0.75		1082
UR	1051	0.574		1086
UR	1032	0.676		1067
0	0		0	0
UR	1017	0.659		1079
UR	1011	0.779		1066
UR	1042	0.514		1075
no UR	0		0	0
UR	1051	0.553		1092
UR	1053	0.725		1107
UR	1047	0.811		1083
UR	1046	0.738		1102
UR	1051	0.655		1085
0	0		0	0
UR	1043	0.836		1111
UR	1066	0.611		1151
UR	1059	0.524		1121
no UR	0		0	0
UR	1059	0.602		1092
UR	1041	0.487		1099
0	0		0	0
no UR	0		0	0
0	0		0	0
UR	1042	0.671		1088
UR	1051	0.557		1077
UR	1035	0.634		1081
UR	1043	0.551		1111
UR	1023	0.629		1077
UR	1061	0.736		1100
no UR	0		0	0
no UR	0		0	0
0	0		0	0
no UR	0		0	0
no UR	0		0	0
no UR	0		0	0
no UR	0		0	0
no UR	0		0	0

0	0		0	0
UR	1051	1.327		1092
UR	1032	1.682		1069
no UR	0		0	0
no UR	0		0	0
UR	1049	1.395		1081
UR	1034	1.377		1073
UR	1032	1.277		1066
UR	1058	1.143		1069
UR	1037	1.418		1066
UR	1043	1.792		1083
UR	1026	1.31		1065
UR	1017	1.027		1072
UR	1030	0.888		1056
UR	1028	0.758		1090
UR	1024	0.926		1090
UR	1028	0.81		1085
UR	1032	0.956		1074
UR	1027	0.971		1078
UR	1030	1.091		1079
UR	1023	1.215		1078
UR	1024	0.976		1073
UR	1028	1.176		1081
UR	1030	1.122		1090
UR	1031	1.178		1088
UR	1041	0.626		1098
UR	1001	1.018		1085
UR	1028	0.866		1110
UR	1029	1.041		1101
UR	1024	0.906		1069
UR	1026	0.835		1092
UR	1047	1.1		1094
UR	1009	0.987		1077
UR	1027	0.869		1070
UR	1001	1.005		1113
UR	1010	1.015		1076
UR	1026	1.068		1076
UR	1037	0.666		1049
UR	1041	1.024		1094
UR	1028	1.101		1074
no UR	0		0	0
UR	1020	0.771		1086
UR	1032	0.801		1110
UR	1044	0.876		1110
UR	1030	1.026		1100
UR	1028	1.187		1079
UR	1028	0.862		1090
UR	1028	0.945		1085
UR	1032	0.411		1073
UR	1034	1.252		1102

no UR	0		0	0
UR	1016	1.063		1078
UR	1028	1.021		1075
no UR	0		0	0
no UR	0		0	0
UR	1030	0.955		1075
UR	1027	1.053		1094
UR	1037	1.011		1098
UR	1037	1.051		1104
UR	1024	0.791		1079
UR	1029	1.056		1080
UR	1009	1.254		1102
no UR	0		0	0
no UR	0		0	0
no UR	0		0	0
no UR	0		0	0
no UR	0		0	0
no UR	0		0	0
no UR	0		0	0
no UR	0		0	0
UR	1045	1.52		1098
UR	1063	1.233		1096
UR	1058	1.083		1099
no UR	0		0	0
no UR	0		0	0
UR	1032	1.38		1072
UR	1054	1.224		1090
UR	1044	1.167		1084
UR	1051	1.205		1087
0	0		0	0
0	0		0	0
UR	1050	0.946		1084
UR	1041	0.957		1083
UR	1023	1.407		1074
UR	1005	0.828		1088
UR	1059	0.973		1092
UR	1046	1.129		1087
UR	1049	0.823		1083
0	0		0	0
UR	1041	0.88		1083
UR	1028	0.865		1077
0	0		0	0
UR	1025	1.136		1084
UR	1045	0.922		1088
UR	1053	1.085		1090
UR	1050	0.921		1097
UR	1045	0.97		1106
UR	1049	1.028		1089
UR	1064	1.305		1110
UR	1052	1.053		1111

UR	1053	1.127		1103
UR	1049	0.974		1100
UR	1032	0.909		1062
UR	1049	0.941		1099
UR	1025	1.124		1067
0	0		0	0
UR	1043	1.059		1082
UR	1053	0.968		1094
UR	1032	0.832		1048
UR	1058	0.891		1120
no UR	0		0	0
UR	1053	0.982		1091
UR	1050	1.033		1092
UR	1043	0.961		1088
UR	1046	1.029		1088
UR	1059	1.033		1108
UR	1053	1.064		1111
UR	1041	1.19		1098
UR	1052	1.071		1113
UR	1022	0.827		1088
no UR	0		0	0
UR	1052	1.07		1108
0	0		0	0
no UR	0		0	0
no UR	0		0	0
UR	1035	0.989		1093
UR	1043	0.826		1110
UR	1037	0.851		1093
UR	1014	0.743		1045
UR	1047	1.123		1108
UR	1070	0.722		1115
UR	1058	1.115		1079
no UR	0		0	0
no UR	0		0	0
no UR	0		0	0
no UR	0		0	0
no UR	0		0	0
no UR	0		0	0
no UR	0		0	0
0	0		0	0
0	0		0	0
UR	1031	1.238		1131
0	0		0	0
no UR	0		0	0
no UR	0		0	0
UR	1058	1.179		1143
UR	1039	1.532		1128
UR	1036	1.459		1127
0	0		0	0
UR	1054	1.243		1172

UR	1037	1.398		1130
UR	1041	1.155		1124
UR	1060		1	1110
0	0		0	0
UR	1083	0.903		1152
0	0		0	0
UR	1002	0.849		1083
0	0		0	0
UR	1036	1.164		1146
UR	1039	0.934		1110
UR	1060	0.647		1134
UR	1043	1.406		1160
UR	1045	1.027		1132
0	0		0	0
UR	1051	0.857		1110
UR	1037	0.967		1110
UR	1064	0.826		1110
0	0		0	0
UR	1073	0.745		1110
UR	1071	0.925		1120
UR	1028	0.725		1094
UR	1051	1.125		1160
UR	1041	0.815		1098
UR	1038	1.578		1153
UR	1054	1.007		1110
UR	1064	0.979		1143
UR	1029	1.264		1120
UR	1032	1.15		1118
UR	1002	0.852		1110
UR	1049	0.705		1115
no UR	0		0	0
UR	1005	1.176		1110
UR	1005	0.951		1110
UR	1060	0.826		1110
UR	1059	0.857		1114
UR	1045	0.9		1110
0	0		0	0
UR	998	0.86		1110
UR	1066	0.773		1120
0	0		0	0
no UR	0		0	0
UR	1054	0.904		1110
UR	1051	0.701		1110
0	0		0	0
no UR	0		0	0
UR	1026	0.985		1110
UR	1037	0.935		1110
UR	1047	0.777		1110
0	0		0	0
UR	1051	1.144		1110

UR	1009	0.681	1110
UR	1034	0.809	1110
no UR	0	0	0
no UR	0	0	0
no UR	0	0	0
no UR	0	0	0
no UR	0	0	0
no UR	0	0	0
no UR	0	0	0
no UR	0	0	0
UR	1046	1.354	1152
UR	1062	1.055	1137
UR	1048	1.092	1122
no UR	0	0	0
UR	1062	1.108	1124
UR	1040	1.087	1105
UR	1057	0.953	1122
UR	1032	0.966	1108
UR	1051	1.084	1107
UR	1039	1.098	1098
UR	1036	1.228	1090
UR	1009	0.98	1088
UR	1046	1.128	1107
UR	1043	1.062	1114
UR	1017	1.01	1079
UR	1039	0.969	1103
UR	1001	1.057	1079
UR	1007	1.162	1092
UR	1001	1.166	1086
UR	1026	1.035	1085
UR	1040	0.955	1101
0	0	0	0
UR	1041	1.081	1105
UR	1037	1.023	1102
UR	1032	1.081	1080
UR	1032	1.049	1100
UR	1047	1.015	1097
UR	1051	0.983	1110
UR	1051	1.005	1113
UR	1026	0.951	1106
UR	1055	1.049	1116
UR	1042	0.98	1128
UR	1046	1.172	1141
0	0	0	0
UR	1034	1.218	1108
UR	1060	1.057	1126
UR	1028	1.11	1087
UR	1057	0.994	1128
UR	1043	0.991	1103
UR	1044	0.933	1118

UR	1023	0.973		1110
UR	1020	0.899		1098
UR	1049	0.998		1135
0	0		0	0
UR	1030	0.937		1098
UR	1043	1.028		1122
UR	1031	1.022		1100
UR	1046	0.524		1116
UR	1060	0.964		1128
UR	1059	0.943		1138
no UR	0		0	0
UR	1045	1.192		1115
UR	1047	1.212		1105
UR	1044	0.94		1116
UR	1030	1.013		1087
UR	1039	1.165		1100
UR	1059	0.886		1128
UR	1055	1.117		1110
UR	1056	1.005		1126
UR	1005	0.891		1073
no UR	0		0	0
UR	1029	1.034		1072
UR	1011	0.69		1096
UR	1020	1.022		1082
UR	1045	1.004		1110
UR	1045	0.956		1115
UR	1041	0.9		1094
UR	1038	0.965		1127
UR	1037	0.822		1110
UR	1045	1.064		1099
no UR	0		0	0
no UR	0		0	0
UR	1028	0.871		1094
no UR	0		0	0
no UR	0		0	0
UR	1032	1.083		1110
UR	1035	1.083		1098
no UR	0		0	0
no UR	0		0	0
UR	1041	1.2		1077
no UR	0		0	0
UR	1046	1.045		1106
no UR	0		0	0
UR	1049	1.039		1125
no UR	0		0	0
UR	1060	0.967		1128
no UR	0		0	0
no UR	0		0	0
UR	1039	1.01		1093
no UR	0		0	0

no UR	0		0	0
no UR	0		0	0
UR	1045	1.047		1126
UR	1051	0.474		1132
UR	1037	1.039		1096
UR	1037	1.038		1100
no UR	0		0	0
no UR	0		0	0
no UR	0		0	0
no UR	0		0	0
UR	1050	1.155		1099
0	0		0	0
UR	1099	1.292		1166
no UR	0		0	0
0	0		0	0
UR	1074	1.002		1103
UR	1069	1.064		1110
0	0		0	0
UR	1071	0.968		1103
UR	1075	1.301		1110
UR	1069	1.044		1119
UR	1043	1.226		1105
UR	1069	0.883		1124
UR	1055	0.932		1104
UR	1059	1.129		1098
UR	1052	1.024		1101
UR	1022	0.656		1075
UR	1058	1.174		1117
UR	1030	1.584		1084
UR	1089	1.159		1137
UR	1045	1.347		1090
UR	1068	1.159		1103
UR	1060	1.265		1106
UR	1051	1.55		1092
UR	1059	1.019		1110
UR	1060	0.94		1110
UR	1058	1.061		1103
UR	1058	0.946		1110
0	0		0	0
UR	1052	1.158		1105
UR	1080	0.876		1130
UR	1052	0.88		1105
UR	1060	0.903		1106
UR	1054	1.119		1110
UR	1055	0.977		1114
UR	1039	1.018		1104
UR	1063	1.392		1109
0	0		0	0
UR	1065	1.122		1103
UR	1054	0.988		1120

UR	1062	0.865		1112
UR	1062	0.766		1115
UR	1077	0.932		1139
UR	1055	1.063		1110
UR	1060	1.119		1109
UR	1068	0.96		1125
UR	1066	0.926		1134
UR	1066	1.032		1109
UR	1070	1.102		1143
UR	1027	0.975		1121
no UR	0		0	0
UR	1083	1.047		1184
UR	1057	1.24		1145
UR	1062	1.256		1125
UR	1071	1.116		1125
UR	1057	1.076		1113
UR	1084	0.764		1139
UR	1067	0.953		1113
UR	1050	1.337		1091
UR	1083	0.831		1125
no UR	0		0	0
0	0		0	0
UR	1071	0.956		1140
UR	1068	0.918		1117
UR	1063	0.981		1104
UR	1057	0.969		1104
UR	1069	1.074		1116
UR	1054	1.001		1135
UR	1060	0.895		1124
UR	1049	1.008		1128
no UR	0		0	0
no UR	0		0	0
UR	1060	0.871		1139
no UR	0		0	0
no UR	0		0	0
UR	1054	0.986		1135
UR	1058	0.99		1143
no UR	0		0	0
0	0		0	0
UR	1056	1.02		1099
no UR	0		0	0
UR	1064	0.947		1110
no UR	0		0	0
UR	1044	1.178		1095
no UR	0		0	0
UR	1061	1.113		1105
no UR	0		0	0
no UR	0		0	0
UR	1052	1.101		1091
no UR	0		0	0

no UR	0		0	0
no UR	0		0	0
UR	1055	1.209		1094
UR	1053	1.079		1095
UR	1051	1.115		1087
UR	1058	0.913		1110
no UR	0		0	0
no UR	0		0	0
no UR	0		0	0
0	0		0	0
UR	1059	1.087		1096
UR	1045	1.252		1081
UR	1037	0.775		1075
0	0		0	0
UR	1040	1.004		1085
UR	1048	0.969		1082
UR	1050	1.123		1090
UR	1005	1.184		1083
UR	1025	1.19		1074
UR	1041	1.241		1078
UR	1044	1.239		1077
UR	1047	1.13		1081
UR	1035	1.177		1100
UR	1046	1.387		1082
UR	1043	1.162		1081
UR	1049	1.5		1088
UR	1017	1.358		1070
UR	1028	0.816		1074
UR	1038	0.944		1074
UR	1045	0.925		1086
UR	1041	0.91		1081
UR	1033	1.02		1090
UR	1035	1.246		1078
UR	1030	1.045		1079
UR	1030	0.893		1084
UR	1046	1.084		1107
UR	1037	1.043		1081
UR	1026	1.217		1084
UR	1044	1.353		1086
UR	1025	1.119		1086
UR	1024	1.225		1074
UR	1041	1.298		1077
UR	1021	1.116		1085
UR	1039	0.959		1075
UR	1048	0.759		1098
UR	1024	0.9		1079
UR	1039	0.995		1083
UR	1025	0.96		1071
UR	1048	1.053		1095
0	0		0	0

UR	1040	1.134		1137
UR	1039	1.106		1085
UR	1045	1.051		1085
UR	1035	0.873		1112
UR	1045	1.078		1103
UR	1046	1.065		1114
UR	1039	1.145		1103
UR	1050	0.981		1104
UR	1037	1.085		1087
UR	1036	1.216		1092
no UR	0		0	0
UR	1039	1.054		1084
UR	1031	1.209		1086
UR	1051	1.09		1075
0	0		0	0
UR	1032	1.071		1079
UR	1044	1.08		1109
UR	1045	0.827		1115
UR	1032	0.499		1111
UR	1049	0.726		1109
no UR	0		0	0
UR	1045	0.881		1093
0	0		0	0
0	0		0	0
UR	1025	1.296		1078
UR	1036	0.99		1074
UR	1037	1.125		1098
UR	1040	0.967		1098
UR	1034	0.991		1088
UR	1041	0.976		1102
no UR	0		0	0
no UR	0		0	0
UR	1025	1.06		1091
0	0		0	0
0	0		0	0
UR	1041	0.852		1092
UR	1042	0.972		1084
0	0		0	0
0	0		0	0
UR	1048	0.929		1094
no UR	0		0	0
UR	1039	0.944		1111
no UR	0		0	0
UR	1018	0.97		1090
no UR	0		0	0
UR	1032	1.09		1075
no UR	0		0	0
no UR	0		0	0
UR	1038	1.159		1090
no UR	0		0	0

no UR	0		0	0
no UR	0		0	0
UR	1041	1.347		1077
UR	1049	1.388		1086
UR	1022	1.318		1077
UR	1027	1.279		1083
no UR	0		0	0
0	0		0	0
no UR	0		0	0
no UR	0		0	0
UR	1044	0.79		1077
UR	1022	1.049		1129
no UR	0		0	0
no UR	0		0	0
UR	1057	0.998		1103
UR	1032	0.858		1075
UR	1042	0.822		1082
UR	1028	0.767		1110
UR	1051	0.618		1124
UR	1037	0.49		1110
UR	1045	0.79		1097
0	0		0	0
0	0		0	0
UR	1026	0.923		1098
UR	1111	1.105		1177
UR	1007	1.293		1087
UR	1017	1.038		1106
UR	1086	1.408		1164
UR	1051	1.177		1092
UR	1013	0.918		1030
UR	1029	1.047		1113
UR	1049	0.868		1118
UR	1042	1.521		1092
UR	1042	0.973		1110
UR	1020	0.9		1090
UR	1056	1.265		1110
UR	1037	0.883		1120
UR	1022	1.474		1062
UR	1034	1.504		1077
UR	1047	0.868		1110
UR	1034	1.108		1054
UR	1056	0.779		1073
UR	1058	0.528		1128
UR	1055	1.1		1101
UR	1036	0.925		1104
UR	1029	1.038		1098
UR	1032	0.869		1086
UR	1043	0.347		1075
UR	1037	1.081		1090
UR	1029	1.069		1070

UR	1036	1.057		1091
UR	1025	1.124		1079
UR	1051	0.863		1110
UR	1028	1.062		1049
UR	1041	0.803		1101
UR	1046	1.147		1097
UR	1015	1.08		1045
UR	1027	1.214		1083
UR	1037	1.186		1049
UR	1032	1.268		1114
no UR	0		0	0
UR	1029	1.347		1076
UR	1045	1.236		1115
UR	1038	1.296		1082
UR	1034	1.126		1076
UR	1015	1.769		1039
UR	1028	1.009		1039
0	0		0	0
UR	1024	1.1		1110
UR	1024	0.93		1030
0	0		0	0
0	0		0	0
UR	1041	1.463		1094
UR	1039	1.051		1094
UR	1058	0.969		1126
UR	1003	0.985		1062
UR	1022	0.964		1045
UR	1051	0.999		1110
UR	1051	0.8		1130
UR	1020	0.907		1056
no UR	0		0	0
no UR	0		0	0
UR	1041	1.013		1105
0	0		0	0
no UR	0		0	0
UR	1046	1.033		1102
UR	1037	1.149		1098
0	0		0	0
no UR	0		0	0
UR	1041	1.06		1094
0	0		0	0
UR	1026	1.142		1067
no UR	0		0	0
UR	1027	1.025		1077
no UR	0		0	0
UR	1053	1.046		1110
no UR	0		0	0
no UR	0		0	0
UR	1043	1.115		1105
0	0		0	0

no UR	0		0	0
no UR	0		0	0
UR	1050	1.005		1130
UR	1051	1.034		1102
UR	1053	1.183		1104
UR	1037	1.006		1108
no UR	0		0	0
no UR	0		0	0
no UR	0		0	0
no UR	0		0	0
UR	1047	0.745		1110
0	0		0	0
UR	1063	0.727		1107
no UR	0		0	0
UR	1051	1.256		1091
UR	1058	0.63		1092
UR	1007	0.49		1069
UR	1062	0.743		1110
UR	1047	0.562		1139
UR	1045	0.814		1092
UR	1067	0.537		1138
UR	1051	0.633		1100
0	0		0	0
UR	1056	1.018		1097
0	0		0	0
UR	1041	1.485		1122
UR	1042	1.211		1091
UR	1033	1.25		1092
UR	1049	0.65		1128
UR	1039	1.155		1093
UR	1049	1.063		1096
UR	1046	1.104		1089
UR	1041	1.061		1090
UR	1033	0.931		1108
UR	1025	1.048		1104
UR	1036	0.932		1101
UR	1050	0.727		1118
0	0		0	0
UR	1038	1.064		1105
UR	1030	1.019		1085
UR	1045	1.187		1086
UR	1046	1.157		1100
0	0		0	0
UR	1036	1.031		1096
UR	1031	1.119		1096
UR	1024	0.773		1116
UR	1037	1.107		1105
UR	1038	0.898		1127
UR	1047	0.938		1133
UR	1037	1.027		1098

UR	1021	1.185		1097
UR	1037	0.982		1107
UR	1041	0.978		1110
UR	1047	0.946		1104
UR	1035	1.129		1088
UR	1027	1.034		1105
UR	1040	1.058		1095
UR	1028	1.153		1088
UR	1039	1.14		1103
UR	1037	1.162		1090
no UR	0		0	0
UR	1048	1.043		1104
UR	1039	1.013		1110
UR	1047	1.087		1092
UR	1048	1.073		1102
UR	1032	1.061		1094
UR	1038	1.115		1096
UR	1042	1.056		1102
0	0		0	0
UR	1039	0.77		1110
0	0		0	0
UR	1053	0.945		1111
UR	1039	0.675		1112
0	0		0	0
UR	1038	1.079		1099
UR	1042	1.087		1101
UR	1043	0.813		1111
UR	1034	1.147		1110
UR	1042	1.006		1103
UR	1039	0.927		1104
no UR	0		0	0
no UR	0		0	0
UR	1037	0.956		1105
no UR	0		0	0
no UR	0		0	0
UR	1049	1.162		1110
UR	1045	0.785		1110
no UR	0		0	0
no UR	0		0	0
UR	1043	1.142		1106
0	0		0	0
UR	1027	1.095		1095
no UR	0		0	0
UR	1033	1.317		1082
no UR	0		0	0
UR	1046	0.862		1101
no UR	0		0	0
no UR	0		0	0
UR	1041	1.165		1095
no UR	0		0	0

no UR	0		0	0
no UR	0		0	0
UR	1039	1.348		1093
UR	1040	1.249		1100
UR	1043	1.193		1096
UR	1042	1.307		1086
no UR	0		0	0
no UR	0		0	0
no UR	0		0	0
no UR	0		0	0
0	0		0	0
0	0		0	0
UR	1051	1.079		1105
no UR	0		0	0
UR	1066	1.1		1130
UR	1056	1.072		1110
0	0		0	0
UR	1047	1.086		1089
UR	1046	1.05		1092
UR	1048	1.065		1092
UR	1046	1.078		1097
UR	1042	1.045		1084
UR	1051	1.099		1101
0	0		0	0
UR	1040	1.064		1108
UR	1047	1.106		1101
UR	1039	1.182		1121
UR	1039	1.02		1125
UR	1039	1.051		1129
0	0		0	0
UR	1036	1.087		1137
0	0		0	0
UR	1050	0.988		1103
UR	1048	0.891		1134
UR	1056	0.89		1097
UR	1054	1.107		1136
0	0		0	0
UR	1052	0.996		1121
0	0		0	0
UR	1048	1.07		1090
UR	1037	1.022		1091
UR	1052	1.158		1101
UR	1008	0.914		1100
UR	1060	0.985		1094
UR	1043	0.94		1112
UR	1046	1.008		1084
UR	1005	1.025		1090
UR	1046	0.878		1092
UR	1037	0.938		1093
0	0		0	0

0	0		0	0
UR	1045	0.933		1096
0	0		0	0
UR	1047	0.918		1085
UR	1039	1.046		1084
UR	1039	0.985		1084
UR	1045	0.986		1088
UR	1037	0.962		1093
UR	1046	0.943		1088
UR	1026	0.97		1108
no UR	0		0	0
UR	1044	1.035		1090
UR	1029	1.03		1079
UR	1045	1.048		1086
UR	1046	0.952		1089
UR	1045	1.182		1093
UR	1045	1.032		1090
0	0		0	0
UR	1049	0.987		1111
UR	1025	1.063		1112
no UR	0		0	0
UR	1048	0.969		1092
UR	1046	1.026		1094
UR	1062	0.816		1171
UR	1013	0.602		1075
0	0		0	0
UR	1013	0.91		1131
UR	1052	1.2		1108
UR	1043	1.026		1103
UR	1054	1.117		1088
no UR	0		0	0
no UR	0		0	0
UR	1045	1.254		1125
no UR	0		0	0
no UR	0		0	0
UR	1046	1.091		1097
UR	1044	0.906		1108
no UR	0		0	0
no UR	0		0	0
UR	1034	1.155		1111
0	0		0	0
UR	1042	1.13		1077
no UR	0		0	0
UR	1045	1.199		1103
no UR	0		0	0
UR	1045	1.107		1158
no UR	0		0	0
no UR	0		0	0
UR	1044	1.128		1086
no UR	0		0	0

no UR	0		0	0
0	0		0	0
UR	1035	1.04		1099
UR	1048	1.124		1097
UR	1044	1.045		1090
UR	1029	1.075		1125
no UR	0		0	0
no UR	0		0	0
no UR	0		0	0
no UR	0		0	0
UR	1016	1.766		1086
UR	1028	1.23		1103
UR	1031	1.282		1095
no UR	0		0	0
UR	1040	1.094		1097
UR	1021	1.055		1081
UR	1026	1.241		1092
UR	1037	1.481		1107
UR	1035	1.245		1104
UR	1005	0.88		1110
UR	1038	1.313		1100
UR	1039	1.586		1152
UR	1027	1.552		1156
UR	1036	1.559		1156
UR	1036	1.653		1153
UR	1040	1.385		1121
UR	1036	1.033		1125
UR	1037	1.141		1120
UR	1041	0.897		1128
UR	1040	1.396		1130
UR	1043	0.803		1099
UR	1038	0.773		1121
UR	1039	1.403		1148
UR	1041	1.001		1100
UR	1038	1.36		1149
UR	1029	1.062		1129
UR	1035	1.409		1116
UR	1040	0.977		1115
UR	1028	1.15		1108
UR	1026	1.096		1110
UR	1037	1.338		1125
UR	1033	1.151		1113
UR	1047	1.047		1166
UR	1043	1.112		1136
UR	1054	0.693		1124
UR	1039	0.746		1128
UR	1049	0.738		1124
UR	1014	0.971		1113
UR	1036	1.162		1127
UR	1049	0.702		1108

UR	1051	1.05		1124
UR	1049	0.448		1110
UR	1033	0.924		1100
UR	1031	0.984		1147
UR	1049	0.676		1107
UR	1043	1.088		1118
UR	1035	1.099		1113
UR	1045	0.523		1110
0	0		0	0
UR	1040	1.049		1112
no UR	0		0	0
UR	1037	0.857		1107
UR	1039	0.667		1110
UR	1046	0.539		1113
UR	1021	1.048		1091
UR	1041	0.895		1098
UR	1015	0.408		1106
UR	1069	0.972		1135
UR	1043	0.776		1112
UR	1042	0.701		1112
no UR	0		0	0
UR	1037	0.807		1125
UR	1043	0.695		1110
UR	1044	0.806		1109
UR	1042	0.771		1095
UR	1043	0.833		1094
UR	1038	0.814		1094
UR	1044	0.598		1113
UR	1033	1.022		1101
UR	1036	1.077		1103
0	0		0	0
no UR	0		0	0
UR	1040	1.179		1101
no UR	0		0	0
no UR	0		0	0
UR	1034	1.001		1128
UR	1031	0.724		1102
no UR	0		0	0
no UR	0		0	0
0	0		0	0
no UR	0		0	0
UR	1040	1.045		1116
no UR	0		0	0
UR	1034	1.011		1109
no UR	0		0	0
UR	1044	0.939		1118
no UR	0		0	0
no UR	0		0	0
UR	1043	1.04		1124
no UR	0		0	0

no UR	0		0	0
no UR	0		0	0
UR	1049	0.527		1110
UR	1040	0.838		1107
UR	1032	1.03		1103
UR	1040	0.691		1103
no UR	0		0	0
no UR	0		0	0
no UR	0		0	0
no UR	0		0	0
UR	1051	0.781		1130
0	0		0	0
UR	1045	0.977		1141
no UR	0		0	0
UR	1051	1.005		1097
UR	1064	0.984		1094
UR	1053	1.057		1087
UR	1042	1.046		1092
UR	1058	0.917		1103
UR	1058	0.98		1097
UR	1057	1.184		1091
UR	1043	0.926		1091
UR	1036	1.053		1081
UR	1066	0.805		1124
UR	1049	1.118		1098
UR	1054	1.169		1095
UR	1053	1.216		1089
UR	1058	0.99		1110
UR	1057	1.073		1093
UR	1043	1.016		1088
UR	1046	1.009		1086
UR	1055	0.967		1097
UR	1008	0.767		1085
UR	1012	0.938		1077
UR	1057	1.034		1103
UR	1049	0.955		1089
UR	1055	1.106		1100
UR	1039	1.026		1073
UR	1047	0.87		1093
UR	1053	1.188		1092
UR	1040	0.971		1089
UR	1041	0.922		1092
UR	1055	0.932		1102
UR	1047	1.069		1092
UR	1062	1.228		1105
UR	1075	1.394		1110
UR	1053	0.899		1098
UR	1054	0.896		1090
UR	1059	1.045		1099
UR	1043	1.064		1094

UR	1024	0.868		1051
UR	1052	1.234		1092
UR	1013	0.768		1024
UR	1013	1.005		1032
UR	1030	1.218		1079
UR	1015	0.846		1043
UR	1017	1.201		1075
UR	1050	1.216		1092
UR	1058	0.865		1098
UR	1053	1.198		1099
no UR	0		0	0
UR	1042	1.127		1092
UR	1052	0.985		1102
UR	1032	1.07		1086
UR	1051	1.096		1095
UR	1052	1.313		1090
UR	1064	1.304		1140
UR	1062	0.965		1135
UR	1045	1.125		1094
UR	1047	0.92		1075
no UR	0		0	0
UR	1050	1.298		1097
UR	1054	1.338		1128
UR	1039	1.514		1109
UR	1019	1.149		1087
UR	1038	1.22		1105
UR	1081	0.988		1150
UR	1092	0.987		1157
UR	1056	1.023		1102
UR	1047	1.32		1090
no UR	0		0	0
no UR	0		0	0
UR	1045	1.322		1084
no UR	0		0	0
no UR	0		0	0
UR	1010	1.173		1085
UR	1035	1.176		1101
no UR	0		0	0
no UR	0		0	0
UR	1062	0.858		1109
no UR	0		0	0
UR	1031	0.991		1095
no UR	0		0	0
UR	1056	1.298		1145
no UR	0		0	0
UR	1045	1.033		1110
no UR	0		0	0
no UR	0		0	0
UR	1041	1.241		1097
no UR	0		0	0

no UR	0	0	0
no UR	0	0	0
UR	1059	1.2	1106
UR	1069	1.267	1151
UR	1035	1.37	1113
UR	1062	1.065	1115
no UR	0	0	0
no UR	0	0	0
no UR	0	0	0
no UR	0	0	0
UR	1041	0.986	1177
0	0	0	0
UR	1037	1.025	1101
no UR	0	0	0
UR	1048	1.509	1165
UR	1047	1.468	1169
UR	1033	1.505	1164
UR	1052	1.357	1171
no UR	0	0	0
no UR	0	0	0
UR	1034	1.063	1110
UR	1045	0.84	1115
UR	1058	1.04	1129
UR	1037	1.466	1077
UR	1041	1.225	1117
UR	1028	1.12	1108
UR	1037	1.026	1110
UR	1016	1.034	1099
UR	1038	1.148	1098
UR	1041	1.201	1136
UR	1051	0.611	1110
UR	1034	0.776	1110
UR	1066	0.776	1110
UR	1070	0.458	1139
UR	1037	0.935	1110
UR	1043	1.039	1110
UR	1043	0.946	1110
UR	1037	0.82	1110
UR	1026	0.792	1100
UR	1045	1.085	1133
UR	1043	1.097	1110
UR	1039	1.067	1104
UR	1032	1.024	1094
UR	1032	1.072	1103
UR	1054	0.71	1110
UR	1045	0.916	1098
UR	1034	0.817	1081
UR	1026	1.111	1095
UR	1040	0.967	1106
UR	1009	1.249	1110

UR	1045	0.862		1090
UR	1032	1.091		1097
UR	1044	0.409		1116
UR	1025	1.143		1115
UR	1043	1.064		1110
UR	1034	1.178		1110
UR	1043	0.453		1134
UR	1039	0.943		1110
UR	1028	0.993		1083
UR	1034	1.199		1110
no UR	0		0	0
UR	1028	1.102		1108
UR	1046	0.996		1154
UR	1041	0.945		1110
UR	1039	1.027		1075
UR	1043	1.136		1110
UR	1030	1.09		1090
UR	1045	1.003		1110
UR	1032	0.985		1054
UR	1047	1.285		1086
no UR	0		0	0
UR	1030	1.238		1110
UR	1039	1.118		1056
UR	1009	0.922		1020
UR	1024	0.991		1120
UR	1032	0.857		1073
UR	1011	1.033		1030
UR	1041	1.111		1104
UR	1043	0.978		1110
UR	1022	0.776		1045
no UR	0		0	0
no UR	0		0	0
UR	1002	0.729		1049
no UR	0		0	0
no UR	0		0	0
UR	1026	0.819		1126
UR	1017	0.853		1071
no UR	0		0	0
no UR	0		0	0
UR	1024	1.004		1058
no UR	0		0	0
UR	1056	1.022		1124
no UR	0		0	0
UR	1023	0.72		1034
no UR	0		0	0
UR	1062	0.834		1150
no UR	0		0	0
no UR	0		0	0
UR	1001	0.825		1034
no UR	0		0	0

no UR	0	0	0
no UR	0	0	0
UR	1017	0.456	1109
UR	1032	0.828	1051
UR	1026	0.794	1126
UR	1026	0.86	1075
no UR	0	0	0
no UR	0	0	0
no UR	0	0	0
no UR	0	0	0
UR	1056	1.432	1172
UR	1053	0.814	1136
UR	1058	1.053	1196
no UR	0	0	0
UR	1055	0.989	1122
UR	1034	1.03	1118
UR	1055	0.988	1134
UR	1051	0.976	1129
UR	1064	0.92	1137
UR	1056	0.996	1139
UR	1044	0.943	1124
UR	1055	0.908	1124
UR	1048	0.925	1145
UR	1054	0.911	1127
UR	1050	0.975	1130
UR	1046	0.9	1117
UR	1046	1.103	1113
UR	1039	1.007	1110
UR	1051	0.776	1121
UR	1038	1.063	1119
UR	1042	1.186	1172
UR	1049	0.952	1127
UR	1047	0.918	1116
UR	1054	1.09	1114
UR	1039	1.134	1116
UR	1010	0.804	1106
UR	1041	1.206	1119
UR	1046	1.078	1115
UR	1049	1.16	1118
UR	1050	1.143	1111
UR	1037	1.145	1117
UR	1043	0.695	1105
UR	1056	0.955	1116
UR	1038	0.542	1110
UR	1036	1.343	1129
UR	1042	1.059	1113
UR	1041	1.015	1110
UR	1044	1.136	1121
UR	1042	1.081	1113
UR	1051	1.169	1121

UR	1042	0.807		1115
UR	1051	0.969		1111
UR	1043	0.816		1113
UR	1046	1.051		1107
UR	1026	0.982		1094
UR	1029	0.71		1112
UR	1044	1.327		1160
UR	1046	0.633		1104
0	0		0	0
UR	1030	1.218		1152
no UR	0		0	0
UR	1047	1.358		1131
UR	1045	0.969		1104
UR	1044	1.057		1106
UR	1027	1.101		1114
UR	1049	1.042		1118
UR	1046	0.968		1104
UR	1049	1.193		1128
UR	1040	1.078		1106
UR	1036	0.907		1110
no UR	0		0	0
UR	1035	1.204		1129
UR	1037	0.978		1115
UR	1050	0.929		1108
UR	1049	0.833		1104
UR	1046	1.052		1122
UR	1031	0.904		1101
UR	1041	0.933		1113
UR	1046	1.026		1109
UR	1037	0.657		1105
no UR	0		0	0
no UR	0		0	0
UR	1046	1.042		1108
no UR	0		0	0
no UR	0		0	0
UR	1045	0.99		1106
UR	1047	1.038		1107
no UR	0		0	0
no UR	0		0	0
0	0		0	0
no UR	0		0	0
UR	1049	1.372		1135
no UR	0		0	0
UR	1030	1.238		1126
no UR	0		0	0
UR	1048	1.269		1138
no UR	0		0	0
no UR	0		0	0
UR	1031	1.396		1124
no UR	0		0	0

no UR	0		0	0
no UR	0		0	0
UR	1032	1.212		1108
UR	1049	0.731		1116
UR	1044	1.022		1106
UR	1049	0.892		1107
no UR	0		0	0
no UR	0		0	0
no UR	0		0	0
no UR	0		0	0
UR	1045	0.578		1113
UR	1055	1.31		1092
UR	1047	1.167		1092
no UR	0		0	0
UR	1051	1.161		1083
UR	1045	1.11		1083
UR	1045	1.371		1088
UR	1054	1.124		1084
UR	1055	1.466		1092
0	0		0	0
UR	1029	1.423		1084
UR	1054	1.038		1090
0	0		0	0
UR	1035	1.291		1083
UR	1025	1.084		1071
UR	1056	1.142		1092
0	0		0	0
UR	1037	0.993		1071
UR	1056	1.373		1084
UR	1030	1.124		1084
UR	1059	1.183		1089
UR	1060	0.836		1092
UR	1062	0.925		1120
UR	1047	1.12		1075
UR	1070	1.087		1107
UR	1061	1.145		1100
UR	1058	0.96		1096
UR	1061	0.865		1103
UR	1038	1.071		1077
UR	1032	1.881		1097
UR	1081	0.831		1139
0	0		0	0
UR	1042	0.841		1083
UR	1052	0.998		1085
UR	1001	0.982		1075
UR	1064	0.695		1133
UR	1058	0.977		1095
UR	1064	1.2		1100
0	0		0	0
UR	1037	0.958		1110

UR	1066	1.104		1114
UR	1057	1.014		1094
0	0		0	0
0	0		0	0
0	0		0	0
UR	1055	1.08		1090
UR	1045	0.756		1110
UR	1056	1.126		1096
UR	1063	0.944		1126
UR	1051	1.12		1092
no UR	0		0	0
UR	1061	0.964		1100
UR	1050	1.107		1135
UR	1071	0.741		1137
UR	1053	0.891		1092
UR	1037	0.309		1064
0	0		0	0
UR	1060	1.142		1103
UR	1050	1.103		1095
UR	1074	0.677		1115
no UR	0		0	0
UR	1061	1.319		1100
UR	1055	1.152		1091
0	0		0	0
UR	1064	1.355		1110
0	0		0	0
0	0		0	0
UR	1052	0.924		1089
UR	1030	0.649		1062
UR	1024	0.71		1081
no UR	0		0	0
no UR	0		0	0
0	0		0	0
no UR	0		0	0
no UR	0		0	0
UR	1064	1.169		1105
UR	1051	1.018		1111
0	0		0	0
no UR	0		0	0
UR	1059	1.001		1098
no UR	0		0	0
UR	1057	0.938		1094
no UR	0		0	0
UR	1038	1.151		1083
no UR	0		0	0
0	0		0	0
no UR	0		0	0
no UR	0		0	0
UR	1067	0.815		1115
no UR	0		0	0

no UR	0		0	0
no UR	0		0	0
UR	1063	0.846		1096
UR	1064	1.207		1129
0	0		0	0
UR	1064	1.041		1109
no UR	0		0	0
no UR	0		0	0
no UR	0		0	0
no UR	0		0	0
UR	1055	1.261		1093
UR	1037	1.43		1110
UR	1044	1.417		1112
no UR	0		0	0
UR	1040	1.382		1107
UR	1054	1.369		1108
UR	1054	1.287		1121
UR	1046	1.14		1126
UR	1037	1.346		1142
UR	1048	1.208		1131
UR	1037	1.383		1131
UR	1005	1.189		1132
UR	1044	1.305		1133
UR	1050	0.704		1114
UR	1052	0.774		1113
UR	1042	1.051		1119
UR	1048	0.558		1115
UR	1058	1.165		1107
UR	1040	0.933		1134
UR	1046	1.307		1113
UR	1034	1.039		1122
UR	1040	1.31		1131
UR	1055	1.293		1136
UR	1042	1.329		1128
UR	1039	1.232		1127
UR	1053	0.969		1121
UR	1056	0.306		1115
UR	1040	0.959		1125
UR	1052	1.191		1126
UR	1032	0.395		1107
UR	1042	0.536		1118
UR	1052	0.605		1120
UR	1045	1.003		1126
UR	1038	1.131		1123
UR	1061	0.629		1119
UR	1047	0.58		1115
UR	1051	0.734		1118
UR	1036	1.146		1112
UR	1045	0.562		1110
0	0		0	0

UR	1046	0.633		1108
UR	1048	0.847		1114
UR	1045	0.486		1117
UR	1041	0.409		1118
UR	1044	1.21		1137
UR	1038	0.597		1108
UR	1042	1.542		1123
UR	1044	0.542		1114
UR	1042	0.925		1112
UR	1049	0.588		1109
no UR	0		0	0
UR	1060	1.051		1114
UR	1033	1.743		1142
UR	1044	1.411		1119
UR	1035	0.913		1116
UR	1013	1.126		1112
UR	1051	0.903		1117
0	0		0	0
UR	1041	1.143		1112
UR	1049	0.455		1108
no UR	0		0	0
UR	1036	0.788		1115
UR	1043	1.77		1056
UR	1032	1.482		1083
UR	1047	0.761		1108
UR	1054	0.92		1121
UR	1041	0.432		1117
UR	1032	0.968		1118
UR	1051	0.267		1110
UR	1042	0.701		1117
no UR	0		0	0
no UR	0		0	0
UR	1042	0.731		1121
no UR	0		0	0
no UR	0		0	0
UR	1036	0.977		1098
UR	1029	1.299		1107
no UR	0		0	0
no UR	0		0	0
UR	1045	0.952		1123
no UR	0		0	0
UR	1039	1.096		1114
no UR	0		0	0
UR	1042	1.343		1168
no UR	0		0	0
UR	1041	1.003		1110
no UR	0		0	0
no UR	0		0	0
UR	1036	1.149		1120
no UR	0		0	0

no UR	0		0	0
no UR	0		0	0
UR	1041	1.173		1117
UR	1030	0.813		1123
UR	1048	0.909		1118
UR	1039	0.784		1105
no UR	0		0	0
no UR	0		0	0
no UR	0		0	0
no UR	0		0	0
UR	907	0.951		1110
UR	1036	1.842		1080
UR	1042	1.837		1067
no UR	0		0	0
UR	1029	1.646		1062
UR	1016	1.525		1060
UR	1040	1.378		1064
UR	1037	1.599		1057
UR	1033	1.646		1053
UR	1023	1.378		1060
UR	1043	1.452		1067
UR	1041	1.43		1066
UR	1037	0.984		1055
UR	1031	1.723		1052
UR	1028	1.495		1058
UR	1034	1.265		1061
UR	1025	1.151		1028
UR	1028	0.96		1055
UR	1016	1.532		1031
UR	1019	1.536		1022
UR	1028	1.428		1046
UR	1022	1.525		1037
UR	1043	1.418		1064
UR	1022	1.492		1052
UR	1037	1.382		1061
UR	1031	1.175		1070
UR	1052	0.954		1058
UR	1031	1.253		1040
UR	1031	0.947		1061
no UR	0		0	0
UR	1034	1.139		1070
UR	1043	1.149		1070
UR	1046	0.93		1070
UR	1034	1.273		1082
UR	1055	1.082		1070
UR	1037	0.923		1052
UR	1052	0.885		1064
UR	1037	1.045		1055
UR	1097	1.289		1204
UR	1034	1.074		1040

no UR	0		0	0
UR	1022	0.831		1110
UR	1046	1.089		1046
UR	1031	0.794		1058
UR	1046	0.825		1061
UR	1019	0.591		1058
UR	1022	1.279		1049
UR	1025	1.176		1064
UR	1031	1.437		1052
UR	1034	1.001		1049
no UR	0		0	0
UR	1037	1.098		1061
UR	1055	1.194		1061
UR	1025	1.85		1061
UR	1040	1.803		1055
no UR	0		0	0
0	0			
UR	1031	1.778		1061
UR	1079	0.78		1110
UR	1049	1.268		1064
no UR	0		0	0
UR	1028	0.653		1052
UR	1049	1.064		1246
UR	1046	1.149		1061
UR	1040	1.258		1061
UR	1037	1.377		1055
UR	1046	1.144		1061
UR	1031	1.45		1028
UR	1106	1.345		1189
UR	1028	0.789		1055
no UR	0		0	0
no UR	0		0	0
UR	1061	1.146		1082
no UR	0		0	0
no UR	0		0	0
UR	1043	1.425		1070
UR	1040	0.729		1067
no UR	0		0	0
no UR	0		0	0
0	0			
no UR	0		0	0
no UR	0		0	0
no UR	0		0	0
UR	1046	0.781		1064
no UR	0		0	0
UR	1034	0.4		1055
no UR	0		0	0
0	0			
UR	1031	0.721		1058
no UR	0		0	0

no UR	0	0	0
no UR	0	0	0
UR	1061	1.079	1079
UR	1040	0.949	1064
UR	1058	0.96	1079
UR	1031	0.751	1061
no UR	0	0	0
no UR	0	0	0
no UR	0	0	0
no UR	0	0	0
UR	1048	0.814	1067
UR	1047	0.732	1081
UR	1058	0.892	1085
no UR	0	0	0
UR	1068	0.994	1090
UR	1072	0.755	1095
UR	1061	0.792	1090
UR	1071	0.604	1095
UR	1063	0.79	1089
UR	1059	0.75	1083
UR	1062	0.811	1087
UR	1054	0.747	1081
UR	1070	0.579	1091
UR	1056	0.692	1083
UR	1058	0.606	1083
UR	1043	0.647	1071
UR	1050	0.715	1077
UR	1069	0.607	1092
0	0	0	0
UR	1069	0.689	1100
UR	1062	0.945	1089
UR	1059	0.804	1086
UR	1058	1.208	1093
UR	1048	1.033	1090
UR	1055	1.022	1085
UR	1061	1.055	1093
UR	1052	1.167	1086
UR	1065	1.163	1115
UR	1058	0.832	1101
UR	1062	0.503	1135
UR	1026	1.202	1083
UR	1053	0.775	1088
UR	1028	0.929	1072
UR	1080	0.683	1134
UR	1060	1.15	1092
UR	1046	1.167	1092
UR	1053	1.369	1088
UR	1056	1.256	1096
UR	1053	0.95	1096
UR	1042	0.82	1122

UR	1073	0.582		1189
UR	1084	0.725		1137
UR	1066	1.051		1100
UR	1046	0.981		1098
UR	1057	1.067		1106
UR	1061	0.96		1097
UR	1062	1.124		1092
UR	1063	0.962		1098
UR	1063	0.982		1108
UR	1053	0.981		1103
no UR	0		0	0
UR	1063	1.023		1100
UR	1073	0.505		1177
UR	1042	1.039		1096
UR	1053	1.25		1133
UR	1074	0.643		1120
UR	1066	0.789		1114
UR	1052	1.021		1097
UR	1059	0.916		1100
UR	1060	0.927		1100
no UR	0		0	0
0	0		0	0
UR	1067	0.67		1101
UR	1066	1.098		1109
UR	1063	0.519		1109
UR	1083	0.029		1267
UR	1056	0.862		1101
UR	1069	0.653		1112
UR	1088	0.602		1125
UR	1066	0.767		1112
no UR	0		0	0
no UR	0		0	0
UR	1059	0.718		1105
no UR	0		0	0
no UR	0		0	0
UR	1059	0.925		1102
UR	1064	0.985		1101
no UR	0		0	0
0	0		0	0
UR	1058	0.979		1092
no UR	0		0	0
UR	1058	0.981		1107
no UR	0		0	0
UR	1066	0.425		1116
no UR	0		0	0
UR	1046	0.851		1091
no UR	0		0	0
no UR	0		0	0
UR	1060	0.87		1105
no UR	0		0	0

no UR	0		0	0
no UR	0		0	0
UR	1058	1.214		1093
UR	1057	0.747		1110
UR	1058	1.282		1100
UR	1048	0.848		1095
no UR	0		0	0
no UR	0		0	0
0	0		0	0
no UR	0		0	0
UR	1054	0.936		1088
UR	1079	0.685		1175
UR	1100	0.824		1158
no UR	0		0	0
UR	1051	1.451		1126
UR	1045	2.382		1124
0	0		0	0
UR	1045	2.089		1086
UR	1045	1.939		1089
UR	1064	1.412		1090
UR	1055	1.894		1175
UR	1063	1.762		1115
UR	1037	2.044		1154
UR	1058	1.928		1118
UR	1055	1.766		1129
UR	1032	1.873		1138
UR	1007	2.083		1071
UR	1060	0.094		1120
no UR	0		0	0
UR	1007	1.249		1007
UR	1030	0.143		1100
UR	1065	1.981		1130
UR	1056	1.198		1132
UR	1056	0.518		1120
0	0		0	0
0	0		0	0
UR	1071	1.307		1110
UR	1074	1.012		1104
UR	1040	0.751		1108
UR	1055	1.337		1122
UR	1058	0.332		1110
UR	1070	1.121		1106
no UR	0		0	0
UR	1068	1.328		1109
no UR	0		0	0
UR	1063	1.52		1101
UR	1055	1.128		1109
UR	1062	1.399		1100
no UR	0		0	0
no UR	0		0	0

UR	1076	0.745		1129
UR	1062	0.274		1109
UR	1076	0.767		1124
UR	1060	1.282		1101
UR	1076	0.647		1119
no UR	0		0	0
UR	1045	0.847		1110
no UR	0		0	0
UR	1058	0.416		1110
no UR	0		0	0
no UR	0		0	0
UR	1066	0.809		1124
UR	1067	1.138		1106
UR	1060	0.378		1110
UR	1020	0.556		1083
no UR	0		0	0
no UR	0		0	0
no UR	0		0	0
UR	1054	0.607		1110
UR	1049	0.63		1147
no UR	0		0	0
UR	1044	1.364		1133
UR	1060	0.777		1143
UR	1056	1.326		1130
UR	1046	1.327		1112
UR	1076	0.569		1122
UR	1071	0.744		1104
UR	1060	1.071		1125
UR	1048	0.928		1125
0	0		0	0
no UR	0		0	0
no UR	0		0	0
UR	1041	2.179		1110
no UR	0		0	0
no UR	0		0	0
UR	1049	0.884		1115
0	0		0	0
no UR	0		0	0
no UR	0		0	0
UR	1039	0.067		1110
no UR	0		0	0
UR	1034	0.223		1110
no UR	0		0	0
UR	1037	0.106		1086
no UR	0		0	0
UR	1069	1.557		1185
no UR	0		0	0
no UR	0		0	0
UR	1073	0.721		1132
no UR	0		0	0

no UR	0		0	0
no UR	0		0	0
UR	1039	2.437		1110
UR	1041	2.103		1110
UR	1050	1.206		1177
UR	1057	0.837		1105
no UR	0		0	0
no UR	0		0	0
no UR	0		0	0
no UR	0		0	0
UR	1037	2.233		1110
0	0		0	0
UR	1027	1.458		1083
no UR	0		0	0
UR	1049	0.709		1058
UR	1022	1.212		1026
UR	1015	1.073		1049
UR	1028	1.016		1062
UR	1049	0.907		1058
UR	1026	0.834		1037
UR	1030	1.311		1066
UR	1039	1.41		1077
UR	1045	0.959		1060
UR	1034	0.812		1056
UR	1014	1.178		1025
UR	1022	1.418		1075
UR	1039	0.929		1056
UR	1033	1.076		1055
UR	1006	1.042		1026
UR	1015	0.943		1023
UR	1032	0.836		1045
UR	1107	0.822		1134
UR	1022	1.06		1083
UR	1061	1.214		1096
UR	1060	0.694		1066
UR	1029	0.756		1036
UR	1034	0.874		1041
UR	1047	1.171		1078
UR	1002	1.088		1032
UR	1018	0.735		1027
UR	1116	0.774		1146
UR	1039	0.664		1044
UR	1037	1.103		1070
UR	1039	0.822		1047
UR	1017	0.649		1026
0	0		0	0
UR	1037	0.779		1039
UR	1034	1.25		1074
UR	1023	0.784		1029
UR	1032	0.693		1041

UR	1032	0.762		1039
UR	1037	1.138		1076
UR	1028	0.714		1037
UR	1020	1.118		1056
UR	1017	0.911		1024
UR	1016	1.197		1032
UR	1051	0.676		1056
UR	1053	1.128		1094
UR	1020	0.853		1023
UR	1039	1.138		1087
no UR	0		0	0
UR	1028	1.076		1062
UR	1020	0.921		1043
UR	1030	0.918		1028
UR	1048	0.608		1056
UR	1001	0.858		1039
UR	1013	0.83		1024
0	0		0	0
UR	1047	0.723		1051
UR	1022	0.895		1058
no UR	0		0	0
UR	1034	0.806		1043
UR	1032	1.121		1070
UR	1040	1.103		1081
UR	1028	0.814		1043
UR	1034	1.111		1073
UR	1038	1.119		1087
UR	1045	1.111		1083
UR	1039	1.13		1165
UR	1034	0.762		1039
no UR	0		0	0
no UR	0		0	0
UR	1037	1.211		1078
no UR	0		0	0
no UR	0		0	0
UR	1013	1.117		1066
UR	1034	0.76		1154
no UR	0		0	0
no UR	0		0	0
UR	1022	0.711		1034
no UR	0		0	0
UR	1102	0.745		1131
no UR	0		0	0
UR	1033	1.15		1078
no UR	0		0	0
UR	1035	1.039		1070
no UR	0		0	0
no UR	0		0	0
UR	1035	1.181		1077
no UR	0		0	0

no UR	0		0	0
no UR	0		0	0
UR	1040	1.085		1087
UR	1032	1.027		1039
UR	1026	1.057		1070
UR	1038	1.183		1083
no UR	0		0	0
no UR	0		0	0
0	0		0	0
no UR	0		0	0
UR	1030	1.094		1090
UR	1035	1.493		1089
UR	1036	1.362		1116
0	0		0	0
UR	1032	1.011		1081
UR	1036	1.034		1104
UR	1039	1.361		1114
UR	1005	0.877		1077
UR	1034	1.6		1121
UR	1018	1.298		1109
UR	1041	1.173		1110
UR	1041	1.51		1117
UR	1013	0.984		1092
UR	996	0.886		1069
UR	1021	1.22		1083
UR	1037	1.255		1110
UR	1033	1.28		1097
UR	1026	1.358		1103
UR	1025	1.232		1107
UR	1030	1.146		1110
UR	1026	1.064		1096
UR	1024	1.924		1079
UR	1045	0.797		1096
UR	1058	1.349		1086
UR	1047	1.052		1110
UR	1037	1.2		1105
UR	1017	0.97		1041
UR	1025	1.368		1096
UR	1033	1.462		1111
UR	1018	0.947		1099
UR	1030	1.253		1100
UR	1022	1.185		1090
UR	1045	1.112		1100
UR	1003	0.896		1069
UR	1036	0.909		1106
UR	1032	1.114		1101
UR	1001	1.239		1039
UR	1051	0.67		1117
UR	1015	1.058		1139
UR	1003	1.195		1036

UR	1043	1.034		1085
UR	1043	0.65		1056
UR	1037	0.994		1110
UR	1013	0.874		1081
UR	1030	0.846		1100
UR	1022	0.66		1110
UR	1023	0.907		1093
UR	1013	0.714		1094
UR	1041	0.771		1088
UR	1044	0.95		1108
no UR	0		0	0
UR	1033	0.686		1036
UR	1020	0.843		1028
UR	1037	0.956		1100
UR	1026	0.681		1051
UR	1017	0.887		1026
UR	1007	0.782		1098
UR	1056	0.853		1156
UR	1035	1.031		1099
UR	1015	0.732		1086
no UR	0		0	0
UR	998	0.767		1058
UR	1039	1.093		1110
UR	1024	0.729		1110
UR	1015	0.804		1110
UR	1051	0.753		1110
UR	1039	0.785		1058
UR	1015	0.988		1110
UR	1041	0.056		1094
0	0		0	0
no UR	0		0	0
no UR	0		0	0
UR	1100	0.325		1117
no UR	0		0	0
no UR	0		0	0
UR	1045	1.11		1112
UR	1011	0.81		1096
no UR	0		0	0
no UR	0		0	0
UR	1068	0.814		1120
no UR	0		0	0
UR	1024	0.586		1110
no UR	0		0	0
UR	1017	0.858		1110
no UR	0		0	0
UR	1039	0.779		1098
no UR	0		0	0
no UR	0		0	0
UR	1056	0.194		1103
0	0		0	0

no UR	0		0	0
no UR	0		0	0
UR	1051	1.145		1119
UR	1030	1.18		1110
UR	1045	1.049		1103
UR	1047	0.654		1110
no UR	0		0	0
no UR	0		0	0
no UR	0		0	0
no UR	0		0	0
UR	1047	0.855		1110
0	0		0	0
UR	1056	1.231		1088
0	0		0	0
0	0		0	0
0	0		0	0
0	0		0	0
no UR	0		0	0
UR	1030	1.08		1049
UR	1064	0.273		1128
UR	1043	0.722		1066
UR	1026	0.637		1062
0	0		0	0
0	0		0	0
UR	1017	0.905		1026
UR	977	-0.014		1022
UR	1039	0.996		1073
UR	1000	-0.311		1026
UR	1015	0.045		1022
UR	1009	0.042		1020
UR	1071	-0.177		1110
UR	1002	-0.223		1028
UR	1022	-0.285		1030
UR	1032	0.887		1075
UR	1002	-0.142		1022
UR	1020	0.451		1039
UR	975	0.11		994
0	0		0	0
no UR	0		0	0
UR	1041	0.656		1073
0	0		0	0
UR	1028	0.714		1043
UR	1039	0.588		1066
UR	1022	0.385		1039
UR	1020	0.46		1024
UR	1015	-0.115		1026
UR	1079	0.127		1126
UR	1077	0.044		1126
UR	1054	0.493		1092
UR	1024	0.833		1054

UR	1077	-0.091		1110
UR	1051	0.689		1077
UR	1086	-0.076		1130
UR	1064	-0.167		1135
UR	1009	-0.133		1135
UR	1045	0.854		1081
0	0		0	0
0	0		0	0
UR	1096	0.234		1203
UR	1088	-0.398		1139
no UR	0		0	0
UR	1015	-0.066		1110
UR	1077	-0.146		1139
UR	1005	-0.037		1030
UR	1041	0.658		1069
UR	1007	0.075		1028
0	0		0	0
UR	1077	-0.286		1100
UR	1094	-0.237		1110
UR	1020	-0.085		1037
no UR	0		0	0
UR	1047	0.802		1069
UR	1024	0.766		1051
UR	1086	-0.254		1110
UR	1030	-0.051		1132
UR	1024		0	1030
UR	1007	-0.23		1069
0	0		0	0
UR	1037	-0.609		1079
UR	1028	-0.394		1066
no UR	0		0	0
no UR	0		0	0
UR	1020	0.119		1034
no UR	0		0	0
no UR	0		0	0
0	0		0	0
UR	1011	0.625		1056
no UR	0		0	0
no UR	0		0	0
UR	1009	-0.265		1064
no UR	0		0	0
UR	1000	0.143		1024
no UR	0		0	0
0	0		0	0
no UR	0		0	0
0	0		0	0
no UR	0		0	0
no UR	0		0	0
UR	1013	-0.292		1081
0	0		0	0

0	0		0	0
no UR	0		0	0
no UR	0		0	0
no UR	0		0	0
UR	1013	-0.002		1110
UR	1060	-0.154		1110
0	0		0	0
no UR	0		0	0
0	0		0	0
no UR	0		0	0
UR	1026	0.907		1073
UR	1028	1.528		1088
UR	1040	1.54		1092
no UR	0		0	0
UR	1054	1.051		1116
UR	1039	1.103		1100
UR	1036	1.321		1084
UR	1043	1.514		1092
UR	1042	1.457		1094
UR	1045	1.399		1096
UR	1044	1.26		1091
UR	1041	1.226		1089
UR	1048	1.011		1106
0	0		0	0
UR	1039	1.207		1091
UR	1048	1.092		1100
UR	1041	1.19		1089
UR	1035	1.656		1155
UR	1040	1.317		1095
UR	1039	1.324		1096
UR	1082	1.047		1157
UR	1044	1.251		1091
UR	1049	1.224		1105
UR	1045	1.401		1093
0	0		0	0
UR	1053	1.312		1087
0	0		0	0
UR	1051	1.182		1091
UR	1044	1.172		1088
UR	1047	1.256		1088
UR	1036	1.103		1088
UR	1053	0.989		1092
UR	1043	1.045		1091
UR	1039	1.121		1092
0	0		0	0
0	0		0	0
UR	1054	1.001		1102
UR	1055	0.643		1089
UR	1054	0.936		1094
UR	1046	0.971		1092

UR	1047	0.906		1094
UR	1046	0.976		1087
UR	1052	1.049		1092
UR	1048	1.076		1090
UR	1035	1.075		1094
UR	1043	0.986		1095
UR	1041	1.034		1093
UR	1039	0.899		1086
UR	1054	0.952		1094
UR	1052	1.01		1105
no UR	0		0	0
UR	1048	0.832		1092
UR	1052	0.728		1109
UR	1041	0.739		1104
UR	1042	0.732		1101
UR	1023	0.772		1093
UR	1052	0.687		1122
UR	1050	0.403		1101
UR	1045	0.164		1135
UR	1052	0.767		1100
no UR	0		0	0
UR	1038	0.857		1087
UR	1024	0.571		1071
UR	1047	0.753		1092
UR	1046	0.716		1100
UR	1019	0.653		1111
UR	1037	0.664		1086
UR	1035	0.579		1101
UR	1015	0.42		1079
UR	1041	0.595		1110
no UR	0		0	0
no UR	0		0	0
UR	1024	0.565		1058
0	0		0	0
no UR	0		0	0
UR	1051	0.805		1092
UR	1047	0.807		1126
no UR	0		0	0
no UR	0		0	0
UR	1050	0.616		1120
no UR	0		0	0
UR	1051	0.723		1164
0	0		0	0
UR	1073	0.961		1112
no UR	0		0	0
UR	1041	0.807		1089
no UR	0		0	0
no UR	0		0	0
UR	1034	0.885		1086
no UR	0		0	0

no UR	0		0	0
no UR	0		0	0
UR	1046	0.813		1094
UR	1056	0.663		1114
UR	1044	0.819		1094
UR	1045	0.76		1089
no UR	0		0	0
no UR	0		0	0
no UR	0		0	0
no UR	0		0	0
UR	1036	0.863		1091
UR	1015	1.127		1043
no UR	0		0	0
UR	1027	1.076		1082
UR	1021	1.329		1070
UR	1010	1.342		1052
UR	1008	1.199		1046
0	0			
UR	1008	1.411		1034
UR	1020	0.997		1065
UR	1011	1.009		1053
UR	1001	1.088		1042
UR	1008	0.929		1064
UR	1012	0.939		1029
UR	1014	0.988		1025
UR	1025	0.877		1074
0	0			
UR	1012	1.132		1046
UR	1018	0.992		1042
0	0			
UR	1005	0.983		1035
UR	1020	1.141		1046
UR	1022	1.139		1048
UR	1008	1.098		1039
UR	1008	1.307		1042
UR	1005	0.975		1031
UR	1008	1.114		1039
UR	1014	1.077		1037
UR	1010	1.019		1046
UR	1014	0.914		1037
0	0			
UR	1012	0.987		1039
UR	1014	0.878		1042
UR	1010	1.043		1042
UR	1008	1.005		1035
UR	1008	1.067		1042
UR	1010	0.889		1057
0	0			
0	0			
UR	1016	0.88		1039

no UR	0		0	0
UR	1012	1.057		1050
UR	1008	1.021		1035
0	0			
UR	1012	0.983		1039
UR	1014	0.91		1050
UR	1010	1.141		1037
UR	1010	0.719		1039
UR	1014	0.904		1044
UR	1014	0.839		1039
no UR	0		0	0
no UR	0		0	0
UR	1017	0.862		1055
no UR	0		0	0
no UR	0		0	0
0	0			
UR	1014	0.979		1046
no UR	0		0	0
no UR	0		0	0
UR	1020	1.033		1062
no UR	0		0	0
UR	1017	0.953		1055
no UR	0		0	0
0	0			
0	0			
UR	1010	1.103		1042
0	0			
no UR	0		0	0
UR	1005	0.961		1039
no UR	0		0	0
no UR	0		0	0
no UR	0		0	0
0	0			
UR	1014	1.194		1044
UR	1012	1.526		1042
0	0			
no UR	0		0	0
0	0			
no UR	0		0	0
no UR	0		0	0
no UR	0		0	0
UR	1049	0.984		1085
no UR	0		0	0
UR	1015	0.824		1042
0	0			
UR	1039	0.864		1079
UR	1027	1.018		1053
UR	1038	0.889		1088
UR	1047	0.905		1085
UR	1012	0.873		1037

UR	1035	0.975		1070
UR	1025	0.81		1050
UR	1016	0.971		1049
UR	1022	0.808		1046
UR	1014	0.851		1044
UR	1030	0.961		1072
UR	1022	0.848		1048
0	0			
UR	1022	0.894		1067
UR	1025	0.888		1057
UR	1042	1.177		1076
UR	1031	0.892		1065
UR	1029	0.932		1086
0	0			
UR	1035	1.017		1067
UR	1018	1.189		1059
UR	1041	1.105		1082
UR	1016	1.168		1039
UR	1033	1.266		1076
UR	1022	1.131		1056
UR	1046	1.24		1076
UR	1018	1.24		1065
UR	1020	1.172		1059
UR	1016	1.114		1059
UR	1018	1.081		1057
UR	1014	1.055		1039
UR	1029	1.113		1064
UR	1024	1.249		1065
UR	1032	0.984		1085
UR	1035	1.136		1092
no UR	0		0	0
UR	1025	1.081		1052
UR	1017	0.98		1045
UR	1016	0.983		1037
0	0			
UR	1026	1.049		1075
UR	1038	1.083		1077
UR	1016	1.191		1057
0	0			
UR	1022	1.022		1052
no UR	0		0	0
no UR	0		0	0
UR	1009	1.144		1037
no UR	0		0	0
no UR	0		0	0
UR	1014	1.037		1039
UR	1043	1.049		1099
no UR	0		0	0
no UR	0		0	0
UR	1032	0.79		1078

no UR	0		0	0
UR	1019	0.878		1057
no UR	0		0	0
UR	1043	0.846		1077
no UR	0		0	0
UR	1042	0.917		1093
no UR	0		0	0
no UR	0		0	0
UR	1023	0.939		1076
0	0			
no UR	0		0	0
no UR	0		0	0
UR	1032	0.946		1062
UR	1046	0.993		1074
0	0			
UR	1031	0.791		1076
no UR	0		0	0
no UR	0		0	0
no UR	0		0	0
no UR	0		0	0
UR	1057	0.92		1095
UR	1039	0.921		1074
no UR	0		0	0
UR	1044	0.938		1074
UR	1052	0.761		1114
UR	1028	0.942		1071
UR	1029	0.938		1074
UR	1027	0.929		1072
UR	1012	0.807		1070
UR	1065	0.679		1110
UR	1020	0.822		1070
UR	1062	0.76		1089
UR	1048	0.703		1097
UR	1042	0.404		1103
0	0			
UR	1042	0.896		1099
UR	1016	1.028		1052
UR	1035	0.566		1095
UR	1018	1.071		1082
UR	1020	1.07		1074
UR	1020	1.118		1057
UR	1012	0.972		1056
UR	1035	1.014		1065
UR	1029	0.852		1074
UR	1037	0.648		1086
UR	1022	0.969		1067
UR	1022	1.577		1076
UR	1037	0.929		1089
UR	1022	1.196		1054
UR	1026		1	1085

UR	1029	1.232		1061
UR	1020	1.223		1080
UR	1031	0.937		1086
UR	1022	0.99		1089
UR	1029	1.692		1081
UR	1029	1.436		1069
UR	1045	0.87		1091
UR	1025	1.781		1063
UR	1069	1.364		1121
UR	1027	1.038		1086
no UR	0		0	0
UR	1025	1.169		1071
0	0			
UR	1014	0.873		1061
UR	1012	0.768		1061
UR	1016	1.209		1059
UR	1016	0.398		1052
UR	1041	1.51		1086
UR	1026	1.096		1056
UR	1016	0.959		1052
no UR	0		0	0
no UR	0		0	0
UR	1034	1.044		1062
no UR	0		0	0
no UR	0		0	0
UR	1025	1.273		1099
UR	1028	1.41		1082
no UR	0		0	0
no UR	0		0	0
0	0			
no UR	0		0	0
0	0			
no UR	0		0	0
UR	1011	1.338		1090
no UR	0		0	0
UR	1031	1.067		1066
no UR	0		0	0
no UR	0		0	0
0	0			
no UR	0		0	0
no UR	0		0	0
no UR	0		0	0
UR	1031	1.605		1093
UR	1033	1.502		1069
UR	1027	1.949		1130
UR	1042	1.218		1091
no UR	0		0	0
no UR	0		0	0
no UR	0		0	0
no UR	0		0	0

UR	1052	1.537		1116
UR	1035	1.25		1073
no UR	0		0	0
UR	1046	1.039		1101
UR	1035	1.349		1072
UR	1019	1.446		1060
UR	1018	1.109		1077
UR	1035	1.313		1086
UR	1036	1.182		1076
UR	1028	1.255		1090
UR	1016	1.153		1062
UR	1016	1.154		1054
UR	1020	1.295		1054
UR	1026	1.431		1057
0	0			
UR	1031	1.323		1072
UR	1031	1.247		1071
UR	1039	1.431		1069
UR	1039	1.325		1092
UR	1042	1.241		1092
UR	1023	1.153		1088
UR	1033	1.225		1083
UR	1019	1.427		1073
UR	1023	1.161		1087
UR	1041	1.027		1091
UR	1041	1.026		1084
UR	1037	1.08		1097
0	0			
UR	1029	1.086		1057
UR	1026	1.131		1060
UR	1029	1.048		1061
UR	1011	1.095		1071
UR	1028	1.019		1059
UR	1041	0.897		1091
UR	1031	1.155		1066
0	0			
0	0			
UR	1031	1.059		1076
UR	1025	0.903		1089
UR	1036	0.869		1070
no UR	0		0	0
no UR	0		0	0
no UR	0		0	0
no UR	0		0	0
UR	1030	0.83		1065
UR	1039	0.965		1079
UR	1027	1.027		1093
UR	1013	0.892		1067
UR	1024	1.027		1055
UR	1025	0.977		1068

no UR	0		0	0
no UR	0		0	0
UR	1030	1.184		1070
no UR	0		0	0
no UR	0		0	0
UR	1035	1.011		1068
UR	1034	0.929		1066
no UR	0		0	0
no UR	0		0	0
UR	1027	1.071		1068
no UR	0		0	0
0	0			
no UR	0		0	0
UR	1024	0.951		1089
no UR	0		0	0
UR	1039	1.032		1086
0	0			
no UR	0		0	0
UR	1043	0.962		1100
no UR	0		0	0
no UR	0		0	0
no UR	0		0	0
UR	1034	1.16		1068
UR	1029	1.034		1067
UR	1033	0.842		1063
UR	1027	0.936		1065
no UR	0		0	0
no UR	0		0	0
no UR	0		0	0
no UR	0		0	0
UR	1023	1.133		1054
UR	1029	1.137		1054
no UR	0		0	0
UR	1024	1.208		1049
0	0			
UR	1019	1.204		1041
0	0			
UR	1026	1.269		1050
UR	1019	1.204		1044
UR	1017	1.249		1042
UR	1010	1.089		1044
UR	1020	1.135		1050
UR	1022	1.066		1046
UR	1027	1.115		1050
UR	1017	1.07		1040
UR	1025	1.107		1049
UR	1010	1.014		1031
0	0			
UR	1023	1.151		1050
UR	1014	1.201		1046

UR	1023	1.092		1050
UR	1031	1.072		1063
UR	1009	1.004		1055
UR	1016	0.996		1037
UR	1016	1.025		1040
UR	1016	1.102		1046
UR	1016	0.969		1035
UR	1014	1.061		1037
UR	1022	1.071		1047
UR	1008	1.074		1044
UR	1020	0.973		1054
UR	1028	1.049		1059
UR	1014	1.158		1050
UR	1031	1.001		1085
0	0			
UR	1048	0.991		1086
UR	1020	0.98		1061
UR	1023	1.036		1054
0	0			
UR	1034	1.128		1064
no UR	0		0	0
UR	1018	1.126		1067
UR	1027	1.053		1061
UR	1033	1.134		1065
UR	1023	1.115		1055
UR	1036		1	1068
0	0			
UR	1035	1.043		1068
UR	1026	0.998		1062
UR	1011	1.006		1051
0	0			
0	0			
0	0			
no UR	0		0	0
no UR	0		0	0
UR	1023	1.061		1075
UR	1020	1.056		1048
no UR	0		0	0
0	0			
UR	1022	1.146		1049
0	0			
UR	1016	1.047		1043
no UR	0		0	0
UR	1010	1.04		1052
no UR	0		0	0
UR	1028	1.011		1059
no UR	0		0	0
no UR	0		0	0
UR	1023	1.047		1064
0	0			

no UR	0		0	0
no UR	0		0	0
UR	1034	0.989		1067
UR	1028	0.95		1060
UR	1033	0.933		1070
UR	1035	0.962		1064
no UR	0		0	0
no UR	0		0	0
no UR	0		0	0
no UR	0		0	0
UR	1031	0.96		1060
UR	1033	1.894		1111
no UR	0		0	0
UR	1022	2.089		1110
UR	1039	2.446		1101
UR	1020	1.193		1071
UR	1012	1.473		1078
UR	1014	1.802		1057
UR	1014	1.072		1061
UR	1020	1.239		1086
UR	1014	1.804		1065
UR	1022	1.318		1059
0	0			
UR	1019	1.043		1059
UR	1018	0.607		1059
UR	1005	0.723		1063
UR	1014	0.517		1069
UR	1037	0.801		1093
UR	1031	0.887		1083
UR	1016	1.104		1057
UR	1031	0.939		1091
UR	1010	1.348		1059
UR	1020	0.561		1065
UR	1026	1.513		1081
UR	1033	0.967		1082
UR	1027	1.247		1061
UR	1019	0.915		1063
UR	1025	1.308		1068
UR	1011	0.922		1092
UR	1019	1.148		1067
UR	1016	1.05		1066
UR	1033	0.643		1080
UR	1027	0.579		1093
UR	1027	1.218		1069
0	0			
UR	1014	2.171		1086
UR	1020	0.467		1067
UR	1020	1.217		1071
0	0			
UR	1035	0.914		1089

no UR	0		0	0
UR	1012	1.115		1059
UR	1008	0.774		1057
UR	1029	1.139		1078
UR	1018	1.279		1067
UR	1031	0.554		1071
UR	1010	0.612		1080
UR	1025	0.684		1072
UR	1012	1.332		1054
UR	1029	1.137		1074
no UR	0		0	0
no UR	0		0	0
UR	1027	0.732		1084
no UR	0		0	0
no UR	0		0	0
UR	1023	0.861		1076
UR	1025	1.213		1073
0	0			
no UR	0		0	0
UR	1026	0.878		1085
no UR	0		0	0
UR	1018	1.48		1080
no UR	0		0	0
UR	1014	1.01		1065
no UR	0		0	0
UR	1026	0.733		1086
no UR	0		0	0
no UR	0		0	0
UR	1025	1.14		1093
no UR	0		0	0
no UR	0		0	0
no UR	0		0	0
UR	1030	0.891		1084
UR	1022	1.554		1070
0	0			
UR	1008	0.745		1050
no UR	0		0	0
no UR	0		0	0
0	0			
0	0			
UR	1021	1.383		1075
UR	1012	1.466		1061
no UR	0		0	0
UR	1033	1.586		1096
UR	1025	1.453		1081
UR	1024	1.48		1063
UR	1015	1.454		1062
UR	1026	1.479		1071
UR	1025	1.257		1069
UR	1012	1.315		1044

UR	1039	0.856		1095
UR	1016	1.304		1057
UR	1017	1.327		1058
UR	1022	1.056		1061
UR	1018	1.205		1050
UR	1018	1.258		1059
UR	1020	1.059		1054
UR	1025		1	1059
UR	1012	1.003		1048
UR	1029	0.903		1076
UR	1016	1.062		1044
UR	1027	1.201		1074
UR	1008	1.219		1044
UR	1020	1.314		1068
UR	1014	1.125		1065
UR	1037	0.748		1069
UR	1033	0.624		1079
UR	1025	0.886		1079
UR	1018	0.752		1065
UR	1030	1.382		1093
UR	1048	0.688		1110
UR	1027	1.283		1074
UR	1045	1.324		1098
UR	1030	1.266		1071
UR	1025	1.121		1132
UR	1025	1.3		1062
UR	1025	1.252		1061
UR	1016	1.388		1057
UR	1027	0.958		1059
UR	1025	1.091		1061
no UR	0		0	0
UR	1020	1.419		1062
UR	1032	1.242		1092
UR	1022	1.243		1065
UR	1014	1.17		1057
UR	1016	0.89		1057
UR	1026	0.904		1080
UR	1027	1.307		1084
UR	1014	0.983		1057
UR	1024	1.562		1068
no UR	0		0	0
no UR	0		0	0
UR	1020	1.028		1071
no UR	0		0	0
no UR	0		0	0
UR	1037	1.26		1078
UR	1014	0.953		1061
no UR	0		0	0
no UR	0		0	0
UR	1034	1.03		1099

no UR	0		0	0
UR	1038	1.392		1095
no UR	0		0	0
UR	1007	1.009		1069
no UR	0		0	0
UR	1020	1.312		1057
no UR	0		0	0
no UR	0		0	0
UR	1025	1.354		1078
no UR	0		0	0
no UR	0		0	0
no UR	0		0	0
UR	1018	1.097		1054
UR	1027	1.108		1067
UR	1005	1.034		1060
UR	1025	0.636		1067
no UR	0		0	0
no UR	0		0	0
no UR	0		0	0
no UR	0		0	0
UR	1039	1.369		1097
UR	1018	1.359		1065
no UR	0		0	0
UR	1023	1.379		1064
UR	1024	1.437		1047
UR	1012	1.255		1046
UR	1029	1.001		1033
UR	1020	1.524		1042
0	0			
UR	1027	1.205		1063
UR	1023	1.237		1048
UR	1025	0.423		1050
UR	1033	1.022		1061
UR	1010	0.98		1044
UR	1029	0.984		1065
UR	1033	1.048		1058
UR	1033	1.028		1064
UR	1022	1.058		1060
UR	1016	1.22		1044
UR	1037	1.052		1069
UR	1035	1.021		1065
UR	1020	1.02		1025
UR	1027	1.155		1059
UR	1016	1.08		1059
UR	1039	1.198		1071
UR	1031	1.037		1065
UR	1026	1.123		1065
UR	1019	0.882		1043
UR	1033	0.985		1048
UR	1029	0.891		1060

UR	1046	0.943		1076
UR	1025	0.988		1065
UR	1018	0.899		1048
UR	1014	1.033		1044
UR	1022	1.028		1057
UR	1031	1.024		1061
UR	1027	0.94		1058
UR	1044	0.776		1054
UR	1001	0.924		1026
UR	1012	0.92		1042
no UR	0		0	0
0	0			
UR	1017	0.906		1042
UR	1034	0.876		1058
UR	1012	1.075		1047
UR	1005	0.566		1030
UR	1056	0.518		1073
0	0			
UR	1036	0.454		1040
UR	1033	0.385		1036
no UR	0		0	0
0	0			
UR	1051	0.822		1094
no UR	0		0	0
no UR	0		0	0
UR	1002	0.994		1050
UR	1086	0.47		1124
no UR	0		0	0
no UR	0		0	0
UR	1076	0.517		1125
no UR	0		0	0
UR	1011	0.651		1033
no UR	0		0	0
UR	1012	0.685		1025
no UR	0		0	0
UR	1079	0.573		1116
no UR	0		0	0
no UR	0		0	0
UR	1025	0.709		1034
no UR	0		0	0
no UR	0		0	0
no UR	0		0	0
UR	1007	0.77		1021
0	0			
UR	1001	0.376		1025
UR	1068	0.461		1074
no UR	0		0	0
no UR	0		0	0
no UR	0		0	0
no UR	0		0	0

UR	1029	0.791		1057
UR	1035	2.344		1123
no UR	0		0	0
UR	1035	1.74		1084
0	0			
UR	1032	1.32		1089
UR	1018	0.289		1061
UR	1037	0.519		1086
UR	1023	0.731		1091
UR	1042	0.254		1097
UR	1034	0.408		1067
UR	1040	0.386		1065
UR	1027	1.41		1062
UR	1022	0.587		1061
0	0			
UR	1027	0.874		1099
UR	1021	0.539		1061
UR	1029	0.723		1077
UR	1027	0.839		1065
UR	1022	0.383		1057
UR	1025	0.206		1071
UR	1031	-0.01		1063
UR	1037	0.301		1076
UR	1022	0.178		1059
UR	1035	0.22		1078
UR	1014		1	1057
UR	1018	1.192		1050
UR	1010	0.933		1050
0	0			
UR	1030	1.026		1070
UR	1033	1.424		1084
UR	1039	0.502		1079
UR	1023	0.495		1068
0	0			
0	0			
UR	1022	0.339		1061
UR	1025	0.191		1069
0	0			
UR	1039	0.15		1095
UR	1029	0.324		1080
no UR	0		0	0
UR	1026	0.812		1071
UR	1042	1.926		1069
UR	1035	0.348		1089
UR	1027	0.423		1093
0	0			
UR	1039	0.478		1091
UR	1042	0.665		1095
UR	1010	2.008		1042
UR	1051	0.993		1109

no UR	0		0	0
no UR	0		0	0
UR	1049	0.398		1102
0	0			
no UR	0		0	0
UR	1031	0.92		1103
UR	1045	0.887		1098
no UR	0		0	0
no UR	0		0	0
UR	1027	1.109		1071
no UR	0		0	0
UR	1042	0.545		1095
no UR	0		0	0
UR	1042	1.479		1101
no UR	0		0	0
UR	1056	1.922		1141
no UR	0		0	0
no UR	0		0	0
UR	1001	1.889		1084
no UR	0		0	0
no UR	0		0	0
0	0			
UR	1039	0.475		1123
UR	1012	2.354		1110
UR	1003	2.052		1080
UR	1035	0.317		1093
no UR	0		0	0
no UR	0		0	0
no UR	0		0	0
no UR	0		0	0
UR	1040	1.357		1106
UR	1038	1.301		1062
0	0			
UR	1036	1.258		1061
0	0			
UR	1022	1.019		1057
UR	1023	1.17		1050
UR	1018	1.205		1052
UR	1018	0.991		1057
0	0			
UR	1037	0.839		1061
UR	1016	1.079		1057
UR	1022	1.119		1050
UR	1018	1.104		1046
UR	1018	0.96		1061
UR	1022	1.112		1050
UR	1012	1.011		1052
UR	1025	1.15		1065
UR	1020	0.824		1054
UR	1027	0.786		1069

UR	1029	0.874		1059
UR	1044	0.832		1069
UR	1037	0.949		1065
UR	1027	1.105		1067
UR	1025	0.98		1054
UR	1022	0.853		1061
UR	1037	1.021		1071
UR	1035	0.764		1067
UR	1035	0.883		1067
UR	1022	0.905		1064
UR	1018	0.784		1052
UR	1022	0.897		1052
UR	1049	1.069		1089
UR	1016	0.772		1071
UR	1040	1.147		1078
UR	1042	0.841		1076
UR	1016	0.919		1057
UR	1020	0.866		1057
UR	1025	1.022		1076
UR	1029	0.852		1074
no UR	0		0	0
UR	1029	1.321		1071
UR	1026	1.179		1070
UR	1035	0.842		1063
UR	1031	1.145		1069
UR	1027	0.989		1065
UR	1027	0.96		1067
UR	1019	1.022		1066
0	0			
UR	1031	0.76		1074
no UR	0		0	0
no UR	0		0	0
UR	1037	1.128		1065
no UR	0		0	0
no UR	0		0	0
0	0			
UR	1025	1.61		1067
no UR	0		0	0
no UR	0		0	0
UR	1050	1.165		1082
no UR	0		0	0
UR	1025	1.024		1061
no UR	0		0	0
UR	1017	1.585		1067
no UR	0		0	0
UR	1020	1.326		1050
no UR	0		0	0
no UR	0		0	0
UR	1029	1.151		1058
no UR	0		0	0

no UR	0		0	0
no UR	0		0	0
UR	1012	1.145		1048
UR	1041	1.228		1072
UR	1012	1.177		1070
UR	1036	1.133		1078
no UR	0		0	0
no UR	0		0	0
no UR	0		0	0
no UR	0		0	0
UR	1052	1.274		1084
UR	1040	1.048		1096
no UR	0		0	0
UR	1028	1.011		1059
UR	1025	1.126		1057
UR	1037	1.113		1081
UR	1014	1.157		1052
0	0			
UR	1021	1.052		1055
UR	1018	1.092		1059
UR	1028	0.852		1055
UR	1022	1.127		1063
0	0			
UR	1016	1.279		1052
UR	1039	1.079		1081
UR	1022	1.051		1059
UR	1037	1.063		1072
UR	1029	1.219		1063
UR	1027	1.113		1071
0	0			
UR	1025	0.996		1082
UR	1027	1.086		1056
UR	1026	1.067		1069
UR	1039	1.137		1072
UR	1018	0.971		1026
UR	1031	0.934		1064
UR	1039	1.11		1078
UR	1027	1.094		1074
UR	1029	1.02		1061
UR	1024	1.106		1060
UR	1024	1.093		1058
UR	1029	1.078		1059
UR	1036	1.012		1080
UR	1033	0.973		1066
UR	1027	1.085		1061
UR	1029	1.01		1058
UR	1031	0.954		1059
UR	1016	0.833		1050
UR	1020	1.208		1060
UR	1046	0.914		1089

0	0			
UR	1017	0.987		1068
UR	1029	1.045		1071
UR	1005	0.895		1078
UR	1020	1.209		1059
UR	1038	0.753		1090
UR	1008	0.917		1059
UR	1013	1.117		1069
UR	1012	0.911		1033
UR	1012	0.966		1063
no UR	0		0	0
no UR	0		0	0
UR	1001	1.054		1021
0	0			
no UR	0		0	0
UR	1017	0.994		1070
UR	1005	0.896		1063
no UR	0		0	0
no UR	0		0	0
UR	1049	0.985		1089
no UR	0		0	0
UR	1019	0.991		1023
no UR	0		0	0
0	0			
no UR	0		0	0
UR	1014	1.03		1022
no UR	0		0	0
no UR	0		0	0
UR	1004	1.119		1052
no UR	0		0	0
no UR	0		0	0
no UR	0		0	0
UR	1017	0.931		1024
UR	1022	1.035		1082
UR	1021	1.056		1033
UR	1014	1.074		1024
no UR	0		0	0
no UR	0		0	0
no UR	0		0	0
no UR	0		0	0
UR	1025	1.14		1094
UR	1055	1.986		1144
no UR	0		0	0
0	0			
UR	1029	1.525		1093
UR	1016	2.19		1074
UR	1016	1.882		1059
UR	1029	1.731		1065
UR	1033	1.071		1076
UR	1027	1.218		1076

0	0			
UR	1037	1.027		1099
UR	1016	1.048		1071
UR	1039	1.244		1076
UR	1037	0.725		1088
UR	1033	0.697		1082
UR	1026	1.268		1084
UR	1039	1.627		1080
UR	1031	0.728		1084
UR	1031	2.369		1110
0	0			
0	0			
UR	1039	0.926		1101
UR	1022	1.793		1084
UR	1042	0.85		1089
UR	1033	0.679		1078
UR	1018	0.567		1065
UR	1027	0.724		1067
UR	1020	1.883		1070
UR	1031	1.285		1078
UR	1025	0.899		1076
UR	1031	0.393		1024
0	0			
UR	1029	0.368		1022
UR	1027	0.869		1063
UR	1008	1.079		1052
UR	1020	1.131		1078
UR	1016	0.919		1069
UR	1035	1.148		1071
UR	1020	1.277		1061
no UR	0		0	0
UR	1014	1.284		1052
0	0			
UR	1029	0.11		1069
UR	1029	0.516		1080
0	0			
UR	1012	1.301		1057
0	0			
UR	1018	1.317		1059
UR	1022	0.171		1082
no UR	0		0	0
no UR	0		0	0
UR	1027	0.83		1061
no UR	0		0	0
no UR	0		0	0
UR	1025	0.889		1076
UR	1022	0.91		1052
no UR	0		0	0
no UR	0		0	0
UR	1022	1.186		1064

no UR	0	0	0
UR	1025	0.48	1059
0	0		
UR	1012	1.332	1061
no UR	0	0	0
UR	1033	1.168	1076
no UR	0	0	0
no UR	0	0	0
UR	1022	1.393	1061
0	0		
no UR	0	0	0
no UR	0	0	0
0	0		
0	0		
UR	1016	0.687	1059
UR	1008	0.735	1063
no UR	0	0	0
0	0		
0	0		
no UR	0	0	0
UR	1020	0.208	1071
0	0		
0	0		
UR	1025	1.345	1056
0	0		
UR	1033	1.13	1069
UR	1022	0.897	1059
0	0		
UR	1029	0.837	1065
UR	1032	1.136	1095
UR	1020	1.112	1063
0	0		
0	0		
UR	1027	0.917	1065
0	0		
UR	1017	1.084	1050
UR	1031	1.054	1074
UR	1017	1.263	1055
0	0		
UR	1021	1.32	1057
UR	1027	1.153	1061
UR	1025	1.049	1063
UR	1022	1.237	1060
UR	1048	0.9	1069
0	0		
UR	1018	0.88	1055
0	0		
UR	1034	1.096	1086
UR	1005	0.915	1059
UR	1020	0.953	1066

UR	1016	0.94		1061
UR	1022	0.814		1067
UR	1022	0.871		1067
0	0			
UR	1006	0.98		1055
0	0			
UR	1031	0.927		1078
UR	1014	0.928		1033
UR	1039	0.826		1078
0	0			
0	0			
UR	1040	1.163		1099
UR	1021	0.821		1091
UR	1037	0.902		1091
UR	1040	1.199		1088
UR	1046	1.011		1084
UR	1022	0.856		1078
UR	1033	0.908		1084
UR	1025	1.111		1065
UR	1039	0.876		1084
no UR	0		0	0
no UR	0		0	0
UR	1031	1.605		1069
no UR	0		0	0
no UR	0		0	0
UR	1014	1.218		1046
UR	1025	0.879		1071
no UR	0		0	0
0	0			
UR	1031	1.095		1075
no UR	0		0	0
0	0			
0	0			
UR	1026	0.935		1062
no UR	0		0	0
0	0			
0	0			
no UR	0		0	0
0	0			
no UR	0		0	0
0	0			
0	0			
UR	1022	1.08		1060
UR	1044	0.885		1069
0	0			
0	0			
no UR	0		0	0
no UR	0		0	0
0	0			
no UR	0		0	0

UR	1028	1.015		1069
UR	1031	1.153		1065
no UR	0		0	0
UR	1064	1.372		1135
UR	1027	1.307		1071
UR	1022	1.178		1067
0	0			
UR	1049	1.023		1077
UR	1026	1.216		1060
UR	1030	1.189		1077
UR	1037	1.075		1101
UR	1037	1.109		1074
UR	1018	1.103		1065
UR	1014	1.13		1078
UR	1037	1.128		1096
0	0			
UR	1008	0.834		1062
UR	1027	1.058		1046
UR	1022	1.086		1078
0	0			
UR	1003	0.971		1081
UR	1043	1.029		1083
UR	1078	0.905		1110
UR	1037	1.033		1074
UR	1024	0.887		1057
0	0			
UR	1052	0.992		1093
UR	1027	1.145		1063
0	0			
UR	1065	0.926		1082
UR	1061	1.01		1089
0	0			
UR	1048	0.995		1074
UR	1054	0.955		1078
UR	1061	0.93		1074
0	0			
UR	1001	1.047		1044
UR	1008	0.999		1054
UR	1035	0.965		1065
UR	1057	1.072		1089
0	0			
UR	1031	0.824		1058
UR	1061	1.083		1082
UR	1061	1.031		1082
UR	1057	1.064		1084
UR	1057	0.865		1093
0	0			
UR	1054	1.026		1086
UR	1049	0.997		1088
0	0			

no UR	0		0	0
no UR	0		0	0
UR	1065	0.845		1086
0	0			
no UR	0		0	0
UR	1059	1.053		1091
0	0			
0	0			
no UR	0		0	0
UR	1018	0.98		1055
no UR	0		0	0
UR	1022	0.875		1050
no UR	0		0	0
0	0			
no UR	0		0	0
UR	1035	1.056		1054
no UR	0		0	0
no UR	0		0	0
0	0			
no UR	0		0	0
0	0			
0	0			
UR	1063	1.058		1095
UR	1061	1.084		1093
0	0			
UR	1069	0.929		1112
no UR	0		0	0
0	0			
no UR	0		0	0
no UR	0		0	0
UR	1036	0.631		1083
UR	1027	1.587		1061
no UR	0		0	0
UR	1014	1.649		1066
UR	1035	2.02		1065
UR	1022	1.837		1060
UR	1025	1.467		1059
UR	1025	1.518		1090
UR	1040	1.495		1092
UR	1030	1.576		1085
UR	1019	1.089		1066
0	0			
UR	1014	1.279		1065
UR	1023	1.235		1069
UR	1023	0.96		1063
UR	1019	1.279		1072
UR	1034	1.113		1115
UR	1022	0.708		1078
UR	1025	0.947		1082
UR	1029	0.908		1074

UR	1017	1.103		1070
UR	1018	1.203		1063
UR	1033	1.034		1083
UR	1027	1.368		1061
UR	1018	0.986		1102
UR	1012	0.939		1069
UR	1029	1.005		1093
UR	1038	0.957		1095
UR	1042	1.07		1117
UR	1039	1.036		1113
UR	1039	0.408		1091
UR	1039	0.995		1116
UR	1053	1.057		1174
UR	1022	0.953		1086
UR	1034	0.897		1110
UR	1027	0.916		1086
UR	1012	0.91		1090
UR	1024	1.203		1072
UR	1031	0.974		1077
0	0			
no UR	0		0	0
0	0			
UR	1020	0.661		1061
UR	1034	0.715		1104
0	0			
UR	1027	0.515		1074
UR	1026	0.828		1065
UR	1026	0.912		1078
UR	1035	0.924		1091
UR	1024	1.026		1081
no UR	0		0	0
no UR	0		0	0
UR	1017	1.043		1071
no UR	0		0	0
no UR	0		0	0
UR	1027	1.179		1066
UR	1019	1.171		1158
no UR	0		0	0
no UR	0		0	0
UR	1029	1.017		1074
no UR	0		0	0
UR	1029	1.189		1078
no UR	0		0	0
UR	1029	1.013		1063
no UR	0		0	0
UR	1022	0.771		1067
no UR	0		0	0
no UR	0		0	0
UR	1041	0.712		1087
no UR	0		0	0

no UR	0		0	0
no UR	0		0	0
UR	1027	1.042		1066
UR	1020	0.335		1069
UR	1014	0.996		1091
UR	1027	0.714		1060
no UR	0		0	0
no UR	0		0	0
no UR	0		0	0
no UR	0		0	0
UR	1022	1.396		1061
UR	1025	1.411		1084
no UR	0		0	0
UR	1020	1.658		1080
UR	1018	1.852		1063
UR	1018	1.436		1061
UR	1018	0.597		1059
UR	1018	1.661		1059
UR	1020	1.095		1061
UR	1018	1.506		1059
UR	1029	0.876		1063
UR	1016	1.121		1054
UR	1016	0.864		1059
UR	1019	1.225		1056
UR	1016	1.024		1052
UR	1014	1.16		1065
UR	1022	0.731		1057
UR	1020	0.821		1054
UR	1020	0.693		1059
UR	1008	1.201		1054
UR	1018	0.752		1057
UR	1016	0.963		1052
UR	1016	1.562		1099
UR	1018	1.127		1057
UR	1020	1.278		1061
UR	1029	1.294		1090
UR	1031	1.411		1110
UR	1010	1.194		1059
UR	1022	0.769		1057
UR	1016	0.991		1056
UR	1025	1.383		1086
UR	1021	0.948		1056
UR	1027	0.871		1071
UR	1018	0.954		1052
UR	1018	0.883		1054
UR	1022	1.453		1097
UR	1024	0.811		1060
UR	1010	0.898		1057
UR	1014	0.965		1059
UR	1027	0.961		1057

no UR	0		0	0
UR	1023	1.001		1064
UR	1025	0.776		1054
UR	1020	0.999		1057
UR	1018	0.446		1057
UR	1022	0.862		1054
UR	1020	0.848		1052
UR	1012	0.824		1054
UR	1018	0.643		1052
UR	1014	0.963		1054
no UR	0		0	0
no UR	0		0	0
UR	1019	0.952		1064
no UR	0		0	0
no UR	0		0	0
UR	1018	1.387		1057
UR	1023	0.637		1054
no UR	0		0	0
no UR	0		0	0
UR	1022	1.089		1057
no UR	0		0	0
UR	1017	1.6		1059
no UR	0		0	0
UR	1025	1.231		1057
no UR	0		0	0
UR	1016	1.169		1059
no UR	0		0	0
no UR	0		0	0
UR	1018	1.135		1057
no UR	0		0	0
no UR	0		0	0
no UR	0		0	0
UR	1014	1.138		1059
UR	1024	0.638		1060
0	0			
UR	1016	0.424		1054
no UR	0		0	0
no UR	0		0	0
no UR	0		0	0
no UR	0		0	0
UR	1018	1.045		1063
UR	1014	1.004		1031
no UR	0		0	0
0	0			
UR	1138	0.534		1244
0	0			
0	0			
UR	1091	1.538		1199
UR	1065	1.077		1103
UR	1067	0.783		1110

UR	1069	0.989		1146
UR	1061	0.352		1110
UR	1067	1.068		1094
UR	1059	0.844		1092
UR	1061	1.126		1086
UR	1058	0.961		1086
UR	1059	0.901		1086
UR	1065	1.238		1093
UR	1058	0.945		1095
UR	1031	0.495		1084
UR	1044	0.304		1081
UR	1067	0.847		1089
UR	1055	0.483		1086
UR	1068	0.429		1104
UR	1062	0.738		1113
UR	1067	0.128		1155
UR	1058	0.455		1093
0	0			
UR	1056	0.693		1086
UR	1059	0.74		1110
UR	1054	1.193		1093
UR	1054	0.715		1089
UR	1055	1.039		1081
UR	1048	0.697		1071
UR	1056	0.962		1083
0	0			
UR	1053	1.034		1083
UR	1053	0.806		1086
UR	1052	0.839		1090
UR	1046	0.507		1074
0	0			
UR	1049	0.826		1072
UR	1051	1.018		1075
UR	1054	0.74		1084
UR	1056	0.911		1086
UR	1038	0.883		1062
UR	1051	0.53		1078
UR	1057	0.586		1088
UR	1054	0.45		1086
UR	1053	0.737		1089
no UR	0		0	0
no UR	0		0	0
UR	1057	0.743		1082
no UR	0		0	0
no UR	0		0	0
0	0			
UR	1061	0.601		1086
no UR	0		0	0
no UR	0		0	0
0	0			

0	0			
UR	1080	0.434		1110
no UR	0		0	0
UR	1064	0.766		1091
no UR	0		0	0
UR	1062	0.623		1086
no UR	0		0	0
no UR	0		0	0
0	0			
no UR	0		0	0
no UR	0		0	0
no UR	0		0	0
UR	1067	0.266		1095
UR	1063	0.727		1093
UR	1078	0.913		1110
UR	1078	0.581		1110
no UR	0		0	0
0	0			
0	0			
no UR	0		0	0
UR	1029	0.749		1050
UR	1037	1.349		1061
no UR	0		0	0
UR	1029	1.34		1052
UR	1018	1.113		1046
UR	1025	1.094		1052
UR	1020	1.267		1057
UR	1024	1.315		1052
UR	1035	1.46		1072
UR	1044	1.287		1092
UR	1022	1.401		1057
UR	1029	1.016		1057
UR	1020	1.151		1048
UR	1029	1.149		1044
UR	1010	1.2		1054
UR	1018	1.108		1054
UR	1020	1.081		1042
UR	1016	1.301		1031
UR	1010	1.458		1069
UR	1018	1.368		1039
UR	1020	1.444		1054
UR	1018	1.324		1037
UR	1016	1.323		1079
UR	1023	1.472		1077
UR	1018	1.385		1048
UR	1018	1.3		1054
UR	1031	1.269		1046
UR	1018	1.126		1035
UR	1034	1.322		1084
UR	1033	1.263		1065

UR	1022	1.496		1076
UR	1031	1.481		1078
UR	1014	1.194		1053
UR	1022	1.123		1076
UR	1013	1.238		1042
UR	1022	1.131		1044
UR	1010	1.176		1044
UR	1016	0.842		1087
UR	1020	1.111		1042
UR	1016	1.225		1046
no UR	0		0	0
UR	1039	1.206		1097
UR	1046	1.142		1082
UR	1033	1.344		1078
UR	1029	1.172		1054
UR	1016	1.381		1027
UR	1014	1.332		1037
UR	1016	1.196		1044
0	0			
UR	1017	1.455		1042
no UR	0		0	0
no UR	0		0	0
UR	1017	1.367		1050
no UR	0		0	0
no UR	0		0	0
UR	1016	1.026		1037
UR	1014	1.428		1048
0	0			
no UR	0		0	0
0	0			
no UR	0		0	0
UR	1020	1.262		1054
no UR	0		0	0
UR	1020	1.011		1048
no UR	0		0	0
0	0			
no UR	0		0	0
no UR	0		0	0
UR	1035	1.319		1061
no UR	0		0	0
no UR	0		0	0
0	0			
UR	1086	0.422		1150
UR	1035	0.259		1059
UR	1033	0.538		1050
UR	1035	0.383		1052
0	0			
no UR	0		0	0
no UR	0		0	0
no UR	0		0	0

UR	1035	0.647		1052
0	0			
no UR	0		0	0
0	0			
UR	1025	0.968		1057
UR	1023	1.121		1052
UR	1033	0.927		1061
UR	1016	1.075		1044
0	0			
UR	1001	0.828		1037
UR	1054	0.929		1078
UR	1057	1.014		1084
UR	1020	0.987		1027
UR	1025	0.984		1027
UR	1044	0.977		1074
0	0			
UR	1044	0.897		1065
UR	1020	0.997		1056
0	0			
UR	1033	1.021		1074
UR	1011	1.055		1037
UR	1056	1.05		1091
UR	1027	0.953		1069
0	0			
UR	1050	1.146		1084
UR	1027	1.015		1074
UR	1052	0.875		1095
UR	1063	1.101		1084
UR	1057	0.965		1123
UR	1033	0.869		1110
UR	1048	0.909		1089
UR	1016	1.019		1048
UR	1044	1.03		1078
UR	1044	1.082		1067
UR	1037	0.995		1078
UR	1027	1.058		1062
UR	1016	1.02		1042
UR	1039	1.099		1065
UR	1021	1.011		1045
UR	1028	0.948		1061
0	0			
UR	1016	1.025		1061
UR	1044	1.553		1080
UR	1048	1.202		1078
UR	1046	1.027		1078
UR	1044	1.114		1065
UR	1040	1.106		1074
UR	1035	1.151		1080
UR	1033	1.239		1071
UR	1028	1.105		1073

no UR	0		0	0
no UR	0		0	0
UR	1039	1.029		1072
no UR	0		0	0
no UR	0		0	0
UR	1026	1.041		1055
0	0			
no UR	0		0	0
no UR	0		0	0
UR	1035	0.946		1093
no UR	0		0	0
0	0			
no UR	0		0	0
UR	1012	1.039		1039
no UR	0		0	0
UR	1061	1.098		1089
no UR	0		0	0
no UR	0		0	0
UR	1067	1.074		1093
no UR	0		0	0
0	0			
no UR	0		0	0
UR	1016	0.917		1048
UR	1065	0.929		1110
UR	1039	1.045		1097
UR	1025	0.992		1029
no UR	0		0	0
no UR	0		0	0
no UR	0		0	0
no UR	0		0	0
UR	1022	0.951		1082
UR	1021	1.226		1048
no UR	0		0	0
0	0			
0	0			
UR	1020	1.058		1080
UR	1018	1.08		1078
UR	1020	0.945		1051
UR	1010	1.021		1063
UR	1018	0.996		1049
UR	1020	0.962		1046
UR	1010	1.007		1069
UR	1016	0.948		1069
UR	1014	1.013		1069
UR	1027	1.073		1074
UR	1018	1.022		1042
UR	1022	0.9		1050
UR	1022	0.924		1048
UR	1022	1.105		1059
UR	1020	1.03		1047

UR	1016	0.996		1074
UR	1023	1.032		1064
UR	1027	0.997		1059
UR	1033	1.009		1082
UR	1018	0.942		1057
UR	1012	1.008		1071
UR	1022	0.914		1057
UR	1020	1.038		1066
UR	1024	1.128		1079
UR	1016	1.084		1069
UR	1012	1.033		1071
UR	1023	1.121		1082
UR	1021	1.117		1057
UR	997	1.092		1076
UR	1044	0.827		1088
UR	1011	1.071		1066
UR	1012	0.812		1059
UR	1030	1.002		1081
UR	1016	0.805		1065
0	0			
no UR	0		0	0
UR	1020	1.082		1074
0	0			
UR	1026	1.154		1078
0	0			
UR	1016	0.95		1059
0	0			
UR	1020	0.962		1063
UR	1027	1.013		1076
UR	1022	1.144		1071
0	0			
no UR	0		0	0
UR	1018	1.03		1076
no UR	0		0	0
no UR	0		0	0
UR	1020	1.092		1089
UR	1022	0.928		1063
no UR	0		0	0
no UR	0		0	0
UR	1022	1.06		1054
no UR	0		0	0
UR	1023	1.014		1060
no UR	0		0	0
UR	1013	0.717		1062
0	0			
UR	1020	0.951		1078
no UR	0		0	0
no UR	0		0	0
UR	1017	0.997		1075
0	0			

no UR	0		0	0
no UR	0		0	0
UR	1019	0.986		1071
UR	1020	0.939		1071
UR	1018	1.062		1110
UR	1031	1.003		1084
no UR	0		0	0
no UR	0		0	0
no UR	0		0	0
no UR	0		0	0
UR	1045	0.939		1108
UR	1022	1.422		1057
no UR	0		0	0
UR	1031	1.053		1054
UR	1020	1.109		1044
UR	1013	1.082		1035
UR	1024	1.14		1061
UR	1011	1.115		1031
UR	1003	1.077		1027
UR	1018	1.183		1039
UR	1016	1.018		1031
UR	1017	1.076		1060
UR	1031	1.024		1054
UR	1014	1.148		1039
0	0			
0	0			
UR	1016	1.1		1044
UR	1020	1.099		1052
UR	1012	1.195		1044
UR	1027		1	1044
UR	1014	1.111		1035
UR	1018	1.095		1042
UR	1025	0.992		1059
UR	1027	0.535		1039
UR	1018	1.073		1039
UR	1020	1.101		1048
UR	1014	1.113		1039
UR	1008	0.977		1035
UR	1014	1.09		1037
UR	1028	1.013		1056
UR	1029	0.726		1037
UR	1010	1.188		1033
UR	1014	0.967		1035
0	0			
UR	1080	0.894		1131
UR	1089	0.878		1121
UR	1050	0.959		1097
UR	1011	1.064		1033
UR	1044	1.02		1080
UR	1022	0.919		1035

0	0			
UR	1018	0.981		1035
UR	1010	1.04		1037
UR	1016	1.2		1042
UR	1016	1.035		1037
UR	1014	1.066		1029
UR	1016	0.98		1046
UR	1050	1.154		1071
UR	1014	0.908		1035
UR	1018	0.892		1039
no UR	0		0	0
no UR	0		0	0
UR	1033	1.145		1055
no UR	0		0	0
no UR	0		0	0
UR	1025	1.215		1063
UR	1029	1.174		1065
no UR	0		0	0
no UR	0		0	0
UR	1016	1.083		1056
no UR	0		0	0
UR	1044	0.8		1077
no UR	0		0	0
UR	1033	0.986		1063
no UR	0		0	0
UR	1050	0.985		1080
no UR	0		0	0
no UR	0		0	0
UR	1037	1.144		1070
no UR	0		0	0
no UR	0		0	0
no UR	0		0	0
UR	1031	1.028		1056
UR	1028	1.007		1056
UR	1014	0.988		1035
0	0			
no UR	0		0	0
no UR	0		0	0
no UR	0		0	0
no UR	0		0	0
UR	1017	1.097		1040
UR	1020	1.649		1048
no UR	0		0	0
UR	1016	1.573		1031
UR	1022	1.333		1048
UR	1014	0.5		1035
0	0			
UR	1010	1.088		1027
UR	1018	1.629		1044
UR	1022	1.705		1044

UR	1027	1.705		1059
UR	1012	1.769		1037
UR	1018	1.105		1042
UR	1033	2.249		1060
UR	1012	1.631		1025
UR	1012	2.284		1027
UR	1031	1.994		1050
UR	1018	1.792		1044
UR	1016	1.92		1039
UR	1025	1.596		1052
UR	1025	1.289		1059
UR	1014	2.631		1020
UR	1031	1.924		1035
UR	1025	2.235		1033
UR	1027	1.847		1042
UR	1014	0.839		1031
UR	1022	1.601		1048
UR	1014	1.165		1031
UR	1010	1.162		1025
UR	1048	2.244		1074
UR	1010	1.557		1035
UR	1031	1.988		1059
UR	1012	1.614		1027
UR	1010	1.615		1031
UR	1012	1.847		1037
UR	1029	1.641		1061
UR	1054	1.426		1086
UR	1001	1.582		1027
UR	1003	1.744		1025
UR	1020	1.335		1046
no UR	0		0	0
UR	1008	1.246		1025
UR	1014	1.262		1031
UR	1018	1.612		1033
UR	1010	1.068		1025
UR	1008	1.58		1035
UR	1018	1.252		1035
UR	1010	1.304		1031
UR	1014	1.35		1035
UR	1008	1.218		1025
no UR	0		0	0
no UR	0		0	0
UR	1025	1.802		1044
no UR	0		0	0
no UR	0		0	0
UR	1010	1.144		1029
0	0			
no UR	0		0	0
no UR	0		0	0
UR	1037	1.224		1071

no UR	0		0	0
UR	1010	1.254		1031
no UR	0		0	0
UR	1031	1.497		1059
no UR	0		0	0
UR	1037	2.664		1065
no UR	0		0	0
no UR	0		0	0
UR	1029	1.32		1052
no UR	0		0	0
0	0			
no UR	0		0	0
UR	1014	1.752		1061
UR	1018	1.445		1035
UR	1010	1.484		1039
UR	1025	1.667		1044
no UR	0		0	0
no UR	0		0	0
no UR	0		0	0
no UR	0		0	0
UR	1018	1.918		1039
UR	1037	1.184		1076
no UR	0		0	0
UR	1039	1.345		1076
UR	1038	1.026		1063
UR	1044	1.073		1071
UR	1035	1.102		1067
0	0			
UR	1036	1.247		1066
UR	1037	1.156		1068
UR	1018	0.781		1080
UR	1031	1.118		1069
UR	1040	1.061		1083
UR	1034	1.087		1073
UR	1028	0.963		1076
UR	1034	1.146		1071
UR	1024	1.157		1070
UR	1024	1.125		1073
UR	1025	1.072		1084
UR	1031	1.09		1086
UR	1032	0.971		1079
UR	1030	1.14		1080
UR	1031	1.204		1074
UR	1043	1.072		1094
UR	1035	0.985		1084
UR	1037	1.209		1074
UR	1042	0.927		1084
UR	1038	0.939		1086
UR	1028	1.09		1075
UR	1034	1.06		1081

UR	1029	1.075		1075
UR	1025	0.973		1067
UR	1039	0.851		1084
0	0			
UR	1021	0.986		1076
UR	1039	0.75		1086
UR	1028	0.984		1082
UR	1023	1.028		1076
UR	1022	0.65		1083
UR	1034	0.959		1080
no UR	0		0	0
UR	1042	1.127		1081
UR	1035	0.954		1089
UR	1027	0.933		1071
UR	1039	0.934		1077
UR	1025	0.816		1080
UR	1042	0.761		1086
UR	1025	0.969		1078
UR	1031	0.905		1078
UR	1014	0.869		1069
0	0			
no UR	0		0	0
UR	1040	1.259		1086
0	0			
no UR	0		0	0
UR	1046	1.027		1080
UR	1025	0.837		1086
no UR	0		0	0
no UR	0		0	0
UR	1029	1.206		1074
no UR	0		0	0
UR	1043	1.271		1085
no UR	0		0	0
UR	1037	0.911		1078
0	0			
UR	1042	0.997		1091
no UR	0		0	0
no UR	0		0	0
UR	1044	1.155		1081
no UR	0		0	0
no UR	0		0	0
no UR	0		0	0
UR	1048	1.045		1093
UR	1033	1.129		1090
UR	1012	0.789		1074
UR	1039	0.919		1077
no UR	0		0	0
no UR	0		0	0
no UR	0		0	0
no UR	0		0	0

UR	1050	1.169		1091
UR	1055	0.877		1080
no UR	0		0	0
UR	1022	0.734		1040
UR	1029	0.812		1048
UR	1025	0.795		1043
UR	1014	0.899		1039
UR	1016	0.808		1039
UR	1018	0.842		1039
UR	1012	0.738		1031
UR	1012	0.695		1027
UR	1010	0.718		1035
UR	1016	0.861		1042
UR	1042	0.586		1065
UR	1016	0.695		1042
UR	1021	0.708		1041
UR	1012	0.674		1031
UR	1014	0.773		1039
UR	1025	0.862		1037
UR	1010	0.839		1037
UR	1022	0.795		1042
UR	1030	0.87		1052
UR	1012	0.942		1057
UR	1018	0.962		1046
UR	1018	0.913		1037
UR	1020	0.968		1037
UR	1018	0.872		1039
UR	1018	0.807		1039
UR	1018	0.815		1037
UR	1018	0.903		1037
UR	1024	0.825		1077
UR	1027	0.791		1042
UR	1016	0.826		1035
UR	1016	0.783		1039
UR	1025	0.873		1044
UR	1022	0.976		1057
UR	1018	0.818		1033
UR	1022	0.88		1050
0	0			
UR	1018	0.778		1037
no UR	0		0	0
0	0			
UR	1031	0.949		1064
0	0			
UR	1019	0.914		1055
UR	1016	0.892		1050
UR	1012	0.996		1049
UR	1010	0.828		1042
UR	1014	0.878		1043
UR	1015	0.827		1037

no UR	0		0	0
no UR	0		0	0
UR	1024	0.846		1056
no UR	0		0	0
no UR	0		0	0
UR	1014	0.734		1039
UR	1020	0.917		1042
no UR	0		0	0
no UR	0		0	0
UR	1031	1.073		1077
no UR	0		0	0
UR	1025	0.933		1050
no UR	0		0	0
UR	1020	0.785		1046
no UR	0		0	0
0	0			
no UR	0		0	0
no UR	0		0	0
UR	1020	0.923		1047
no UR	0		0	0
no UR	0		0	0
no UR	0		0	0
0	0			
UR	1015	0.717		1056
UR	1020	1.023		1057
UR	1021	0.953		1059
no UR	0		0	0
no UR	0		0	0
no UR	0		0	0
no UR	0		0	0
0	0			
UR	1018	1.214		1054
no UR	0		0	0
UR	1018	1.264		1052
0	0			
UR	1022	1.096		1059
UR	1020	1.16		1071
UR	1037	1.147		1110
UR	1022	1.265		1052
UR	1028	1.164		1100
UR	1024	1.256		1086
UR	1037	1.151		1107
UR	1022	1.3		1075
UR	1020	1.229		1065
UR	1020	1.244		1074
UR	1012	1.206		1054
UR	1031	0.998		1095
UR	1033	0.95		1077
UR	1014	1.261		1062
UR	1032	1.118		1086

UR	1033	0.874		1091
UR	1027	0.749		1074
UR	1022	0.728		1067
UR	1030	0.821		1084
UR	1032	0.761		1096
UR	1048	0.855		1094
0	0			
UR	1042	1.072		1098
UR	1023	0.841		1068
0	0			
UR	1031	0.849		1086
UR	1022	1.198		1081
UR	1022	1.07		1071
UR	1025	0.951		1059
UR	1033	0.93		1077
0	0			
UR	1024	1.087		1074
0	0			
0	0			
UR	1020	0.771		1074
no UR	0		0	0
UR	1027	1.014		1091
UR	1022	1.067		1069
UR	1020	1.195		1071
UR	1018	1.072		1070
UR	1025	0.849		1062
UR	1025	0.963		1067
UR	1020	1.028		1065
UR	1021	0.905		1068
UR	1038	0.913		1100
no UR	0		0	0
0	0			
UR	1018	0.976		1057
0	0			
no UR	0		0	0
UR	1022	1.283		1077
0	0			
no UR	0		0	0
no UR	0		0	0
UR	1024	0.952		1053
no UR	0		0	0
UR	1022	1.223		1080
no UR	0		0	0
UR	1024	1.124		1070
no UR	0		0	0
0	0			
0	0			
no UR	0		0	0
UR	1020	1.028		1069
0	0			

no UR	0		0	0
no UR	0		0	0
0	0			
UR	1018	1.153		1074
0	0			
UR	1021	0.707		1063
no UR	0		0	0
no UR	0		0	0
0	0			
0	0			
UR	1029	1.073		1086
UR	1028	1.276		1055
no UR	0		0	0
UR	1040	1.257		1080
UR	1023	1.115		1061
UR	1021	1.068		1062
UR	1024	1.103		1057
UR	1039	1.145		1070
UR	1046	0.967		1097
UR	1027	1.059		1064
UR	1026	1.275		1061
no UR	0		0	0
UR	1035	1.008		1086
UR	1022	1.195		1064
UR	1038	1.22		1076
0	0			
UR	1037	1.063		1067
UR	1031	1.202		1059
UR	1028	1.262		1059
UR	1026	1.135		1062
UR	1023	1.17		1048
UR	1021	1.16		1050
0	0			
UR	1039	1.187		1069
UR	1028	1.247		1076
UR	1033	1.194		1074
UR	1018	0.918		1025
UR	1021	1.126		1057
UR	1031	1.383		1059
0	0			
UR	1039	1.184		1078
UR	1029	0.835		1082
UR	1031	1.258		1067
UR	1018	1.449		1057
UR	1026	1.273		1053
UR	1028	1.321		1058
UR	1025	1.311		1060
UR	1046	0.961		1084
UR	1028	1.148		1074
UR	1027	1.242		1079

no UR	0		0	0
UR	1035	1.279		1073
UR	1016	0.912		1065
UR	1028	1.083		1059
UR	1025	1.012		1064
UR	1028	1.257		1057
UR	1021	0.449		1062
UR	1033	1.148		1083
UR	1019	1.196		1112
UR	1029	1.107		1065
no UR	0		0	0
no UR	0		0	0
UR	1033	0.901		1081
no UR	0		0	0
no UR	0		0	0
UR	1027	1.163		1062
UR	1027	1.398		1079
no UR	0		0	0
no UR	0		0	0
UR	1027	1.18		1079
no UR	0		0	0
UR	1019	1.26		1056
no UR	0		0	0
UR	1021	1.073		1067
no UR	0		0	0
0	0			
no UR	0		0	0
no UR	0		0	0
UR	1027	1.189		1070
no UR	0		0	0
no UR	0		0	0
no UR	0		0	0
UR	1036	1.089		1090
UR	1031	1.113		1067
UR	1032	1.195		1071
UR	1029	1.176		1064
no UR	0		0	0
no UR	0		0	0
no UR	0		0	0
no UR	0		0	0
UR	1036	1.262		1068
